# AJICAP Second Generation: Improved Chemical Site-Specific Conjugation Technology for Antibody–Drug Conjugate Production

**DOI:** 10.1021/acs.bioconjchem.3c00040

**Published:** 2023-03-09

**Authors:** Tomohiro Fujii, Yutaka Matsuda, Takuya Seki, Natsuki Shikida, Yusuke Iwai, Yuri Ooba, Kazutoshi Takahashi, Muneki Isokawa, Sayaka Kawaguchi, Noriko Hatada, Tomohiro Watanabe, Rika Takasugi, Akira Nakayama, Kazutaka Shimbo, Brian A. Mendelsohn, Tatsuya Okuzumi, Kei Yamada

**Affiliations:** † Ajinomoto Co., Inc., 1-1, Suzuki-Cho, Kawasaki-Ku, Kawasaki-Shi, Kanagawa 210-8681, Japan; ‡ Ajinomoto Bio-Pharma Services, 11040 Roselle Street, San Diego, California 92121, United States

## Abstract

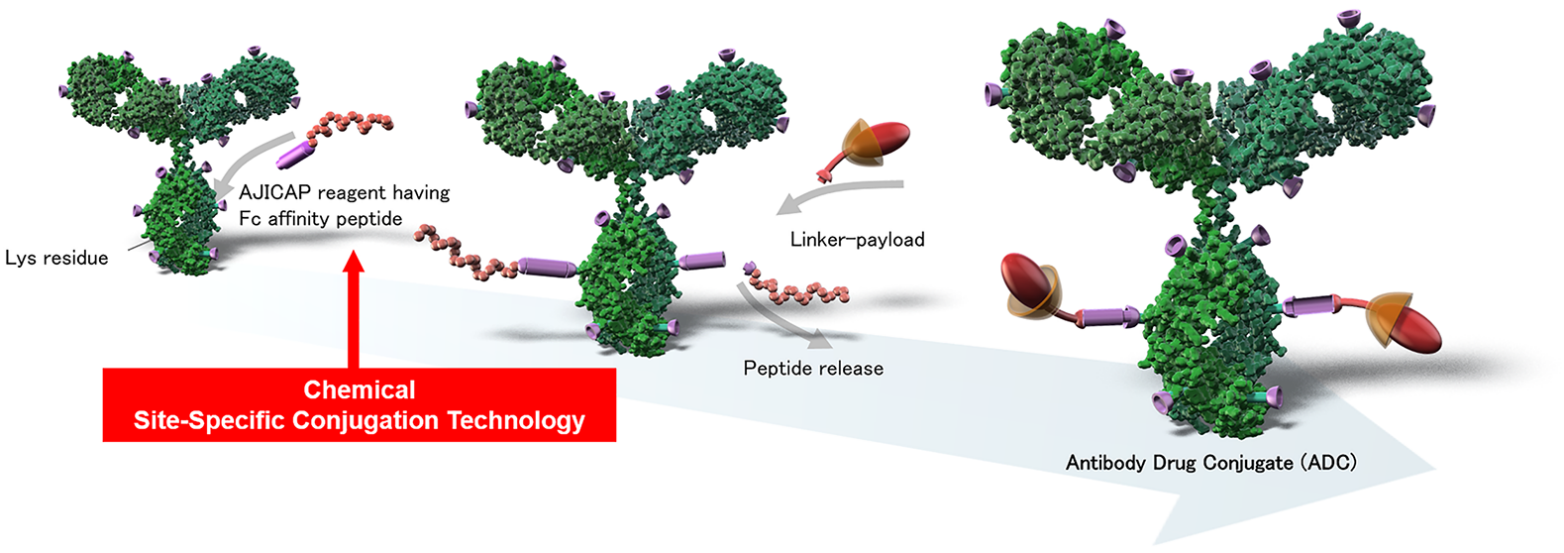

The site-directed chemical conjugation of antibodies remains an area of great interest and active efforts within the antibody–drug conjugate (ADC) community. We previously reported a unique site modification using a class of immunoglobulin-G (IgG) Fc-affinity reagents to establish a versatile, streamlined, and site-selective conjugation of native antibodies to enhance the therapeutic index of the resultant ADCs. This methodology, termed “AJICAP”, successfully modified Lys248 of native antibodies to produce site-specific ADC with a wider therapeutic index than the Food and Drug Administration-approved ADC, Kadcyla. However, the long reaction sequences, including the reduction–oxidation (redox) treatment, increased the aggregation level. In this manuscript, we aimed to present an updated Fc-affinity-mediated site-specific conjugation technology named “AJICAP second generation” without redox treatment utilizing a “one-pot” antibody modification reaction. The stability of Fc affinity reagents was improved owing to structural optimization, enabling the production of various ADCs without aggregation. In addition to Lys248 conjugation, Lys288 conjugated ADCs with homogeneous drug-to-antibody ratio of 2 were produced using different Fc affinity peptide reagent possessing a proper spacer linkage. These two conjugation technologies were used to produce over 20 ADCs from several combinations of antibodies and drug linkers. The *in vivo* profile of Lys248 and Lys288 conjugated ADCs was also compared. Furthermore, nontraditional ADC production, such as antibody–protein conjugates and antibody–oligonucleotide conjugates, were achieved. These results strongly indicate that this Fc affinity conjugation approach is a promising strategy for manufacturing site-specific antibody conjugates without antibody engineering.

## Introduction

In the past decade, chemical conjugation to produce site-specific antibody–drug conjugates (ADCs) has received considerable attention in the oncology field.^1,2^ The first example of site-specific ADC approved by the Food and Drug Administration was relished by Daiichi-Sankyo’s unique chemical conjugation approach using a high drug-to-antibody ratio (DAR) technology consisting of reduction of all interchain disulfide bonds, followed by thiol-maleimide coupling with their original drug linker (deruxutecan).^3,4^ Since this technique cleaves all interchain disulfide bonds, it is characterized by its ability to obtain near-homogeneous ADCs. This elegant solution, even though the resultant ADCs contain a few lower DAR species,^5^ enables the production of nearly homogeneous ADC with DAR = 8 without aggregation and loss of antibody properties, such as antigen binding.^3,4^ However, this technology is limited by compatible drug linkers. For example, MC-VC-MMAE, a commonly used ADC drug linker in the market,^6^ cannot be applied to this high DAR technology because hydrophobicity causes aggregation, lowering the physical and biological profile of the ADCs.^7^ Moreover, a high DAR ADC may not be an ideal molecular format for every ADC. Another ADC manufactured by Daiichi-Sankyo’s conjugation technology using deruxutecan in the clinical stage has a DAR lower than 8, supporting that the appropriate DAR must be adjusted based on the pharmacology of the target and/or the toxicity profiles of ADCs.^8^ The Seattle Genetics (now Seagen) group published an *in vivo* pharmacokinetics (PK) and efficacy comparison study between chromatographically purified DAR = 2, DAR = 4, and DAR = 8 ADCs, suggesting that the lower DAR, especially DAR = 2, had an ideal biological profile.^9^ Several developments have been reported that improve the PK profile by reducing the hydrophobicity of high DAR ADCs;^2,7^ however, the DAR = 2 ADC remains a promising ADC format because of its simple structure that does not require hydrophilic linkers.

Owing to the limitations in high DAR technology and the potential requirement of DAR = 2 production, the Ajinomoto group commenced the development of site-specific conjugation technology utilizing Fc-affinity peptide reagents in 2019.^10^ The proof-of-concept study revealed that this affinity-guided approach enabled the modification of a specific lysine in the Fc region of various antibodies, including immunoglobulin-G (IgG)1, IgG2, and IgG4, to produce homogeneous DAR = 2 ADCs. This site occupancy was proved via several analyses, including trypsin-digested peptide mapping, which revealed that the conjugation site of the resultant ADCs is solely Lys248.^11^ The Fc region is a constant domain of every antibody;^12^ therefore, this Fc-affinity conjugation technology, termed AJICAP, enables the application (theoretically) of all antibodies without complicated reaction optimization. Furthermore, biological evaluation of site-specific AJICAP-ADC consisting of cytotoxic monomethyl auristatin E (MMAE) indicated that AJICAP first-generation technology enhanced therapeutic index compared with stochastic cysteine-based ADCs.^13^ This improvement in the *in vivo* profile was also observed with different payload (maytansinoid) cases. Site-specific AJICAP-ADC, consisting of maytansinoid, demonstrated higher *in vivo* efficacy and tolerability than Kadcyla,^14^ a clinical ADC approved by the Food and Drug Administration. These results indicate that AJICAP technology has great potential for producing next-generation ADCs.

**Figure 1:**
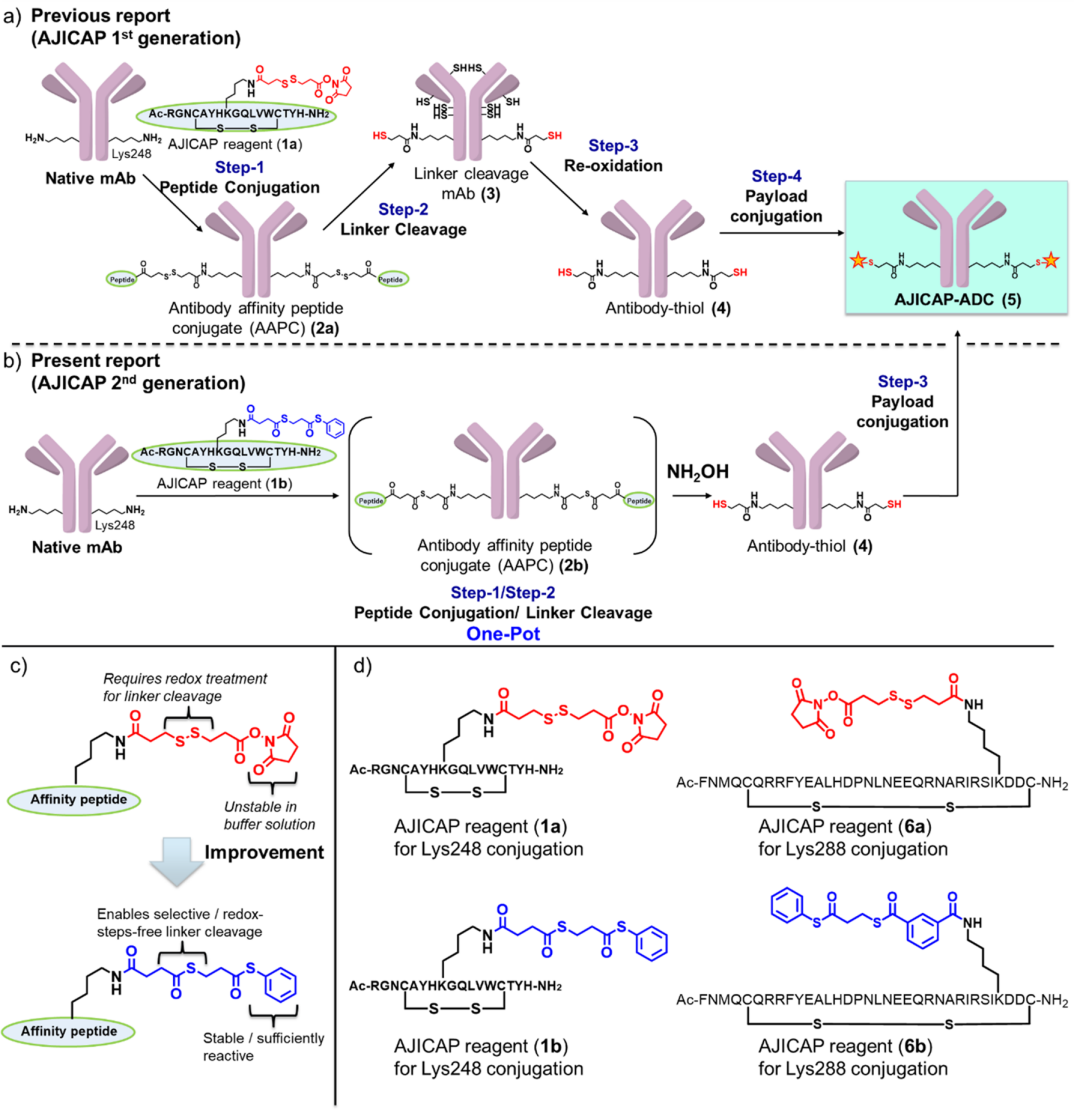
Comparison of AJICAP first and second technology: (a) AJICAP first-generation technology, including redox treatment (steps 2 and 3). (b) AJICAP second generation, including one-pot peptide or linker cleavage reaction. (c) Comparison of AJICAP first and second generation reagents. (d) The structures of AJICAP reagents for Lys248 and Lys288 conjugation.

Although AJICAP first-generation technology is a promising approach for manufacturing site-specific ADCs, several challenges remain (Figure 1a). This previous approach requires tris(2-carboxyethyl)-phosphine hydrochloride (TCEP) reduction to cleave the linkage between antibodies and AJICAP peptide reagent to install thiol groups on specific lysine. However, this reduction also cleaves the disulfide bonds of interchain cysteines in antibodies. Therefore, the disulfides must be reconstructed following the reoxidation step using dehydroascorbic acid. This redox treatment is also used in THIOMAB conjugation technology;^15^ however, it can lead to disulfide bond scrambling.^2^ A more critical concern in this approach is the risk of aggregation. Small amounts of aggregates (5–10%) were found in ADCs produced using the AJICAP first-generation technology.^13^ Additionally, a more streamlined manufacturing sequence consisting of fewer reaction steps is preferred from the chemical manufacturing control perspective.

Our research group performed optimization studies to streamline the conjugation reaction sequence to overcome these issues (Figure 1b). This improved technology, termed “AJICAP second generation,” enables the production of site-specific ADC while preventing aggregation due to selective cleavage reaction utilizing thioester chemistry. The stability of the AJICAP peptide reagent in buffer solution was improved, resulting in higher selectivity to target lysine and improved DAR value of ADCs. This thioester-based strategy provides two different AJICAP peptide reagents to access two different conjugation sites (Lys248 and Lys288). *In vivo* biological studies of Lys248- and Lys288-based ADCs revealed comparable efficacy and tolerability. Furthermore, the application of conjugation chemistry to nontraditional ADC production was investigated.

## Results and Discussion

### AJICAP Reagent Design and Antibody Modification

There are two major challenges to improving AJICAP peptide reagents (Figure 1c): (1) replacing the unstable *N*-succinimide (NHS) group with an amine group of lysine in the antibody and (2) replacing disulfide bonds in the AJICAP reagent requiring redox treatment in the ADC synthesis scheme.

Considering the shelf life and reactivity balance, the thiophenyl ester group was selected as a reactive group for antibody modification.^16^ The thiophenol group has higher stability and slightly lower reactivity than the NHS group. However, we expected that the proximity effect of the Fc affinity peptide would enhance reactivity for antibody modification. Additionally, the longer shelf life of thiophenyl ester in the reaction buffer solution compared to NHS ester improved the modification yield and site-specificity. An alkylthioester linkage was selected to replace the disulfide bond in the AJICAP first-generation reagent. Alkylthioesters are well-known protecting groups of thiols. *N*-Succinimidyl *S*-acetylthioacetate is a widely used alkylthioester reagent for protein functionalization.^17^ Modification of lysine of protein using *N*-succinimidyl *S*-acetylthioacetate followed by specific deprotection using hydroxylamine can install sulfhydryl groups on the lysine residue. An optimized AJICAP reagent (**1b**) was produced from these thioesters (alkyl and phenyl) (Figure 1d; Supporting Information (SI), Figure S1–S3). Furthermore, another AJICAP peptide reagent (**6b**) possessing different affinity peptide sequence was designed to modify the Lys288 of antibodies (SI, Figure S4 and S5). In previous studies, the modification yield of Lys288 was lower than that of Lys248; therefore, we installed a rigid architecture^18^ to allow the reactive thiophenyl ester group of the AJICAP reagent to be close to Lys288 in buffer conditions.

Trastuzumab and rituximab were used to demonstrate thiophenyl ester chemistry (Table 1).

**Table 1:**
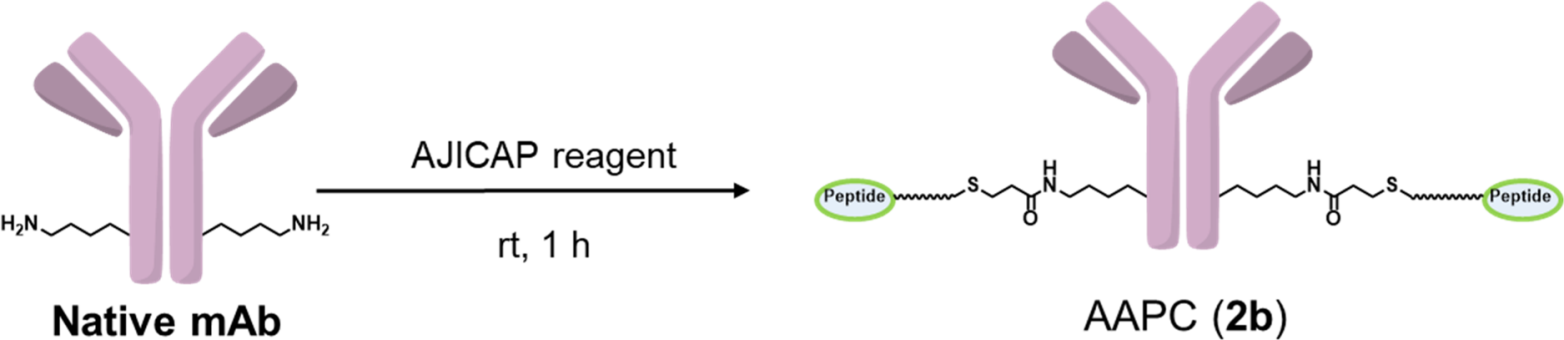
Site-Specific Peptide Conjugation

					Peptide antibody ratio (PAR)^a^				
Entry	Antibody	Conjugation site	Conjugation method	AJICAP reagent	0	1	2	3	Average^b^ PAR
1	Trastuzumab	K248	First Gen.	**1a**	-	5%	95%	-	1.9
2	Trastuzumab	K248	Second Gen.	**1b**	-	1%	98%	1%	2.0
3	Trastuzumab	K288	First Gen.	**6a**	23%	50%	26%	1%	1.1
4	Trastuzumab	K288	Second Gen.	**6b**	-	2%	95%	3%	2.0
5	Rituximab	K248	Second Gen.	**1b**	-	10%	90%	-	2.0
6	Rituximab	K288	Second Gen.	**6b**	-	-	97%	3%	2.0

a Calculated by Q-TOF-MS.

b Analyzed by HIC-HPLC.

The conversion yield from antibodies to conjugates was calculated by measuring the peptide-to-antibody ratio (PAR) using quadrupole time-of-flight mass spectrometry (Q-TOF MS) (SI, Figures S6–S10)^13^ and hydrophobic interaction chromatography (HIC)-HPLC (SI, Figures S11–S21).^19^

In the previous Lys248 conjugation, AJICAP reagent (**1a**) provided higher PAR (entry 1);^10^ however, AJICAP reagent (**1b**), consisting of a thiophenyl group, achieved further reactivity optimization to meet the targeted PAR and improved PAR = 2 selectivity (entry 2). In the case of Lys288 conjugation, further optimization studies were needed. In previous studies, the first generation AJICAP reagent (**6a**) provided insufficient PAR (entry 3^10^). To understand this phenomenon, we performed a structural analysis of the complex of the base peptide (termed Z34C) of reagents **6a** and **6b**, with the Fc protein;^20,21^ the predicted distance from the Z34C peptide to Lys288 of the antibody was farther than the predicted distance from FcIII-like peptide^22^ to Lys248 (SI, Figure S22). These results indicate that the AJICAP reagent for Lys288 modification requires a longer length linker than the AJICAP reagent for Lys248 modification (SI, Figures S5, S8, and S14).^23^ Therefore, we tuned the linker lengths of the peptide reagents. Several groups have reported unique affinity-guided protein modification technologies; in some cases, rigid architectures, such as piperazine^18^ and proline^24^ in the linker portion, are powerful tools for reaching the reactive group to target amino acids with improved conversion. These reports prompted us to screen linkers to improve the modification efficiency of Lys288, and the aromatic ring was found to be a suitable linkage. The buffer pH in peptide conjugation step depended on the binding affinity of the peptide moiety of the AJICAP reagents; in the case of Lys 248 conjugation, FcIII-like peptide showed the highest binding affinity in acidic buffer (around pH 5.5). On the other hand, Z34C, which was used for Lys 288 conjugation, showed favorable affinity strength in slightly alkaline conditions (around pH 8.0, data not shown).

In both reagents (**1b** and **6b**), the following linker cleavage using hydroxylamine was completed (SI, Figures S15, S16, S20, and S21), indicating that the thiophenyl group reacted with lysine in the antibody, while no side reaction via alkylthioester occurred during peptide conjugation.

### The Capability of AJICAP Technology

The compatibility of this conjugation chemistry using several antibodies was evaluated via HIC-HPLC analysis (SI, Figures S23–S44). In the traditional cysteine-conjugation case, the antibody characteristics affected the reactivity and DAR of the resultant ADCs;^25^ therefore, a conjugation examination using several ADCs with different isoelectric points (PIs)^26^ was conducted to understand the reaction tolerability of AJICAP second-generation technology. In addition to trastuzumab (PI = 8.8, entries 1 and 2) and rituximab (PI = 9.1, entries 3 and 4), infliximab (PI = 7.6, entries 5 and 6), cetuximab (PI = 8.7, entries 7 and 8), denosumab (PI = 8.9, IgG_2_, entries 9 and 10), and pembrolizumab (PI = 8.7, IgG_4_, entries 11 and 12) were evaluated. In all cases, both AJICAP reagents (**1b** and **6b**) reacted sufficiently with these mAbs to produce conjugates with PAR = 2.0, indicating that this conjugation does not depend on the PI difference or IgG subtypes. These AJICAP reagents also modified the Fc protein (entries 13 and 14, confirmed using Q-TOF analysis (SI, Figures S45 and S46)), demonstrating the potential use of this Fc-modification technology for Fc-fusion protein production. Fc-Fusion is a commonly used technique for prolonging the *in vivo* half-life of small-and medium-sized molecules.^27^ Furthermore, this powerful conjugation technology enabled the modification of polyclonal antibodies (whole IgGs purified from human serum) (entries 15 and 16).^28^ Sequential linker cleavage using excess equivalents of hydroxylamine enables the release of an affinity peptide portion, providing site-specific thiol-incorporating antibodies (**4**). In all reaction cases, aggregation percentages analyzed using size exclusion chromatography (SEC) were retained postconjugation or linker cleavage (SI, Figures S47–S62).

In addition, we succeeded in omitting the purification step after AJICAP peptide conjugation. As described in Table 2, the one-pot reaction of peptide conjugation followed by linker cleavage modified all antibodies, with the same efficiency and aggregation profile as the stepwise conversion. This streamlined process is applicable to ADC manufacturing.

**Table 2:**
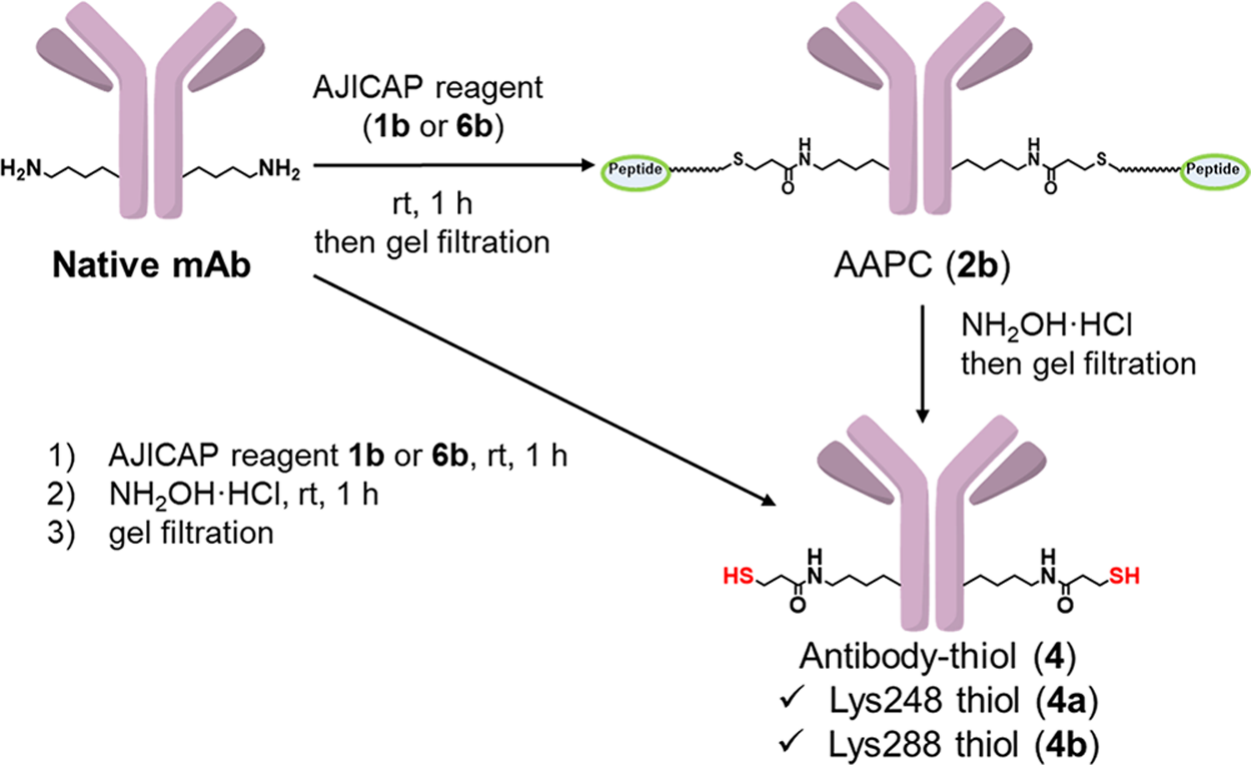
Stepwise and One-Pot Conversion from Native Mabs to Antibody-Thiol (**4**)

Entry	Antibody	Subtype	Conjugation site	AJICAP reagent	Average PAR^a^ of **2b**	Aggregation of **4**
1	Trastuzumab	IgG1	K248	**1b**	2.0	<1%
2	Trastuzumab	IgG1	K288	**6b**	2.0	1.0%
3	Rituximab	IgG1	K248	**1b**	2.0	<1%
4	Rituximab	IgG1	K288	**6b**	2.0	1.0%
5	Infliximab	IgG1	K248	**1b**	2.0	<1%
6	Infliximab	IgG1	K288	**6b**	2.0	<1%
7	Cetuximab	IgG1	K248	**1b**	2.0	<1%
8	Cetuximab	IgG1	K288	**6b**	2.0	1.7%
9	Denosumab	IgG2	K248	**1b**	2.0	<1%
10	Denosumab	IgG2	K288	**6b**	2.0	<1%
11	Pembrolizumab	IgG4	K248	**1b**	2.0	<1%
12	Pembrolizumab	IgG4	K288	**6b**	2.0	<1%
13	Fc-Protein	-	K248	**1b**	2.0^b^	1.0%
14	Fc-Protein	-	K288	**6b**	2.0^b^	1.0%
15	Polyclonal antibody	-	K248	**1b**	2.0	2.6%
16	Polyclonal antibody	-	K288	**6b**	2.0	3.3%

a Analyzed by HIC-HPLC.

b Analyzed by Q-TOF.

c Analyzed by SEC-HPLC.

### ADC Syntheses

Over 20 ADCs were synthesized to demonstrate the compatibility of antibody-thiols (**4**) produced by AJICAP second-generation technology (Table 3).

**Table 3:**
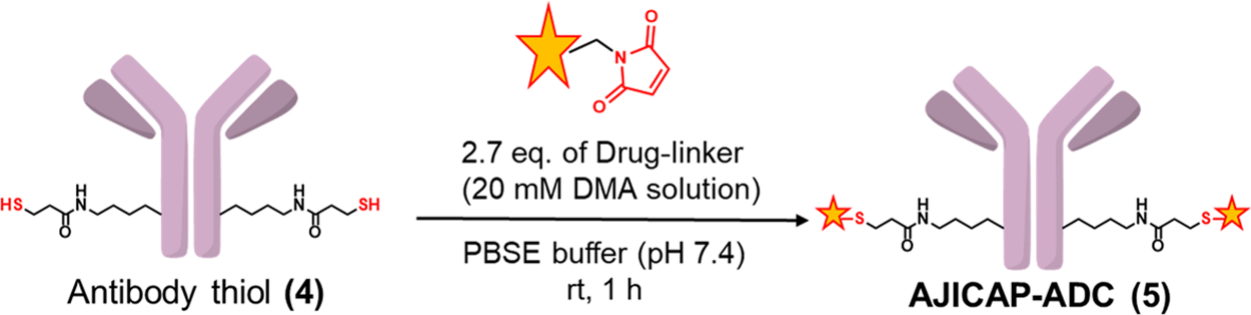
ADC Syntheses

Entry	mAb	Drug-linker	Conj. site	DAR by Q-TOF	DAR by HIC	Aggregation
1	Trastuzumab	MCC-Maytansinoid	K248	1.9	1.9	1.8%
2	Trastuzumab	MC-VC-PAB-MMAE	K248	1.9	1.9	1.9%
3	Trastuzumab	MC-MMAF	K248	1.8	1.9	1.8%
4	Trastuzumab	Tesirine	K248	1.9	1.9	2.0%
5	Trastuzumab	MC-GGFG-Dxd	K248	2.0	1.9	1.9%
6	Trastuzumab	MCC-Maytansinoid	K288	1.8	1.9	2.5%
7	Trastuzumab	MC-VC-PAB-MMAE	K288	1.7	1.8	2.0%
8	Trastuzumab	MC-MMAF	K288	1.9	1.9	2.5%
9	Trastuzumab	Tesirine	K288	2.0	1.9	2.6%
10	Trastuzumab	MC-GGFG-Dxd	K288	2.0	1.8	2.5%
11	Rituximab	MCC-Maytansinoid	K248	1.9	1.9	2.0%
12	Rituximab	MC-VC-PAB-MMAE	K248	1.8	1.9	1.8%
13	Rituximab	MC-MMAF	K248	1.8	1.8	2.2%
14	Rituximab	Tesirine	K248	1.8	1.8	3.8%
15	Rituximab	MC-GGFG-Dxd	K248	1.9	1.8	2.0%
16	Rituximab	MCC-Maytansinoid	K288	1.9	1.8	3.3%
17	Rituximab	MC-VC-PAB-MMAE	K288	1.7	1.8	3.0%
18	Rituximab	MC-MMAF	K288	1.9	1.9	3.2%
19	Rituximab	Tesirine	K288	1.9	1.9	3.2%
20	Rituximab	MC-GGFG-Dxd	K288	2.0	1.8	2.0%

There are various drug linkers in the current ADC field, each with unique hydrophobicity and reactivity.^29^ Therefore, we evaluated five drug linkers (MCC-maytansinoid, MC-VC-PAB-MMAE, MC-MMAF, Tesirine, and MC-GGFG-Dxd; Figure S63 in SI presents their structures). All ADCs converted from trastuzumab-Lys248-thiol, trastuzumab-Lys288-thiol, rituximab-Lys248-thiol, and rituximab-Lys288-thiol had higher DAR values than previously reported (AJICAP first generation production).^10^ DAR was determined using two different analytical methods (Q-TOF MS and HIC-HPLC) (SI, Figures S64–S83 and S84–S103). The average DAR values derived from two different analyses were indistinguishable. The measured differences are attributed to method sensitivity, accuracy, or linearity.^30,31^ The aggregation percentage of all ADCs was less than 3.0% (SI, Figures S104–S123), which were not significantly different from the naked antibodies (trastuzumab and rituximab). These results indicated that this improved technology could be a more practical approach for various ADCs production than previous AJICAP technology.^32^

### Conjugation Site Determination

**Figure 2:**
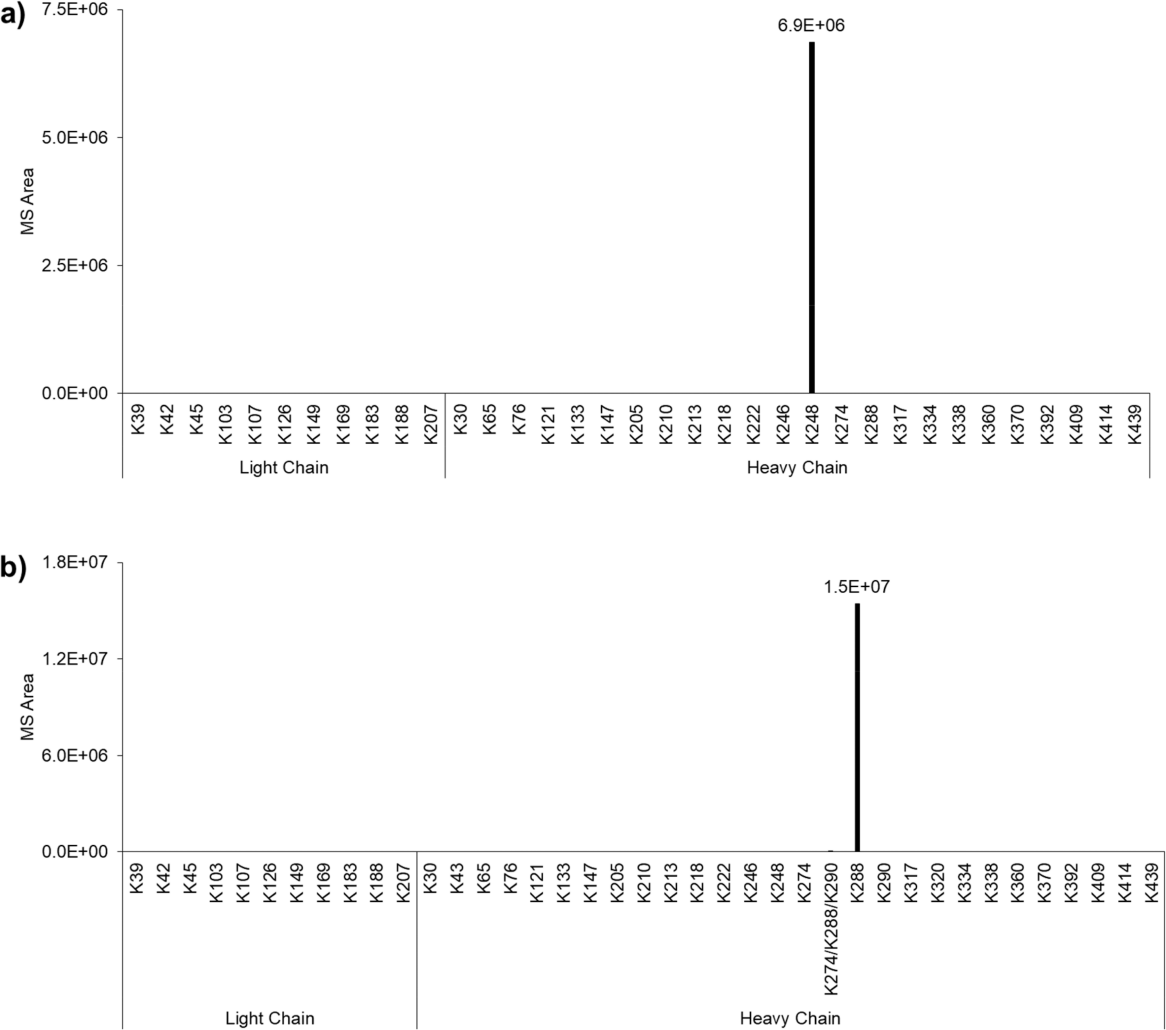
Summary of lysine residue search by BioPharma Finder: (a) analysis of trastuzumab-Lys248-thiol (**4a**); (b) analysis of trastuzumab-Lys288-thiol (**4b**). Each result shows the high specificity of the AJICAP reaction.

**Figure 3:**
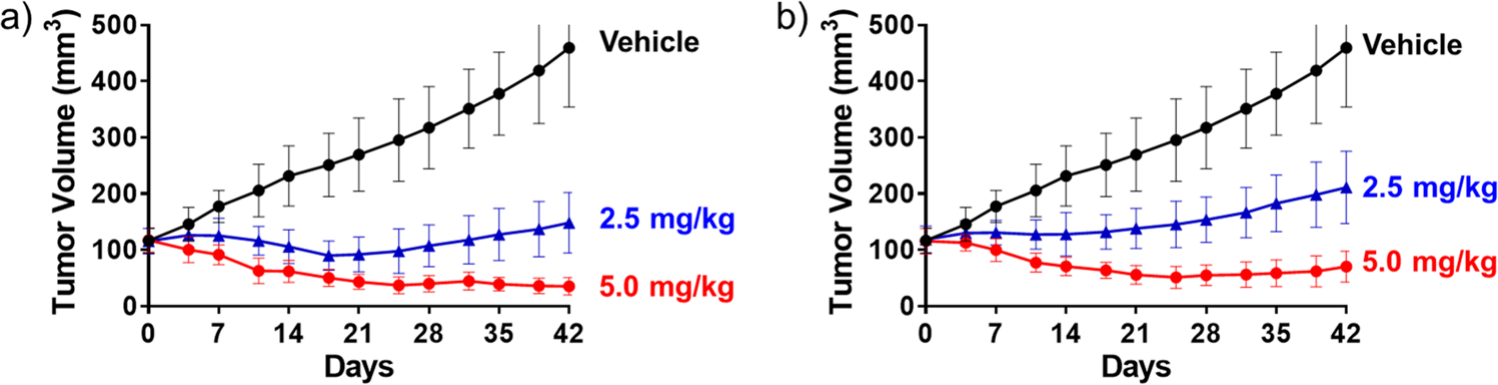
*In vivo* efficacy comparison between Lys248-ADC and Lys288-ADC: (a) Antitumor activity of anti-HER2 trastuzumab-Lys248-MMAE (**5a**) in the NCI-N87 xenograft tumor model. (b) Antitumor activity of anti-HER2 trastuzumab-Lys288-MMAE (**5b**) in the NCI-N87 xenograft tumor model.

The conjugation site of these trastuzumab-thiols (**4a** derived from **1b** and **4b** derived from **6b**) was determined using peptide mapping analysis (Figure 2). In our previous peptide mapping analysis, the sequence coverage was approximately 80% because of a single enzyme (trypsin) digestion.^10^ To increase this coverage, double enzymatic digestion was performed using trypsin and Lys-C.^33^ In addition, we optimized HPLC condition including a long gradient and newly constructed HPLC system to increase peak resolution (see Experimental Section). Consequently, the sequence coverage was improved to more than 95%. The thiol adduct was compared to the results, and target-specific modification of AJICAP-antibody-thiols (Lys248 and Lys288) was indicated (Figure 3). Among detected lysines, the target site (Lys248 and Lys288, respectively) was specifically subject to 3-(2-amino-2-oxo-ethyl) sulfanylpropionate modification. In fact, for the AJICAP-Lys248-thiol sample, we detected a peptide fragment including two candidate residues Lys246 and Lys248 (THTCPPCPAPELLGGPSVFLFPPK^246^PK^248^DTLMISR) for modification. In this analysis, Lys-C and trypsin for digestion were used; therefore lysines and arginines excepting followed by proline are recognized and cleaved. Lys246 is located just behind the proline and is inhibited from enzymatic cleavage, while Lys248 should be cleaved by the enzyme. From these considerations, we concluded Lys248 is specifically modified. Similarly for AJICAP-Lys288-thiol, we identified FNWYVDGVEVHNAK288TK290PR, having two lysines Lys288 and Lys290. We concluded that AJICAP-Lys288-thiol achieved specific modification at Lys288 for the same reason. Detailed extracted ion chromatogram (XIC) and MS/MS results supporting the site-specificity of two antibody-thiols (Lys248 and Lys288) are shown in SI, Figures S124 and S125.

### *In Vivo* Biological Evaluation of AJICAP-ADCs

In previous studies, our research group revealed enhanced therapeutic index using AJICAP conjugation technology than stochastically conjugated ADCs.^13,14^ Following these evaluations, we examined the biological evaluation of AJICAP-ADCs produced using second-generation methods (Figure 3). In addition, Lys288-conjugated ADC was evaluated to compare the *in vivo* efficacy of these two conjugation sites. Trastuzumab-Lys248-MMAE (**5a**) and trastuzumab-Lys288-MMAE (**5b**) were selected in these biological studies.

**Figure 4:**
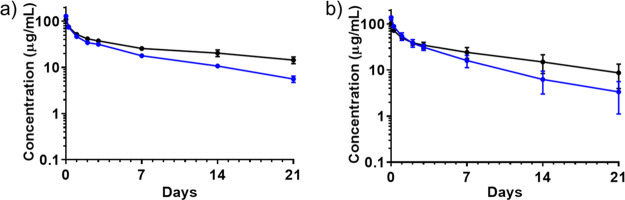
PK profile comparison between Lys248-ADC and Lys288-ADC: (a) Plasma concentration of total mAb (black line) and total ADC (blue line) of anti-HER2 trastuzumab-Lys248-MMAE (**5a**) in rats measured using ELISA. (b) Plasma concentration of mAb (black line) and total ADC (blue line) of trastuzumab-Lys288-MMAE (**5b**) in rats measured using ELISA.

First, two different doses of trastuzumab-derived AJICAP-ADC (**5a**) (conjugation site: Lys248, payload: MMAE) were evaluated in a HER2-positive NCI-N87 gastric cancer xenograft model (Figure 4a). The Lys248 conjugated ADC (**5a**) at a dose of 5 mg/kg displayed significant tumor regression, comparable to previous ADC produced using the AJICAP first-generation technology.^13^ The other AJICAP-ADC (**5b**) (conjugation site: Lys288, payload: MMAE) also displayed significant efficacy at a 5 mg/kg dosage (Figure 4b). These results indicate that AJICAP conjugation technology can produce ADCs with comparable efficacy that are independent of conjugation site and generation. In previous studies,^34^ the minimum effective dose of stochastic DAR = 4 trastuzumab-MMAE ADC produced via native cysteine conjugation was 2.5 mg/kg. Despite the DAR number difference (stochastic ADC = 4, AJICAP-ADC = 2), the *in vivo* efficacy of AJICAP-ADCs was comparable to that of stochastic ADCs.

AJICAP conjugation technology modified the antibody’s Fc region; therefore, the resultant ADC was at risk of losing binding affinity to FcRn because of steric interference by the payload linker conjugated close to the FcRn binding site.^22^ However, in 2021, our research group discovered that AJICAP-ADC (conjugation site: Lys248, payload: MMAE) retained FcRn binding affinity postconjugation.^35^ These findings are reasonable considering the plasma stability of AJICAP-ADCs measured in a rat PK study.^13^ In this study, the FcRn binding affinity and PK profile of two different AJICAP-ADCs (**5a** and **5b**) (conjugation site: Lys248 or Lys288, payload: MMAE) produced using second-generation technology were confirmed. Biolayer interferometry analysis^36^ of these ADCs, as in a previous study^35^ revealed that both ADCs had comparable binding strength for immobilized FcRn with naked trastuzumab (Table 4). These affinity results of two different ADCs provided via biolayer interferometry can be rationalized from their molecular structures (SI, figure S126). Lys248 and Lys288 are acceptable sites for FcRn binding that avoids steric hindrance due to conjugated payload linkers. Rat PK studies supported this FcRn binding result (Figures 4). Total antibody levels from two AJICAP-conjugated ADCs (**5a** and **5b**) (conjugation site: Lys248 or Lys288, payload: MMAE) indicated a half-life similar to that of an unconjugated antibody. Total ADC analysis in rat PK studies indicated that these AJICAP-ADCs demonstrated sufficient stability in blood circulation. In contrast, stochastic ADC produced via cysteine conjugation technology showed insufficient stability in rats.^13^ Furthermore, we conducted an initial toxicology study of AJICAP-ADC (**5b**) (conjugation site: Lys288, payload: MMAE), which showed that this ADC did not negatively impact body weight changes in rats at the 80 mg/kg dosage (single dose, SI, Figure S127). The other ADC (**5a**) (conjugation site: Lys248, payload: MMAE) has already been evaluated in a rat toxicology study, which indicated that the maximum tolerated dose was >80 mg/kg.^13^ In previous studies, the estimated maximum tolerated dose value of trastuzumab-stochastic MMAE was approximately 10 mg/kg (single dose, previously reported^13^). These results indicate that the two AJICAP-ADCs have significantly wider tolerability than traditional stochastic ADCs. Further toxicological studies to determine the maximum tolerated dose of Lys288 conjugated ADC are underway.

**Table 4:** Binding Kinetics against Human FcRn

Entry	Analyte	Conjugation site	*K*_d_ (M)	*K*_on_ (1/Ms)	*K*_dis_ (1/s)
1	Trastuzumab	-	5.65 × 10^–9^	6.67 × 10^5^	3.77 × 10^–3^
2	Trastuzumab-K248-MMAE (**5a**)	K248	5.60 × 10^–9^	1.52 × 10^5^	8.48 × 10^–4^
3	Trastuzumab-K288-MMAE (**5b**)	K288	7.22 × 10^–9^	5.30 × 10^5^	3.83 × 10^–3^

Based on these *in vivo* efficacy, PK, and initial safety studies, we concluded that AJICAP second-generation technology enables the production of site-specific ADCs with higher therapeutic indexes than traditional stochastic ADCs.

In these *in vivo* data sets, no significant differences were observed in Lys248-ADCs and Lys288-ADCs. In the antibody development, some point mutations in the Fc region of an antibody to enhance FcRn binding were reported.^37^ Some of their mutated antibodies might lose their affinity for the AJICAP-Lys248 peptide reagent. The discovery of a novel AJICAP-ADC conjugated to Lys288 described herein may provide another alternative to using Fc region-mutated antibodies for AJICAP technology.

### Application to Novel Format Antibody Conjugates

**Figure 5:**
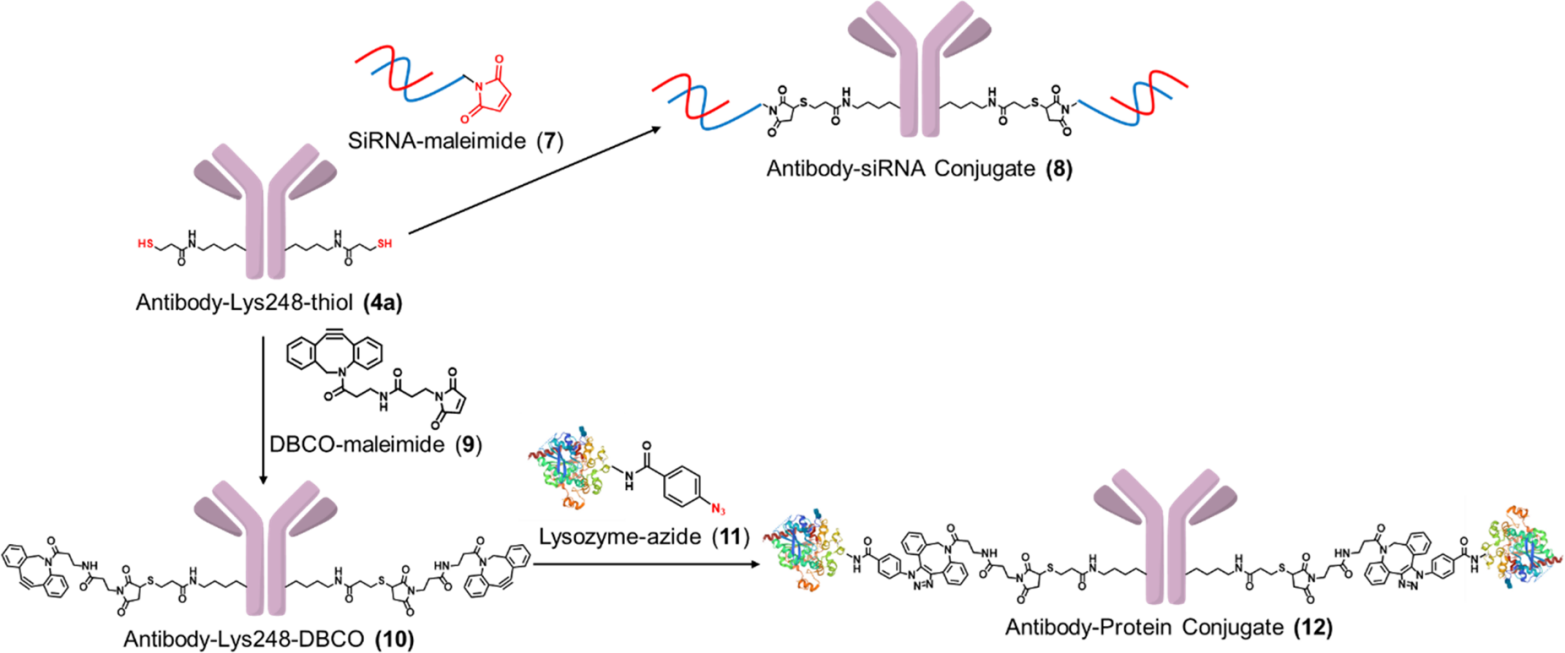
Synthesis of novel format antibody conjugates. The production of an antibody-SiRNA conjugate (**8**) (upper). The production of an antibody–protein conjugate (**12**) (lower).

Recently, innovations have enlightened the oncology or bioconjugation community and broadened the research and development area to include ADC alternative antibody–oligonucleotide conjugates^38^ and antibody–protein conjugates.^39^ Feasibility studies were performed using model conjugates to meet the potential requirements for producing these novel format antibody conjugates (Figure 5).

As a model SiRNA compound, we used a SiRNA compound whose sequence targets peptidylprolyl isomerase B (PPIB, cyclophilin B) reported by the Genentech group.^40^ Following their report, model SiRNA possessing C6 amine linker was converted to a maleimide conjugate (**7**) by amidation. This maleimide labeled SiRNA was conjugated with antibody-Lys248-thiol (**4a**) to afford the antibody–SiRNA conjugate (**8**) by thiol-maleimide coupling (detailed information is in the Experimental Section in SI). The resulting conjugate (**8**) was purified using preparative SEC-HPLC to remove excess unreacted SiRNA from the antibody-conjugate composition. SDS-PAGE analysis of this conjugate revealed a considerable conversion yield (SI, Figure S128). Lysozyme was selected as the model compound for protein conjugate production. Random lysine conjugation using an azide-NHS reagent produced the azide-incorporated lysozyme (**11**). Antibody-DBCO (**10**) transformed from antibody-Lys248-thiol (**4a**) and reacted with this azide-lysozyme (**11**) to form an antibody–protein conjugate (**12**). A Q-TOF MS analysis of (**12)** revealed that this conjugation proceeded smoothly (SI, Figures S129–131). These results indicate that the AJICAP conjugation strategy can be applied to produce ADCs with small cytotoxic molecules and ADC alternatives whose payloads are medium-sized molecules. Further investigations, including DAR determination, process development, and *in vitro* and *in vivo* evaluation, are currently ongoing.

## Conclusion

Our research group has completed proof-of-concept studies of AJICAP second-generation and improved chemical conjugation technology using Fc-affinity reagents. This novel methodology is compatible with various antibody formats, including antibody fragments and polyclonal antibodies. The linker structural tuning enabled site-specific modification of native IgGs at the novel conjugation site (Lys288) with a high conversion yield. This conjugation technology can easily introduce two payload linkers per native antibody, producing over 20 site-specific ADCs without aggregation during the reaction. *In vivo* biological studies, including mouse efficacy, rat PK, and toxicology studies, of ADCs produced by this chemical site-specific conjugation technology indicate a widened therapeutic index.

Furthermore, preliminary feasibility studies for application to nontraditional ADCs, including antibody–oligonucleotide conjugates and antibody–protein conjugates, were completed. This technology enables rapid, versatile, streamlined, site-specific conjugation to various native antibodies with several linker payloads, including medium-sized molecules, to produce next-generation antibody conjugates.

## Experimental Procedures

### Materials

Human IgG1 trastuzumab (Herceptin) and rituximab (Rituxan) were purchased from the Roche Pharmaceutical Company (Switzerland). Human IgG1 infliximab (Remicade) was purchased from Sigma-Aldrich (USA). Human IgG1 cetuximab (Erbitux), Human IgG2 denosumab (Prolia), and human IgG4 pembrolizumab (Keytruda) were purchased from Midwinter. Polyclonal antibody (human IgG Whole molecule, cat#: 143–09501) was purchased from Fujifilm Wako. Human IgG1 Fc Recombinant Protein (cat#: A42561) was purchased from Thermo Fisher Scientific. MCC-maytansinoid (cat#: TCRS-1262) and MC-MMAF (CAS#:1228105–51–8) were purchased from Abzena (USA). MC-VC-MMAE (CAS#: 646502–53–6), MC-PEG8-VA-PBD (CAS#: 1595275–62–9), and MC-GGFG-Dxd (CAS#: 1599440–13–7) were purchased from NJ Biopharmaceuticals, LLC (USA). All other chemical reagents were purchased from Sigma-Aldrich (USA).

### Instruments and Analytical Methods

The ADC concentration and recovery were measured using the Slope Spectroscopy method with a Solo-VPE system.^13^

Q-TOF MS analysis was performed as previously reported.^13^

Hydrophobic interaction chromatography-HPLC analysis was performed as previously reported.^19^

The SEC-HPLC analysis of antibody-thiols (**4**) was performed using an AdvanceBio SEC column (200 Å, 4.6 × 300 mm, 1.9 μm) as previously reported.^28^

SEC-HPLC analysis of ADCs (**5**) was performed using Waters ACQUITY UPLC Protein BEH SEC column (200 Å, 4.6 × 300 mm, 1.7 μm) as previously reported.^28^

Biolayer interferometry assay to analyze FcRn binding was performed as previously reported.^36^

### Synthetic Protocols to Produce AJICAP Reagents

Supporting Information contains detailed synthetic protocols for producing AJICAP reagents.

### AJICAP Peptide Reagent Conjugation

#### Lys248 Conjugation Using AJICAP Reagent (**1b**)

We added 2.5 equiv of AJICAP reagent (**1b**) (20 mM in DMF) to each mAb (10 mg/mL, 20 mM acetate buffer, pH 5.5), and the mixture was incubated for 1 h at 20 °C. After 1 h, the reaction mixture was purified using NAP-25 desalting columns and eluted with 20 mM acetate buffer (pH 5.5).

#### Lys288 Conjugation Using AJICAP Reagent (**6b**) or (**6c**)

Six equivalents of the AJICAP reagent (**6b** or **6c** (structure in SI)) (20 mM in DMF) were added to each mAb (10 mg/mL, 20 mM borate buffer, pH 8.2), and the mixture was incubated for 1 h at 20 °C. After 1 h, the reaction mixture was purified using NAP-25 desalting columns and eluted with 20 mM acetate buffer (pH 5.5).

#### Lys248 Conjugation Using AJICAP Reagent (**1b**) Followed by Linker Cleavage (One-Pot)

We added 2.5 equiv of AJICAP reagent (**1b**) (20 mM in DMF) to each mAb (10 mg/mL, 20 mM acetate buffer, pH 5.5), and the mixture was incubated for 1 h at 20 °C. After 1 h, excess NH_2_OH HCl was added, and the mixture was stirred for an additional 1 h. This reaction mixture was purified using a NAP-25 desalting column and eluted with 20 mM acetate buffer at pH 5.5.

#### Lys288 Conjugation Using AJICAP Reagent (**6b**) Followed by Linker Cleavage (One-Pot)

Six equivalents of AJICAP reagent (**6b**) (20 mM in DMF) were added to each mAb (10 mg/mL, 20 mM borate buffer, pH 8.2), and the mixture was incubated for 1 h at 20 °C. After 1 h, excess NH_2_OH HCl and 1 M acetate buffer (pH 4.7) for adjusting the pH of the reaction mixture (less than pH 6.0) were added and stirred for an additional 1 h. Subsequently, the reaction mixture was purified using a Centripure P50 desalting column and eluted with 20 mM acetate buffer (pH 5.5).

### ADC Synthesis

Payload conjugation with antibody-thiol (**4**) was achieved using a previously established procedure.^13^ The SI shows the payload linkers used in this study.

### Peptide Mapping for Conjugation Site Determination

For peptide mapping, 20 μg of each sample (trastuzumab, trastuzumab-Lys248-thiol, and trastuzumab-Lys288-thiol) was diluted to 110 μL with 0.25 M Tris-HCl (pH 7.5)/0.75 M GnHCl buffer. Reductive alkylation was achieved by adding dithiothreitol (DTT) and iodoacetamide (IAM) successively. The sample was then buffer exchanged to 100 mM Tris-HCl (pH 7.5) by Zeba Spin Desalting columns. For enzymatic digestion, we added 3:1 mixture of trypsin and Lys-C, then incubated at 37 °C for 18 h. Digestion was quenched by adding formic acid and acetonitrile.

The resulting peptide mixture was analyzed on an Orbitrap Fusion Tribrid (Thermo Fisher Scientific) interfaced with a Vanquish Duo (Thermo Fisher Scientific). We used an ACQUITY UPLC CSH C18 Column (1.7 μm, 2.1 × 150 mm, Cat #186005298, Waters) with a column temperature of 50 °C. The chromatographic method consisted of a 2 min hold at 2% solvent B (0.1% formic acid in acetonitrile) and a 55 min linear gradient from 2 to 42% solvent B. After a linear rise to 90% solvent B in 5 min, a wash step was performed with a 5 min hold at 90% solvent B. Subsequently, solvent B was reduced to 2% in 1 min and maintained at 2% for 12 min. The flow rate was set to 250 μL/min, and solvent A contained 0.1% formic acid. Mass spectrometry analysis was conducted in data-dependent acquisition mode with full scans (350–2,000 *m*/*z*) acquired at a mass resolution of 120,000. Tandem mass spectra were produced using the higher energy collision-induced dissociation method.

The resulting MS/MS data were compared against the trastuzumab sequence (Figure S22) using BioPharma Finder 3.2 (Thermo Fisher Scientific). Carbamidomethylation of cysteine (+57.021 Da) was specified as a fixed modification, and oxidation of methionine (+15.995 Da) and 3-(2-amino-2-oxo-ethyl) sulfanylpropionate of lysine (+145.019 Da) were included as variable modifications.

### Biological Evaluation Procedure

Detailed biological evaluation procedure can be found in the SI.

## Supporting Information Available

Details of the in vivo studies, figures supporting the molecular modeling, HPLC chromatograms, and QTOF MS analyses (PDF)

## Abbreviations

ADC: antibody–drug conjugate
IgG: immunoglobulin-G
DAR: drug-to-antibody ratio
NHS: *N*-succinimide
PAR: peptide-to-antibody ratio
PK: pharmacokinetic
Q-TOF MS: quadrupole time-of-flight mass spectrometry
SEC: size exclusion chromatography
