# Supplementary material for: AJICAP Second Generation: Improved Chemical Site-Specific Conjugation Technology for Antibody–Drug Conjugate Production
Source: Bioconjug Chem. 2023 Mar 9;34(4):728–38. doi: 10.1021/acs.bioconjchem.3c00040 (PMC10119932; doi:10.1021/acs.bioconjchem.3c00040)
Supplement: Supplementary file 1 — bc3c00040_si_001.pdf [file bc3c00040_si_001.pdf]

Supporting information

AJICAP Second Generation: Improved Chemical Site-Specific Conjugation Technology for Antibody-Drug Conjugate Production

Tomohiro Fujii[†1], Yutaka Matsuda*[‡], Takuya Seki[†], Natsuki Shikida[†], Yusuke Iwai[†], Yuri Ooba[†], Kazutoshi Takahashi[†], Muneki Isokawa[†], Sayaka Kawaguchi[†], Noriko Hatada[†], Tomohiro Watanabe[†], Rika Takasugi[†], Akira Nakayama[†], Kazutaka Shimbo[†], Brian A. Mendelsohn[‡§], Tatsuya Okuzumi[†], Kei Yamada*[†1]

* Corresponding authors

KY: kei.yamada.wa9@asv.ajinomoto.com

YM: Yutaka.Matsuda@US.AjiBio-Pharma.com

[1] These authors contributed equally.

[†]Ajinomoto Co., Inc., 1-1, Suzuki-Cho, Kawasaki-Ku, Kawasaki-Shi, Kanagawa 210-8681, Japan.

[‡]Ajinomoto Bio-Pharma Services, 11040 Roselle Street, San Diego, CA 92121, United States.

[§]Present addresses: Exelixis Inc, 1851 Harbor Bay Pkwy, Alameda, CA 94502, United States.

Table of Content

1. Experimental procedure

1-1 AJICAP reagents synthesis S–3

1-2 Molecular modeling S–14

1-3 in vivo xenograft study S–14

1-4 Rat PK study S–16

1-5 Rat safety study S–16

1-6 Synthesis of novel format antibody conjugates S–17

2. Analytical results

2-1 AJICAP reagents synthesis: 1H, 13C NMR, HPLC, and LC-MS S–19

2-2 Peptide conjugation of trastuzumab and rituzimab: Q-TOF MS and HIC analysis S–24

2-3 Molecular modeling of interaction between human IgG1 Fc and the affinity peptides S–35

2-4 Capability of AJICAP technology: HIC, Q-TOF MS, and SEC analysis S–36

2-5 ADC syntheses: payload-linker structure, Q-TOF MS, HIC, and SEC analysis S–55

2-6 Peptide mapping S–96

2-7 Structural analysis of FcRn/Fc protein S–100

2-8 In vivo evaluation of AJICAP-ADCs: Mouse xenograft, Rat PK study and safety study S–101

2-9 Application to novel format antibody conjugates: SDS-Page and Q-TOF MS analysis S–103

1. Experimental procedure

1-1 AJICAP reagents synthesis

1-1-1 Overview of synthetic process for AJICAP reagent (1b)

AJICAP peptide (**1b**) was synthesized as below protocol (Scheme S1.).

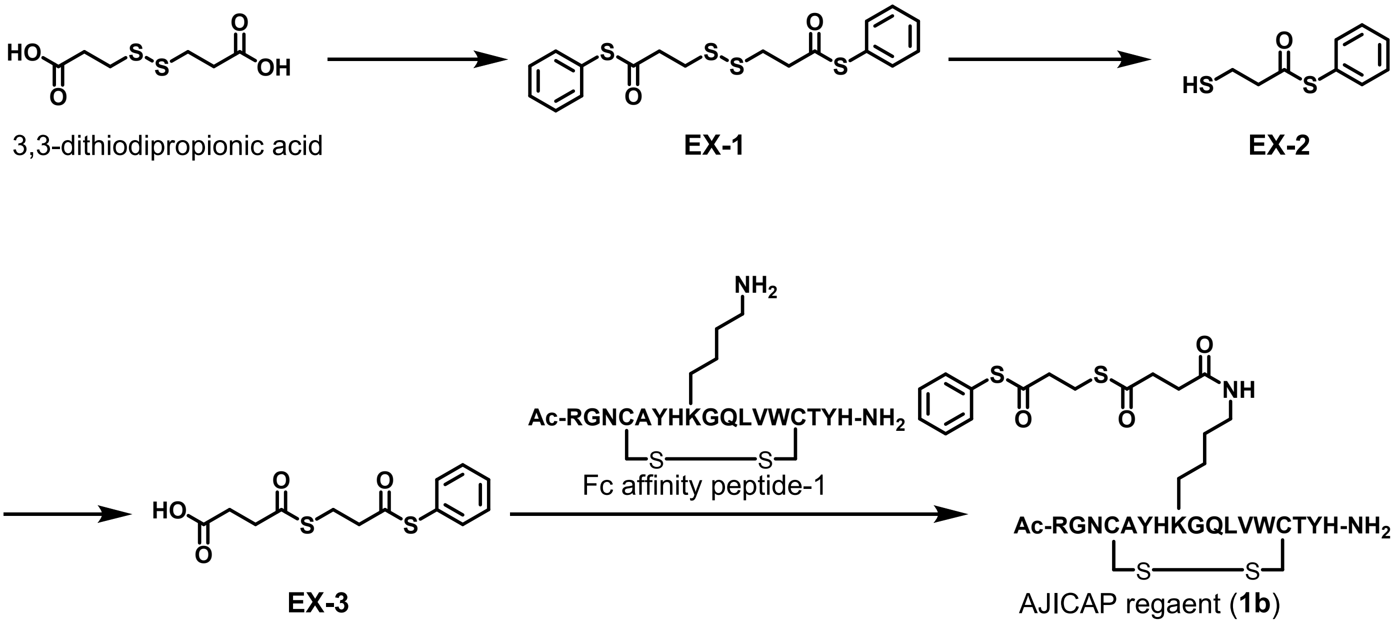

Scheme S1 Synthesis of AJICAP reagent (**1b**)

1-1-2 Synthesis of *S*-phenyl 3-[(3-oxo-3-phenylsulfanyl propyl) disulfanyl] propanethioate (EX-1)

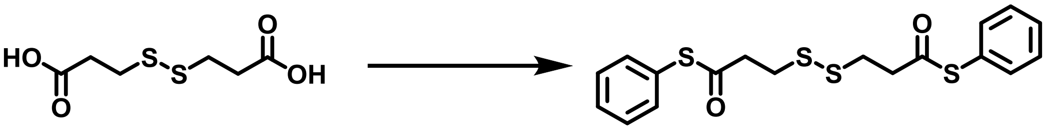

3,3-dithiodipropionic acid **EX-1**

To a solution of 3,3'-Dithiopropionic acid (1.0 g, 5.0 mmol) in 10 mL of THF and 0.01 mL of DMF, pyridine (4.0 mL, 50 mmol) and oxalyl chloride (1.5 mL, 15.0 mmol) were added dropwise at 0 ˚C, and the resulting mixture was stirred at room temperature for 2 h. After adding thiophenol (1.53 mL, 15.0 mmol) dropwise to the above solution, and the resulting mixture was further stirred at room temperature for 2 hours. The reaction solution was concentrated under reduced pressure and purified by column chromatography with hexane/ethyl acetate to obtain *S*-phenyl 3-[(3-oxo-3-phenylsulfanyl propyl) disulfanyl] propanethioate (1.77 g, 4.5 mmol, 90%) as a target product (**EX-1**).

^{1}H NMR (400 MHz, $CDCl_3$, $CHCl_3$ = 7.26) δ 3.00 (m, 4H), 3.08 (m, 4H), 7.42 (s, 10H); ^{13}C NMR (100 MHz, $CDCl_3$, $CDCl_3$ = 77.00) δ 33.2, 43.0, 127.4, 129.4, 129.7, 134.6, 195.7; MS (ESI) *m/z* $(M+H)^+$ 395.1.

1-1-3 Synthesis of *S*-phenyl 3 sulfanylpropanethioate (EX-2)

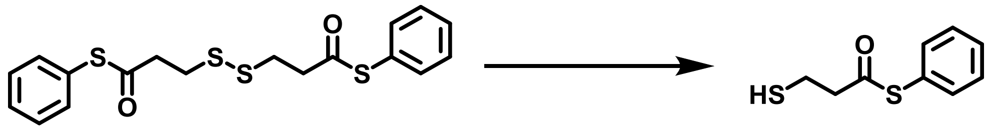

EX-1 **EX-2**

To a solution of *S*-phenyl 3-[(3-oxo-3-phenylsulfanyl propyl) disulfanyl] propanethioate (**EX-1**, 1.77 g, 4.5 mmol) in 5.0 mL of water and 1.0 mL of DMSO, tris(2-carboxyethyl) phosphine hydrochloride (1.93 g, 6.75 mmol) was added at room temperature. After 2 h stirring, this reaction mixture was extracted with ethyl acetate to obtain *S*-phenyl 3 sulfanylpropanethioate (**EX-2**, 1.29 g as crude product) as a target product, which was used to the next reaction without further purification.

1-1-4 Synthesis of 4-oxo-4-(3-oxo-3-phenylsulfanyl-propyl) sulfanyl-butanoic acid (EX-3)

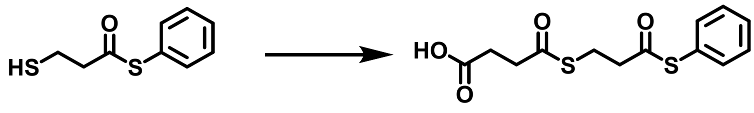

EX-2 EX-3

To a solution of crude *S*-phenyl 3 sulfanylpropanethioate (**EX-2**, 1.29 g) in 2.7 mL of acetonitrile and 0.3 mL of pyridine, succinic anhydride (0.65 g, 6.5 mmol) and dimethylaminopyridine (12.2 mg, 0.1 mmol) were added at room temperature. After 1 h stirring, water was added and aqueous layer was washed with ethyl acetate. 1 M aqueous HCl (20 mL) was added at 0 ˚C and the mixture was extracted by ethyl acetate. The organic layer was washed by saturated aqueous solution of NaCl, dried over Na_2SO_4 and concentrated under reduced pressure to obtain 4-oxo-4-(3-oxo-3-phenylsulfanyl-propyl) sulfanyl-butanoic acid (**EX-3**, 1.25 g as crude product) as a target product, which was used to the next reaction without further purification.

1-1-5 Synthesis of AJICAP reagent (1b)

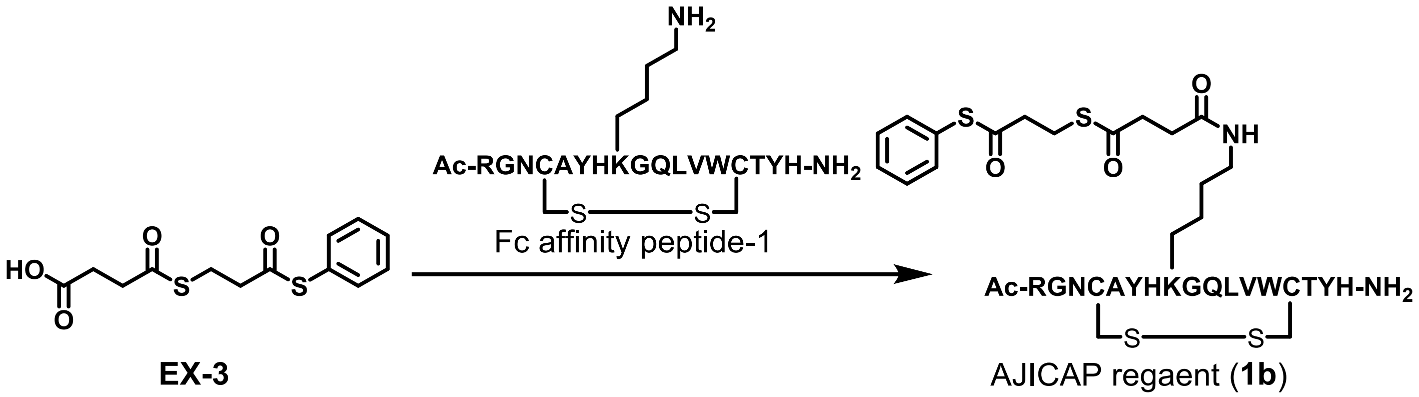

To a solution of known Fc affinity peptide-1[10] (30 mg, 0.0144 mmol) in 1.0 mL of DMF, crude ((4-oxo-4 (3-oxo-3-phenylsulfanyl-propyl) sulfanyl-butanoic acid (**EX-3**, 64 mg) and WSC-HCl (41 mg, 0.22 mmol) were added at room temperature. To this reaction mixture was added 4 mL of 0.05% TFA in water and the product was purified by preparative reverse phase chromatography based on previously reported procedure[10]. The fractions containing the desired AJICAP reagent (**1b**) were collected and combined. Acetonitrile and volatile organics were removed under reduced pressure and water was removed by sublimation to obtain target molecule **1b** (22.3 mg, 0.094 mmol) in 65% yield as an amorphous colorless white solid.

MS (ESI) z=2 1178 $[M+2H]^{2+}$, z=3 786 $[M+3H]^{3+}$

Purity: 85.8%

1-1-6 Overview of synthetic process for AJICAP reagent (6b)

AJICAP peptide (**6b**) was synthesized as below protocol (Scheme S2.).

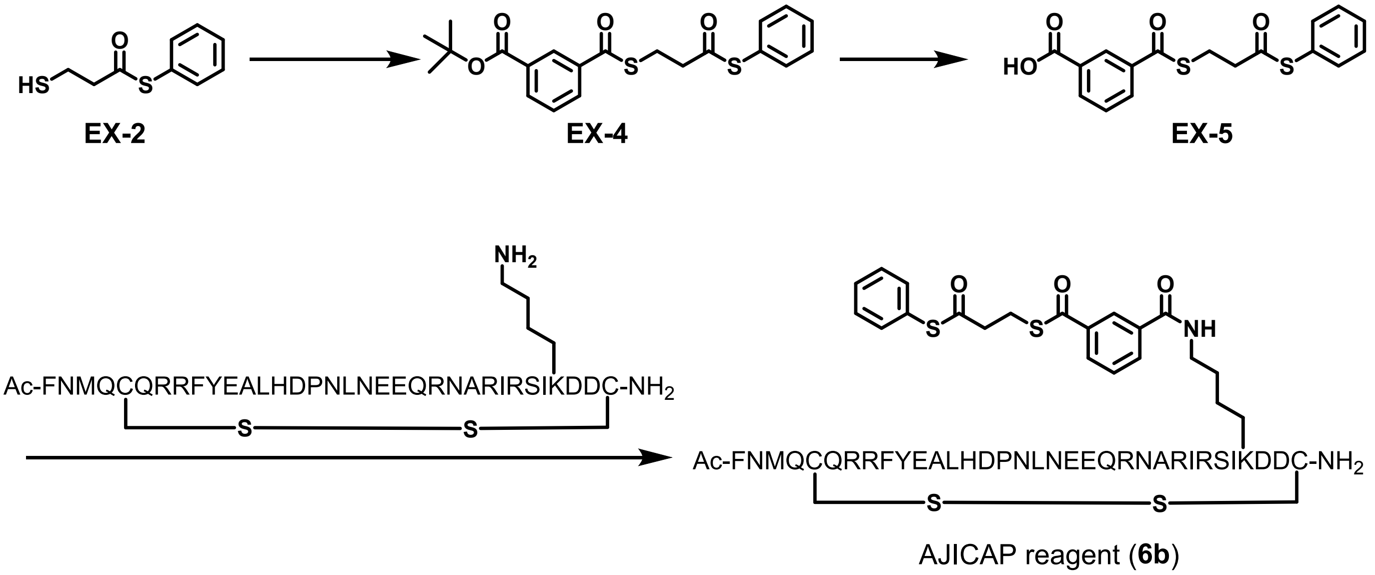

Scheme S2 Synthesis of AJICAP reagent (**6b**)

1-1-7 Synthesis of *tert*-butyl 3-(((3-oxo-3-(phenylthio)propyl)thio)carbonyl)benzoate (EX-4)

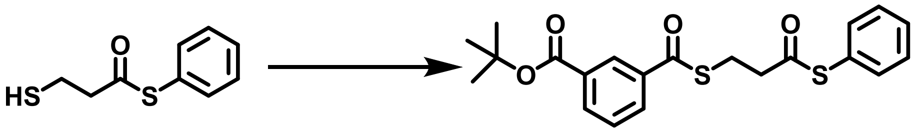

EX-2 EX-4

To a solution of crude *S*-phenyl 3 sulfanylpropanethioate (**EX-2**, 295 mg) in 13.5 mL of CH_2Cl_2, 3- (*tert*-Butyloxycarbonyl) benzoic acid (300 mg, 1.35 mmol), diisopropylethylamine (0.7 mL, 2.03 mmol) and benzotriazol-1-yloxytripyrrolidinophosphonium hexafluorophosphate (843 mg, 1.62 mmol) were added at room temperature. After 1 h stirring, water was added and aqueous layer was washed with ethyl acetate. The organic layer was washed by saturated aqueous solution of NaCl, dried over Na_2SO_4 and concentrated under reduced pressure to obtain *tert*-butyl 3-(((3-oxo-3-(phenylthio)propyl)thio)carbonyl)benzoate (**EX-4**, 259 mg as crude product) as a target product, which was used to the next reaction without further purification.

1-1-8 Synthesis of 3-(((3-oxo-3-(phenylthio)propyl)thio)carbonyl)benzoic acid (EX-5)

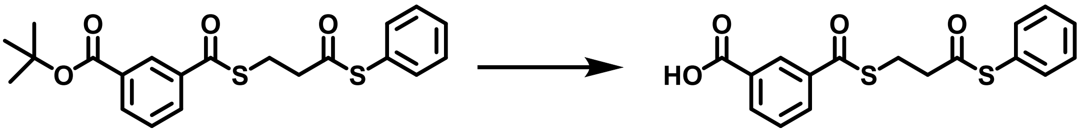

EX-4 **EX-5**

Crude *tert*-butyl 3-(((3-oxo-3-(phenylthio)propyl)thio)carbonyl)benzoate (**EX-4**, 259 mg) was dissolved in a mixed solution of CH_2Cl_2/ TFA = 1/1 (3 mL) and stirred at room temperature for 1 hour. The reaction solution was concentrated and then vacuum dried to obtain the crude compound. This crude compound was filtered through a short column of silica gel. The filtrate was concentrated under reduced pressure to give a 4-oxo-4-(3-oxo-3-phenylsulfanyl-propyl) sulfanyl-butanoic acid (**EX-5**, 227 mg as crude product) as a target product, which was used to the next reaction without further purification.

1-1-9 Synthesis of AJICAP reagent (6b)

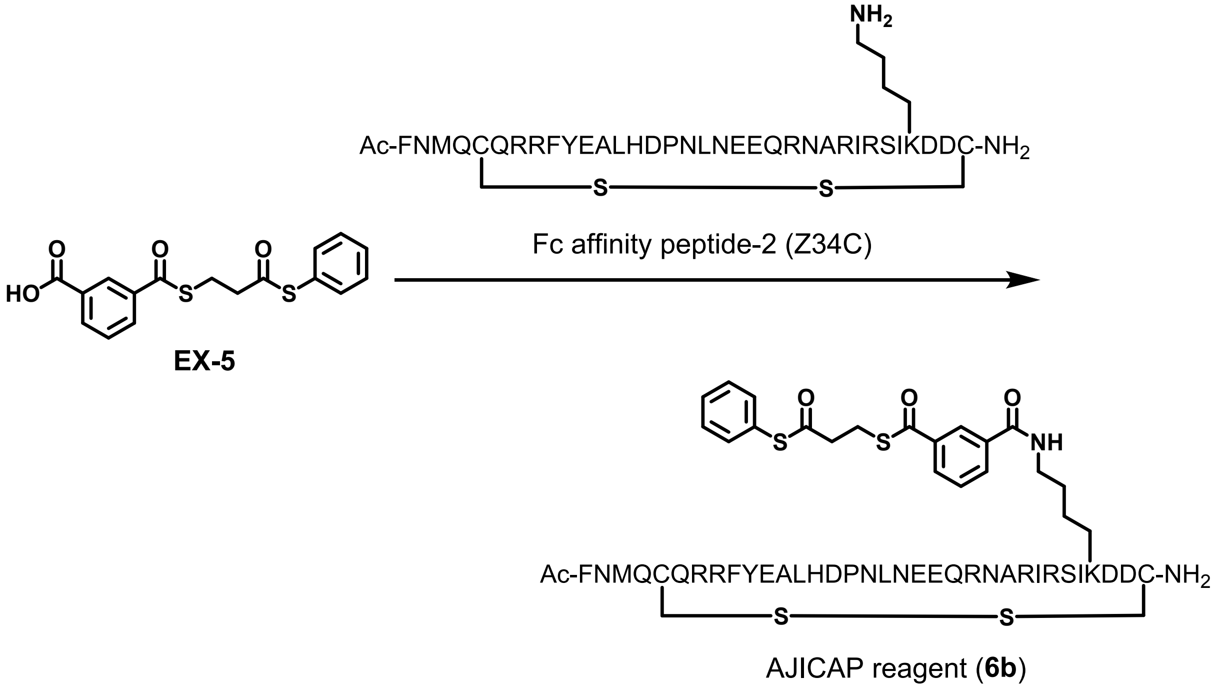

To a solution of Fc affinity peptide-2[10] (30 mg, 0.0071 mmol) in 1.0 mL of DMF, 4-oxo-4-(3-oxo-3-phenylsulfanyl-propyl) sulfanyl-butanoic acid (**EX-5**, 72 mg) and WSC-HCl (41 mg, 0.22 mmol) were added at room temperature. To this reaction mixture was added 4 mL of 0.05% TFA in water and the product was purified by preparative reverse phase chromatography based on previously reported procedure[10]. The fractions containing the desired AJICAP reagent (**6b**) were collected and combined. Acetonitrile and volatile organics were removed under reduced pressure and water was removed by sublimation to obtain target molecule **6b** (10.1 mg, 0.022 mmol) in 31% yield as an amorphous colorless white solid.

MS (ESI) m/z: z=3 1527 $[M+3H]^{3+}$, z=4 1146 $[M+4H]^{4+}$

Purity: 89.3%

1-1-10 Synthesis of AJICAP reagent (6c)

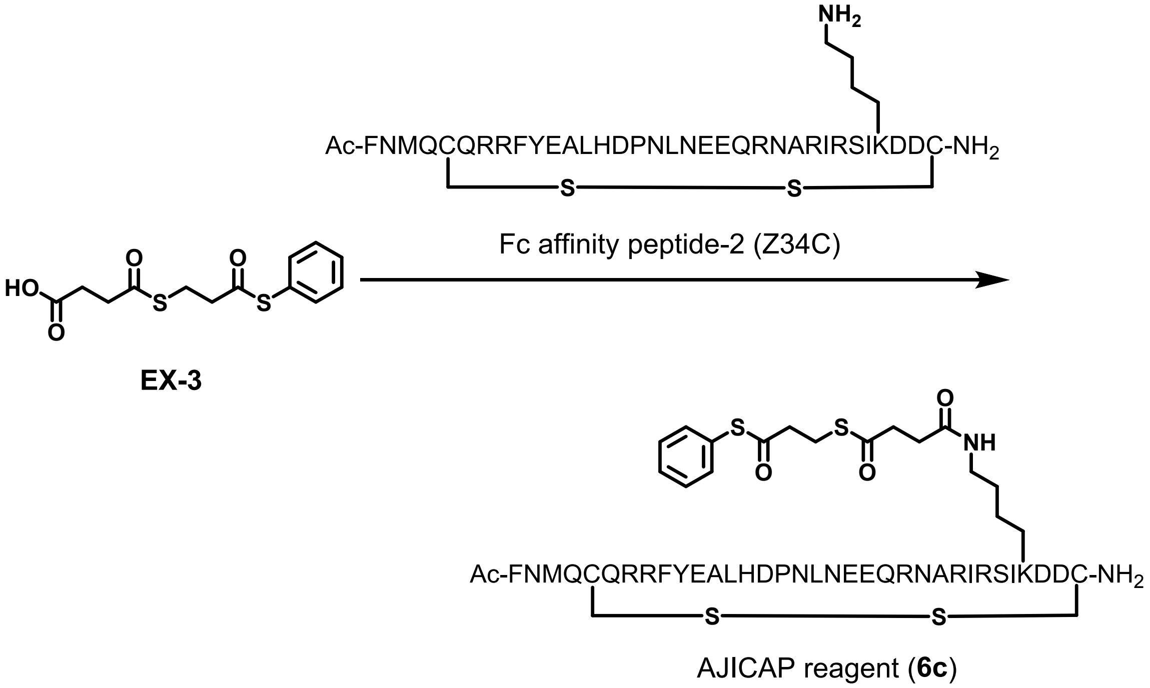

To a solution of Fc affinity peptide-2[10] (30 mg, 0.0071 mmol) in 1.0 mL of DMF, crude ((4-oxo-4 (3-oxo-3-phenylsulfanyl-propyl) sulfanyl-butanoic acid (**EX-3**, 64 mg) and WSC-HCl (41 mg, 0.22 mmol) were added at room temperature. To this reaction mixture was added 4 mL of 0.05% TFA in water and the product was purified by preparative reverse phase chromatography based on previously reported procedure[10]. The fractions containing the desired AJICAP reagent (**6c**) were collected and combined. Acetonitrile and volatile organics

were removed under reduced pressure and water was removed by sublimation to obtain target molecule **6c** (10.3 mg, 0.023 mmol) in 32% yield as an amorphous colorless white solid.

MS (ESI) m/z: z=3 1511 $[M+3H]^{3+}$, z=4 1134 $[M+4H]^{4+}$

Purity: 89.0%

1-2 Molecular modeling

Model structure of Fc-Z34C peptide was generated using Protein Preparation module facilitated by Maestro Suite[21]). Based on crystal structure of Fc-peptide (PDB:6IQG for FcⅢ analog[22], PDB:1L6X for Z34C)[20]), Arg was mutated to Lys, then energy minimized.

1-3 in vivo xenograft study

Cells

NCI-N87 cells (Cat # CRL-5822) were purchased through ATCC. Cells were cultured by a previously established procedure[13].

Animals

NOD.CB17 homozygous mice were procured, fed, and housed by a previously established procedure[13].

Implantation

Implantation was performed as previously reported.[13]

Study Arms and Treatments for NCI-N87

Tumor volumes were monitored, and on first day (when mean tumor volume reached ~120mm^3), mice were stratified and placed into 3 treatment groups of (10) mice as outlined in Table S1, S2.

Table S1. Study arms in NCI-N87 first run.

Group	N	Agent	mg/kg
1	10	Vehicle	
2	10	Trastuzumab-Lys248-MMAE (**5a**)	5
3	10	Trastuzumab-Lys248-MMAE (**5a**)	2.5

Table S2. Study arms in NCI-N87 second run.

Group	N	Agent	mg/kg
1	10	Vehicle	
2	10	Trastuzumab-Lys288-MMAE (**5b**)	5
3	10	Trastuzumab-Lys288-MMAE (**5b**)	2.5

Treatments were administered by tail vein injection (100 µL volumes). Doses were administered on day 0, 4, 7, 11 for a total of 4 doses for the study. Animal weights and tumor volumes were measured.

1-4 Rat PK study

Rat PK study (ELISA method) was performed as previously reported.[13]

1-5 Rat safety study

Animal experiments

Sixty 8-week-old female Sprague-Dawley rats (Charles River Japan, Tokyo, Japan) were provided with a standard diet (Oriental Yeast, Tokyo, Japan) and water ad libitum. Following the acclimatization period (1 week), animals were stratified by body weight and randomly assigned as the control group that were treated with the vehicle (histidine buffer), trastuzumab-Lys288-MMAE (**5b**) that was treated with 80 mg/kg. Each group was consisted of 5 animals for body weight measurement. All rats were administered once via the caudal vein with a butterfly needle for intravenous injection, a 30 mL polypropylene syringe, and a syringe pump (Pump 11 Elite, Harvard Apparatus). The experimental procedure was approved by the institutional ethics committee.

Clinical observations

The animals were observed once daily for clinical signs. Individual body weight were measured on Days 0, 1, 2, 3, 4, 5, 6, 7, 8, 9, 10, 11, 12, 13, and 14 with the first day of administration defined as Day 0.

1-6 Synthesis of novel format antibody conjugates

1-6-1 Antibody-siRNA conjugate production

Sequence of siRNA-maleimide prepration

The following sequences of siRNA were used.

Sense strand: 5'-6-FAM-A (M) C (M) AGC (M) AAAU (M) U (M) C (M) C (M) AU (M) C (M) GU (M) GU (M) -N (6)

Antisense strand: 5′-p-AC (F) AC (F) GAU (F) GGAAU (F) U (F) U (F) GC (F) U (F) GU (F) UU

P: Phosphorylation, (F): 2′-Fluoro, (M): 2′-O-Me (methyl), N (6): amino C6 linker added to the 3 ′ end

Si RNA-maleimide preparation

To a solution of siRNA in PBS buffer (pH 7.4), 3-(Maleimido) propionic acid N-succinimidyl ester (100 eq, 200 mM, dissolved in DMF) was added. The mixture was stirred at 4 ° C. for 20 h, and then purified using NAP-5 Columns (GE Healthcare).

Antibody-siRNA conjugates production

Conjugation was performed as ADC synthesis[14]. Purification by ÄKTA purifier (GE Healthcare) was performed under the following conditions.

Column: Superdex 200 Increase 10 / 300GL (GE Healthcare)

Buffer: pH 7.4 PBS buffer flow rate: 0.4 ml / min

Detector: Detected at wavelengths of 215 and 280 nm.

SDS-PAGE analysis

The conjugate was analyzed by SDS-PAGE with previously reported methods[18].

1-7-2 Antibody Protein conjugate production

Lysozyme-azide preparation

To a solution of Lysozyme (pH 7.4 PBS buffer solution), *N*-hydroxysuccinimide 4-azidobenzoate (6 eq, 20 mM DMF solution) was added, and the mixture was stirred at room temperature for 3 h and then purified using NAP-5 column to obtain an azido-incorporating lysozyme.

Antibody-DBCO intermediate synthesis

Conjugation of trastuzumab-Lys248-thiol with DBCO-maleimide (CAS No: 1395786-30-7) was performed as ADC synthesis[14].

Click reaction to produce antibody-protein conjugate

The conjugate was synthesized by click reaction with previously reported methods[13].

2. Experimental procedure

2-1 AJICAP reagents synthesis: 1H, 13C NMR, HPLC, and LC-MS

2-1-1 1H and 13C NMR results

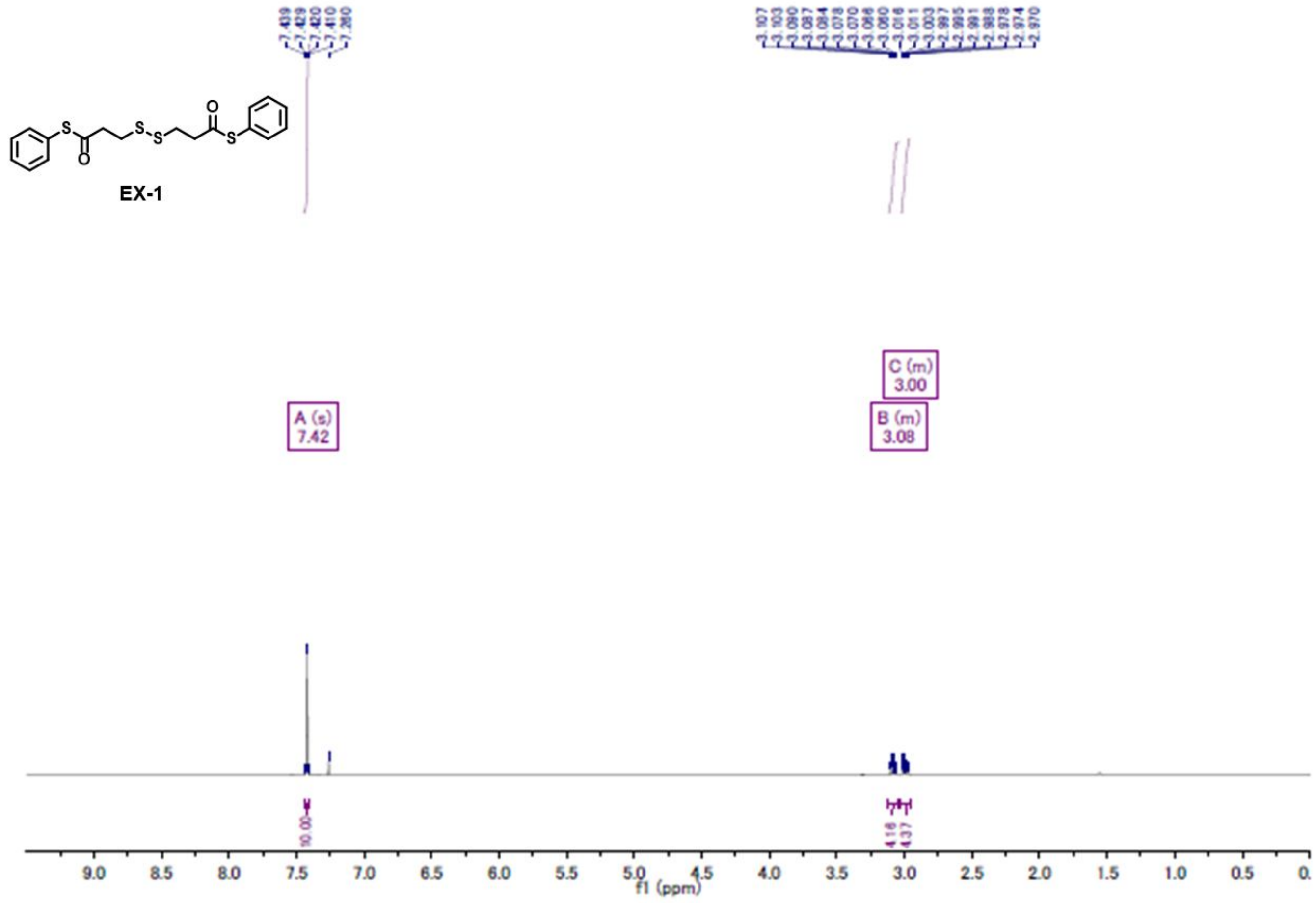

Figure S1. 1H NMR spectra of EX-1

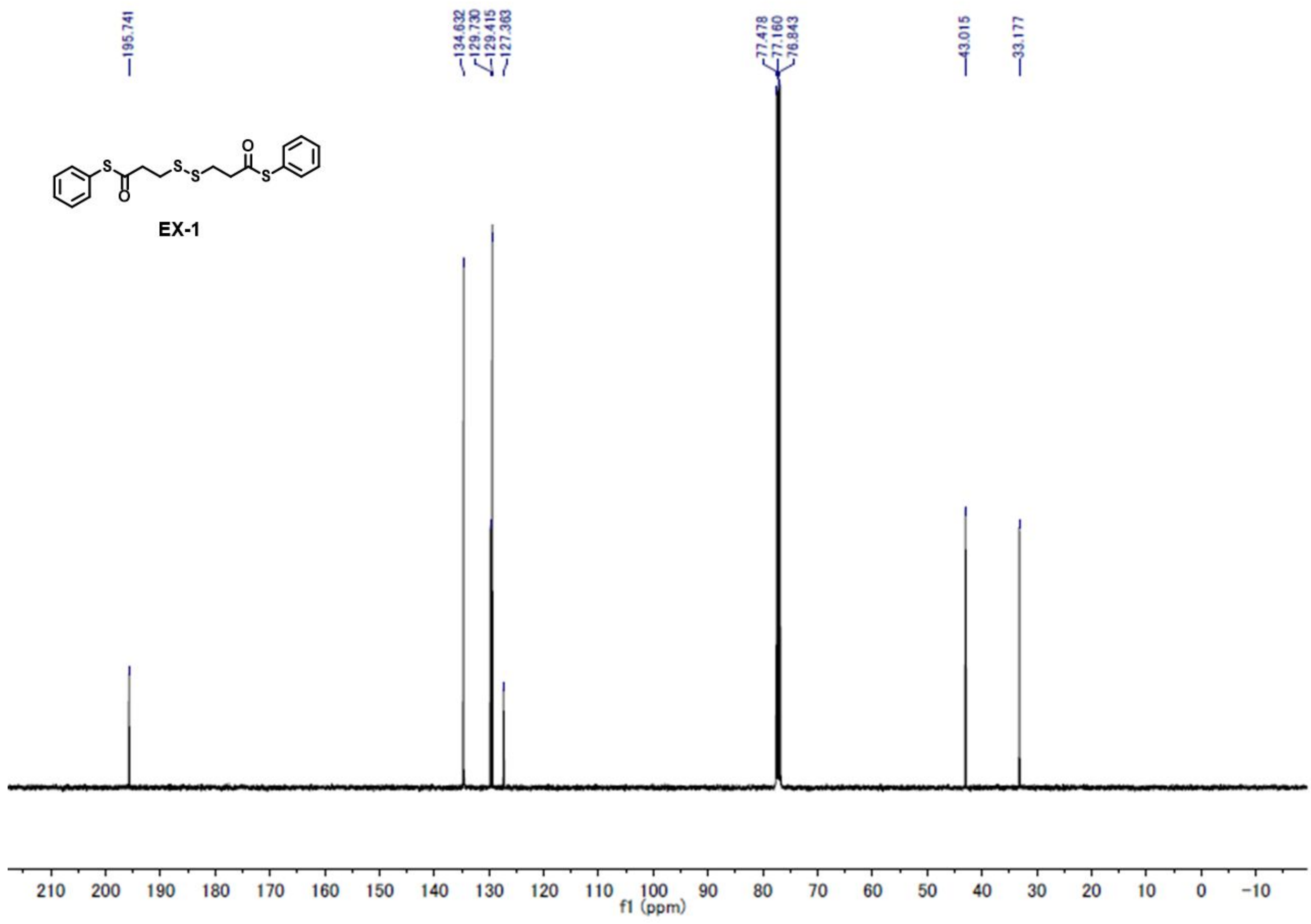

Figure S2. 13C NMR spectra of EX-1

2-1-2 Purity and LC-MS analysis of AJICAP reagents

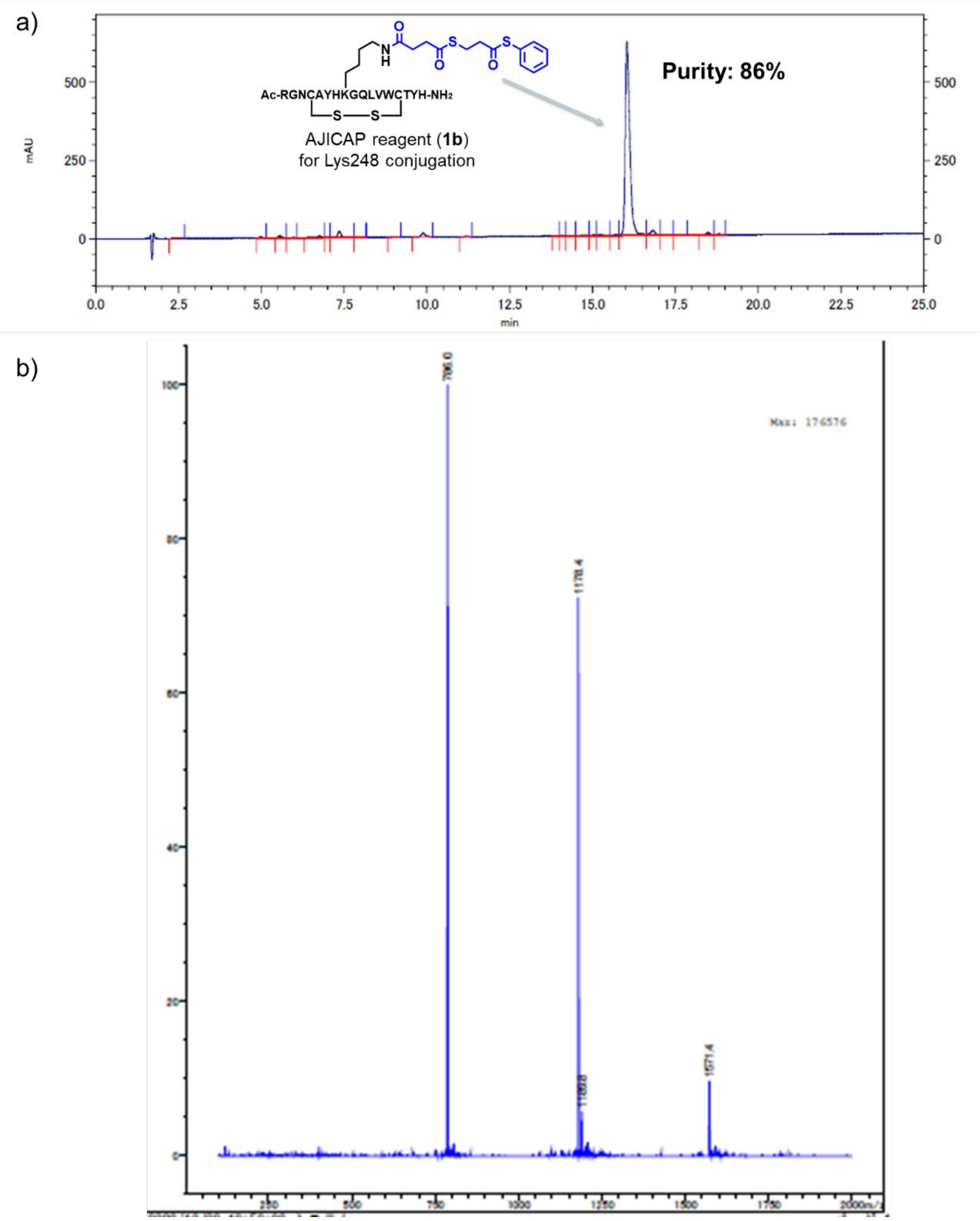

Figure S3. Analysis of AJICAP reagents (**1b**); a) HPLC analysis, b) MS spectrums

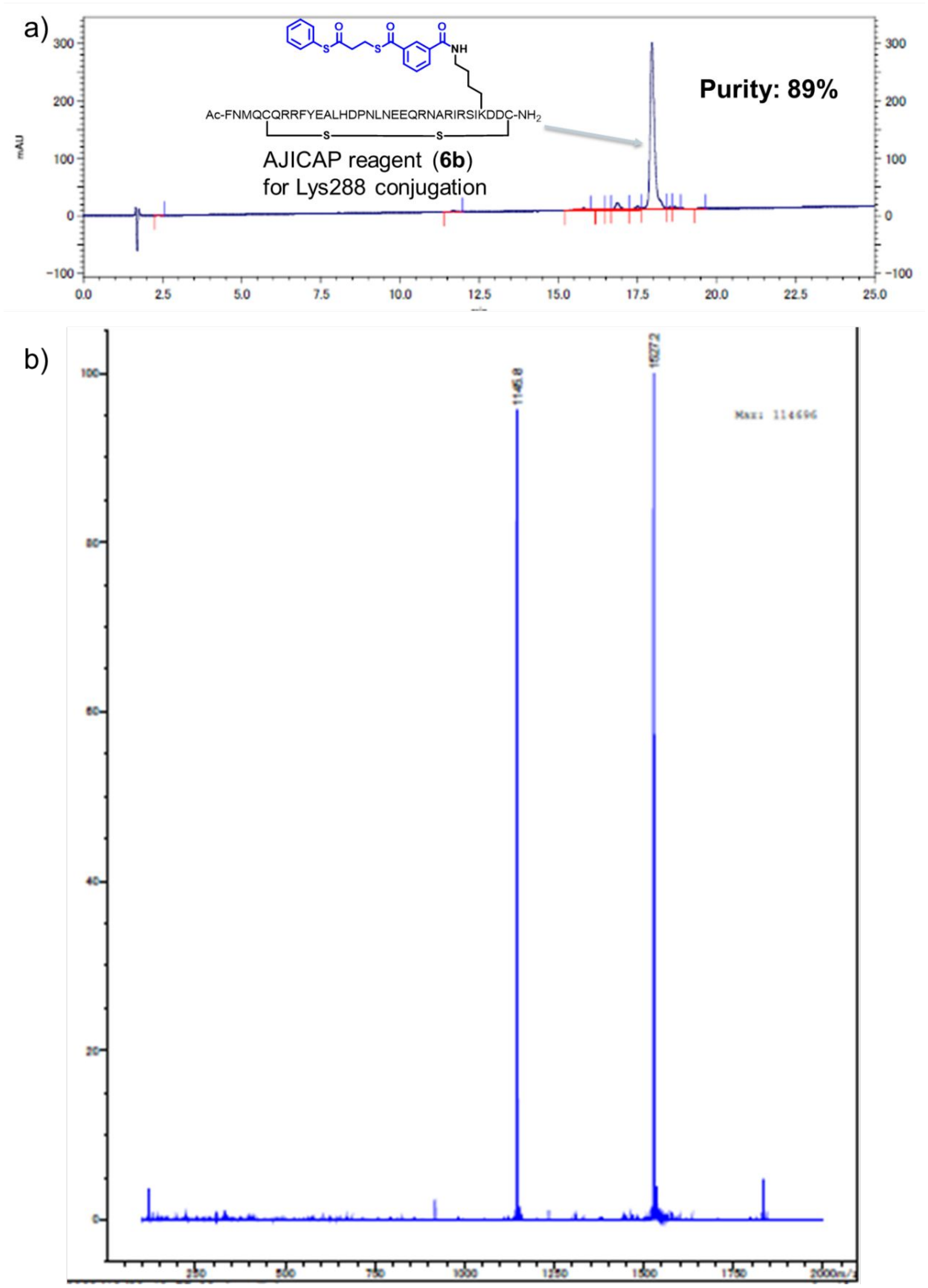

Figure S4. Analysis of AJICAP reagents (**6b**); a) HPLC analysis, b) MS spectrums

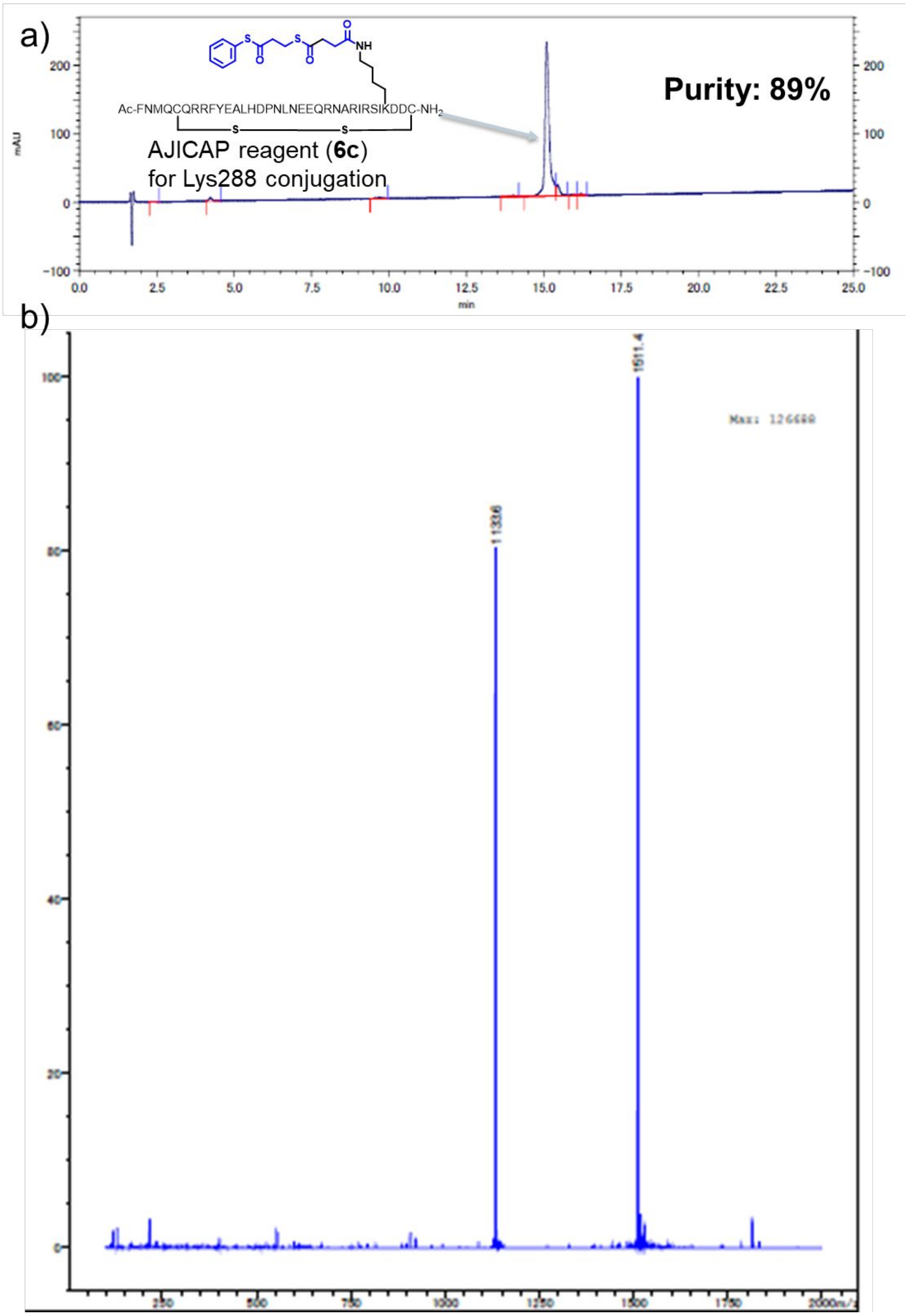

Figure S5. Analysis of AJICAP reagents (**6c**); a) HPLC analysis, b) MS spectrums

2-2 Peptide conjugation of trastuzumab and rituximab: Q-TOF MS and HIC analysis

2-2-1 Q-TOF MS analysis

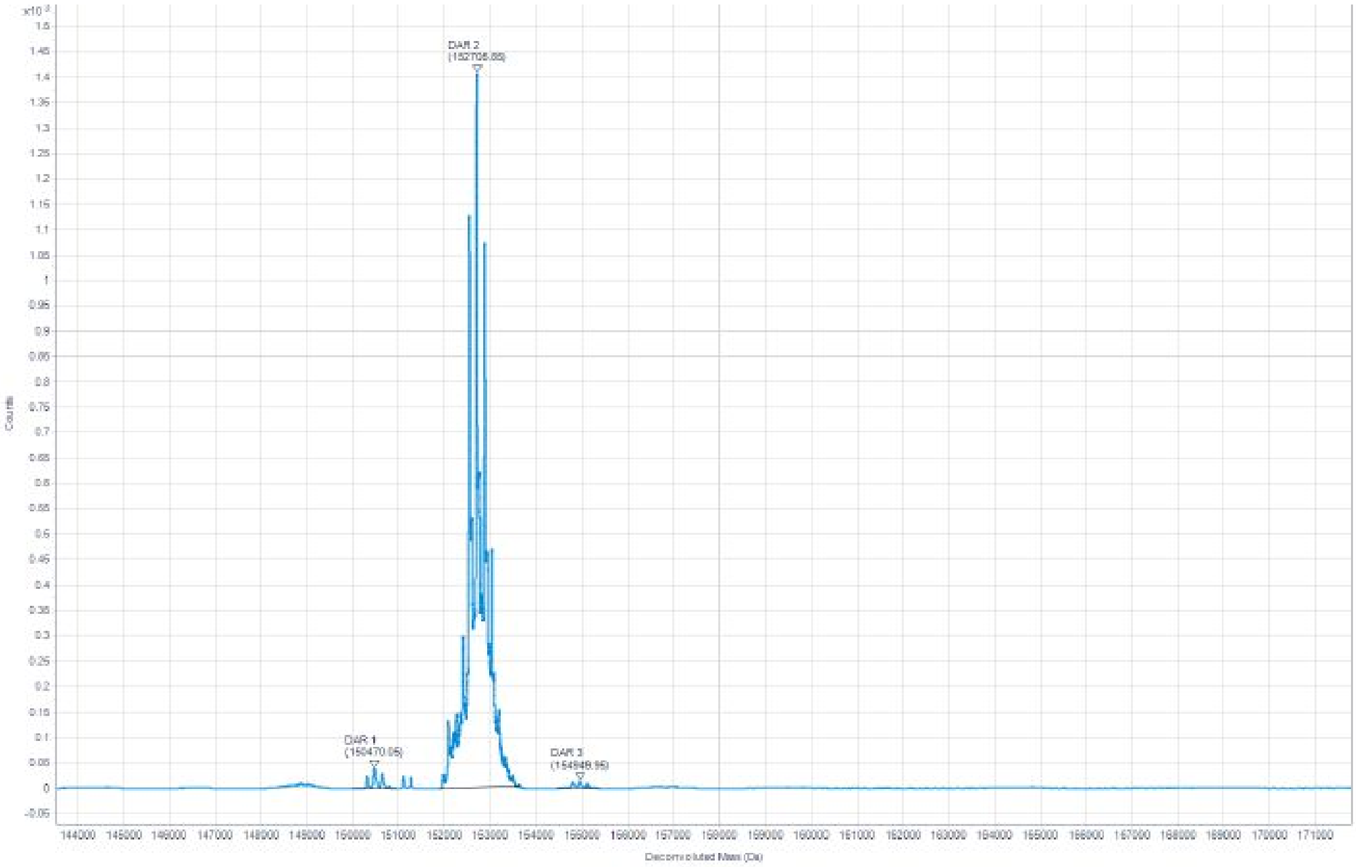

DAR Peak List

DAR Peak	Theoretical Mass (Da)	Observed Mass (Da)	Area	% Area
1	150580	150470.05	5.06E+003	1.38
2	152935	152705.85	3.59E+005	97.90
3	155290	154949.95	2.64E+003	0.72

Figure S6. Q-TOF analysis of AAPC (trastuzumab-Lys248 peptide) derived from AJICAP reagent **1b**

Average PAR = 2.0

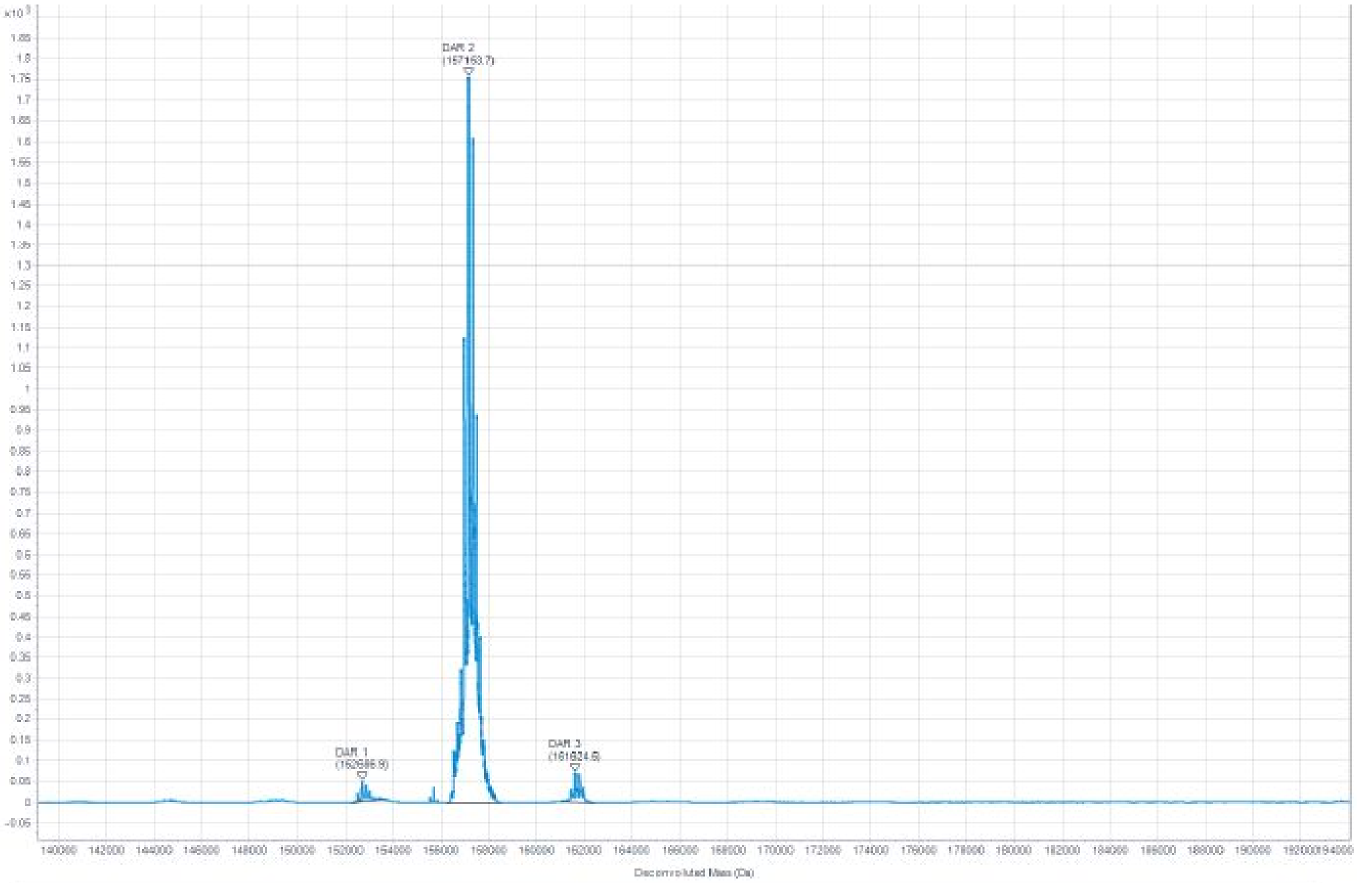

DAR Peak List

DAR Peak	Theoretical Mass (Da)	Observed Mass (Da)	Area	% Area
1	152834	152686.9	8.87E+003	1.68
2	157413	157153.7	5.05E+005	95.43
3	161992	161624.5	1.53E+004	2.89

Figure S7. Q-TOF analysis of AAPC (trastuzumab-Lys288 peptide) derived from AJICAP reagent **6d**

Average PAR = 2.0

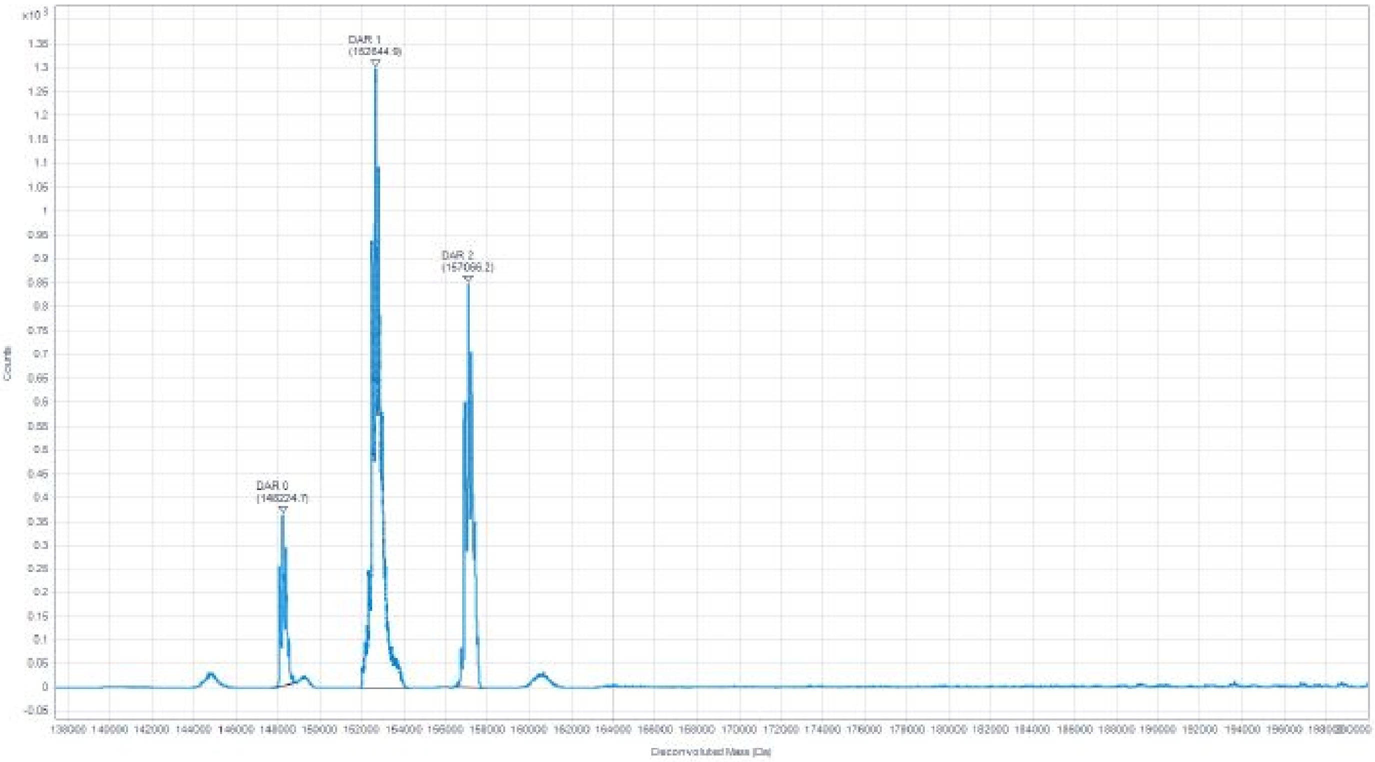

DAR Peak List

DAR Peak	Theoretical Mass (Da)	Observed Mass (Da)	Area	% Area
0	148225	148224.7	8.63E+004	10.00
1	153656	152644.9	5.10E+005	59.08
2	159087	157066.2	2.67E+005	30.92

Figure S8. Q-TOF analysis of AAPC (trastuzumab-Lys288 peptide) derived from AJICAP reagent **6c**

Average PAR = 1.2

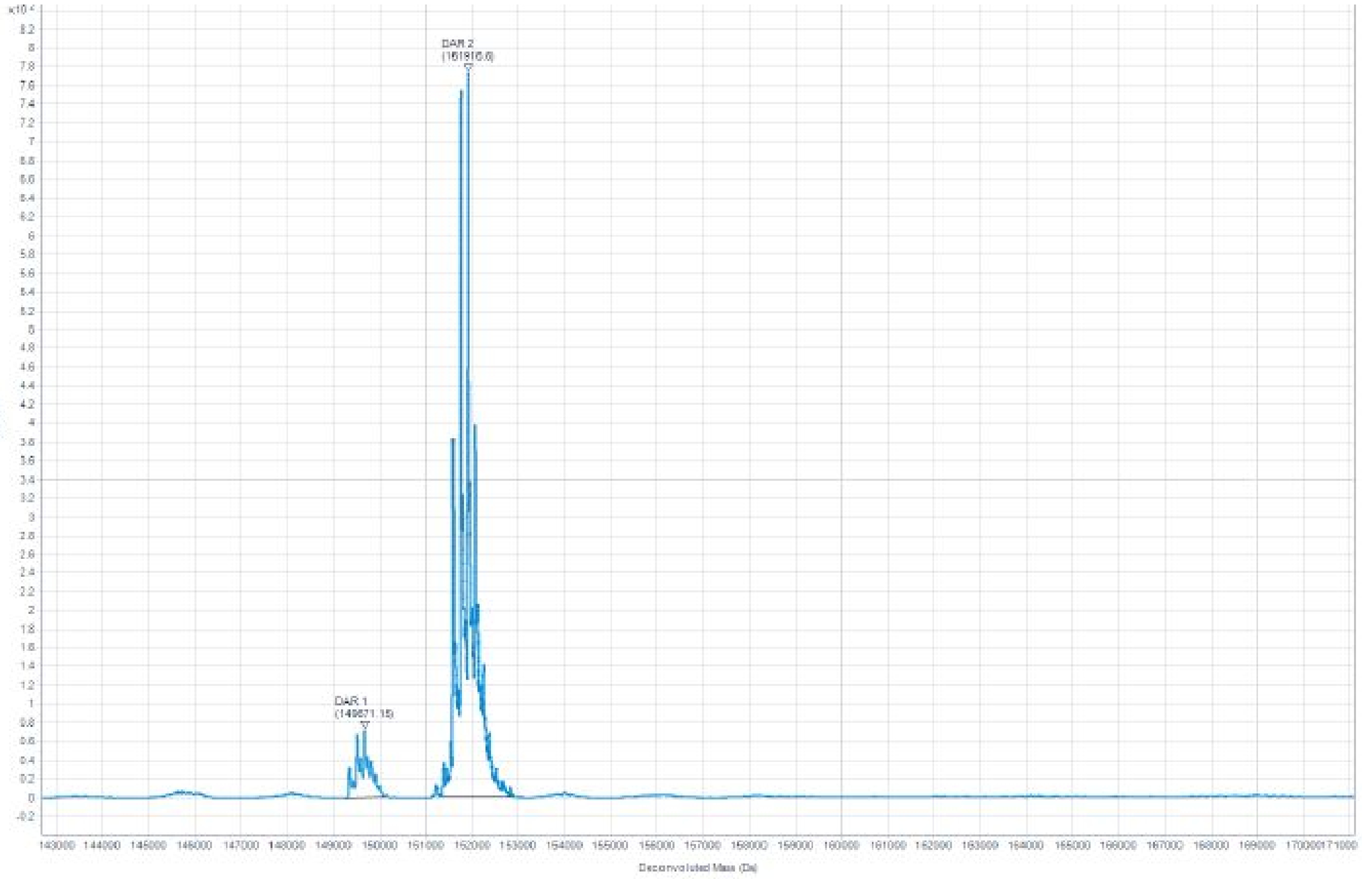

DAR Peak List

DAR Peak	Theoretical Mass (Da)	Observed Mass (Da)	Area	% Area
1	149773	149671.15	1.91E+004	10.42
2	152142	151916.6	1.65E+005	89.58

Figure S9. Q-TOF analysis of AAPC (rituximab-Lys248 peptide) derived from AJICAP reagent **1b**

Average PAR = 1.9

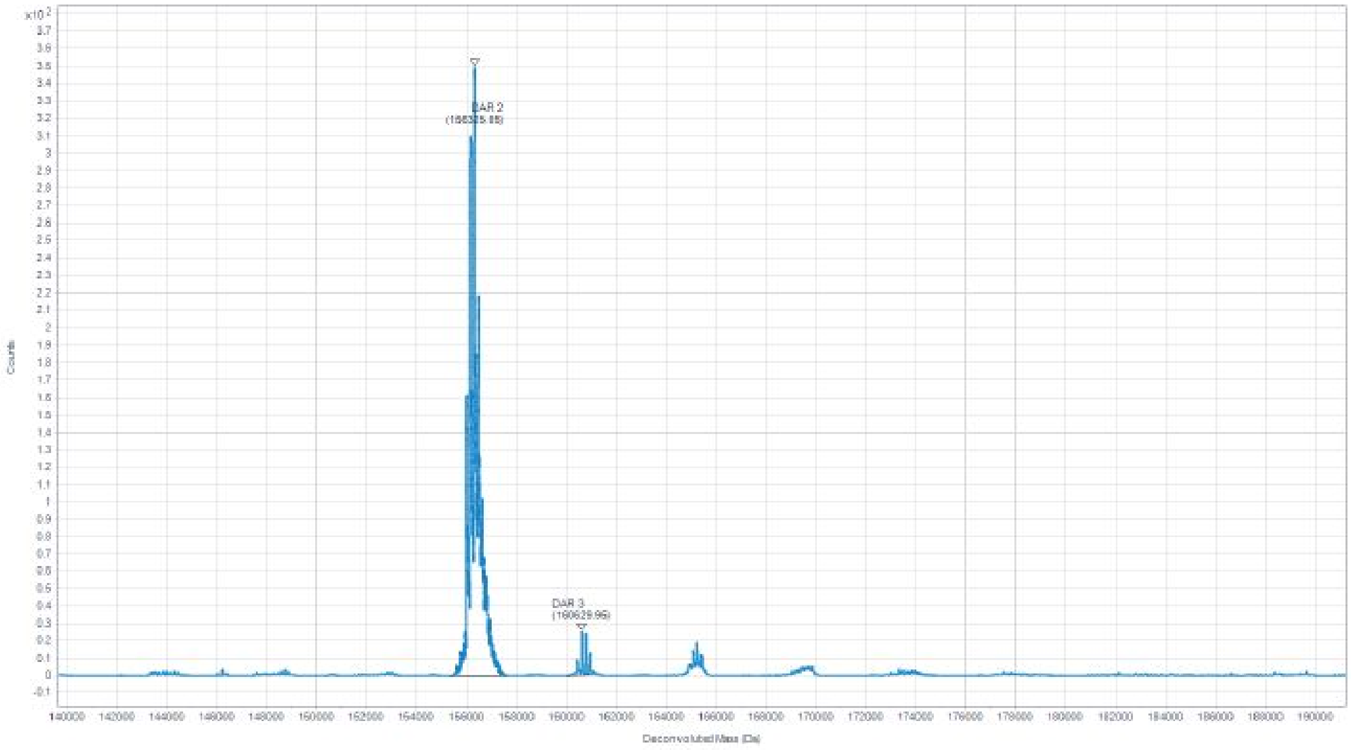

DAR Peak List

DAR Peak	Theoretical Mass (Da)	Observed Mass (Da)	Area	% Area
2	156825	156325.85	9.67E+004	97.32
3	161125	160629.95	2.67E+003	2.68

Figure S10. Q-TOF analysis of AAPC (rituximab-Lys288 peptide) derived from AJICAP reagent **6b**

Average PAR = 2.0

2-2-2 HIC-HPLC analysis

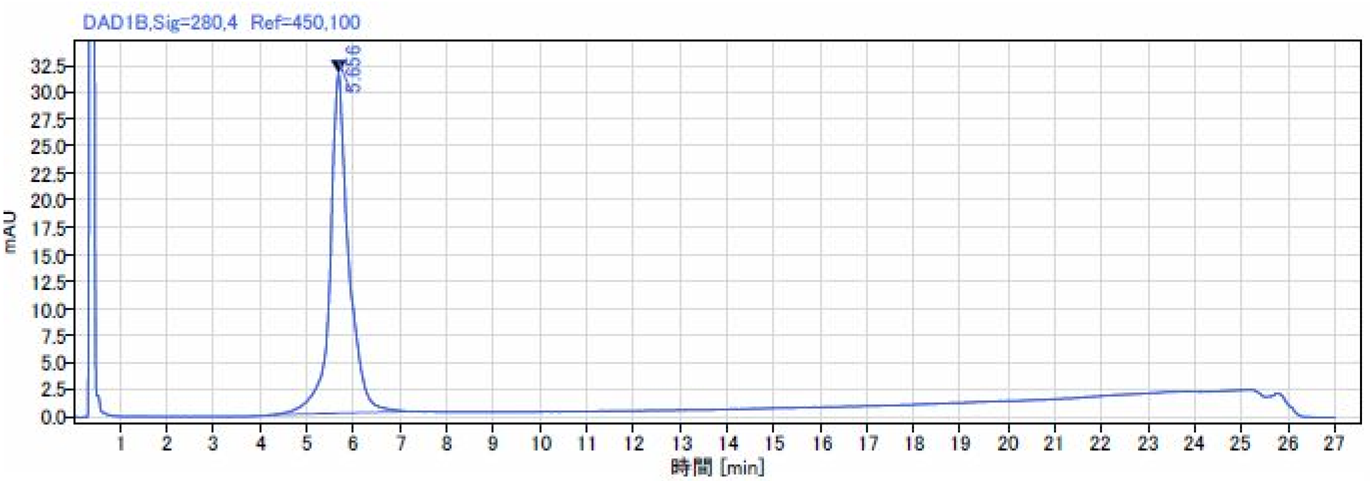

Figure S11. HIC analysis of trastuzumab

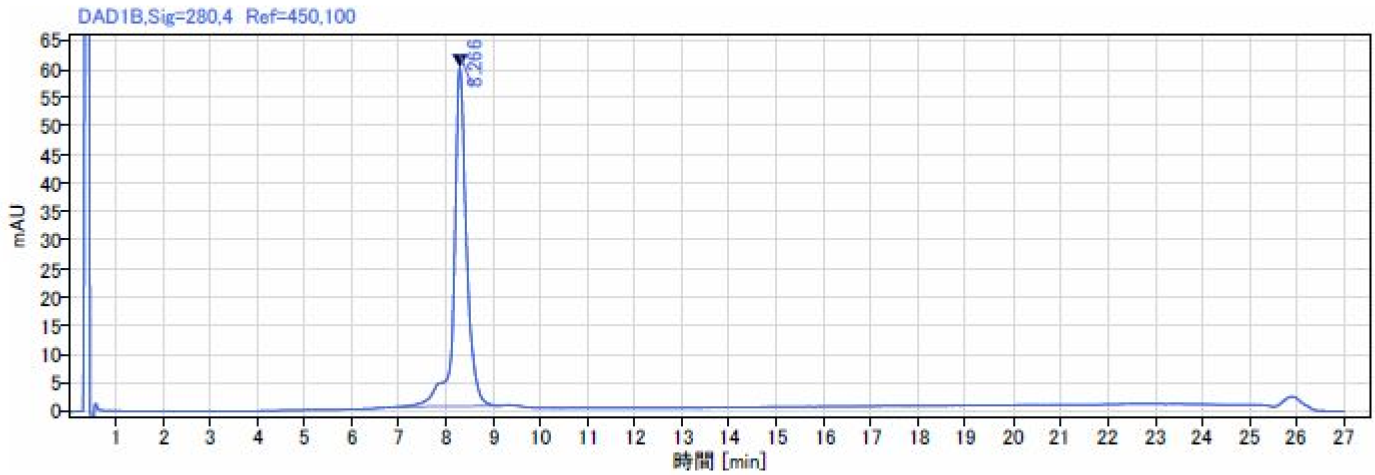

Figure S12. HIC analysis of AAPC (trastuzumab-Lys248 peptide) derived from AJICAP reagent **1b**

Average PAR = 2.0

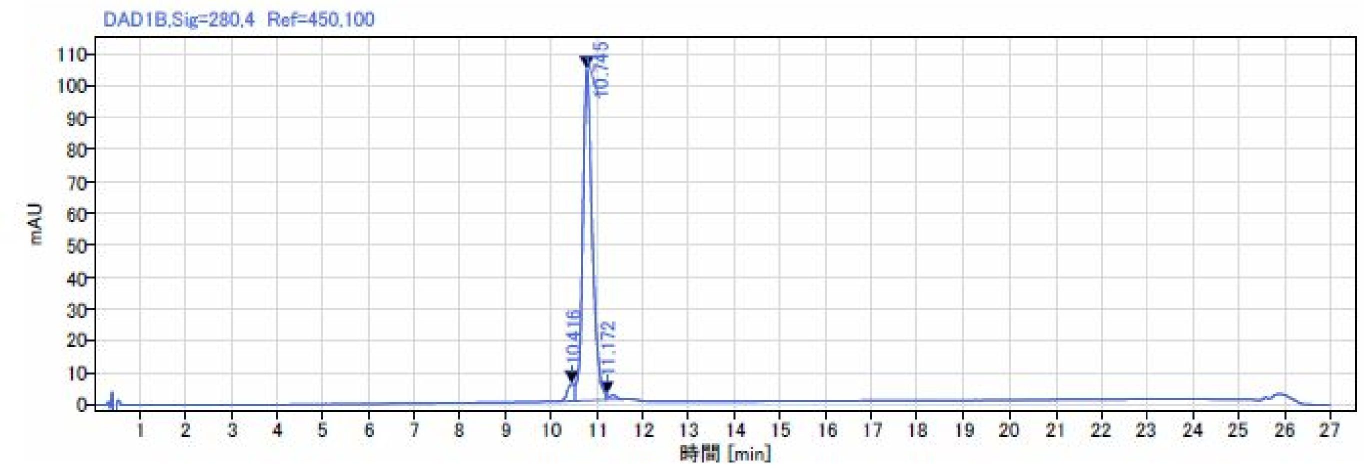

Figure S13. HIC analysis of AAPC (trastuzumab-Lys288 peptide) derived from AJICAP reagent **6b**

Average PAR = 1.9

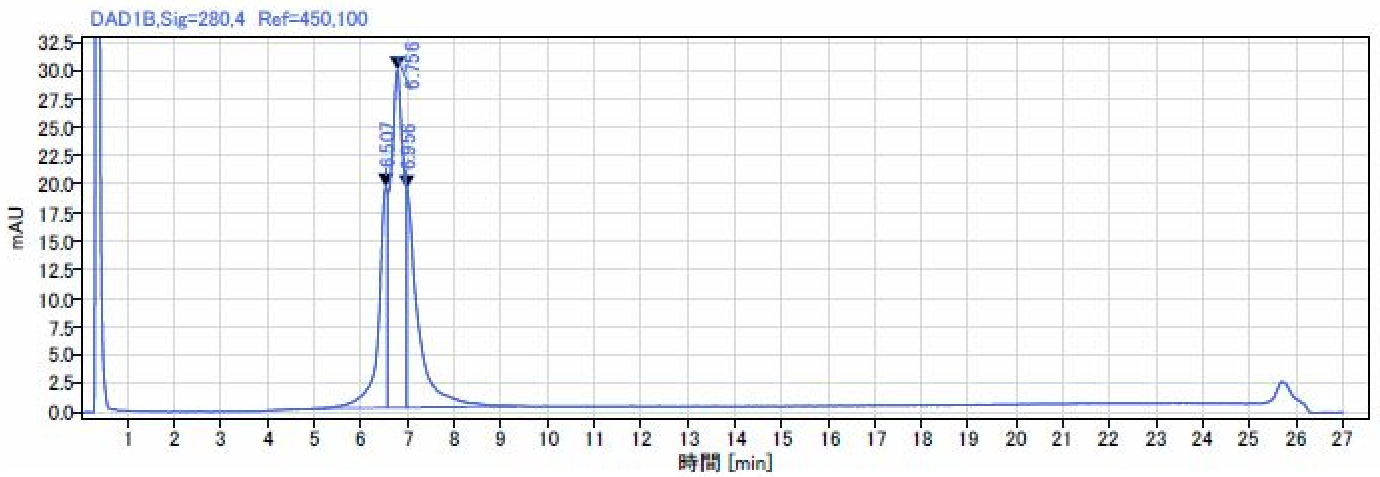

Figure S14. HIC analysis of AAPC (trastuzumab-Lys288 peptide) derived from AJICAP reagent **6c**

Average PAR = 1.1

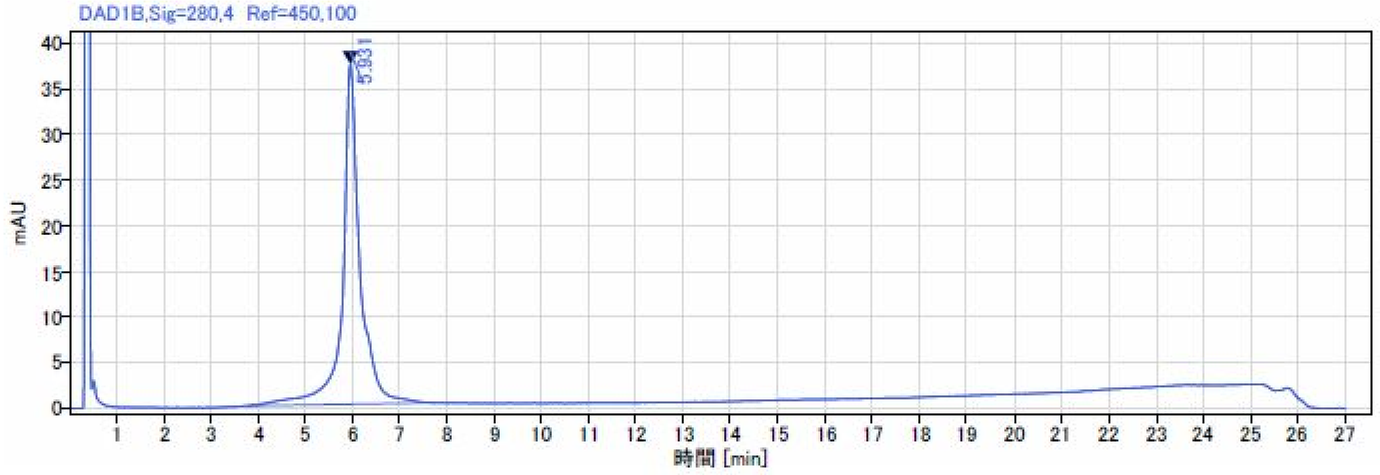

Figure S15. HIC analysis of trastuzumab-Lys248-thiol derived from AJICAP reagent **1b**

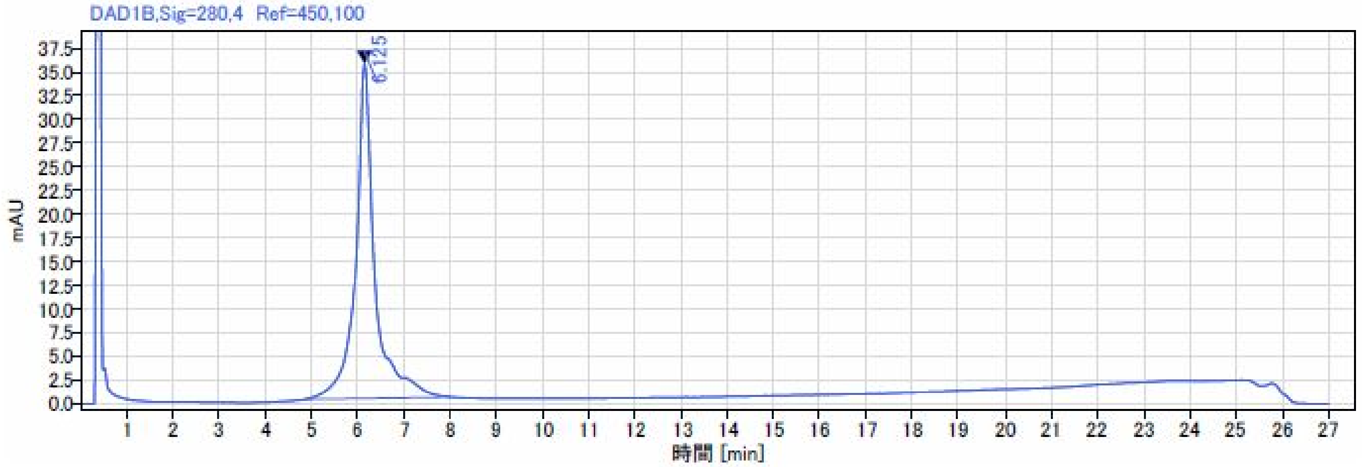

Figure S16. HIC analysis of trastuzumab-Lys288-thiol derived from AJICAP reagent **6b**

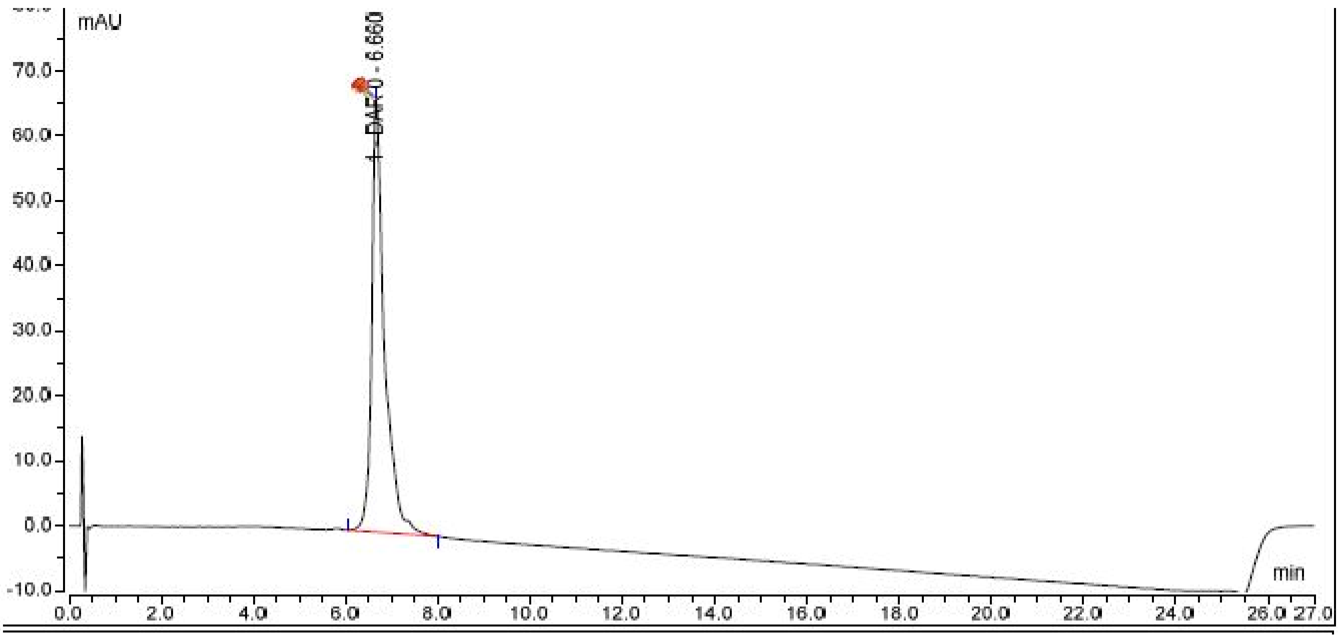

Figure S17. HIC analysis of rituximab

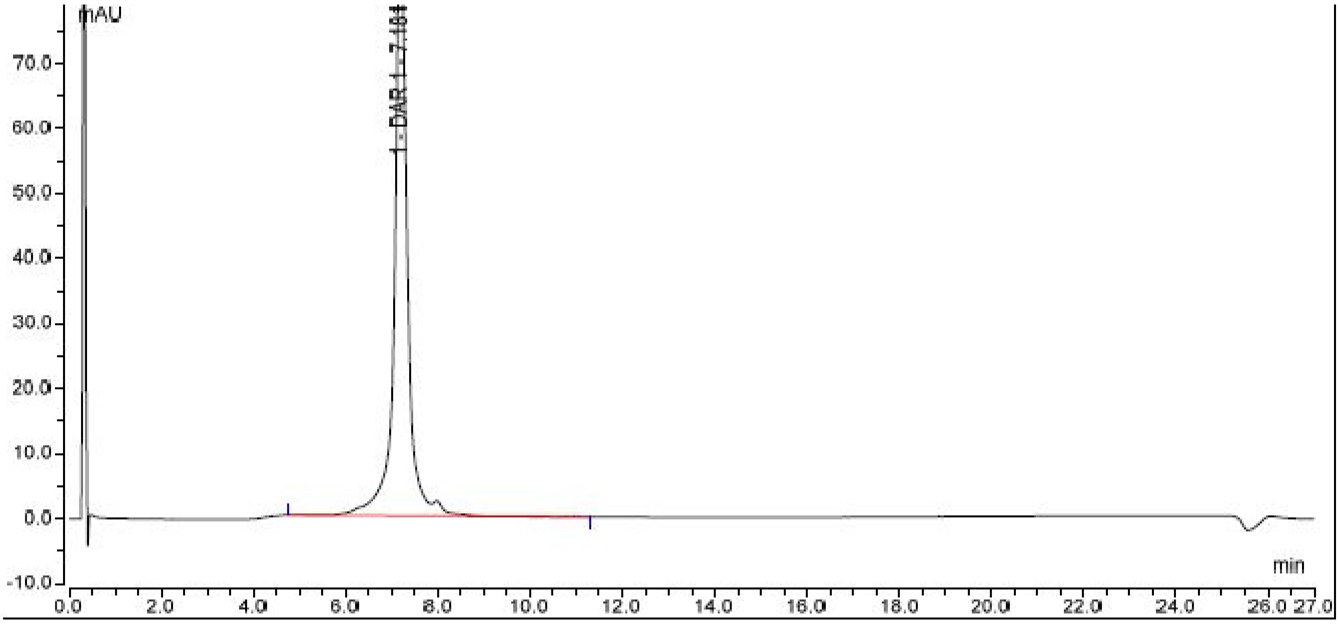

Figure S18. HIC analysis of AAPC (rituximab-Lys248 peptide) derived from AJICAP reagent **1b**

Average PAR = 2.0

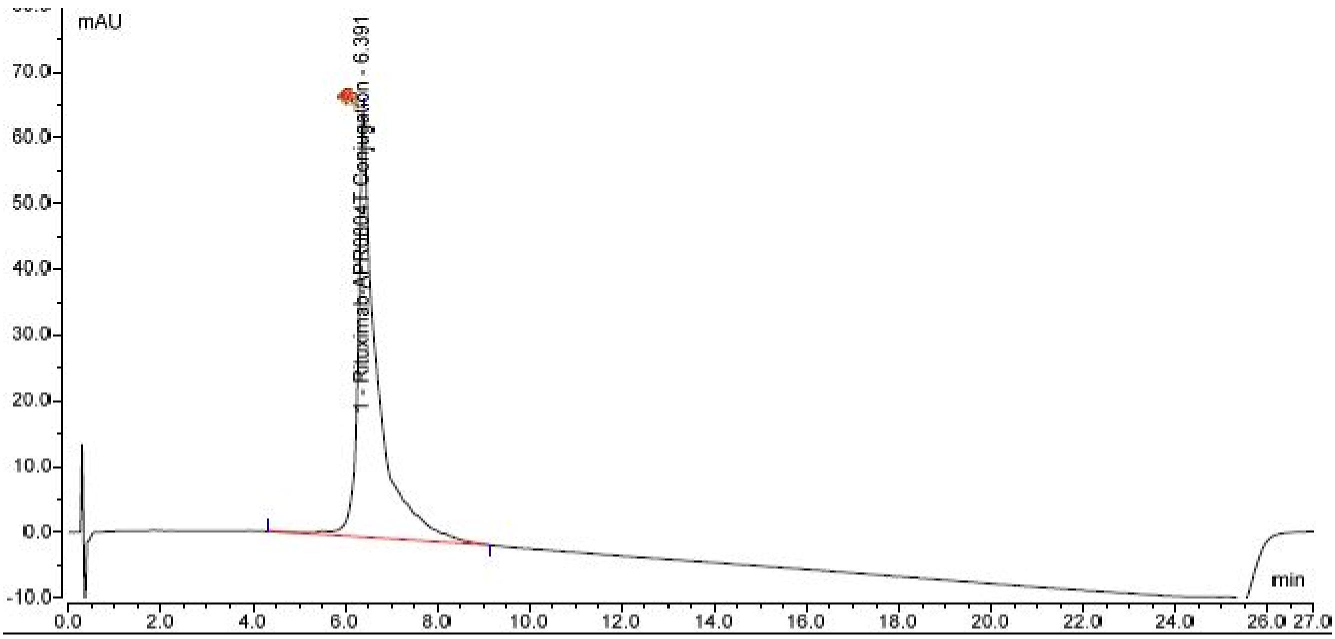

Figure S19. HIC analysis of AAPC (rituximab-Lys288 peptide) derived from AJICAP reagent **6b**

Average PAR = 1.9

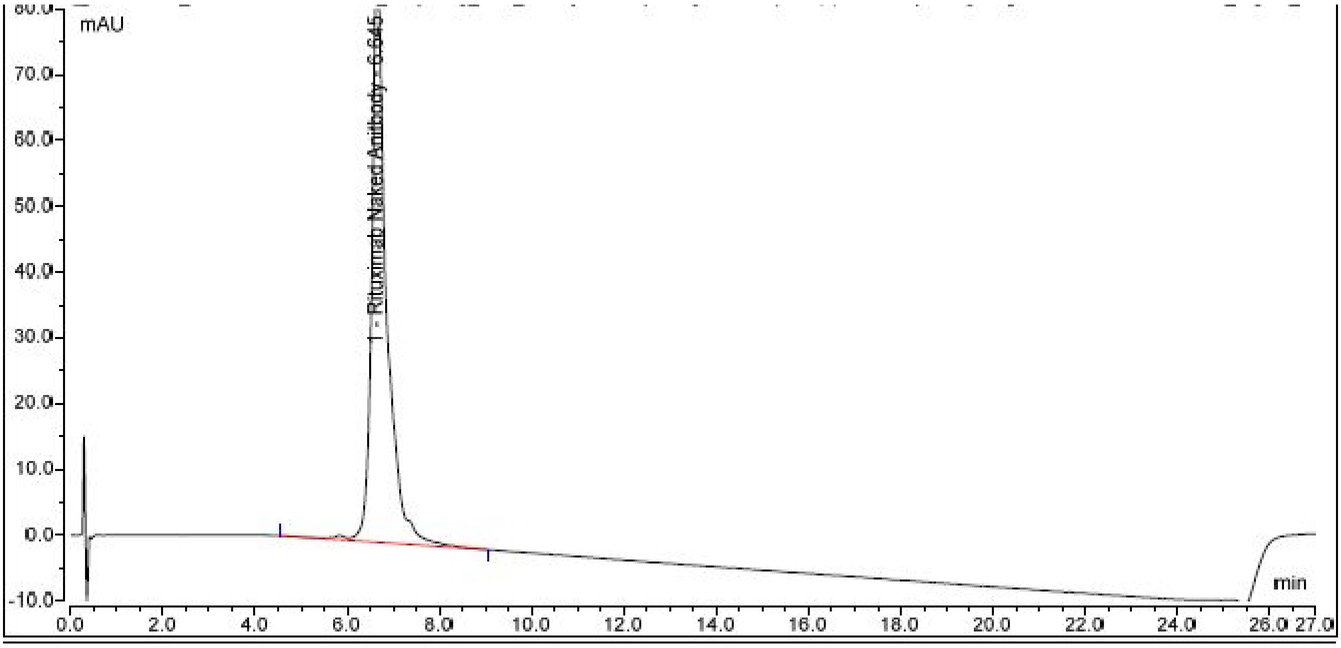

Figure S20. HIC analysis of rituximab-Lys248-thiol derived from AJICAP reagent **1b**

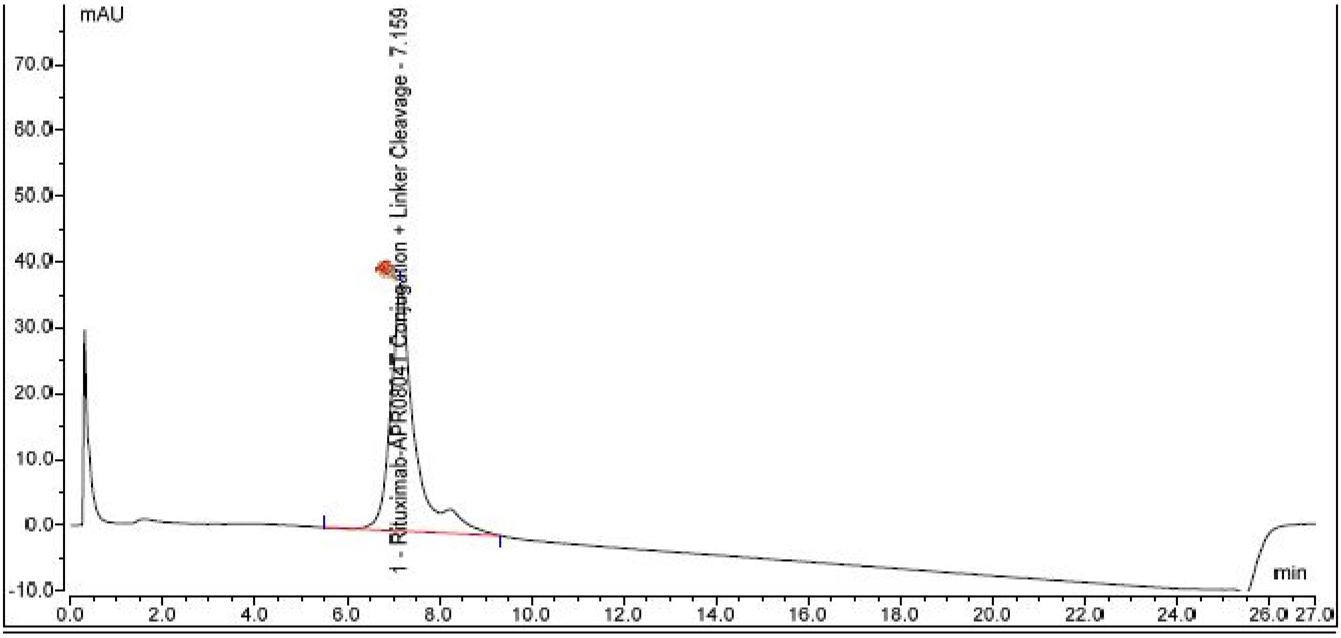

Figure S21. HIC analysis of rituximab-Lys288-thiol derived from AJICAP reagent **6b**

2-3 Molecular modeling of interaction between human IgG1 Fc and the affinity peptides

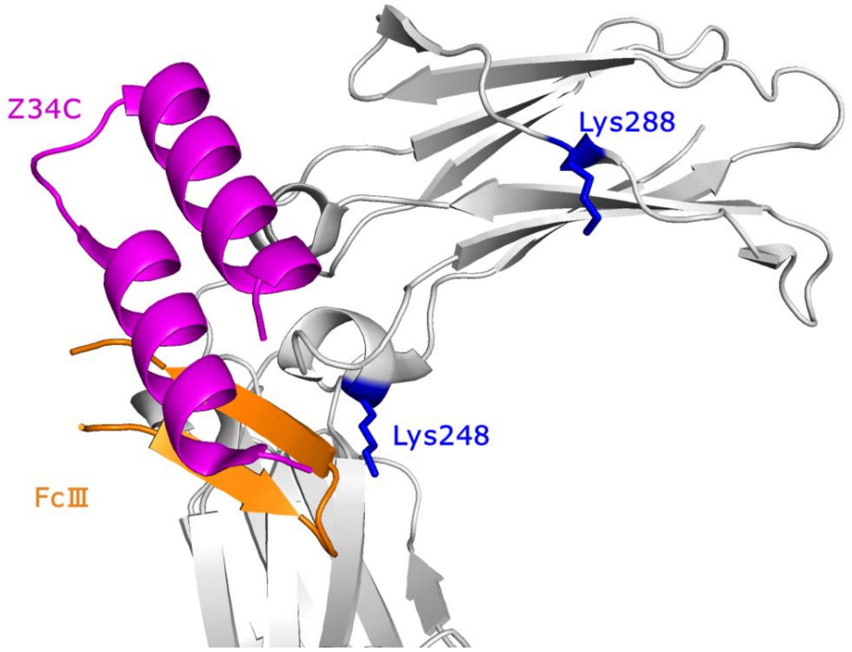

Figure S22. The interaction between human IgG1 Fc and the FcⅢ peptide (PDB: 6IQG), Z34 C peptide (PDB: 1L6X)

2-4 Capability of AJICAP technology: HIC, Q-TOF MS, and SEC analysis

2-4-1 HIC-HPLC analysis

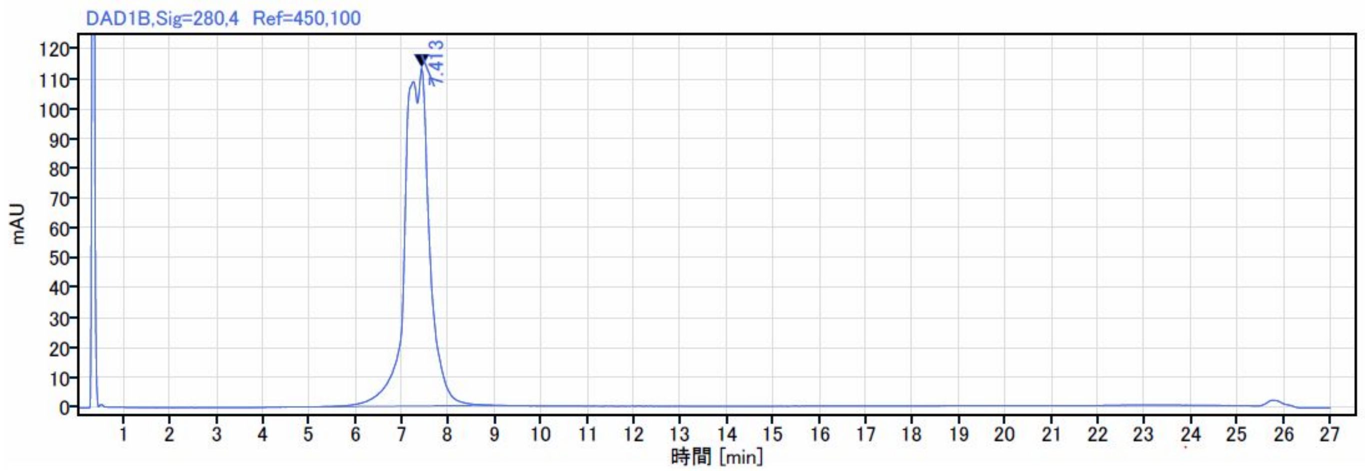

Figure S23. HIC analysis of infliximab

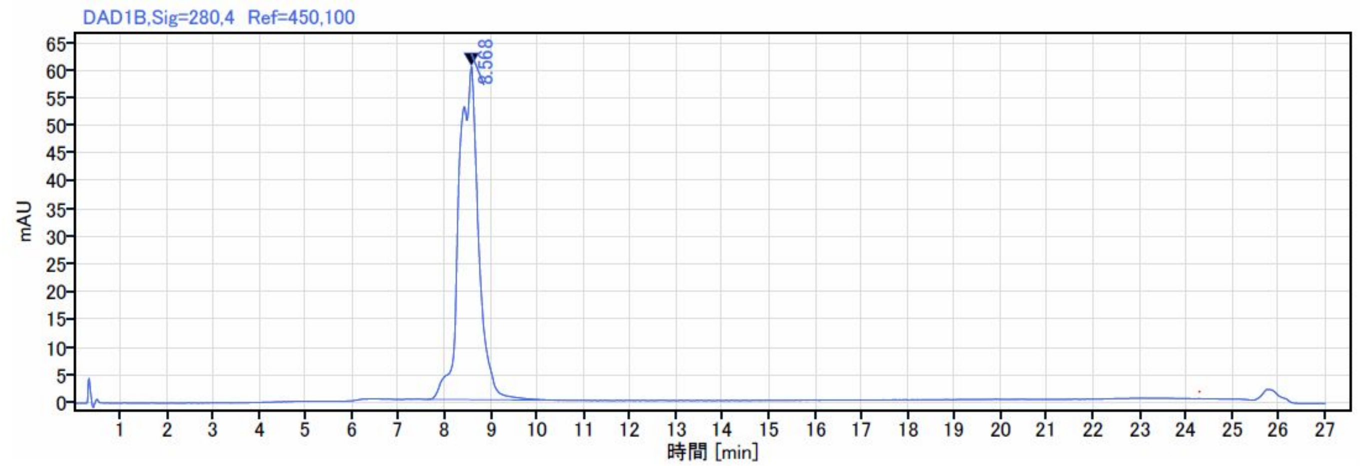

Figure S24. HIC analysis of AAPC (infliximab-Lys248 peptide) derived from AJICAP reagent **1b**

Average PAR = 2.0

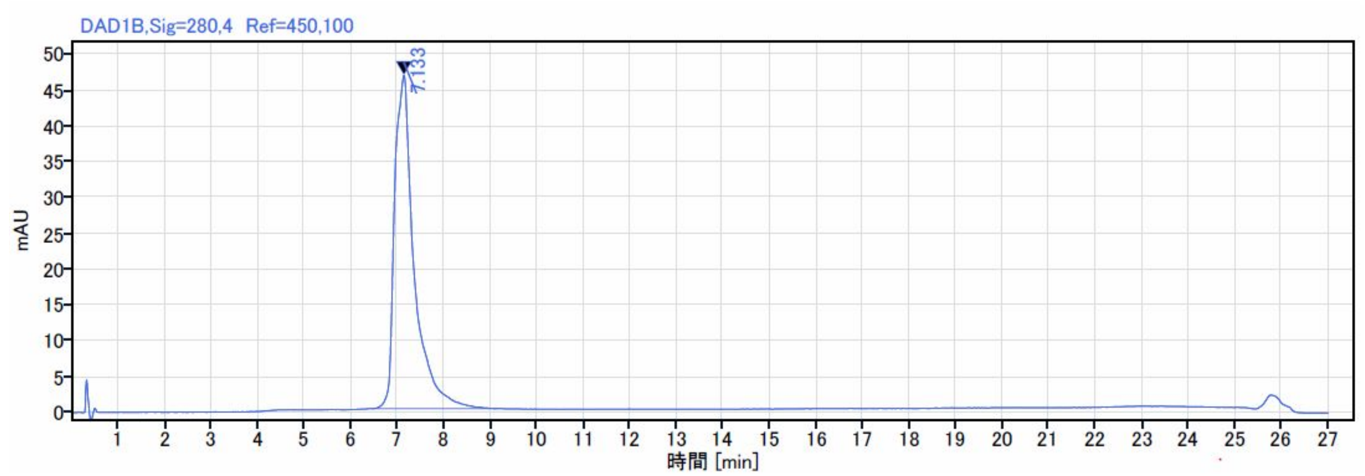

Figure S25. HIC analysis of AAPC (infliximab-Lys288 peptide) derived from AJICAP reagent **6b**

Average PAR = 2.0

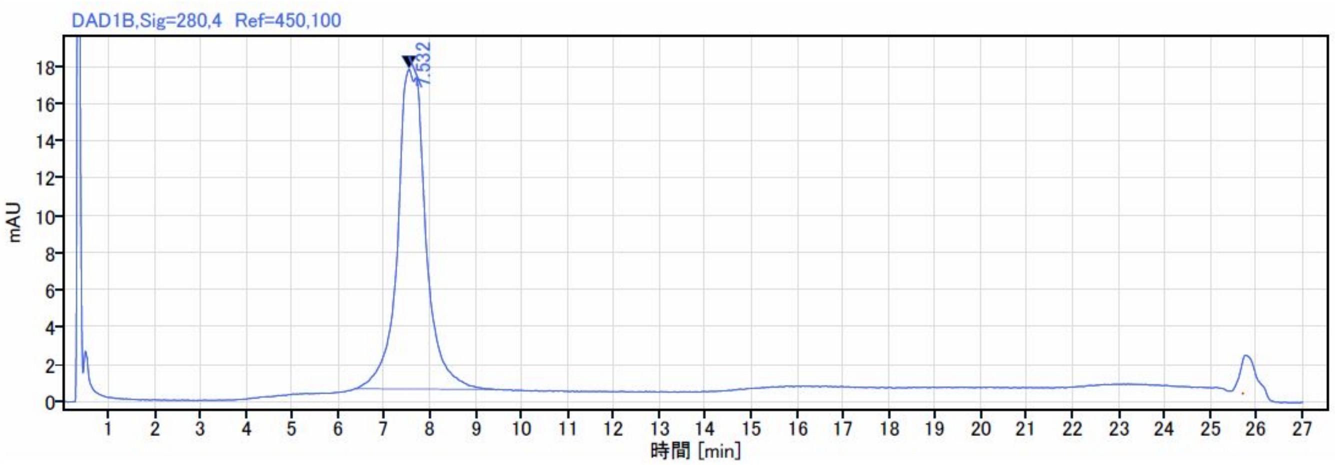

Figure S26. HIC analysis of infliximab-Lys248-thiol derived from AJICAP reagent **1b**

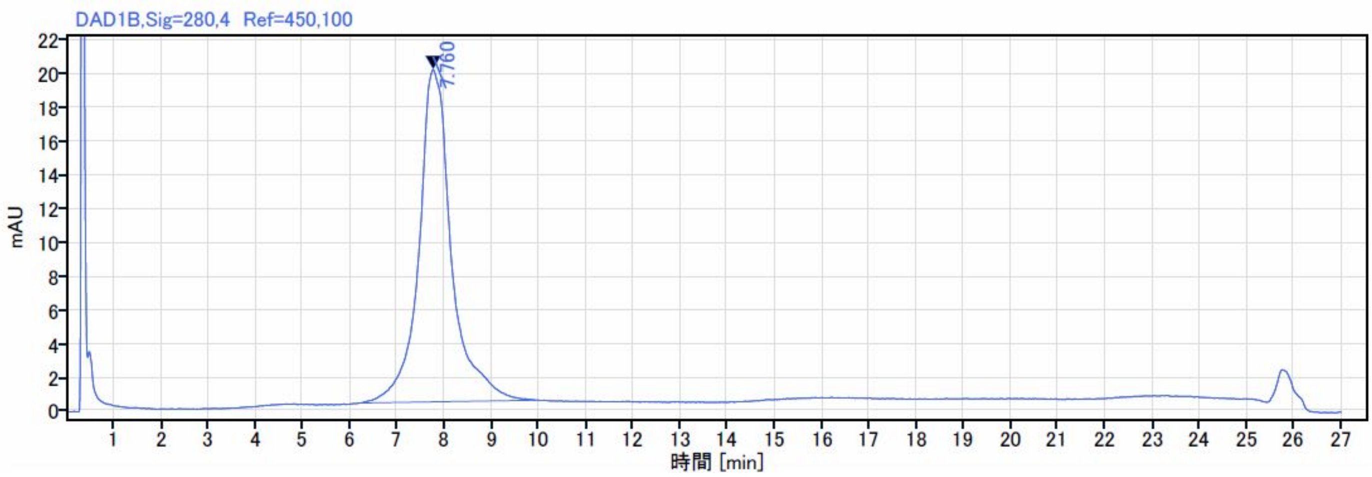

Figure S27. HIC analysis of infliximab-Lys288-thiol derived from AJICAP reagent **6b**

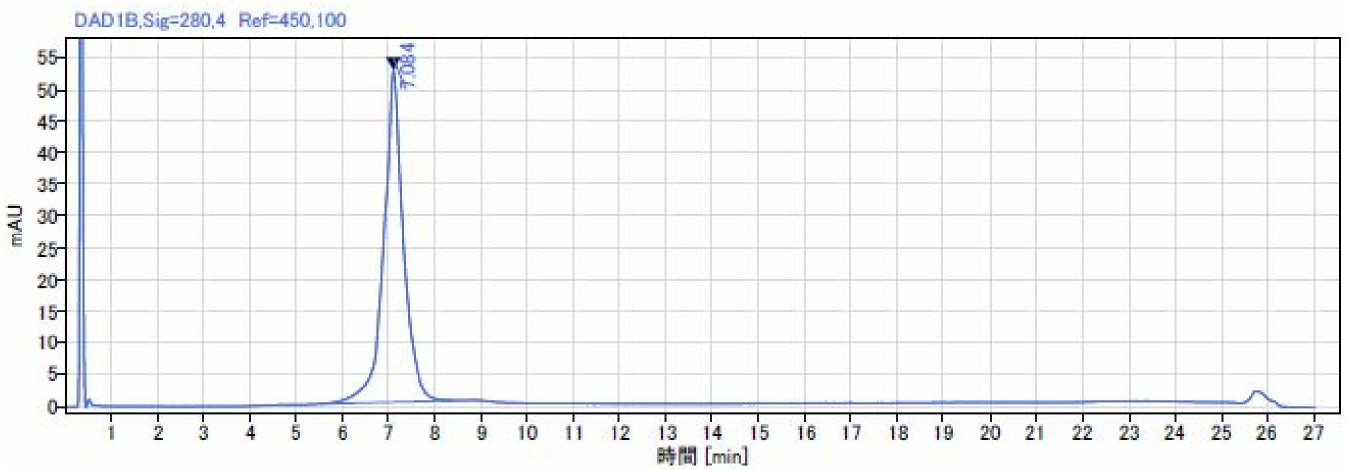

Figure S28. HIC analysis of Cetuximab

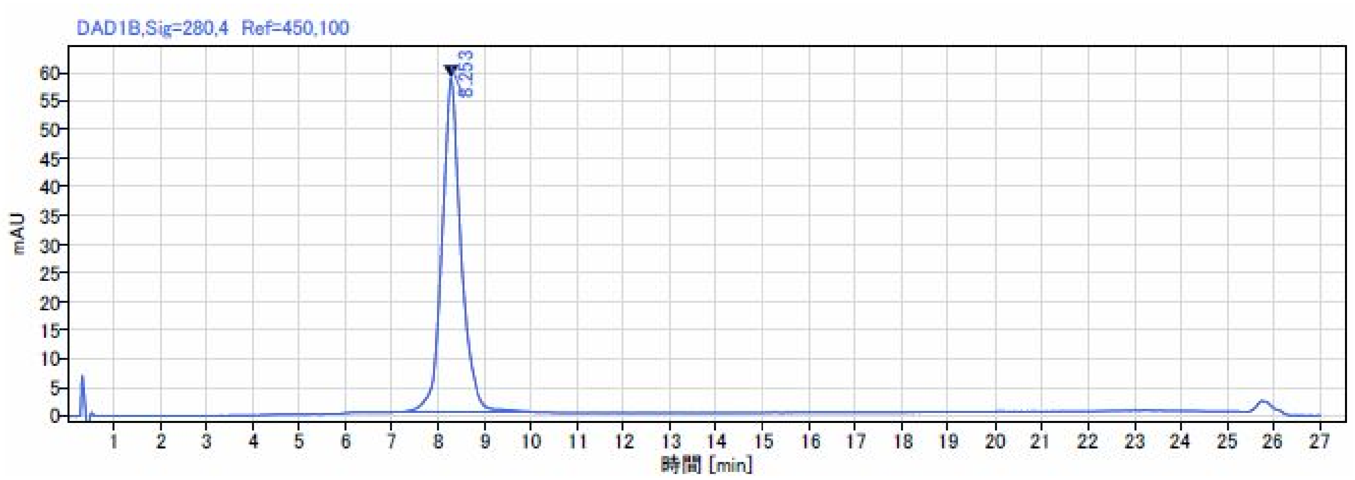

Figure S29. HIC analysis of AAPC (cetuximab-Lys248 peptide) derived from AJICAP reagent **1b**

Average PAR = 2.0

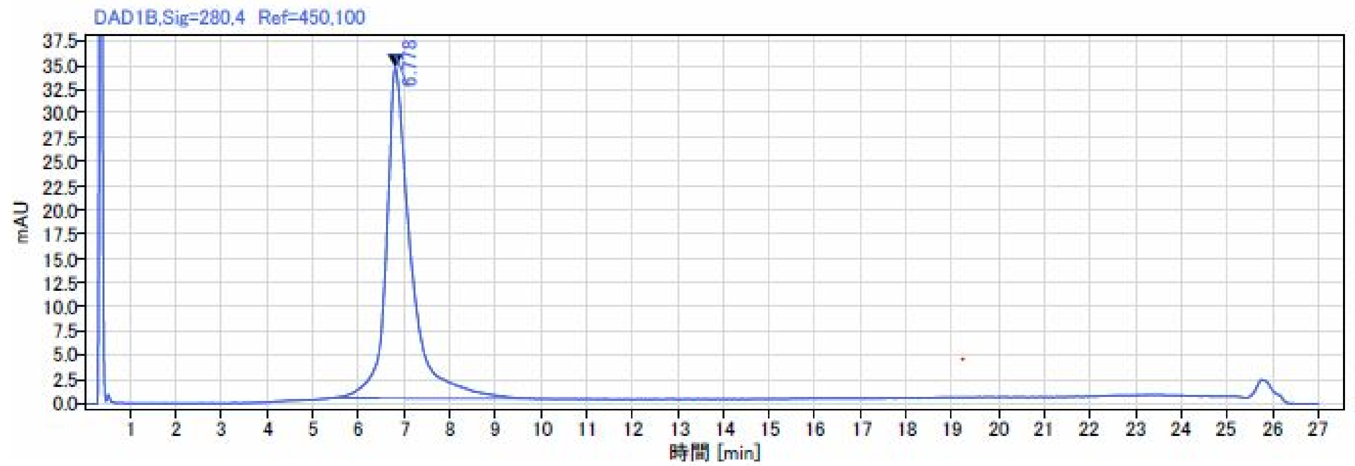

Figure S30. HIC analysis of AAPC (cetuximab-Lys288 peptide) derived from AJICAP reagent **6b**

Average PAR = 2.0

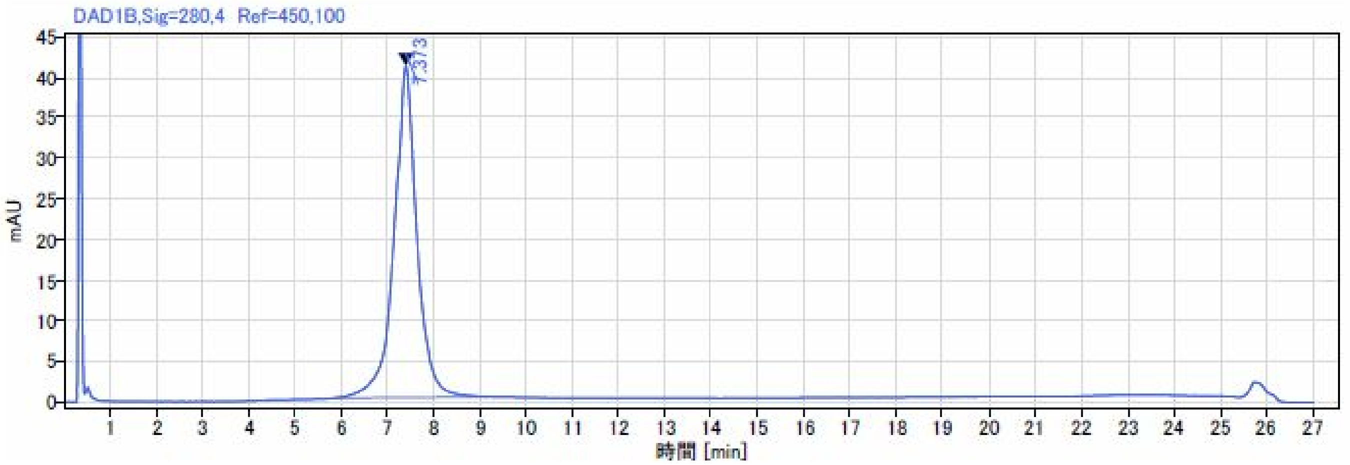

Figure S31. HIC analysis of cetuximab-Lys248-thiol derived from AJICAP reagent **1b**

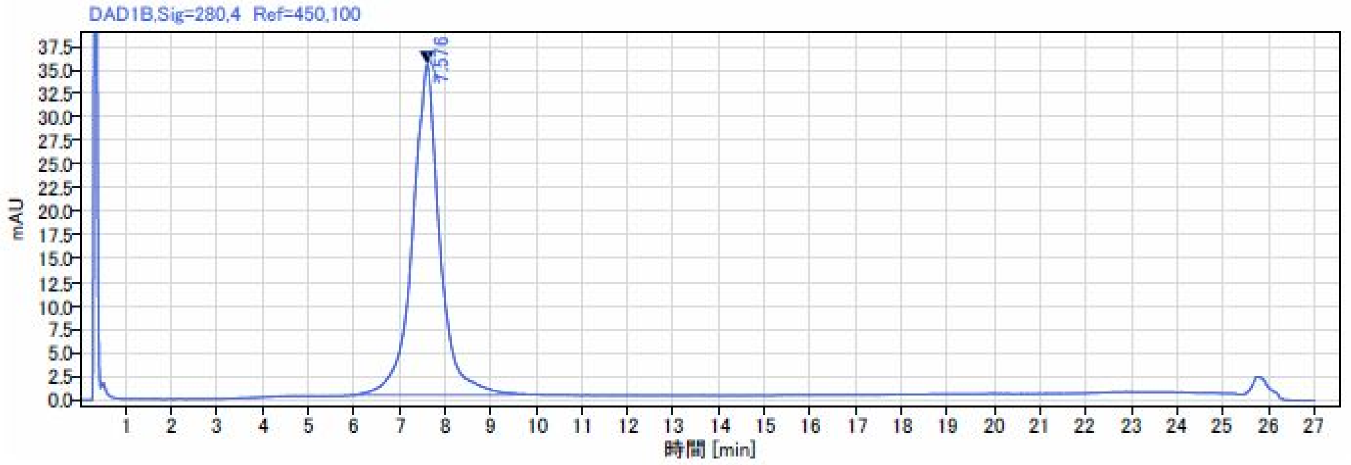

Figure S32. HIC analysis of cetuximab-Lys288-thiol derived from AJICAP reagent **6b**

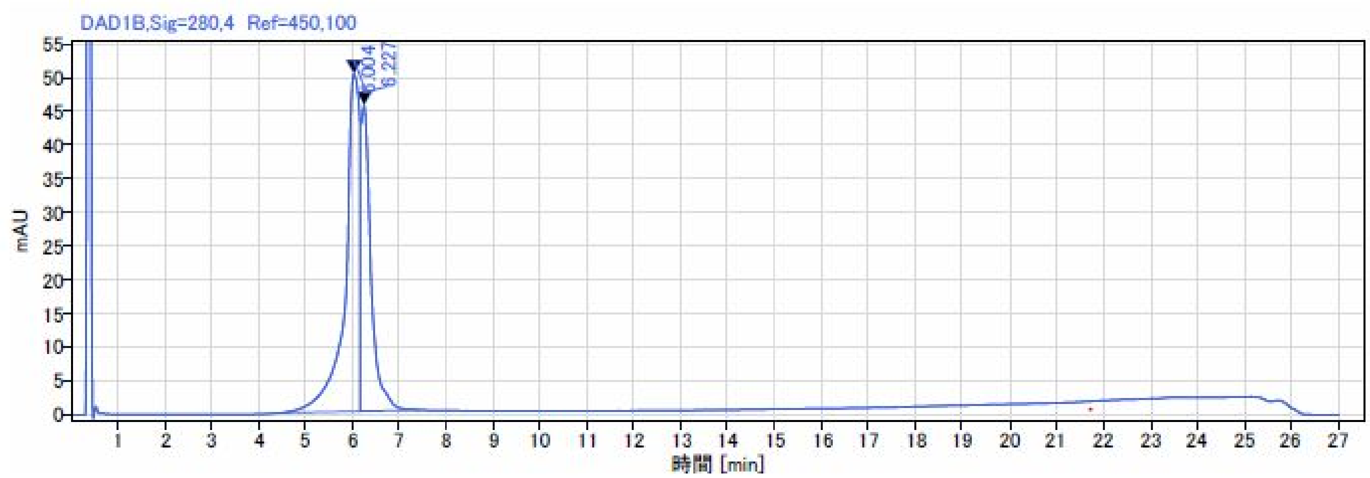

Figure S33. HIC analysis of denosumab

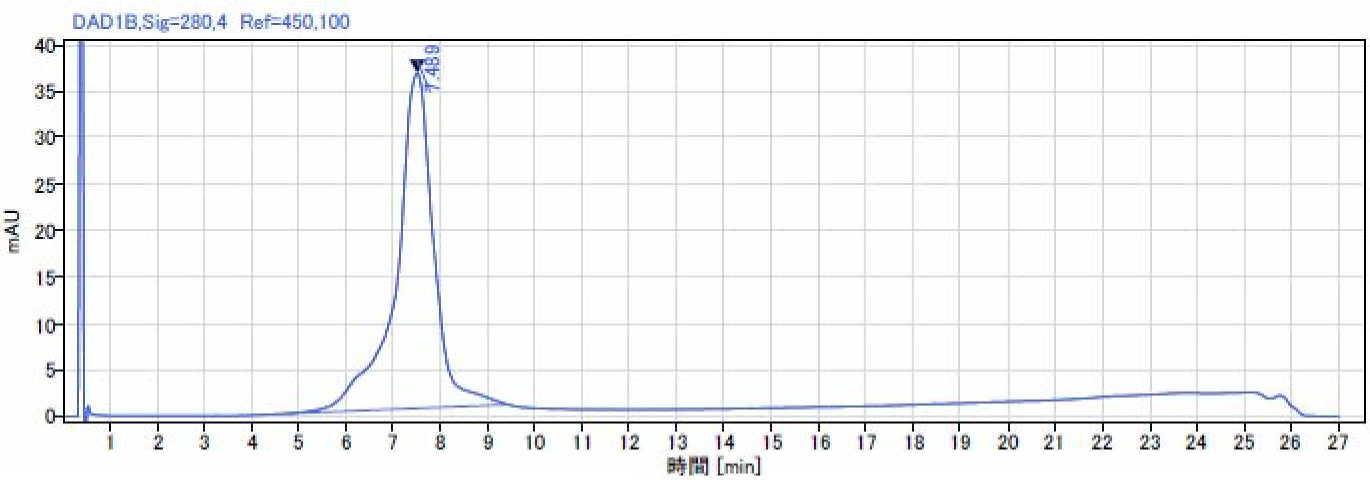

Figure S34. HIC analysis of AAPC (denosumab-Lys248 peptide) derived from AJICAP reagent **1b**

Average PAR = 2.0

=

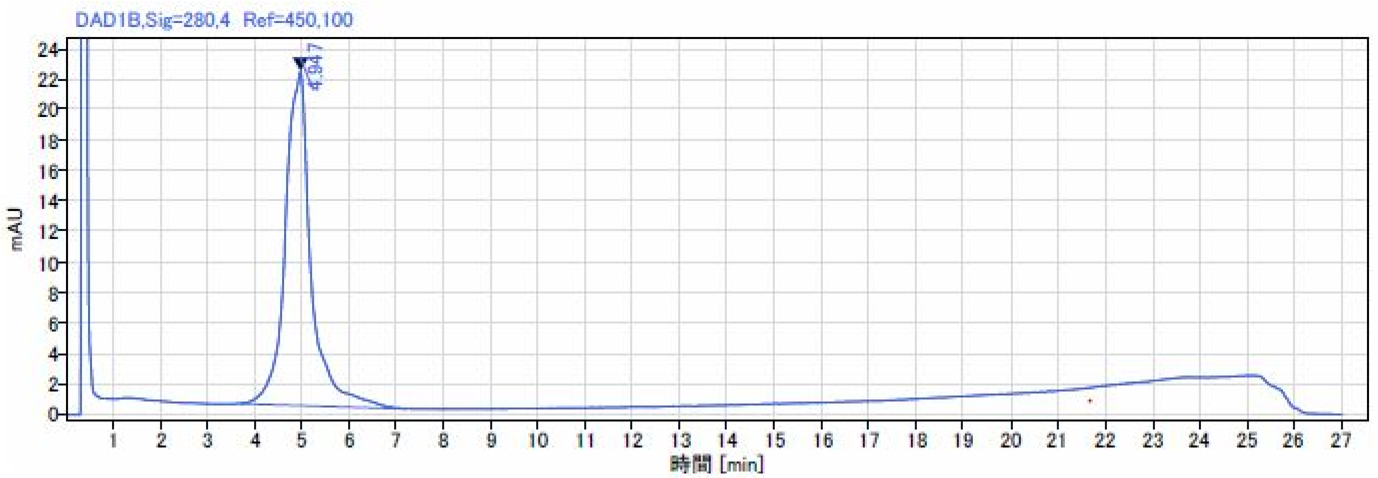

Figure S35. HIC analysis of AAPC (denosumab-Lys288 peptide) derived from AJICAP reagent **6b**

Average PAR = 2.0

=

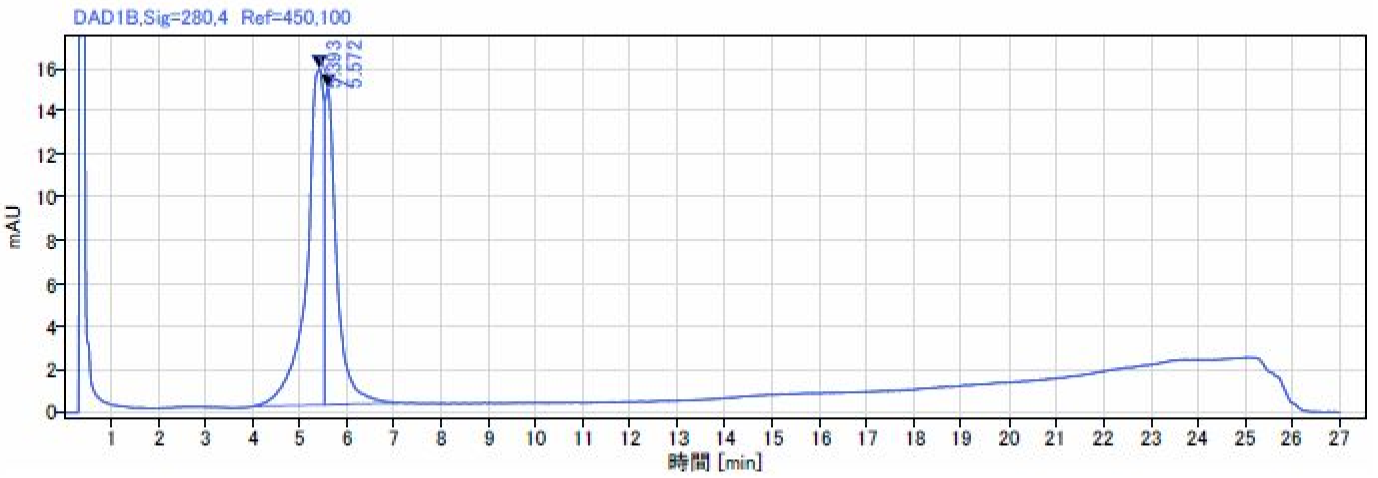

Figure S36. HIC analysis of denosumab-Lys248-thiol derived from AJICAP reagent **1b**

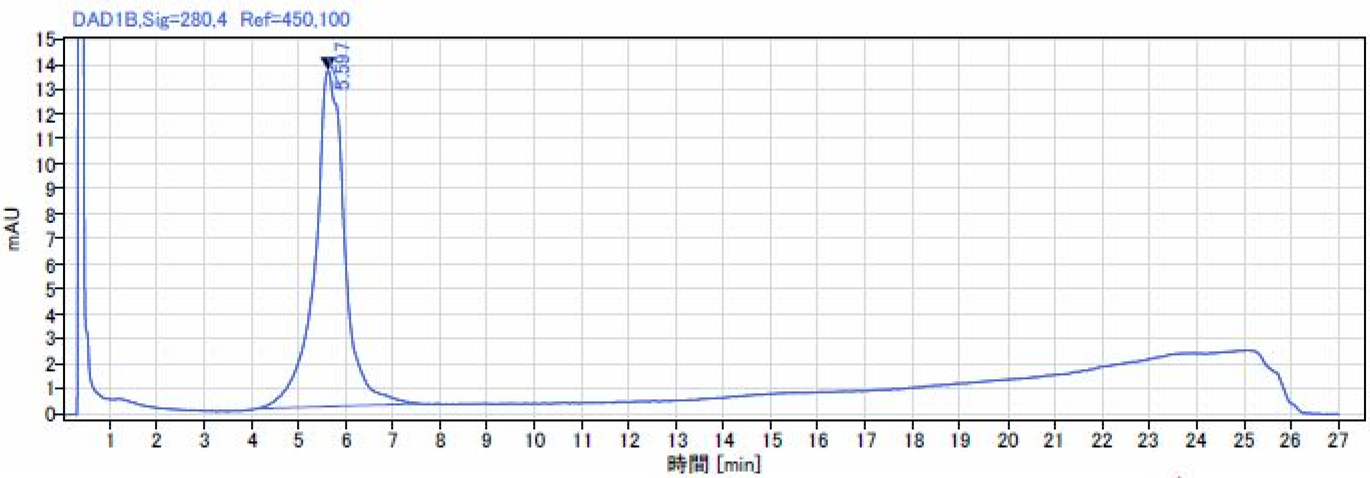

~~Figure S37. HIC analysis of denosumab-Lys288-thiol derived from AJICAP reagent **6b**~~

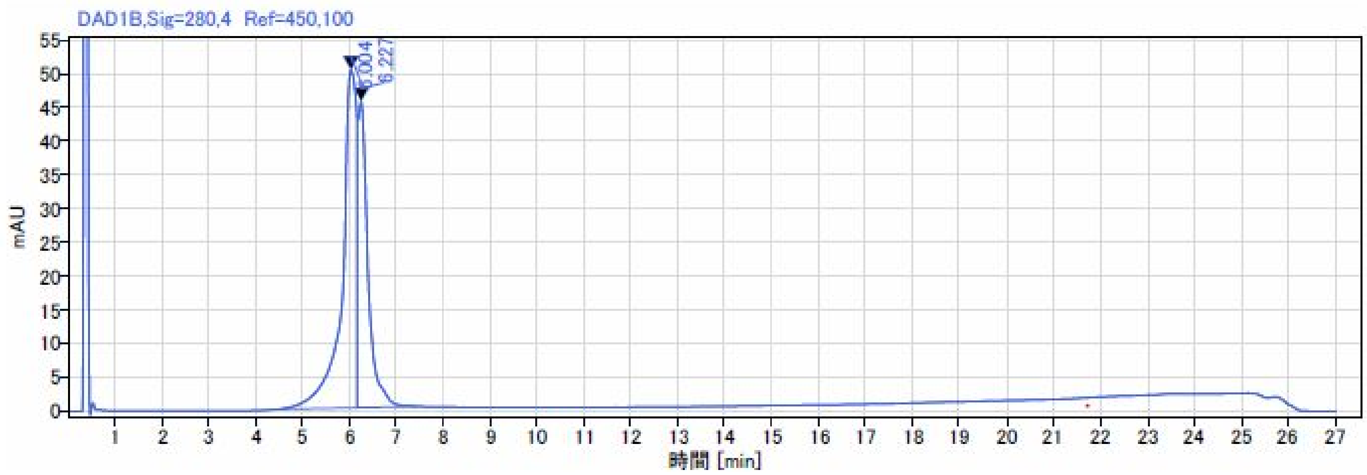

Figure S33. HIC analysis of denosumab

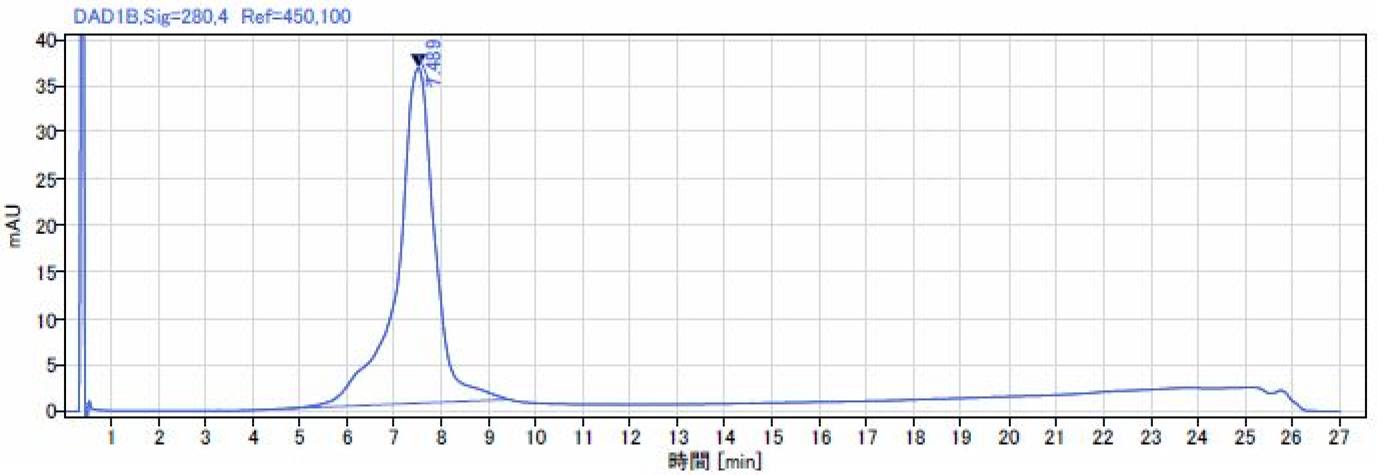

Figure S34. HIC analysis of AAPC (denosumab-Lys248 peptide) derived from AJICAP reagent **1b**

Average PAR = 2.0

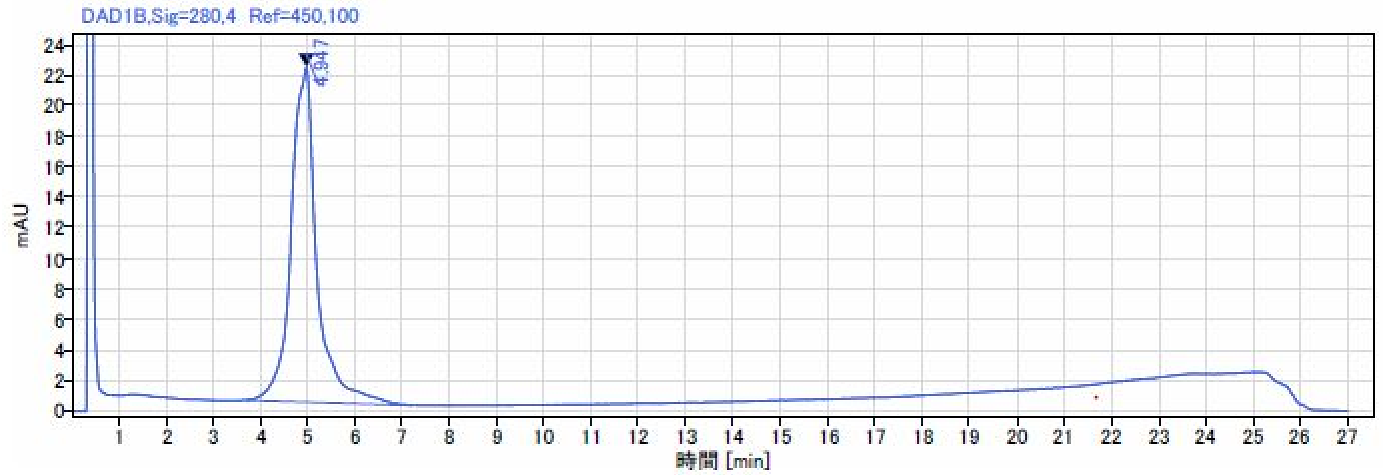

Figure S35. HIC analysis of AAPC (denosumab-Lys288 peptide) derived from AJICAP reagent **6b**

Average PAR = 2.0

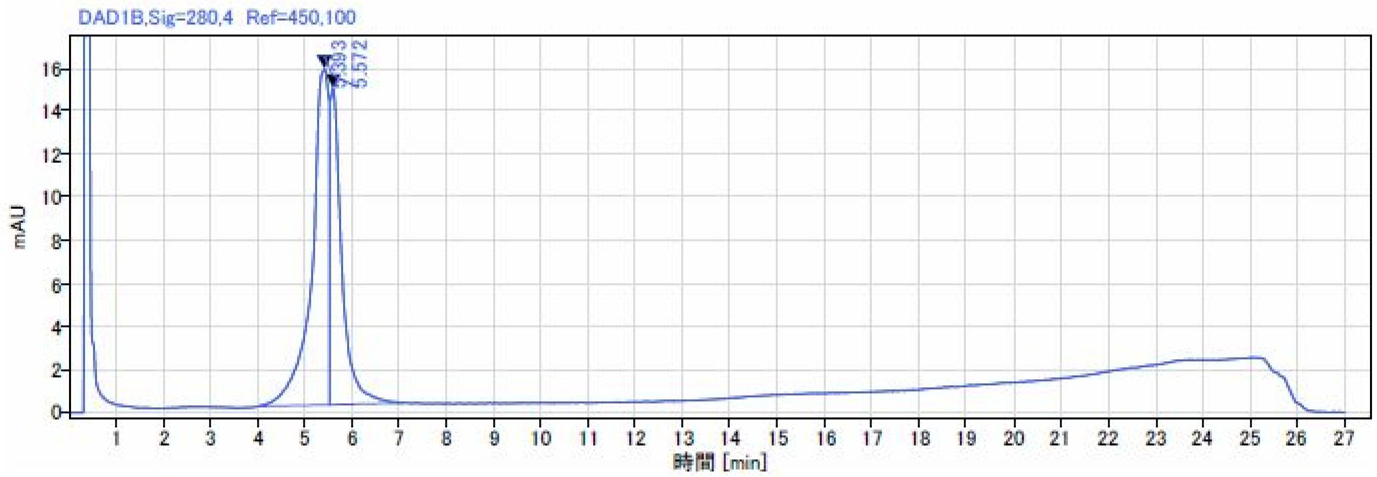

Figure S36. HIC analysis of denosumab-Lys248-thiol derived from AJICAP reagent **1b**

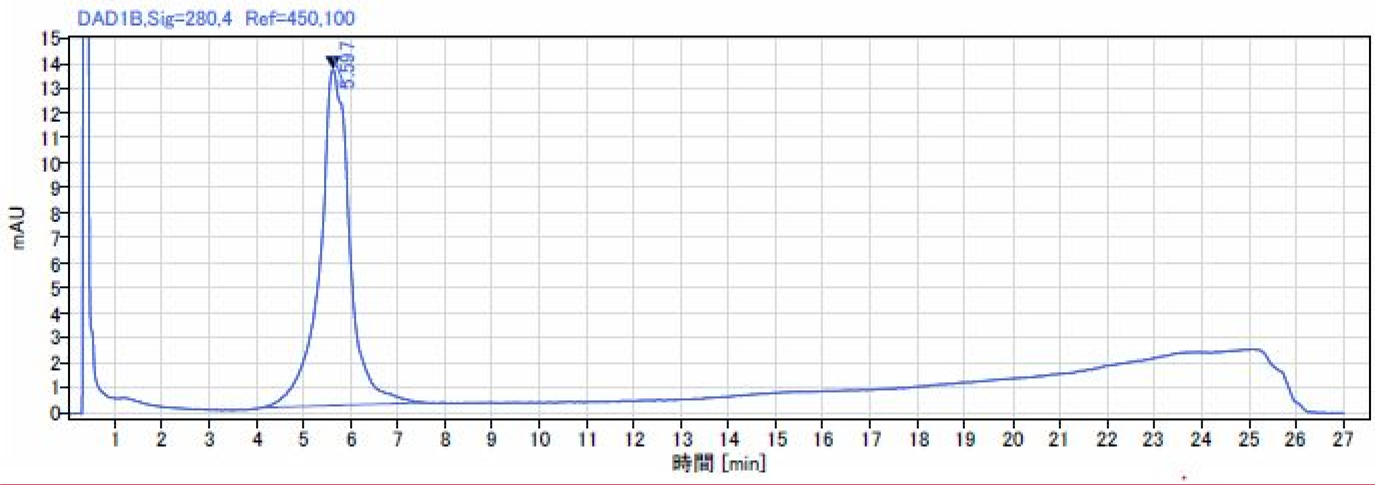

Figure S37. HIC analysis of denosumab-Lys288-thiol derived from AJICAP reagent **6b**

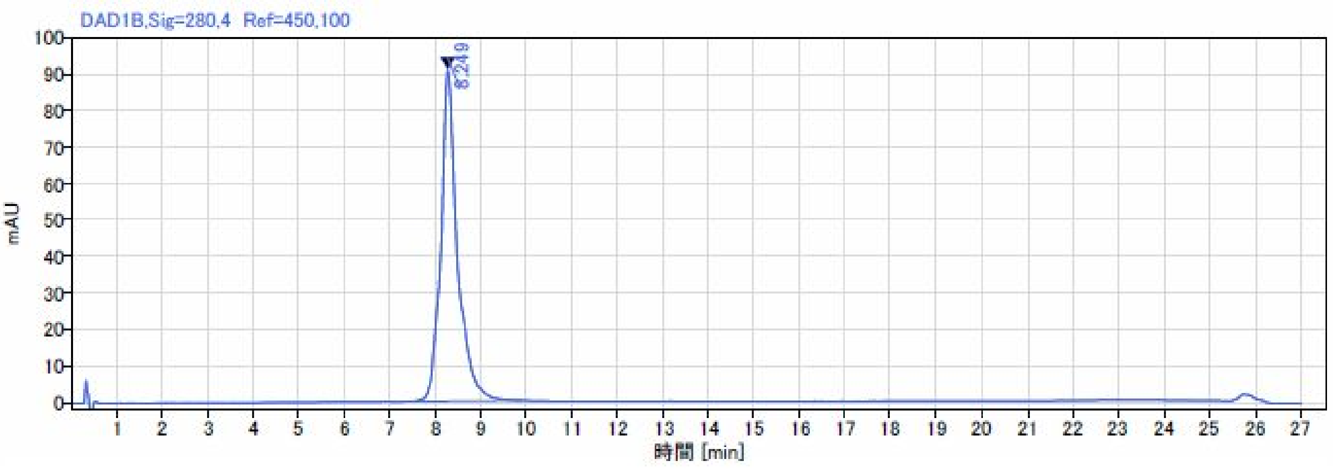

Figure S38. HIC analysis of pembrolizumab

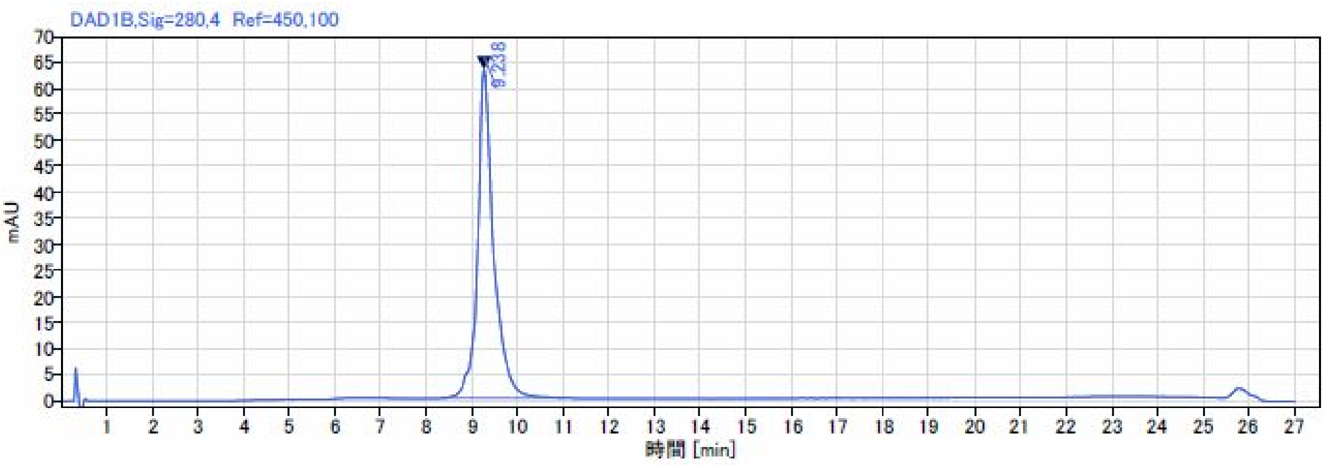

Figure S39. HIC analysis of AAPC (pembrolizumab-Lys248 peptide) derived from AJICAP reagent **1b**

Average PAR = 2.0

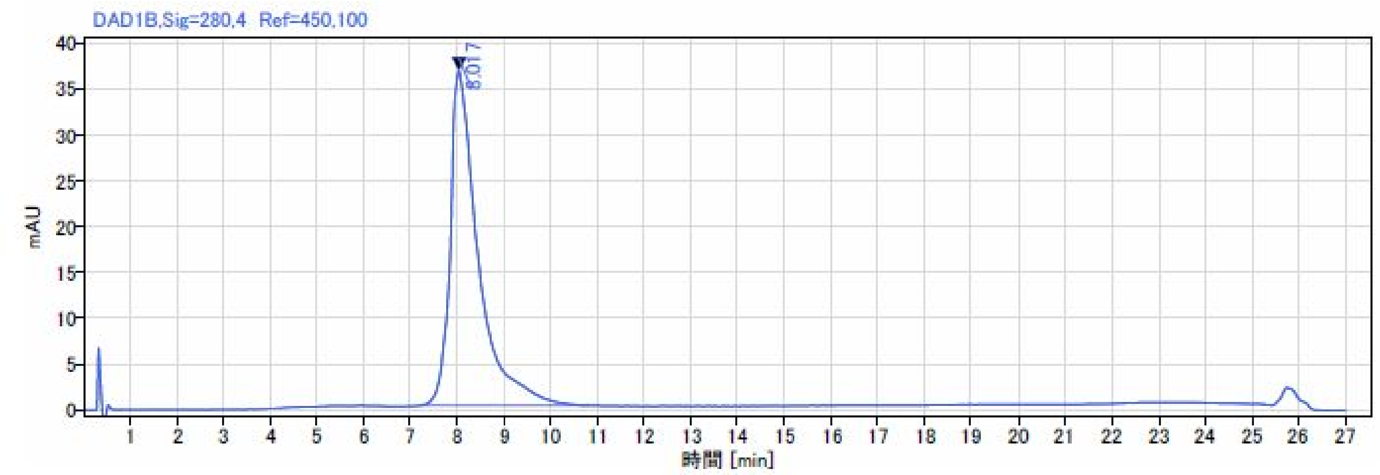

Figure S40. HIC analysis of AAPC (pembrolizumab-Lys288 peptide) derived from AJICAP reagent **6b**

Average PAR = 2.0

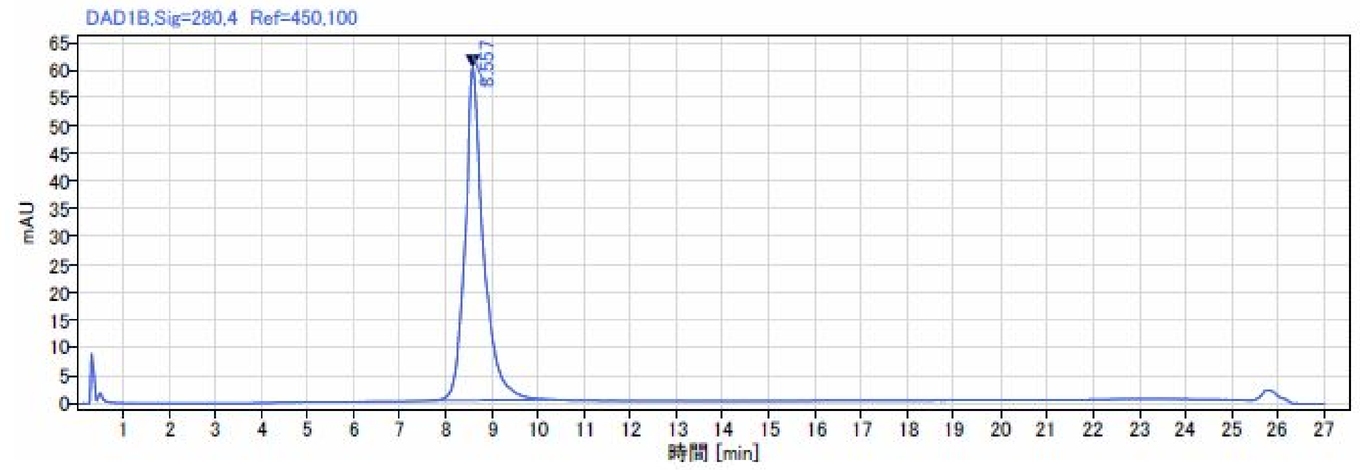

Figure S41. HIC analysis of pembrolizumab-Lys248-thiol derived from AJICAP reagent **1b**

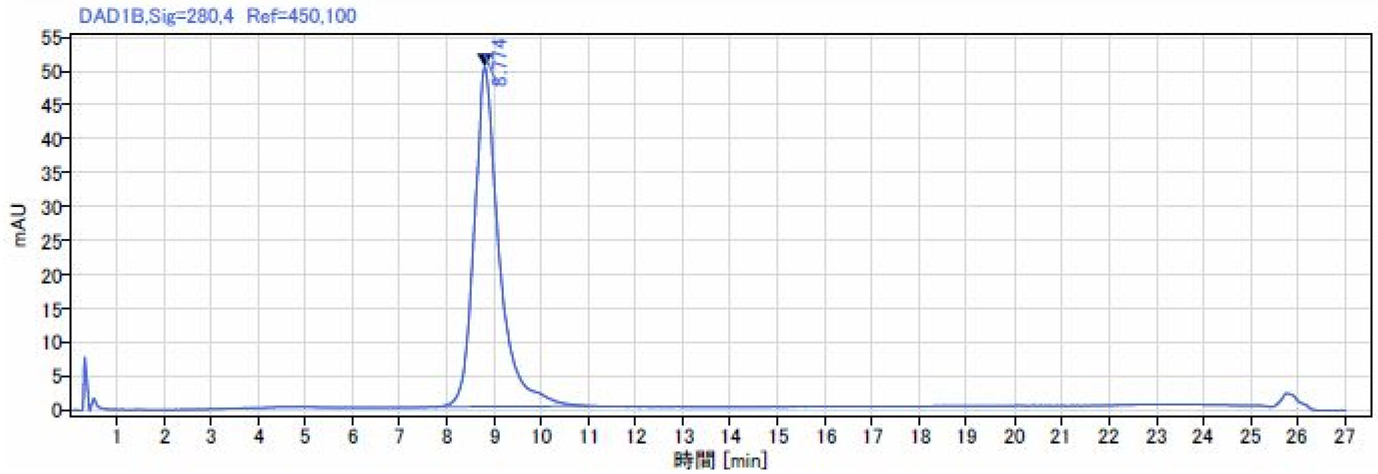

Figure S42. HIC analysis of pembrolizumab-Lys288-thiol derived from AJICAP reagent **6b**

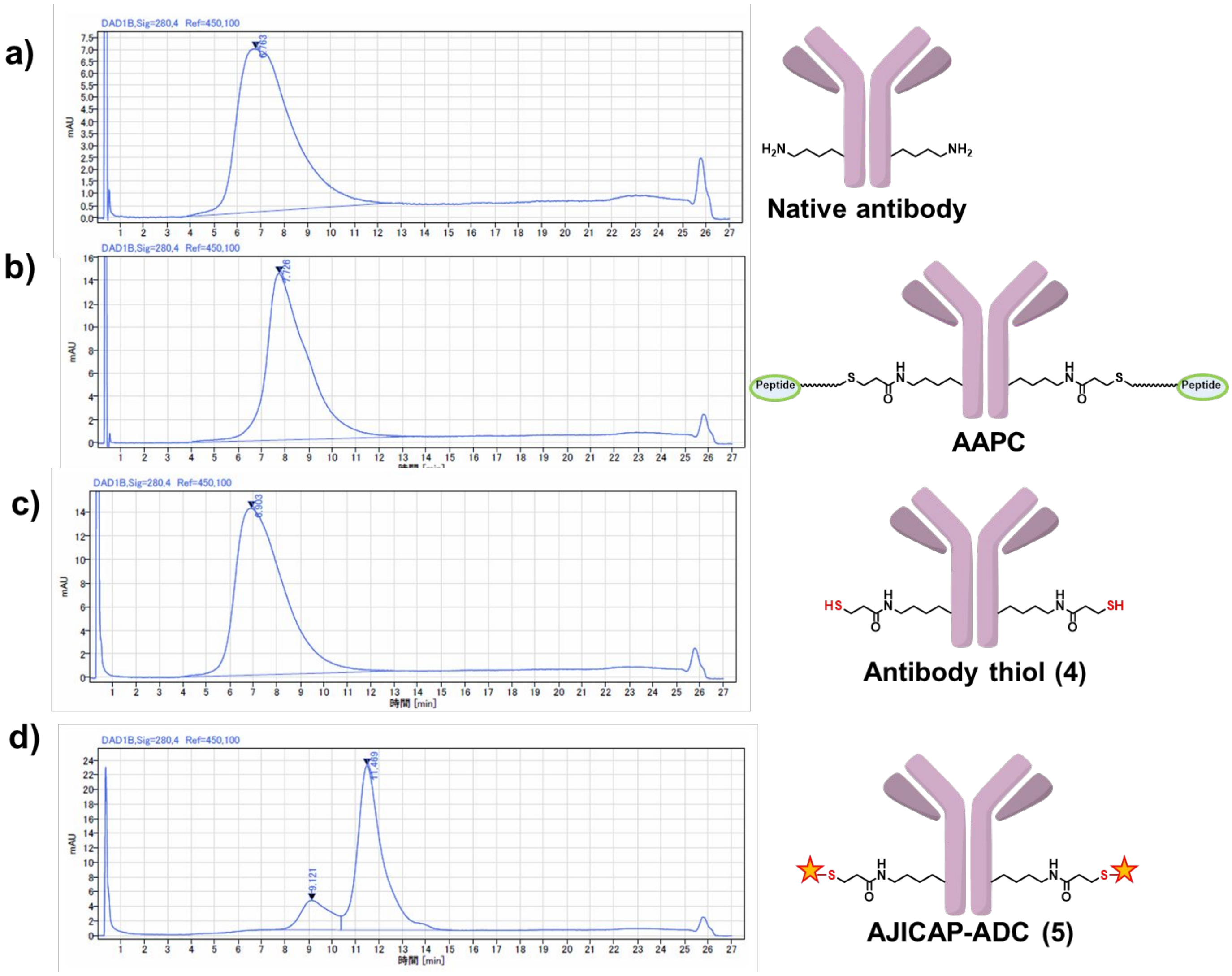

Figure S43. HIC analysis of polyclonal mAb-Lys248-MMAE derived from AJICAP reagent **1b**; a) naked antibody, b) AAPC, c) antibody-thiol, d) antibody-Lys248-MMAE (DAR=1.8)

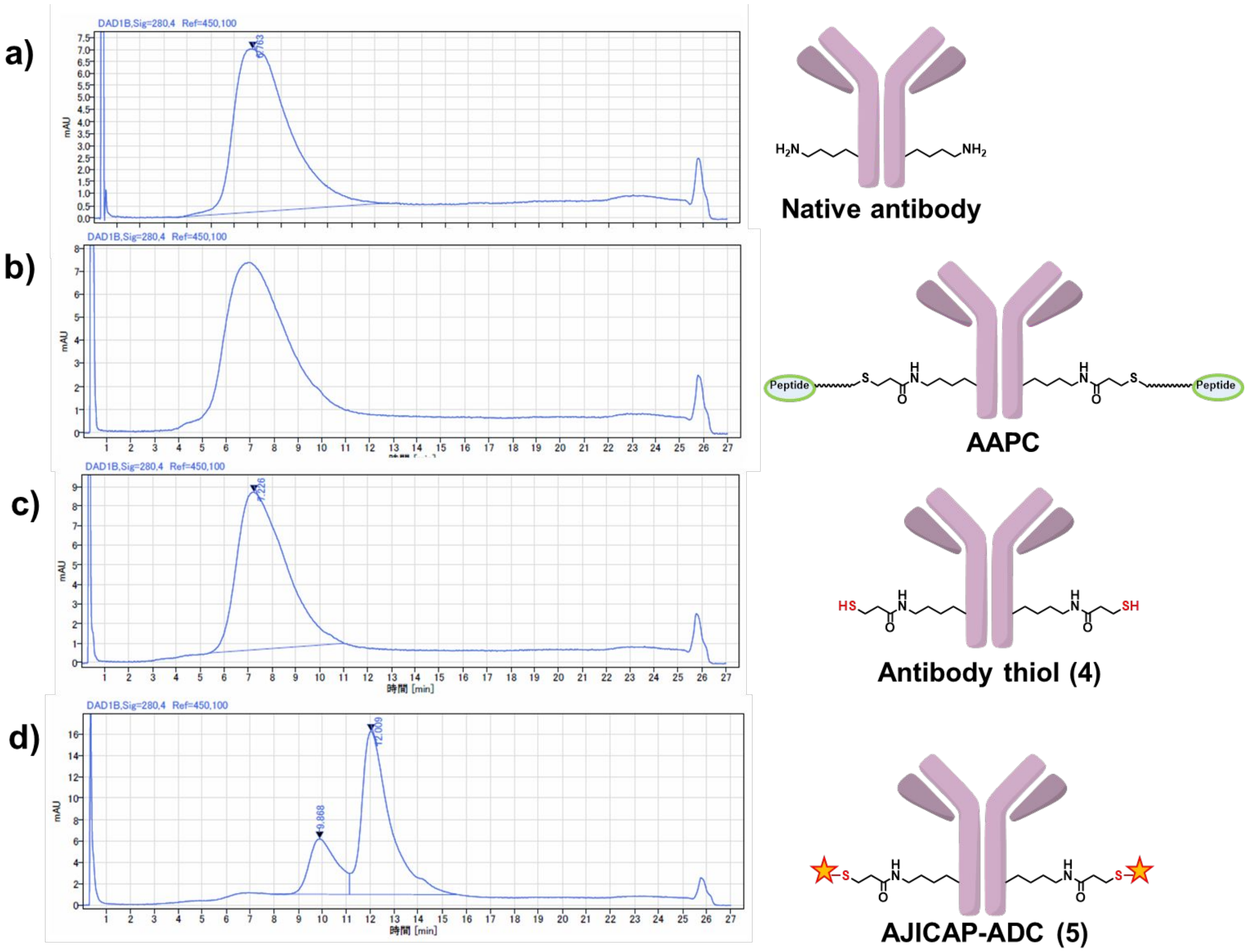

Figure S44. HIC analysis of polyclonal mAb-Lys288-MMAE derived from AJICAP reagent **6b**; a) naked antibody, b) AAPC, c) antibody-thiol, d) antibody-Lys288-MMAE (DAR=1.8)

2-4-2 Q-TOF MS analysis

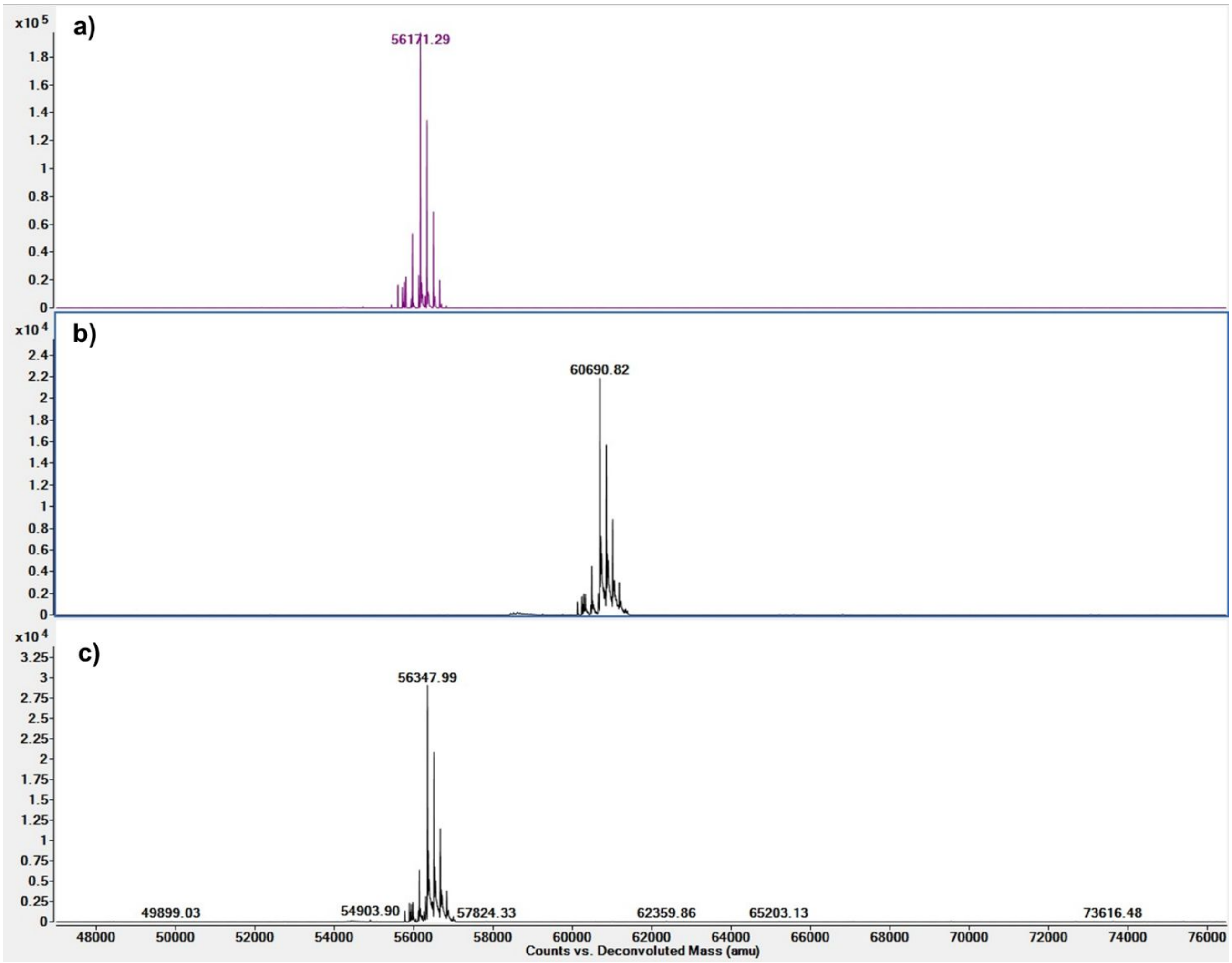

Figure S45. Q-TOF MS analysis of Fc Protein-Lys248-MMAE derived from AJICAP reagent **1b**; a) naked Fc protein, b) AAPC (PAR=2.0), c) antibody-thiol

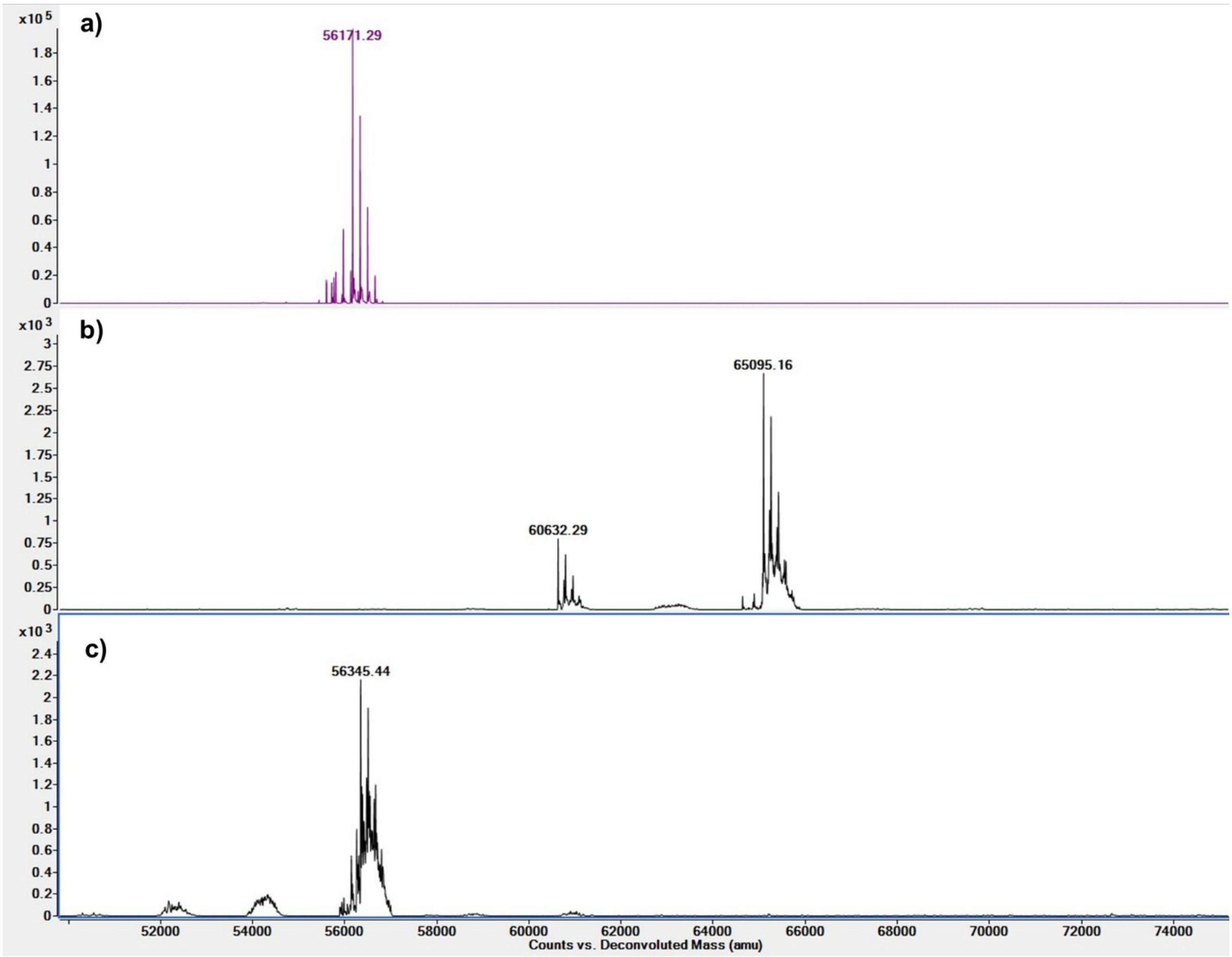

Figure S46. Q-TOF MS analysis of Fc Protein-Lys288-MMAE derived from AJICAP reagent **1b**; a) naked Fc-protein, b) AAPC (PAR=1.8), c) antibody-thiol

2-4-3 SEC-HPLC analysis

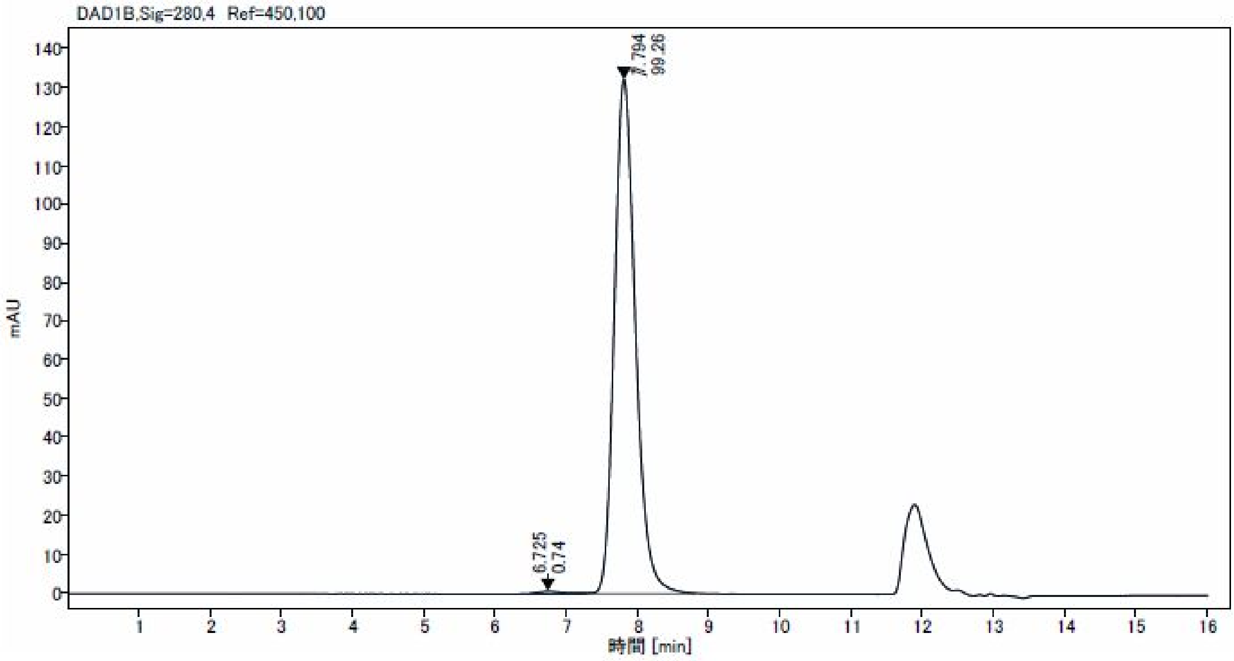

Figure S47. SEC analysis of trastuzumab-Lys248-thiol derived from AJICAP reagent **1b**

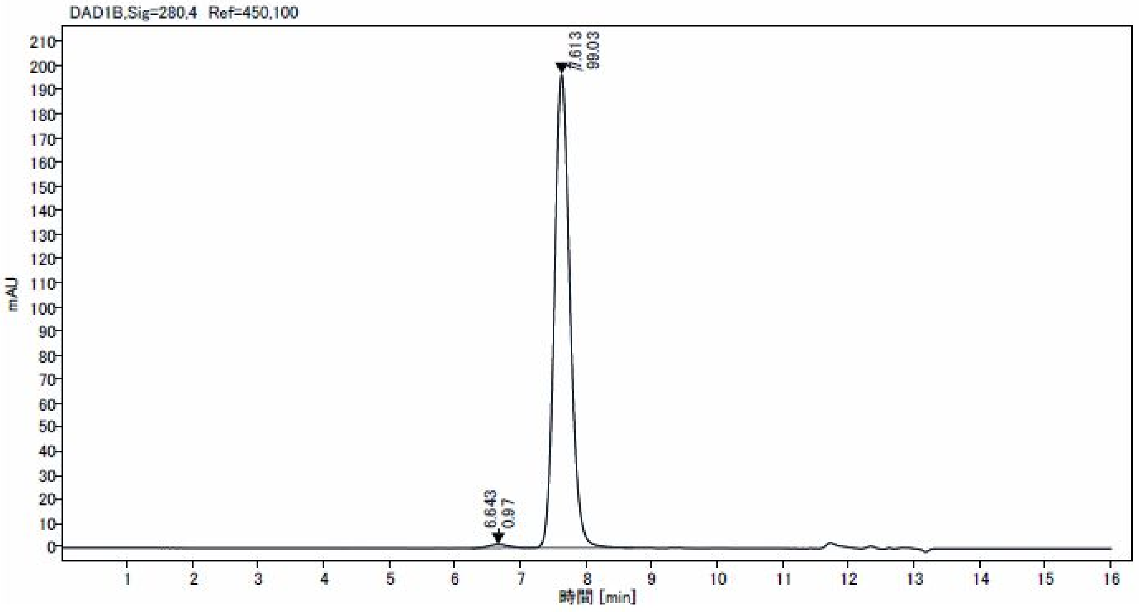

Figure S48. SEC analysis of trastuzumab-Lys288-thiol derived from AJICAP reagent **6b**

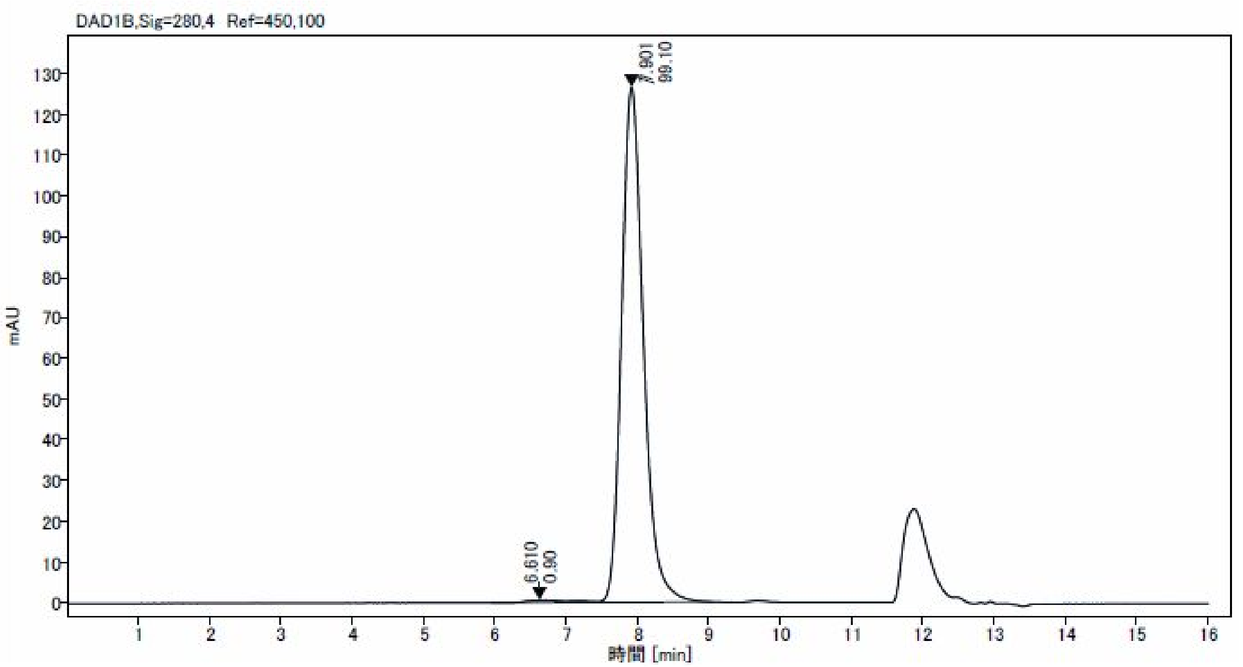

Figure S49. SEC analysis of rituximab-Lys248-thiol derived from AJICAP reagent **1b**

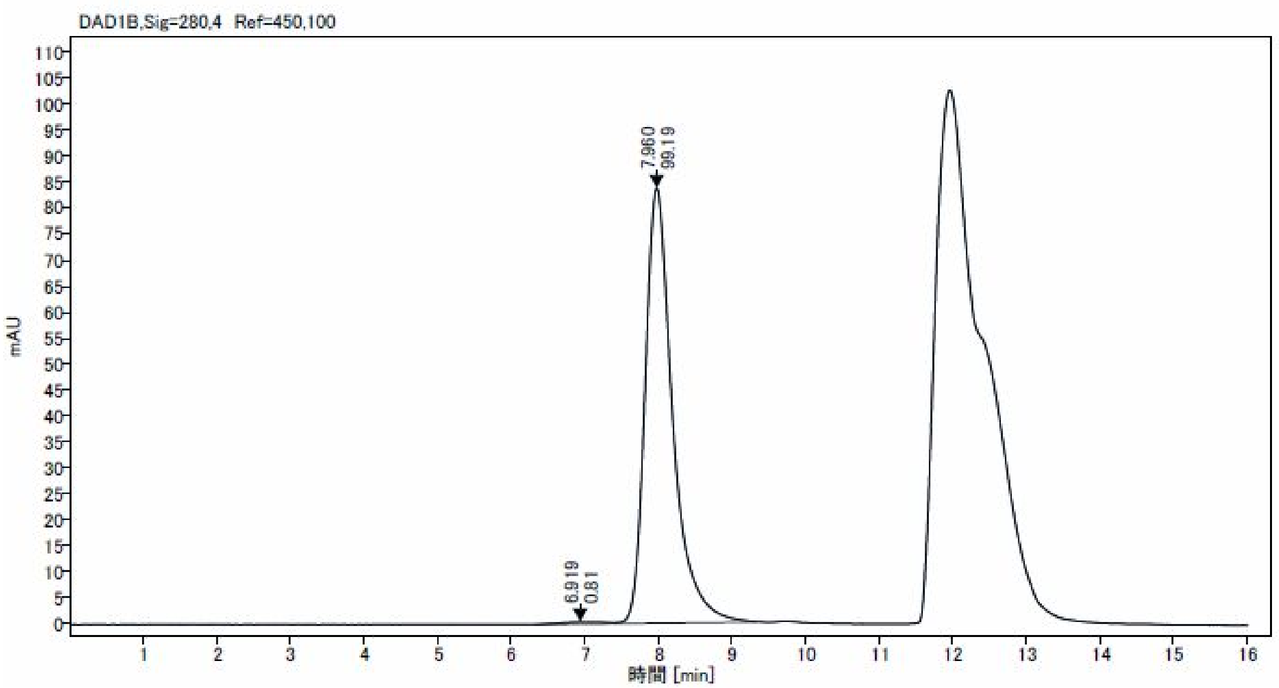

Figure S50. SEC analysis of rituximab-Lys288-thiol derived from AJICAP reagent **6b**

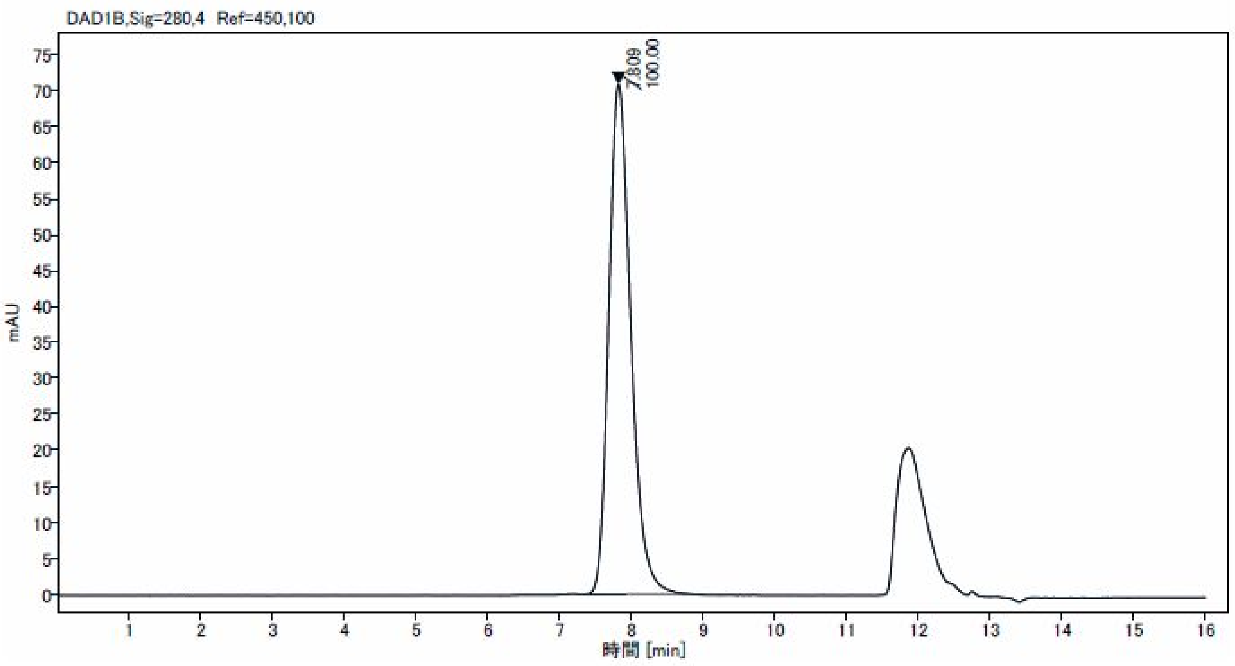

Figure S51. SEC analysis of infliximab-Lys248-thiol derived from AJICAP reagent **1b**

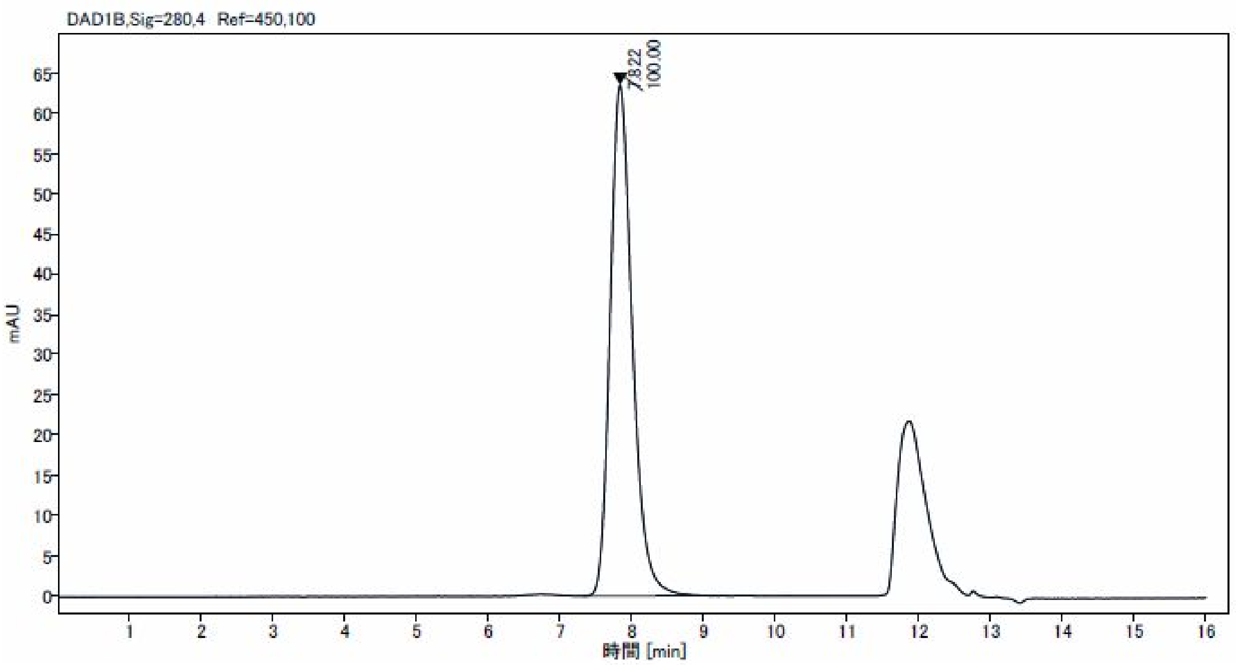

Figure S52. SEC analysis of infliximab-Lys288-thiol derived from AJICAP reagent **6b**

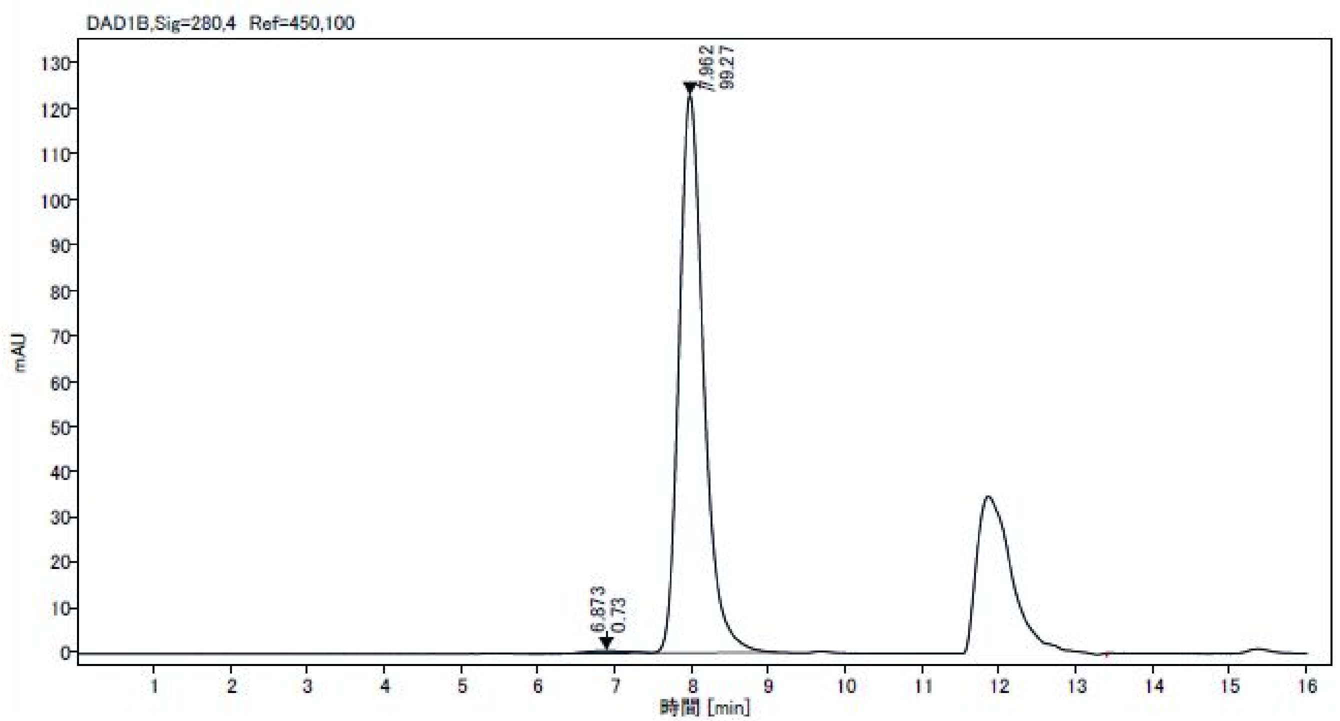

Figure S53. SEC analysis of cetuximab-Lys248-thiol derived from AJICAP reagent **1b**

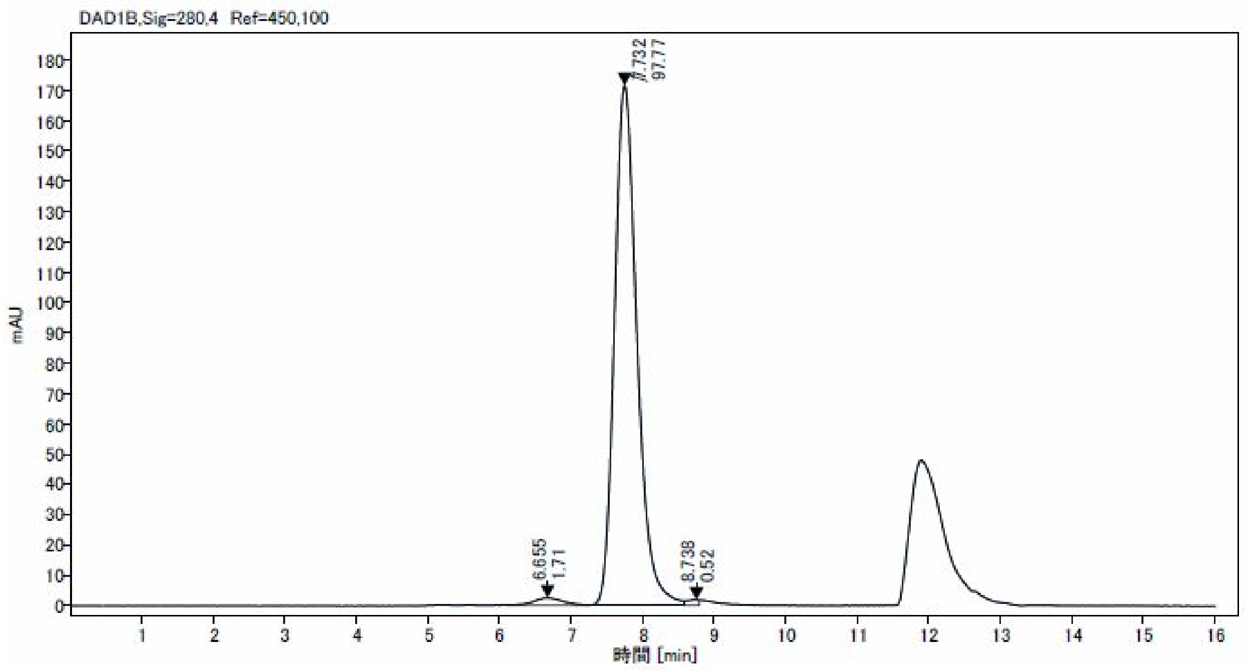

Figure S54. SEC analysis of cetuximab-Lys288-thiol derived from AJICAP reagent **6b**

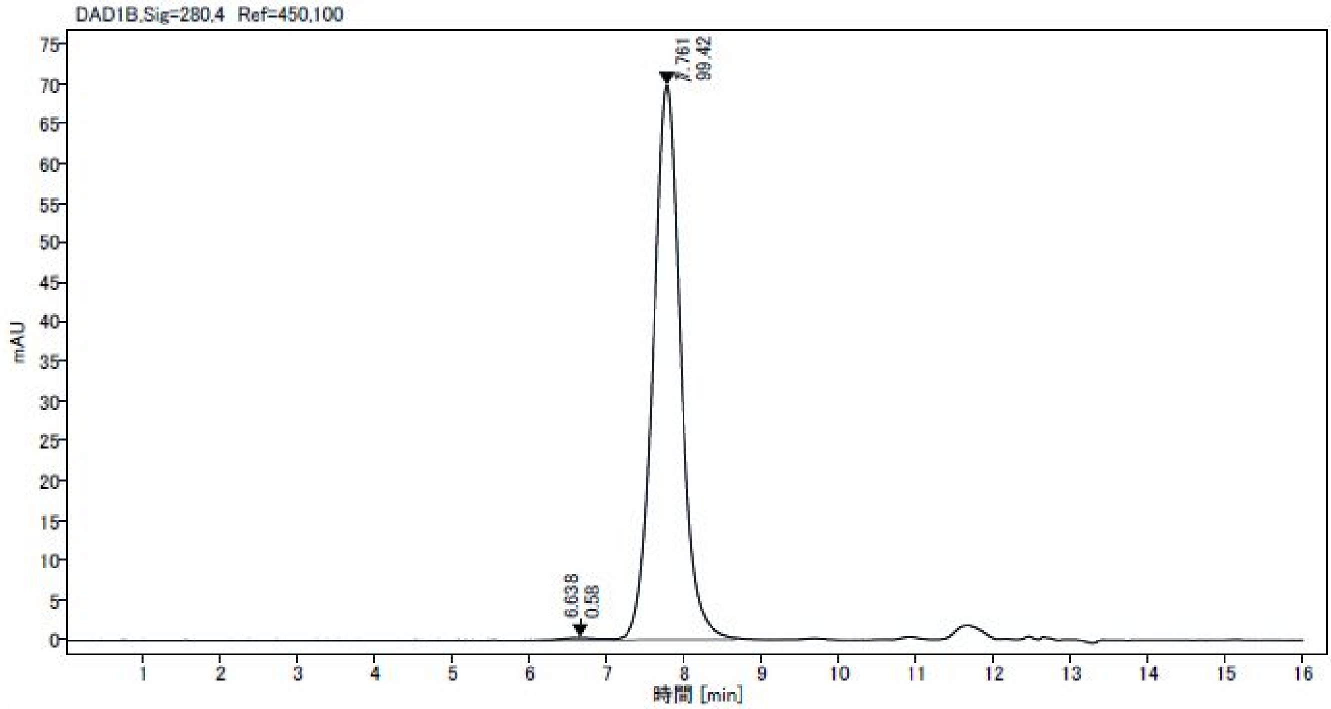

Figure S55. SEC analysis of denosumab-Lys248-thiol derived from AJICAP reagent **1b**

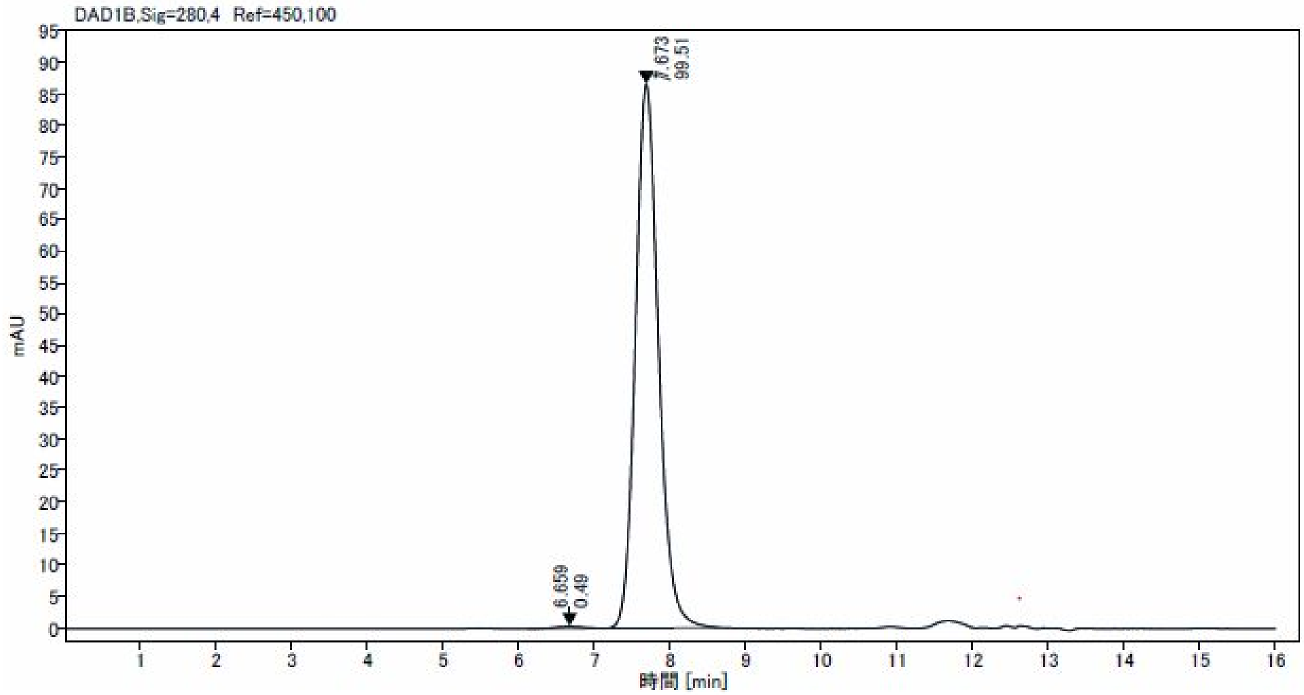

~~Figure S56. SEC analysis of denosumab-Lys288-thiol derived from AJICAP reagent **6b**~~

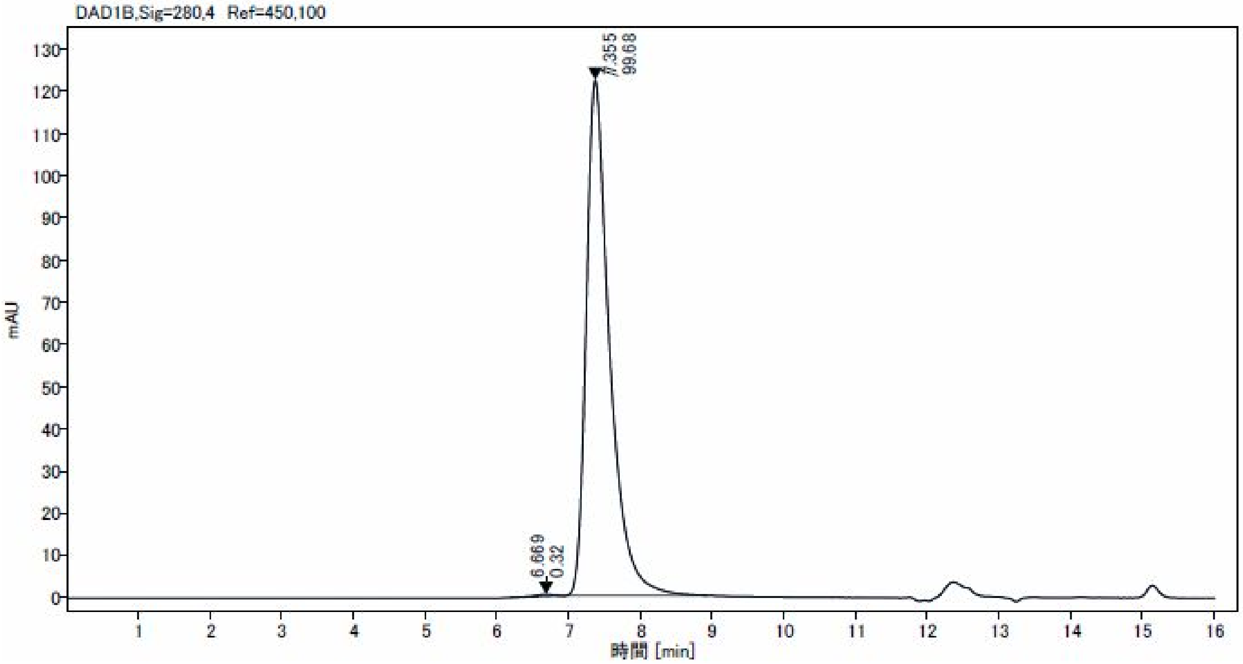

Figure S57. SEC analysis of pembrolizumab-Lys248-thiol derived from AJICAP reagent **1b**

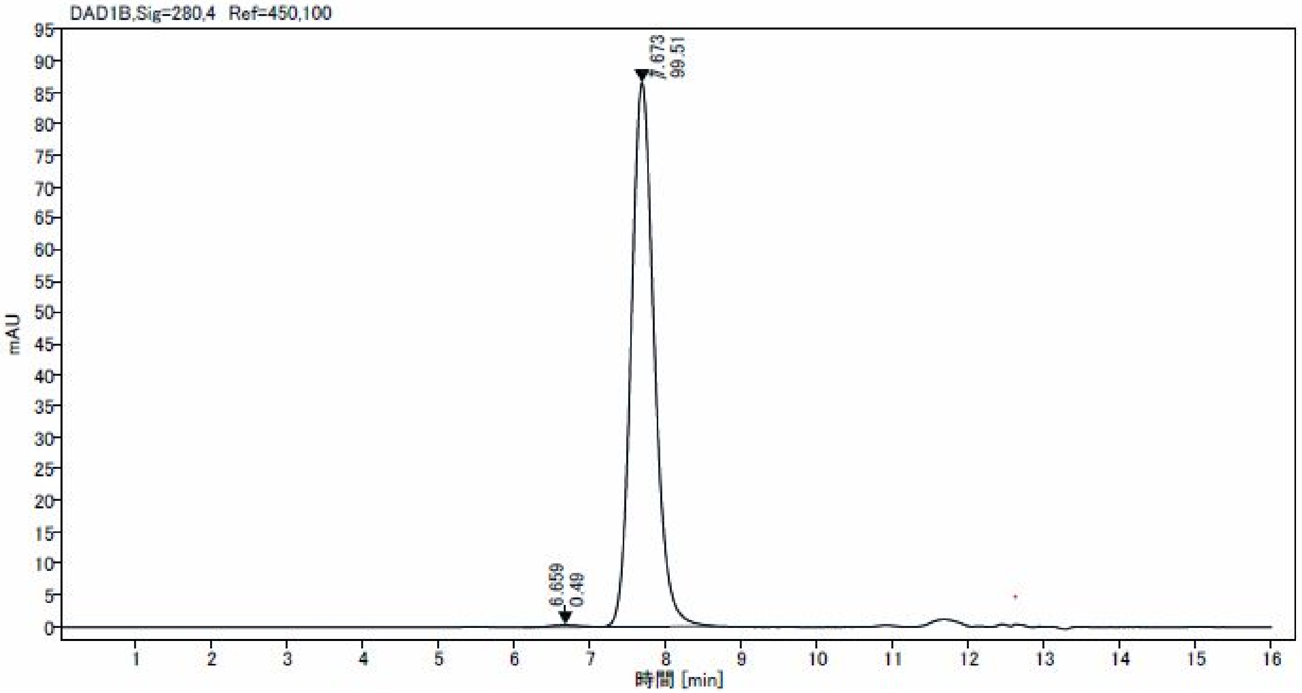

Figure S56. SEC analysis of denosumab-Lys288-thiol derived from AJICAP reagent **6b**

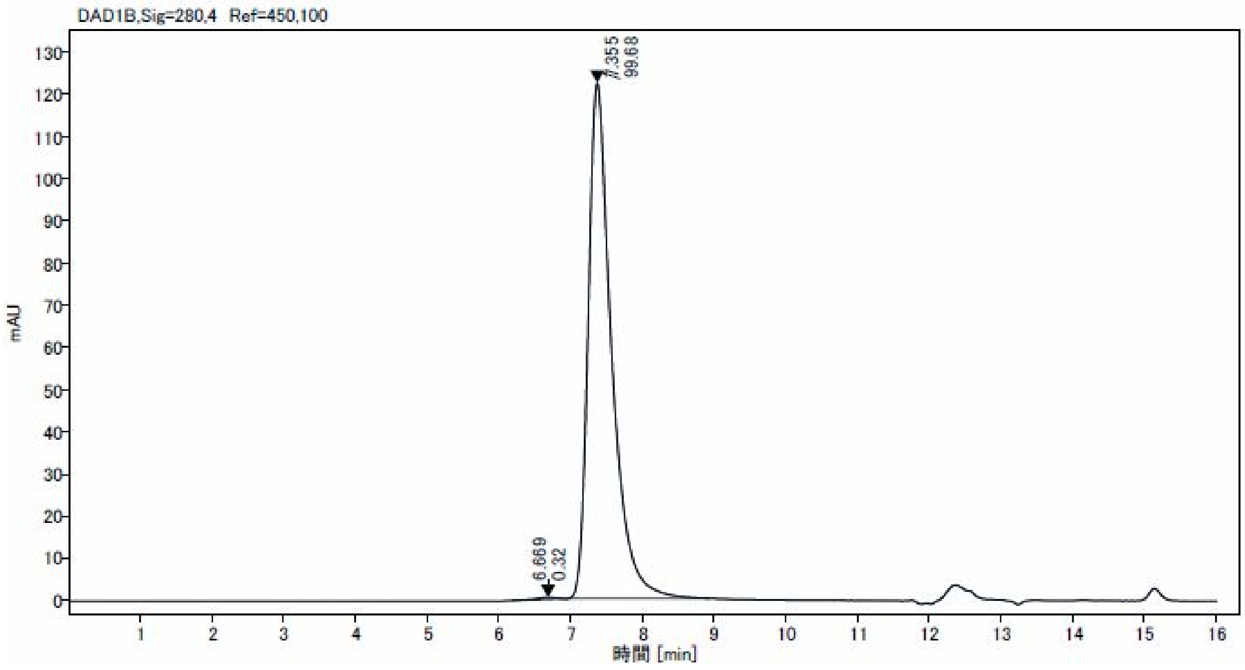

Figure S57. SEC analysis of pembrolizumab-Lys248-thiol derived from AJICAP reagent **1b**

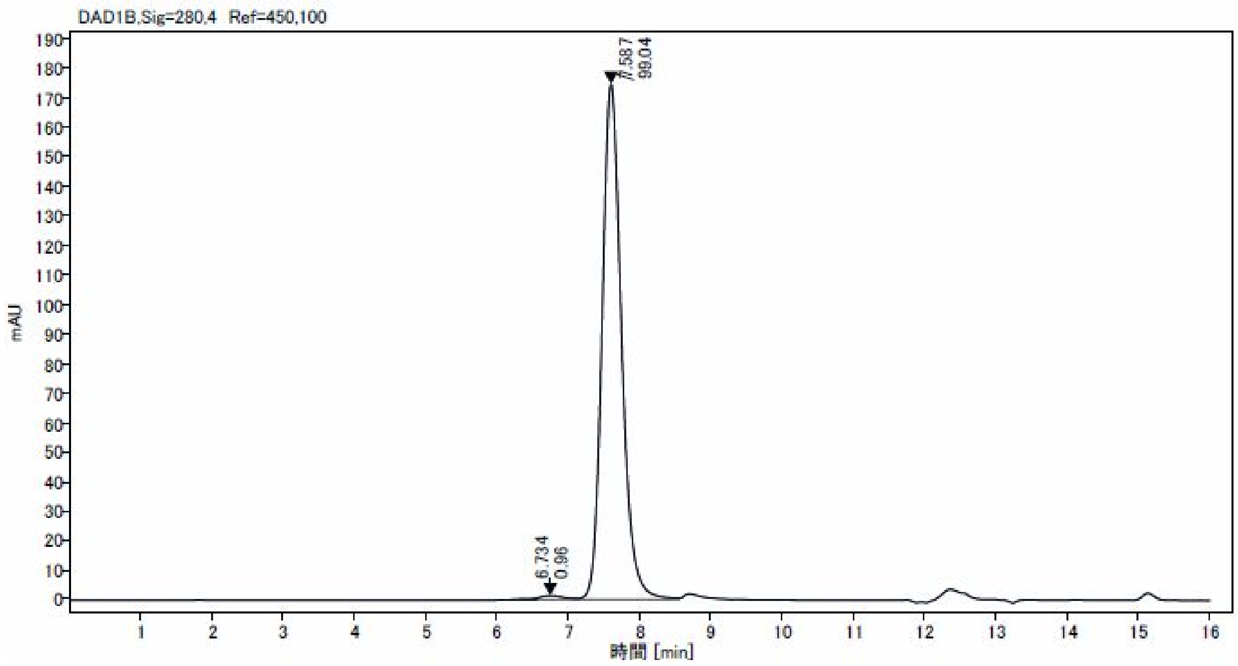

Figure S58. SEC analysis of pembrolizumab-Lys288-thiol derived from AJICAP reagent **6b**

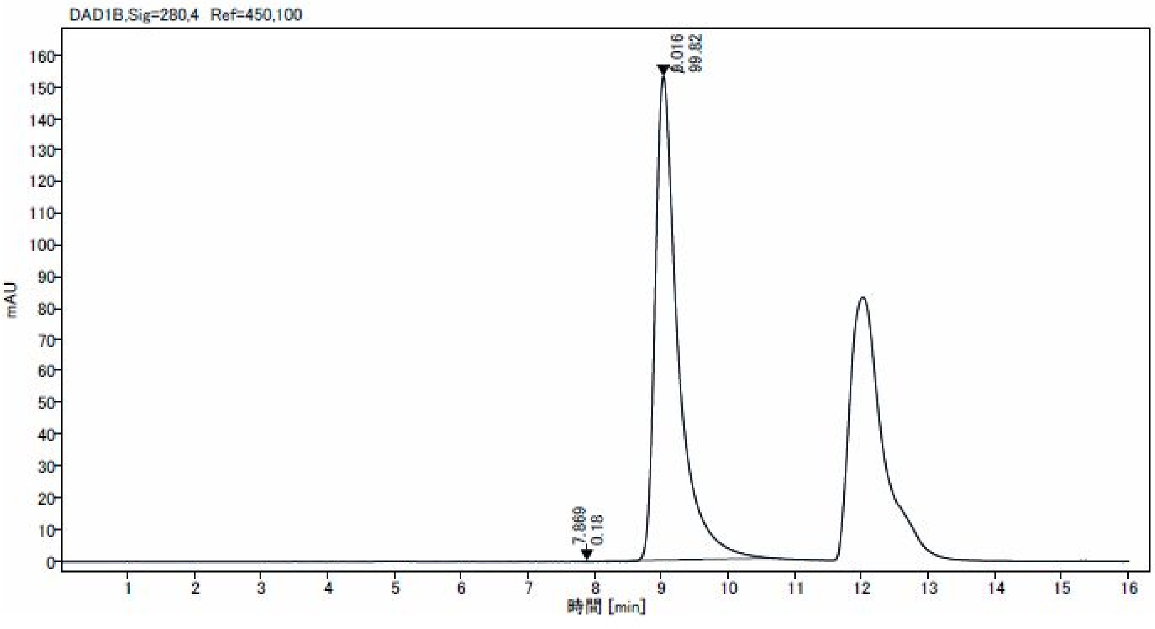

Figure S59. SEC analysis of Fc-Protein-Lys248-thiol derived from AJICAP reagent **1b**

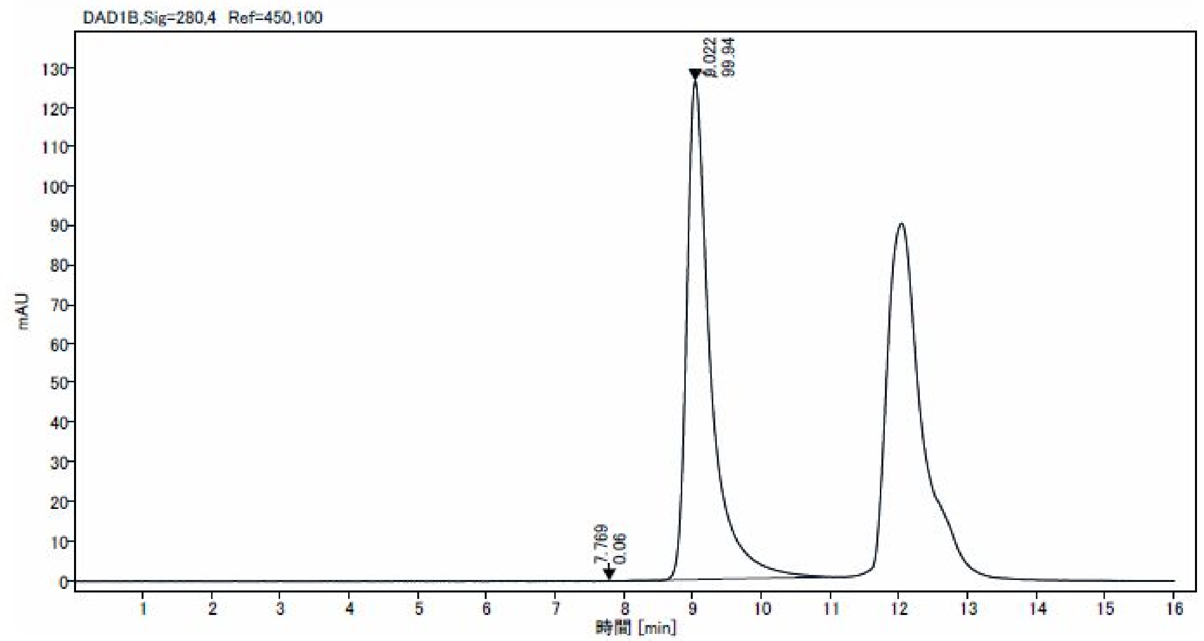

Figure S60. SEC analysis of Fc-Protein-Lys288-thiol derived from AJICAP reagent **6b**

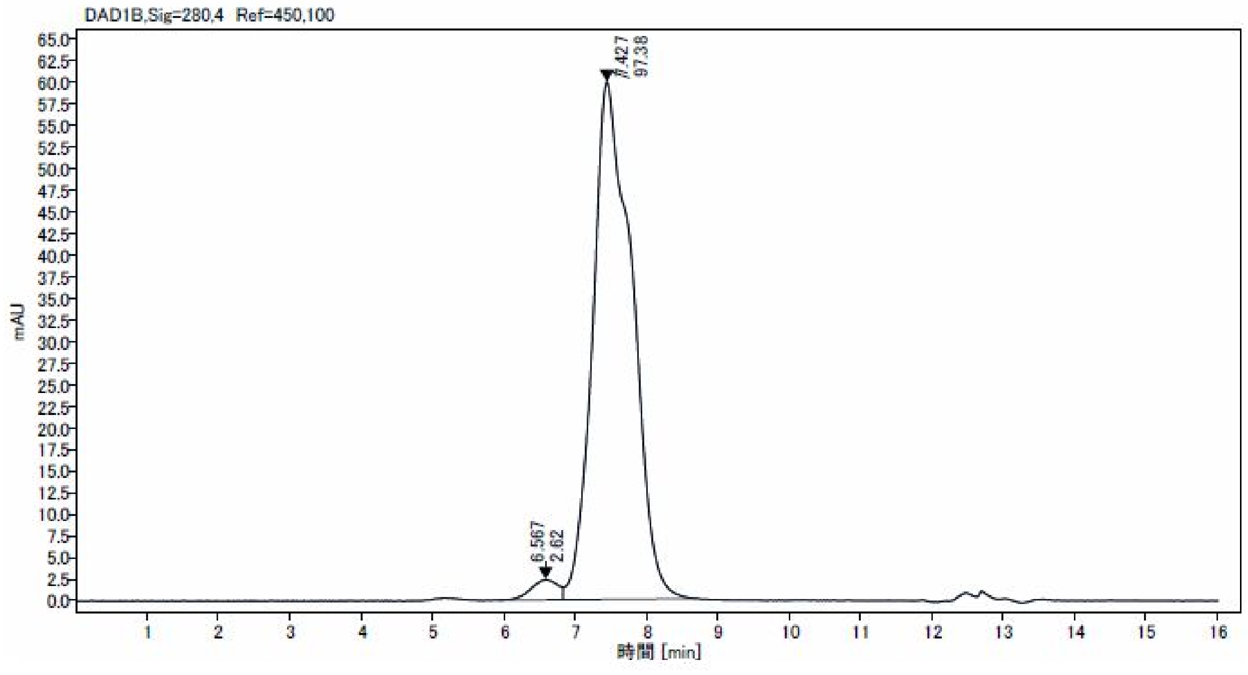

Figure S61. SEC analysis of polyclonal antibody-Lys248-thiol derived from AJICAP reagent **1b**

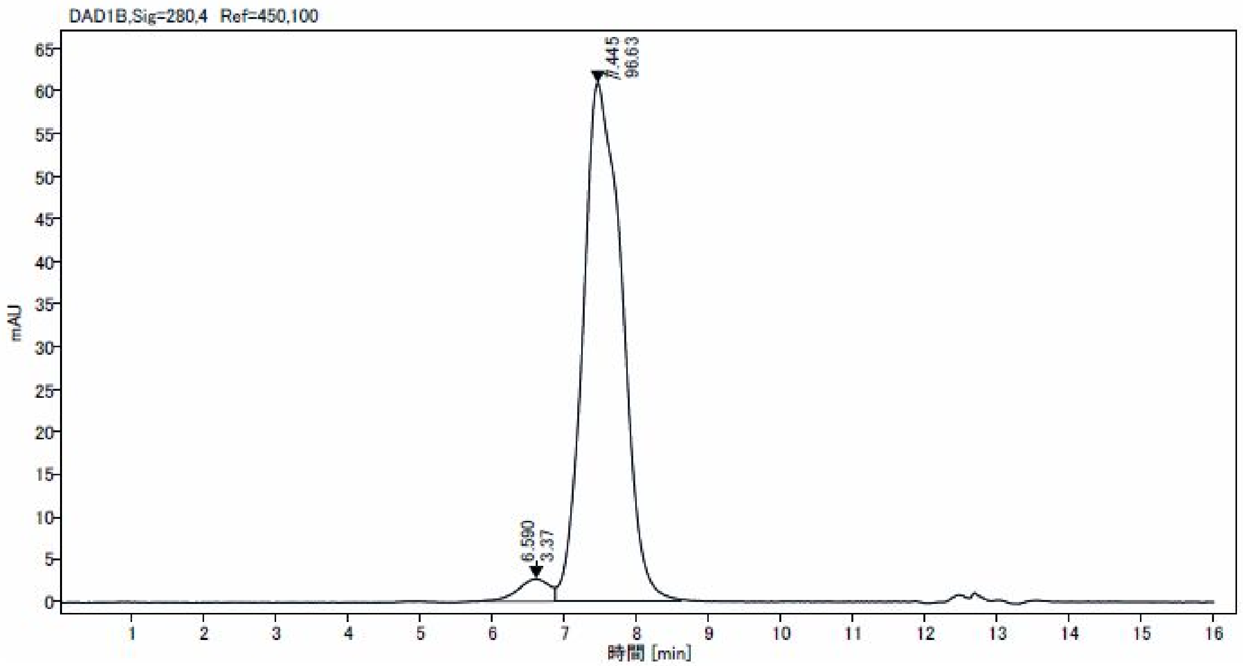

Figure S62. SEC analysis of polyclonal mAb-Lys288-thiol derived from AJICAP reagent **6b**

2-5 ADC syntheses: payload-linker structure, Q-TOF MS, HIC, and SEC analysis

2-5-1 payload-linker structure

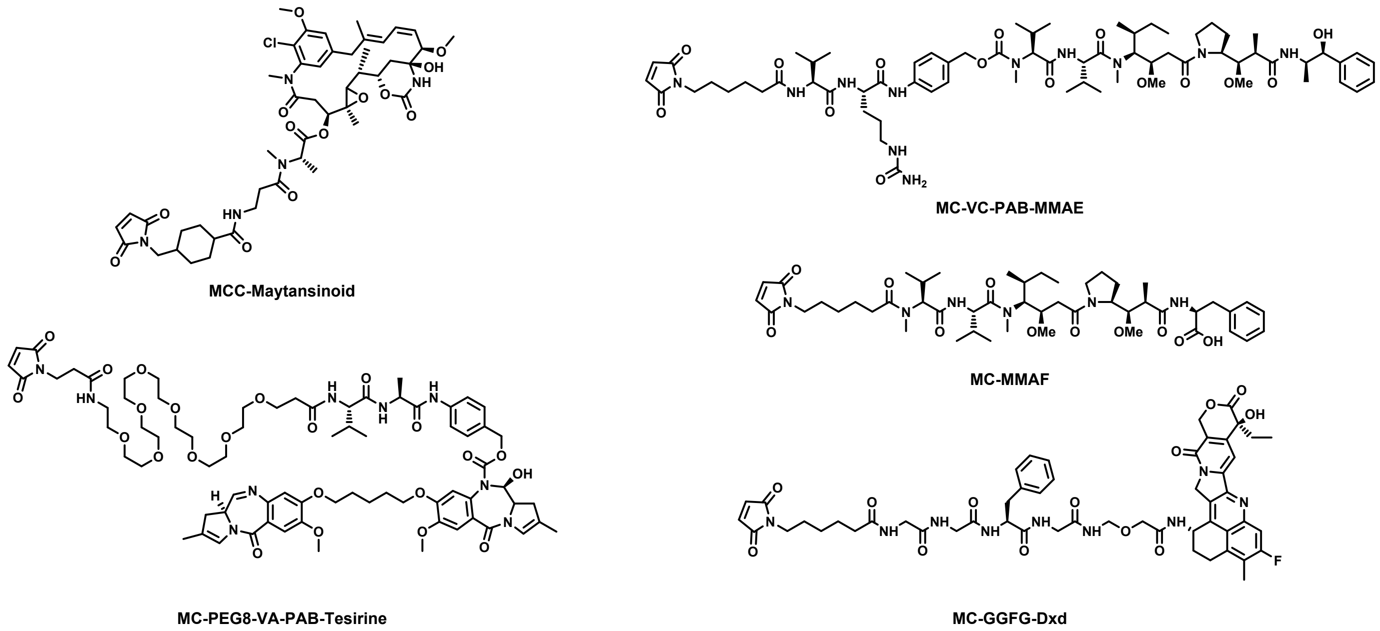

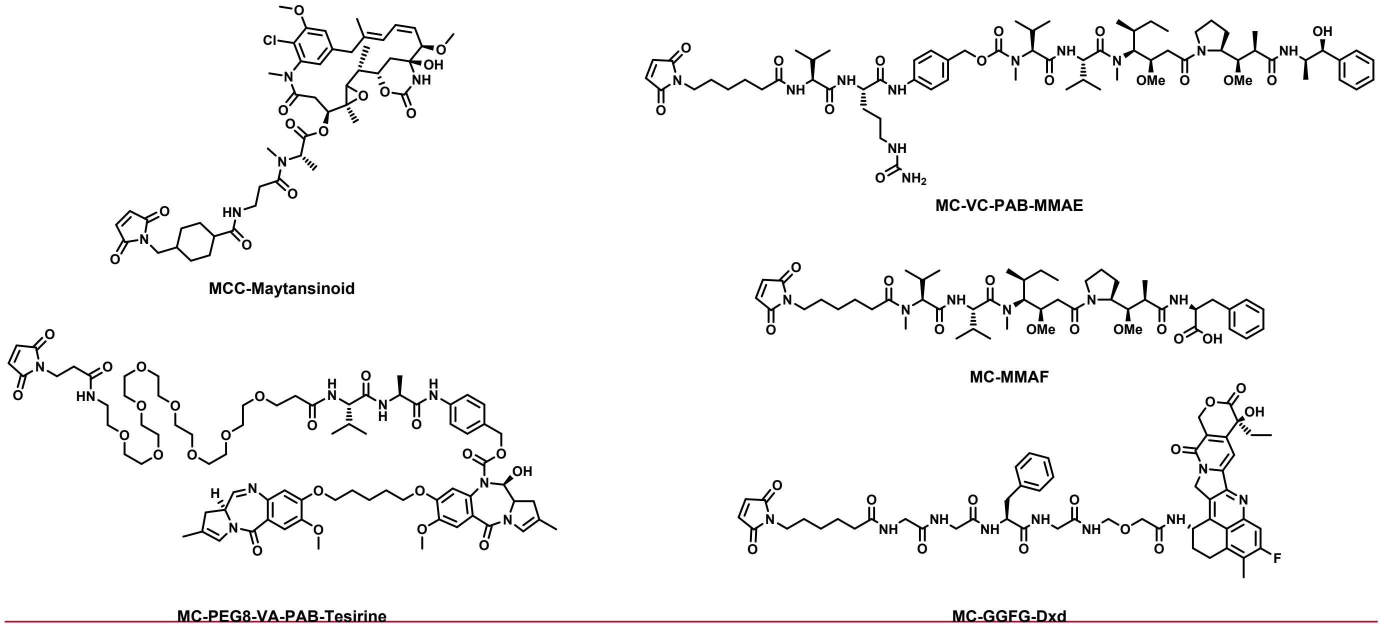

Figure S63~~56~~. Chemical structure of each payload-linkers

2-5-2 Q-TOF MS analysis

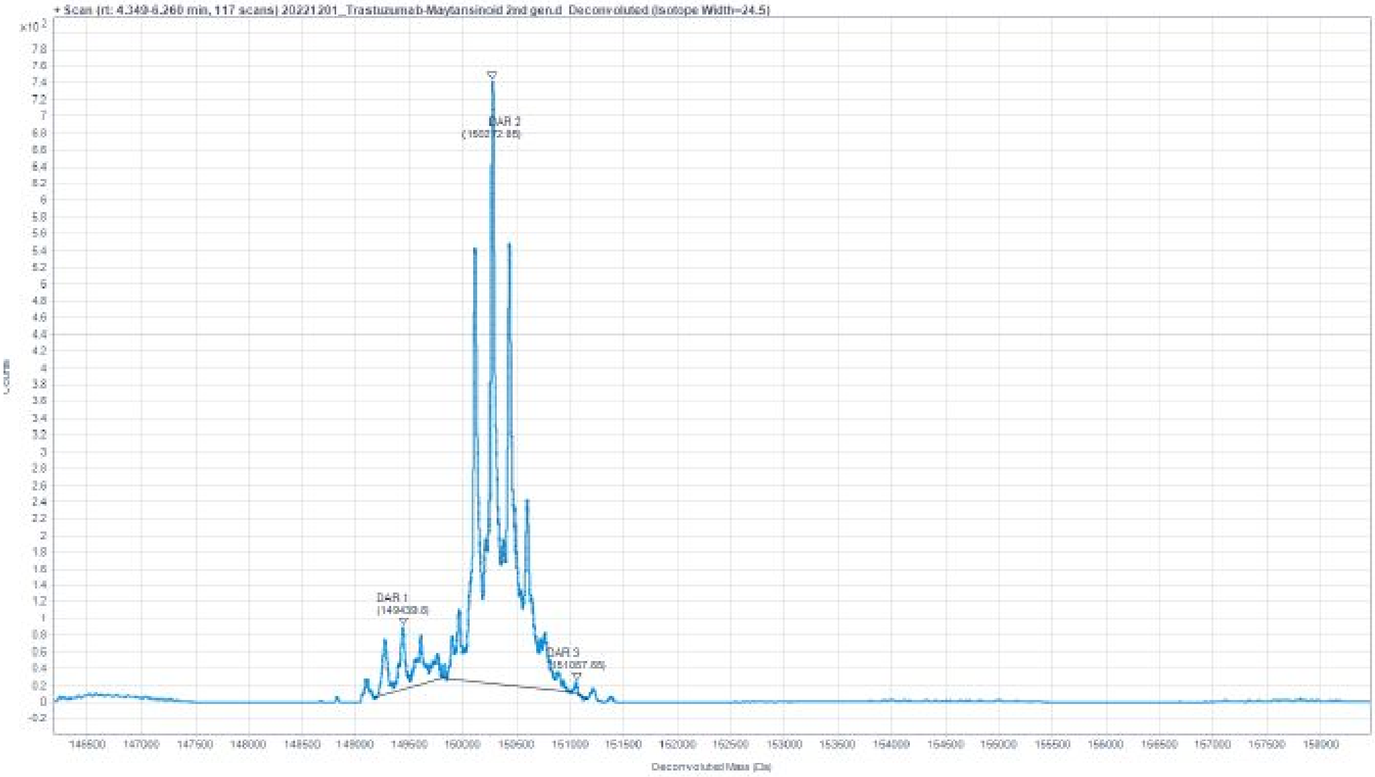

DAR Peak List

DAR Peak	Theoretical Mass (Da)	Observed Mass (Da)	Area	% Area
1	149249	149439.8	1.55E+004	9.17
2	150273	150272.85	1.53E+005	90.60
3	151297	151057.65	3.74E+002	0.22

Figure S64~~57~~. Q-TOF analysis of trastuzumab-Lys248-maytansinoid

Average DAR = 1.9

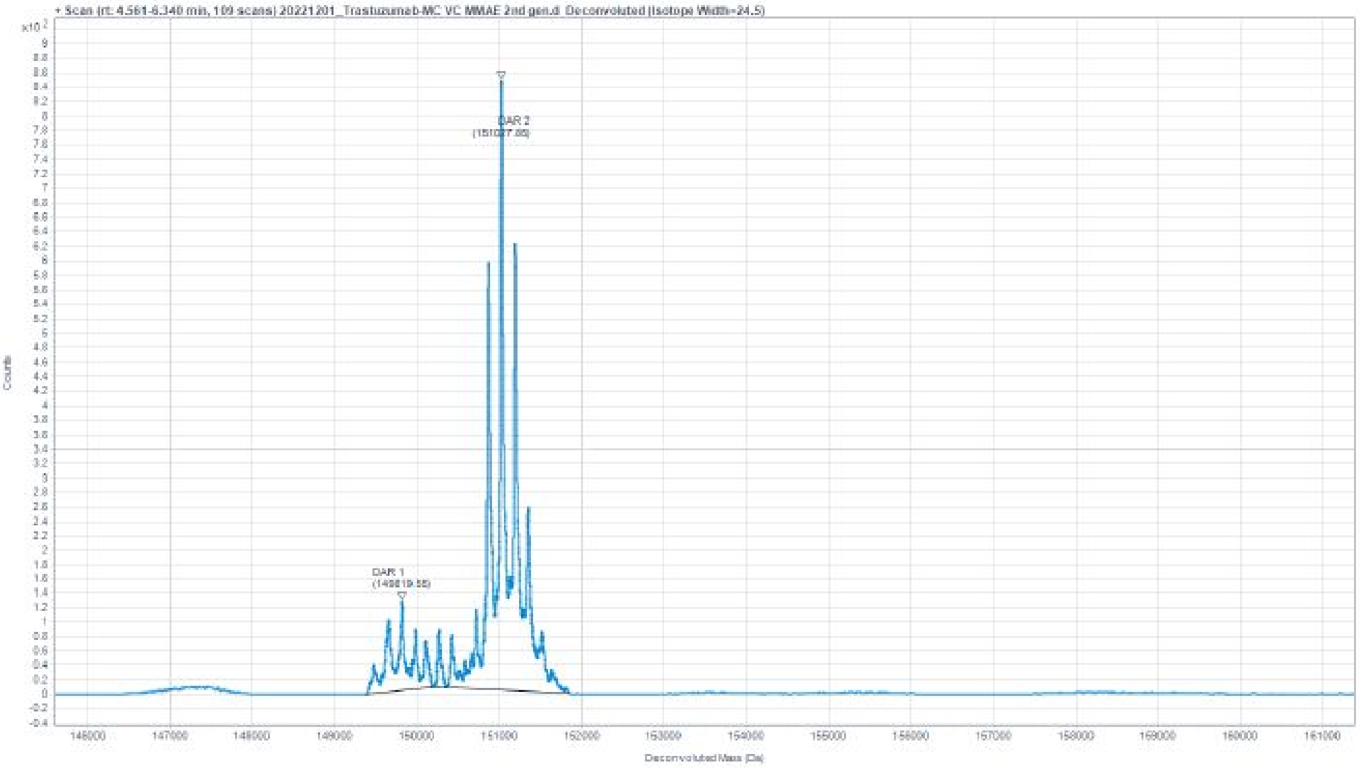

DAR Peak List

DAR Peak	Theoretical Mass (Da)	Observed Mass (Da)	Area	% Area
1	149541	149819.55	2.86E+004	14.20
2	150857	151027.85	1.73E+005	85.80

Figure S65~~58~~. Q-TOF analysis of trastuzumab-Lys248-MMAE

Average DAR = 1.9

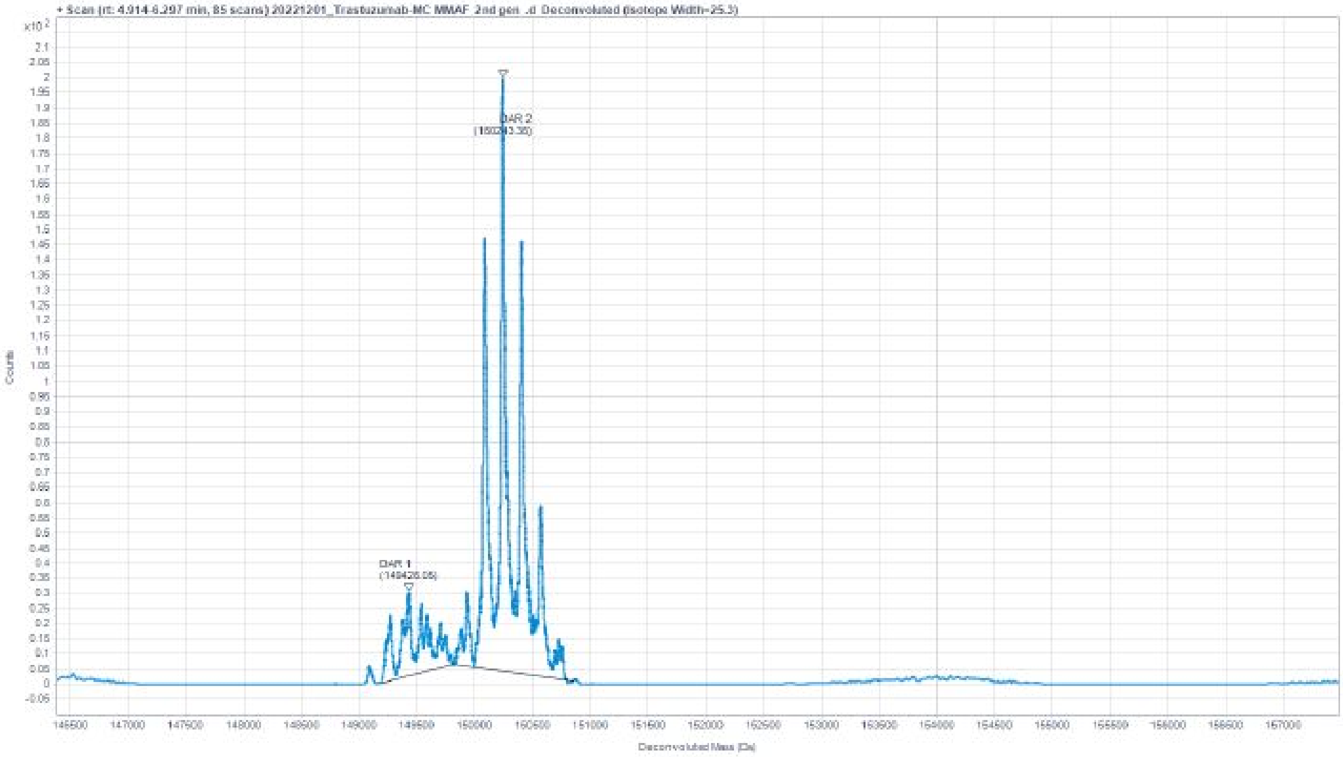

DAR Peak List

DAR Peak	Theoretical Mass (Da)	Observed Mass (Da)	Area	% Area
1	149150	149426.05	5.99E+003	16.31
2	150075	150243.35	3.07E+004	83.69

Figure S66~~59~~. Q-TOF analysis of trastuzumab-Lys248-MMAF

Average DAR = 1.8

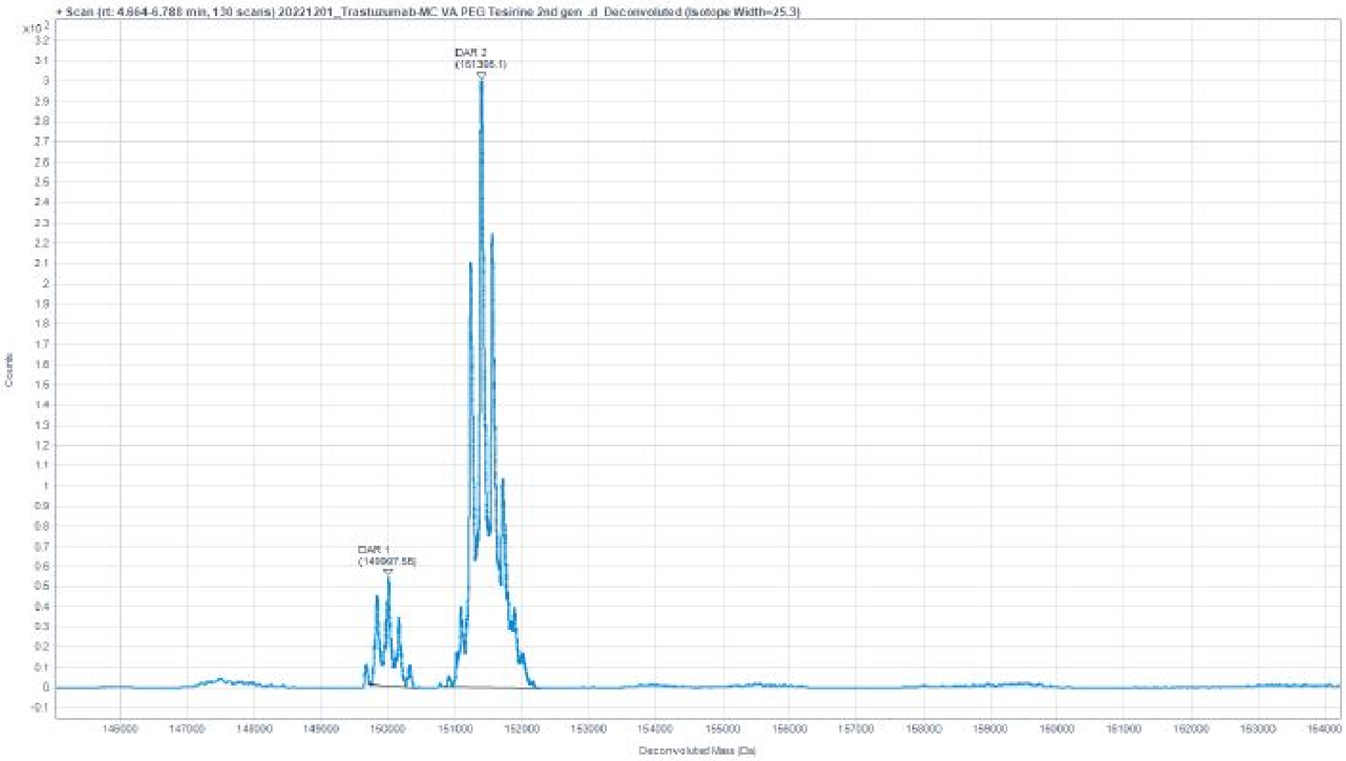

DAR Peak List

DAR Peak	Theoretical Mass (Da)	Observed Mass (Da)	Area	% Area
1	149825	149997.55	1.06E+004	11.68
2	151425	151395.1	8.04E+004	88.32

Figure S67~~0~~. Q-TOF analysis of trastuzumab-Lys248-tesirine

Average DAR = 1.9

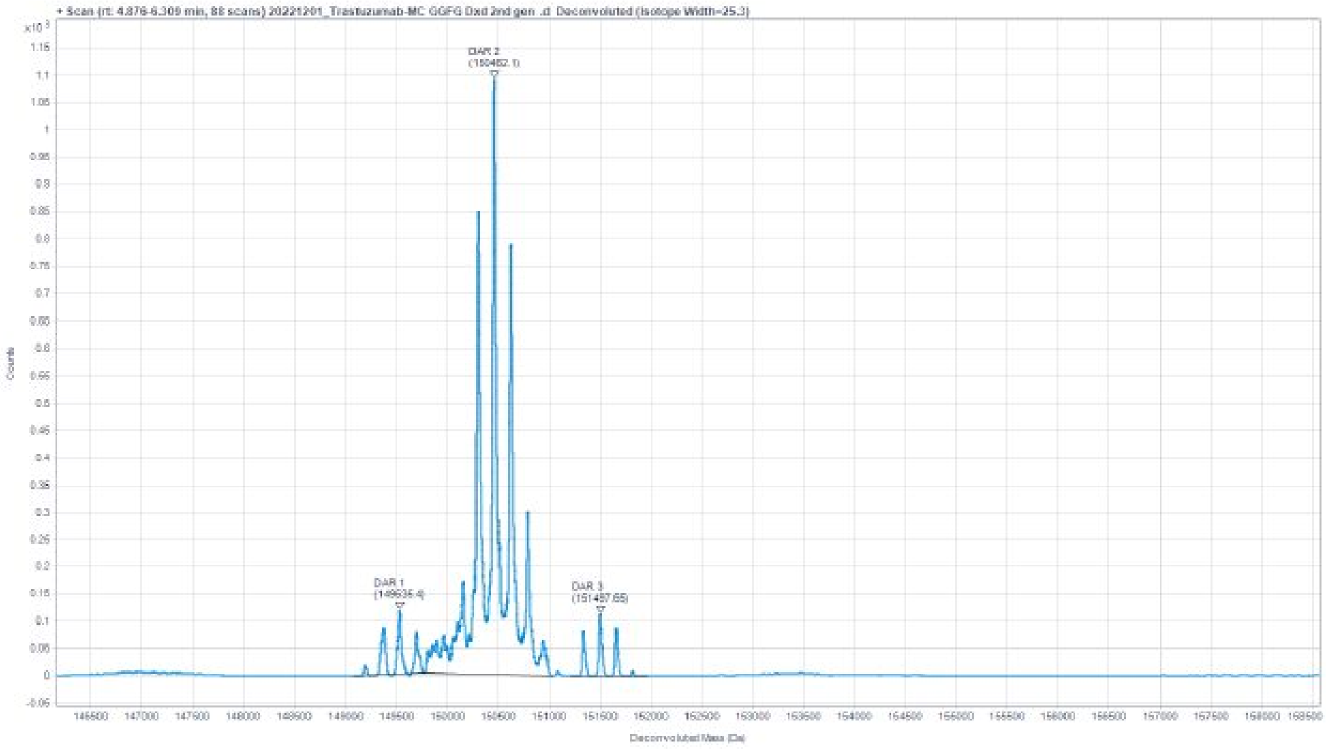

DAR Peak List

DAR Peak	Theoretical Mass (Da)	Observed Mass (Da)	Area	% Area
1	149259	149535.4	1.22E+004	5.88
2	150293	150462.1	1.86E+005	89.63
3	151327	151497.65	9.33E+003	4.49

Figure S68~~1~~. Q-TOF analysis of trastuzumab-Lys248-maytansinoid

Average DAR = 2.0

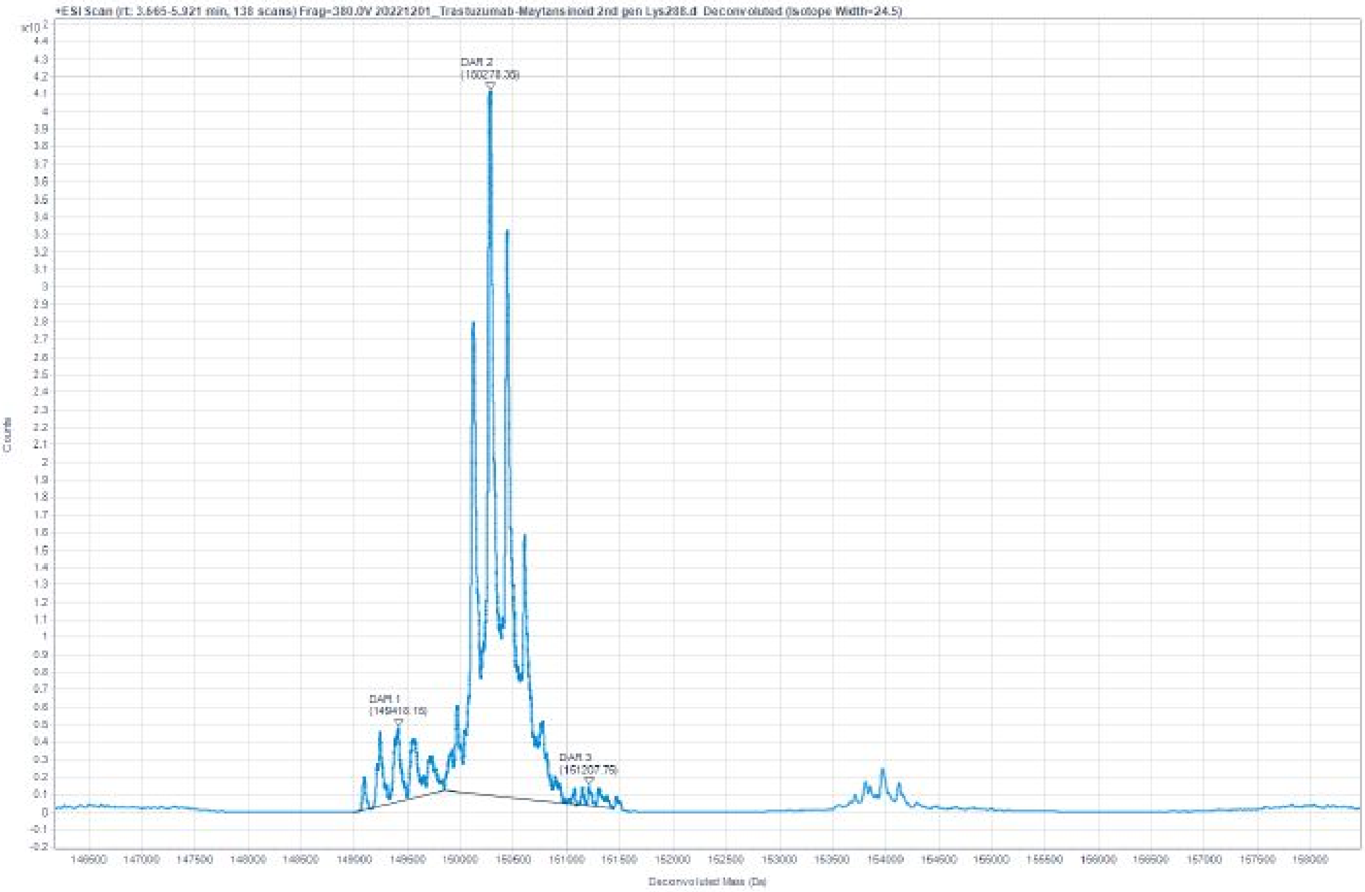

DAR Peak List

DAR Peak	Theoretical Mass (Da)	Observed Mass (Da)	Area	% Area
1	149249	149418.15	1.12E+004	10.29
2	150273	150278.35	9.60E+004	88.06
3	151297	151207.75	1.80E+003	1.65

Figure S69~~2~~. Q-TOF analysis of trastuzumab-Lys288-maytansinoid

Average DAR = 1.9

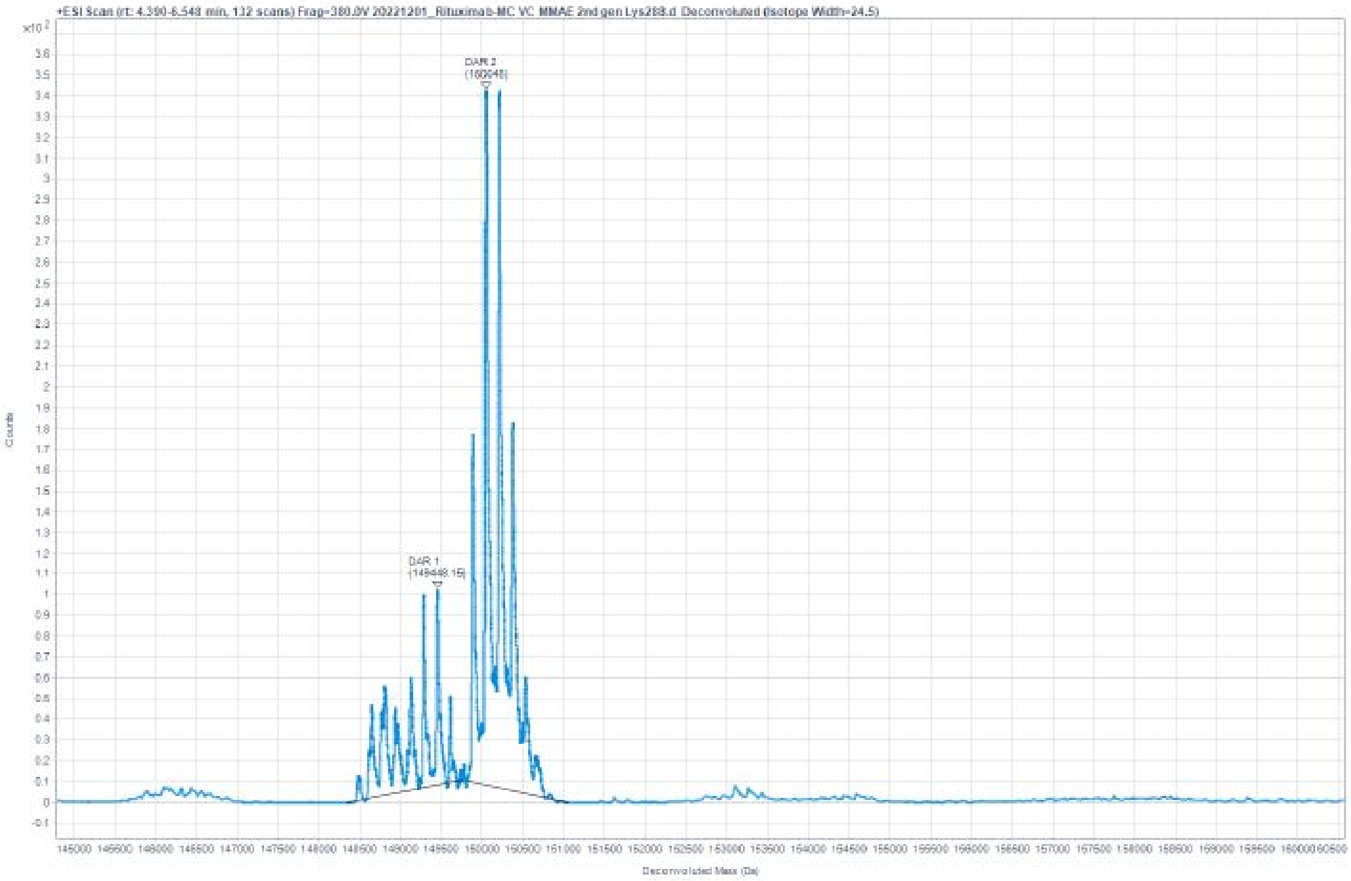

DAR Peak List

DAR Peak	Theoretical Mass (Da)	Observed Mass (Da)	Area	% Area
1	148720	149448.15	2.31E+004	26.39
2	150036	150046	6.43E+004	73.61

Figure S~~63~~70. Q-TOF analysis of trastuzumab-Lys288-MMAE

Average DAR = 1.8

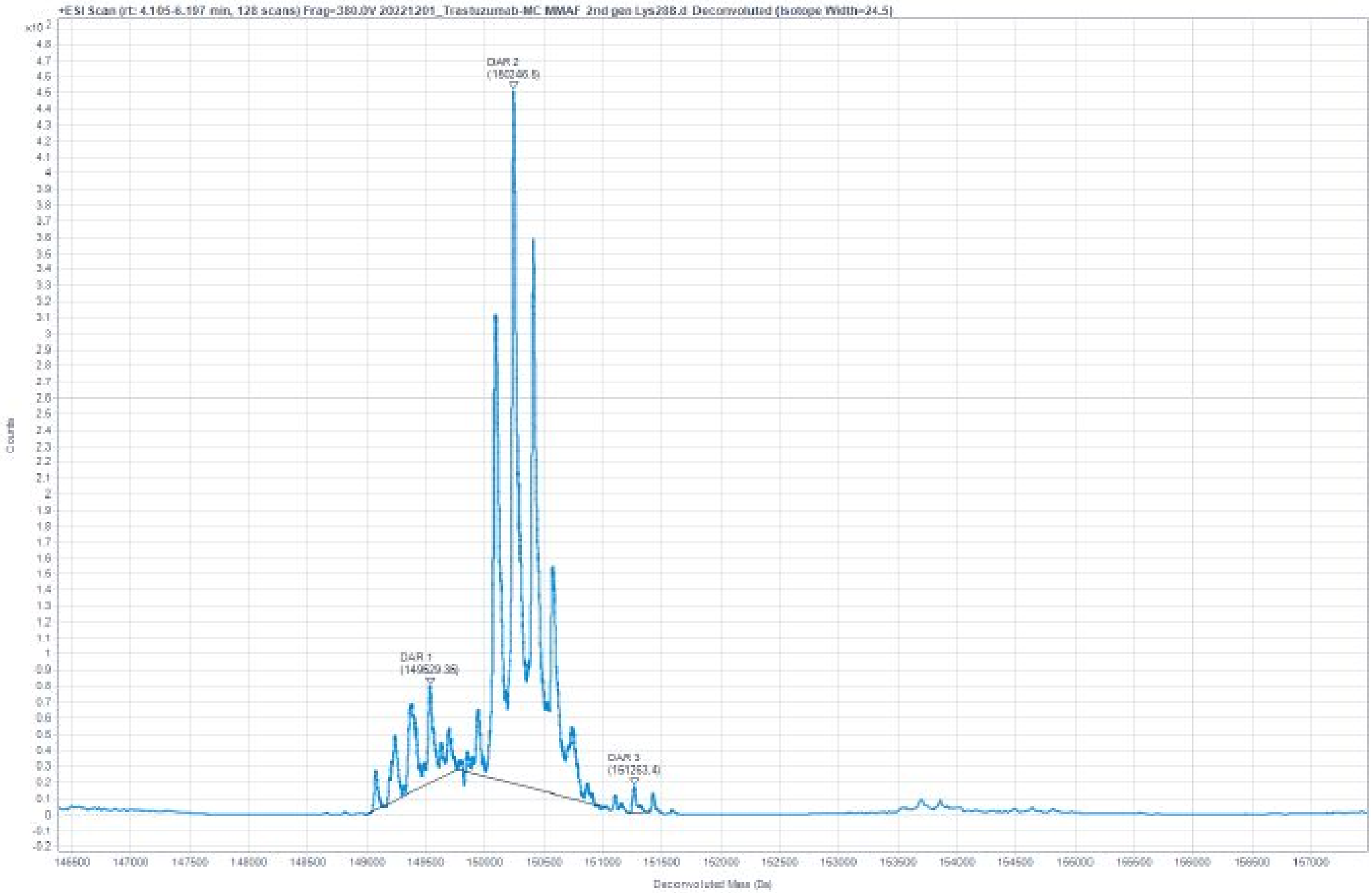

DAR Peak List

DAR Peak	Theoretical Mass (Da)	Observed Mass (Da)	Area	% Area
1	149150	149529.35	1.27E+004	12.74
2	150075	150246.5	8.65E+004	86.56
3	151000	151263.4	7.02E+002	0.70

Figure S7164. Q-TOF analysis of trastuzumab-Lys288-MMAF

Average DAR = 1.9

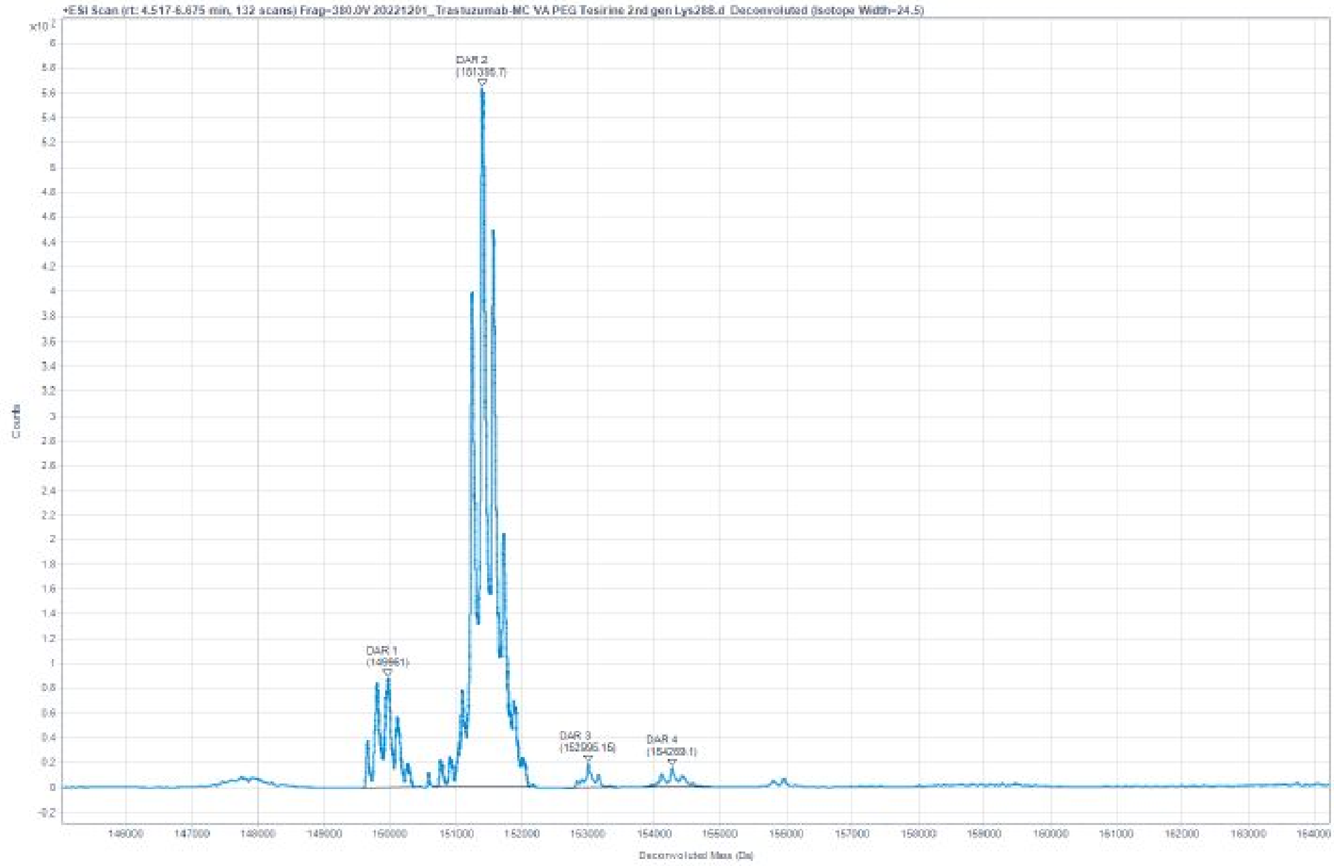

DAR Peak List

DAR Peak	Theoretical Mass (Da)	Observed Mass (Da)	Area	% Area
1	149825	149961	2.42E+004	12.49
2	151425	151395.7	1.63E+005	84.10
3	153025	152995.15	2.97E+003	1.53
4	154625	154269.1	3.64E+003	1.88

Figure S72~~65~~. Q-TOF analysis of trastuzumab-Lys288-tesirine

Average DAR = 2.0

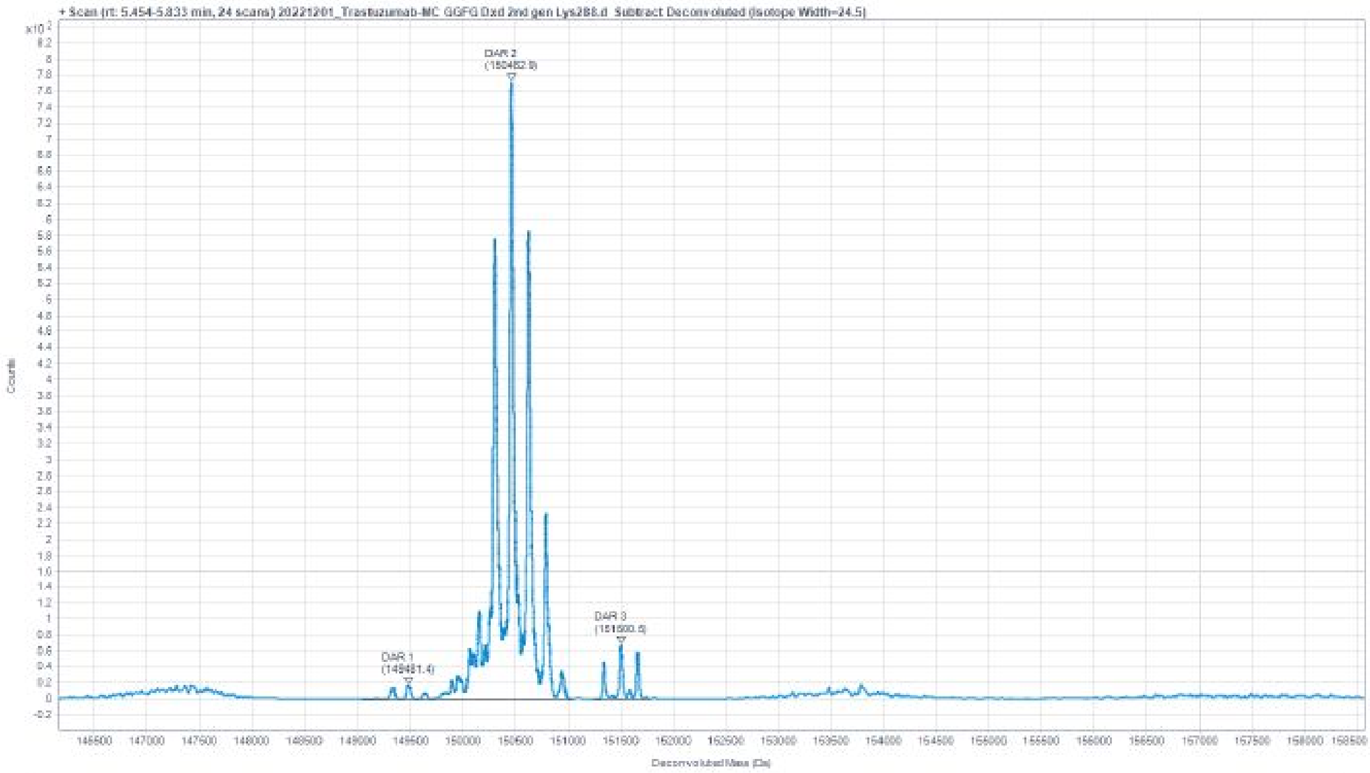

DAR Peak List

DAR Peak	Theoretical Mass (Da)	Observed Mass (Da)	Area	% Area
1	149259	149481.4	1.57E+003	1.14
2	150293	150462.9	1.31E+005	95.08
3	151327	151500.5	5.21E+003	3.78

Figure S~~66~~73. Q-TOF analysis of trastuzumab-Lys288-Dxd

Average DAR = 2.0

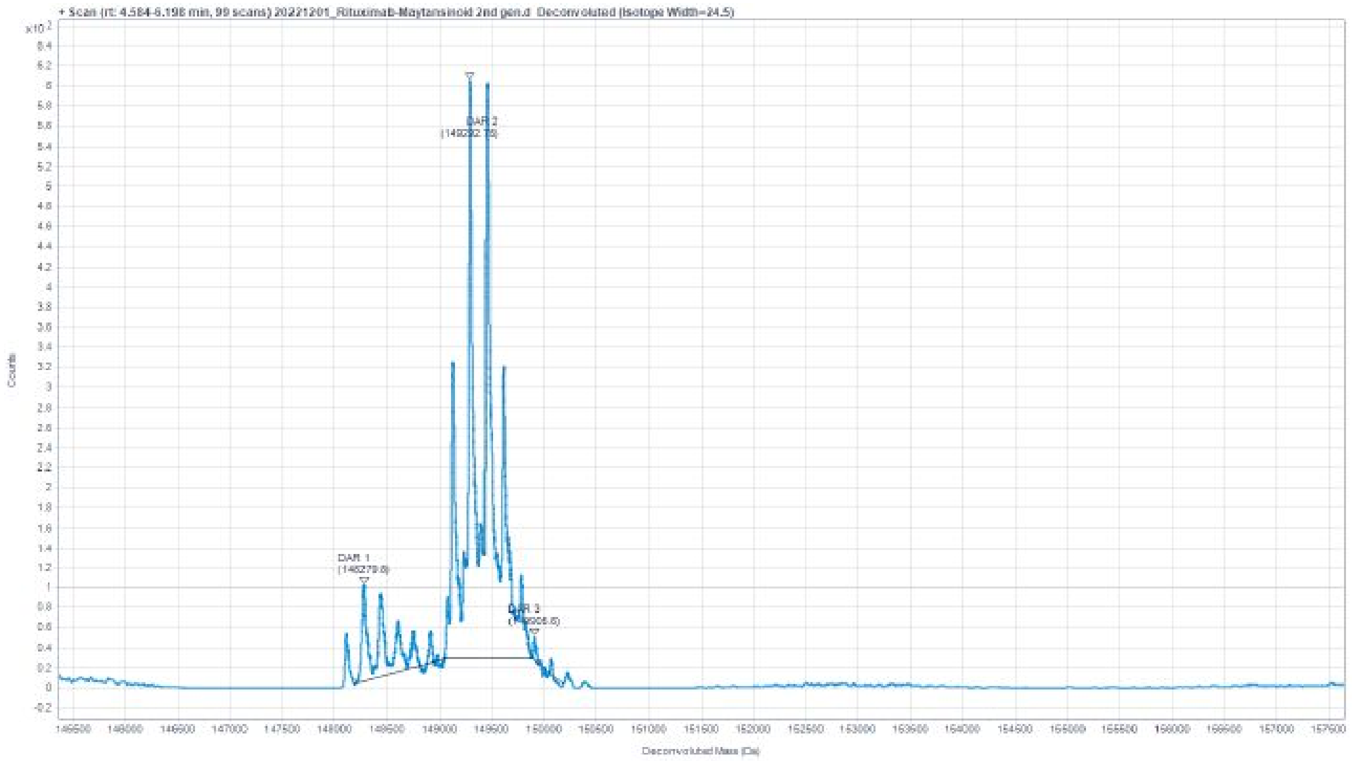

DAR Peak List

DAR Peak	Theoretical Mass (Da)	Observed Mass (Da)	Area	% Area
1	148428	148279.8	1.60E+004	12.52
2	149452	149292.75	1.12E+005	87.21
3	150476	149905.6	3.48E+002	0.27

Figure S74~~67~~. Q-TOF analysis of rituximab-Lys248-maytansinoid

Average DAR = 1.9

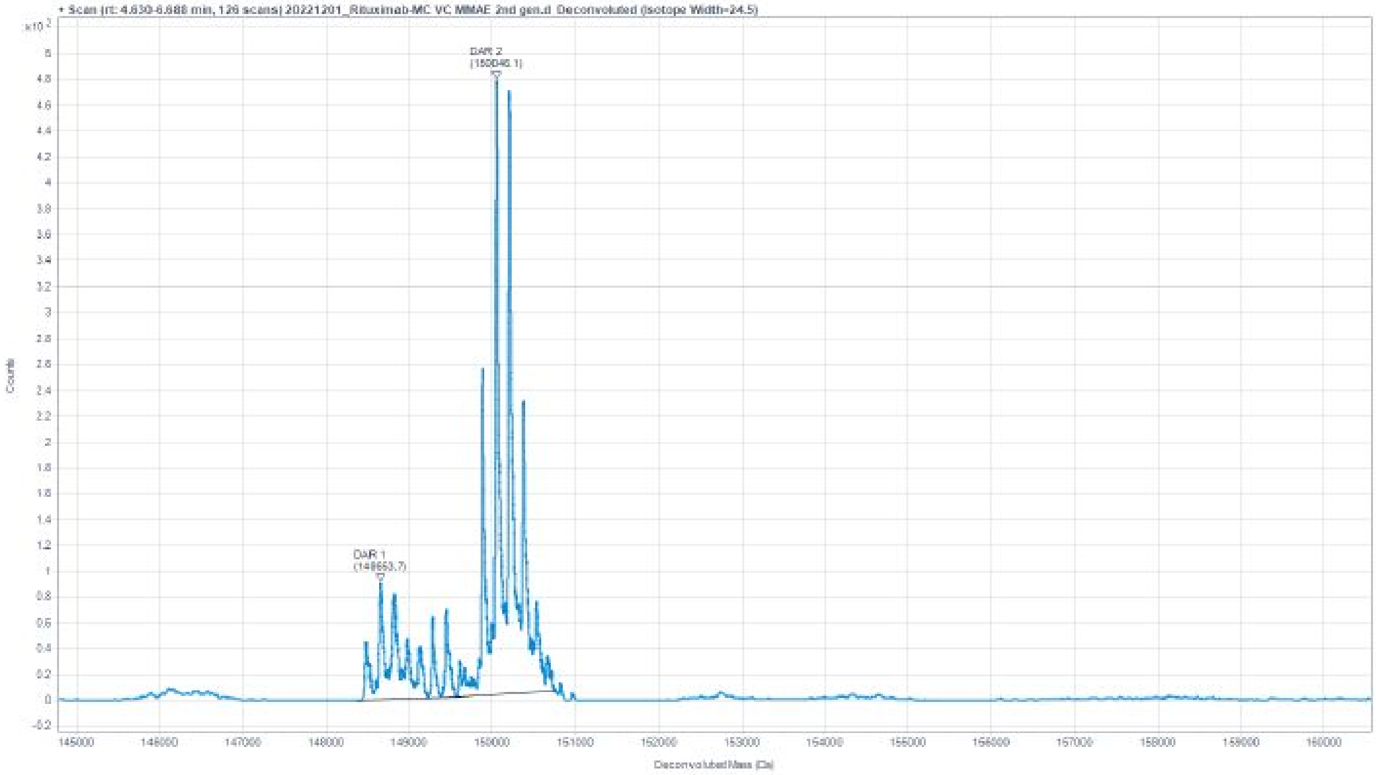

DAR Peak List

DAR Peak	Theoretical Mass (Da)	Observed Mass (Da)	Area	% Area
1	148720	148653.7	2.71E+004	23.55
2	150036	150046.1	8.80E+004	76.45

Figure S~~68~~75. Q-TOF analysis of rituximab-Lys248-MMAE

Average DAR = 1.8

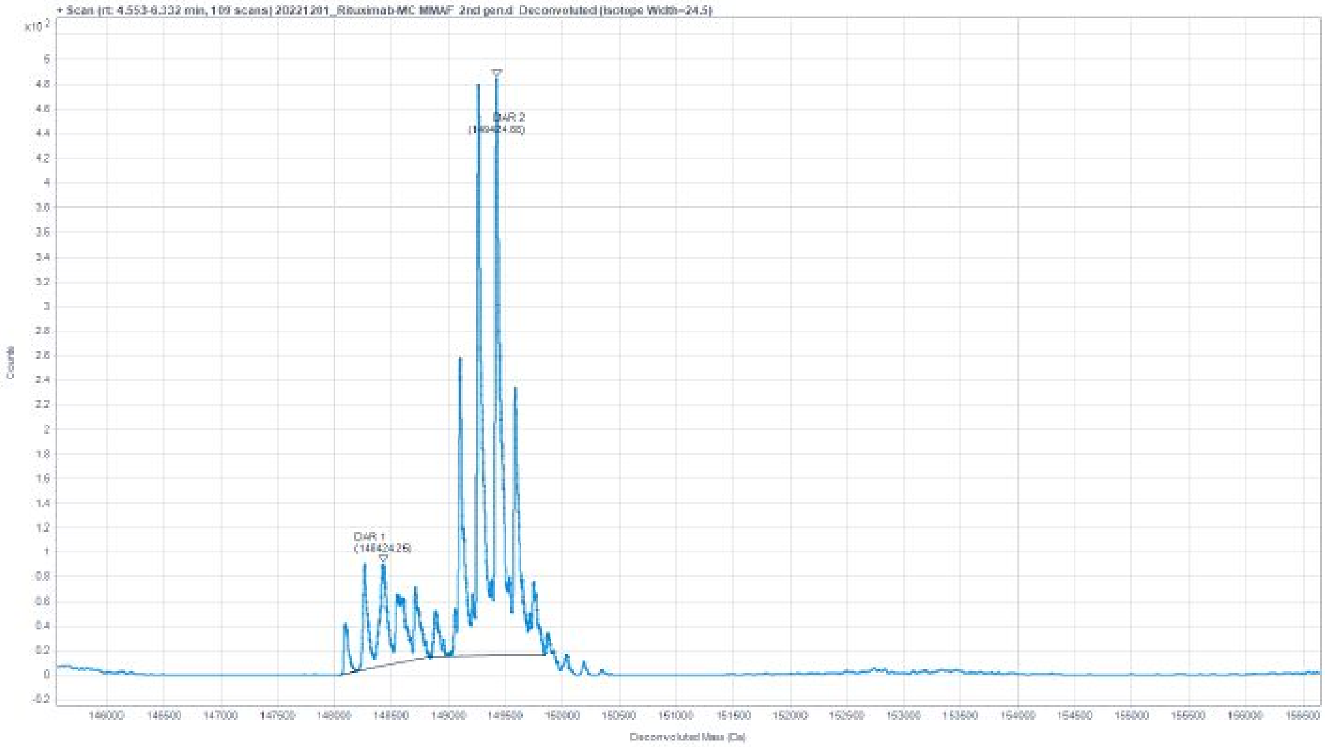

DAR Peak List

DAR Peak	Theoretical Mass (Da)	Observed Mass (Da)	Area	% Area
1	148329	148424.25	2.14E+004	20.90
2	149254	149424.65	8.08E+004	79.10

Figure S76.~~69.~~ Q-TOF analysis of rituximab-Lys248-mMMAF

Average DAR = 1.8

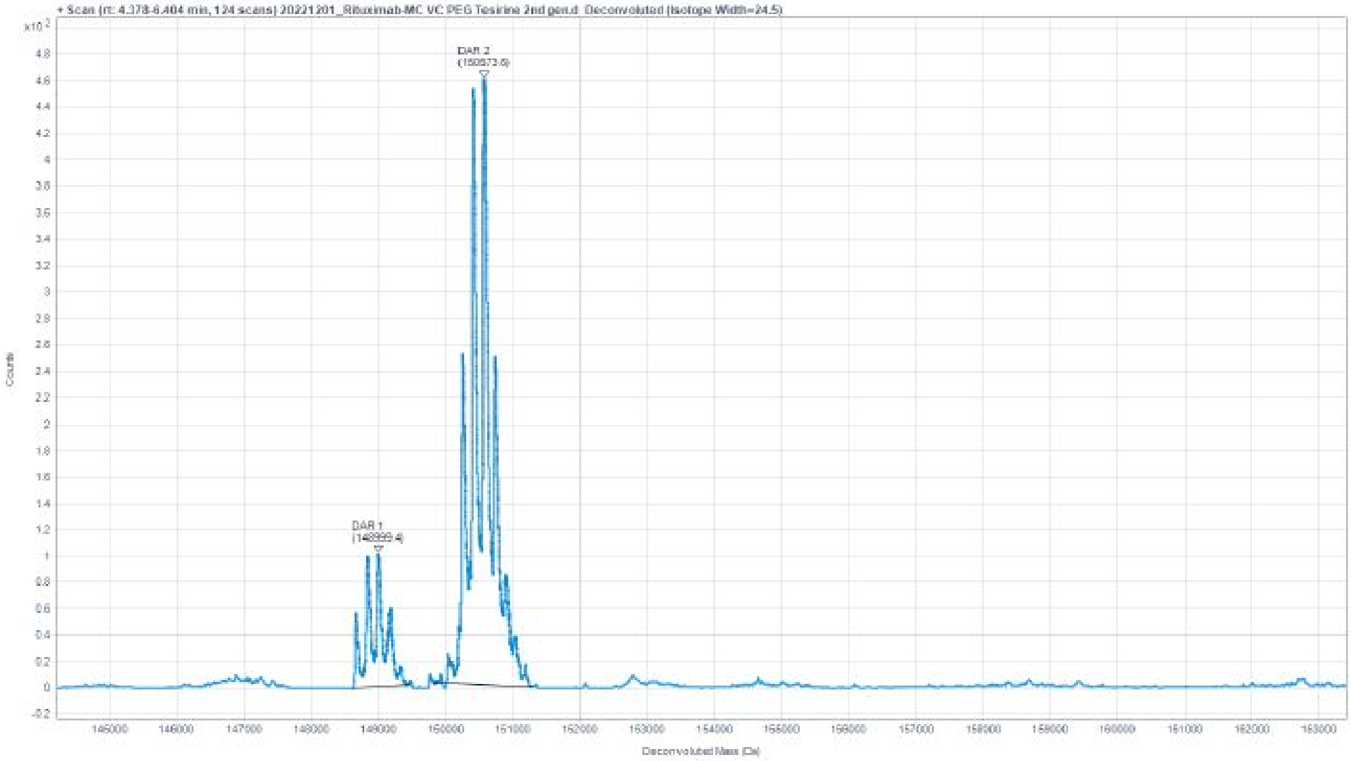

DAR Peak List

DAR Peak	Theoretical Mass (Da)	Observed Mass (Da)	Area	% Area
1	149004	148999.4	2.49E+004	16.15
2	150604	150573.6	1.29E+005	83.85

Figure S770. Q-TOF analysis of rituximab-Lys248-tesirine

Average DAR = 1.8

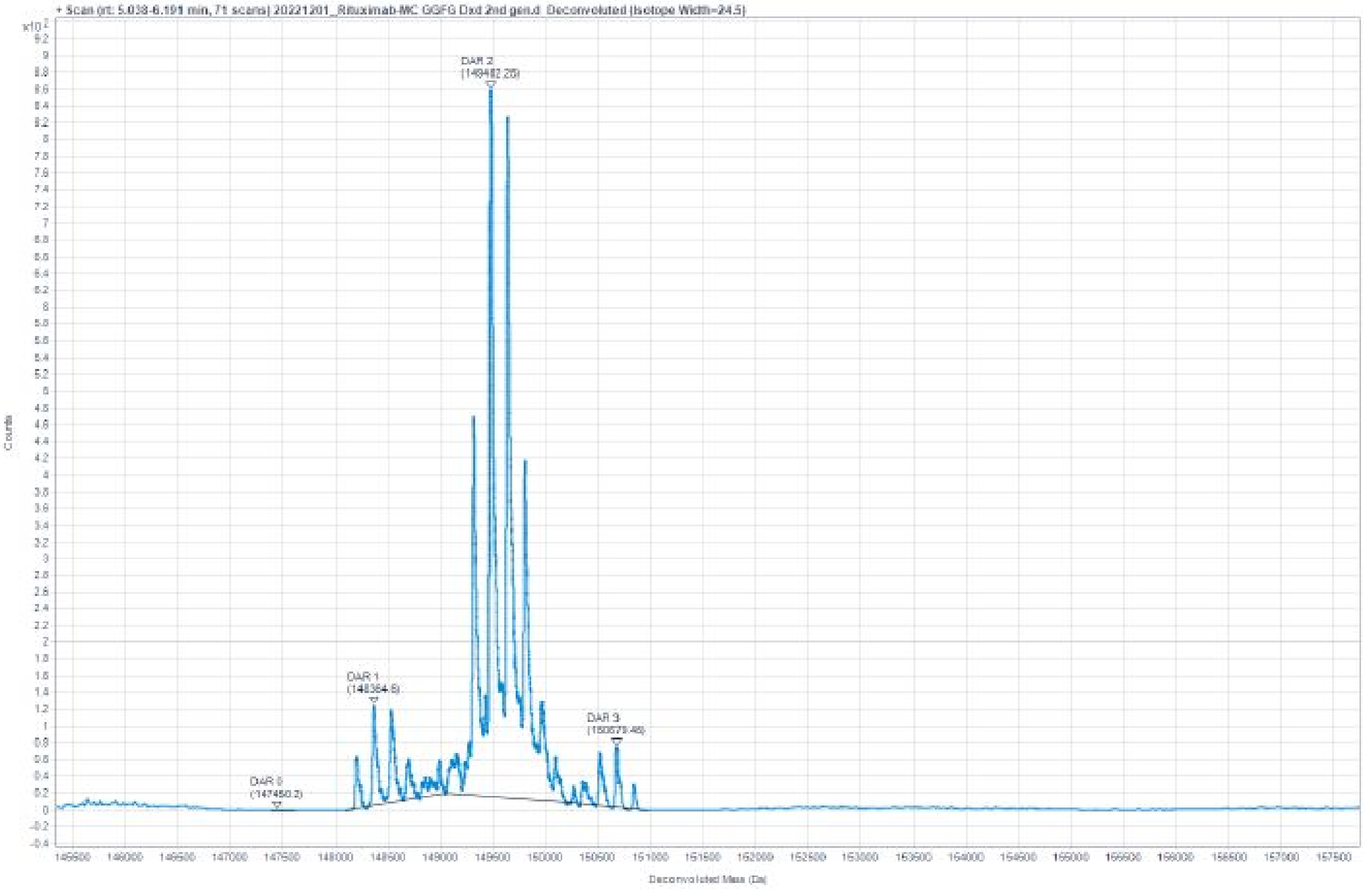

DAR Peak List

DAR Peak	Theoretical Mass (Da)	Observed Mass (Da)	Area	% Area
0	147404	147450.2	6.10E+000	0.00
1	148438	148364.6	2.03E+004	10.73
2	149472	149482.25	1.61E+005	85.17
3	150506	150679.45	7.72E+003	4.09

Figure S7~~1~~8. Q-TOF analysis of rituximab-Lys248-Dxd

Average DAR = 1.9

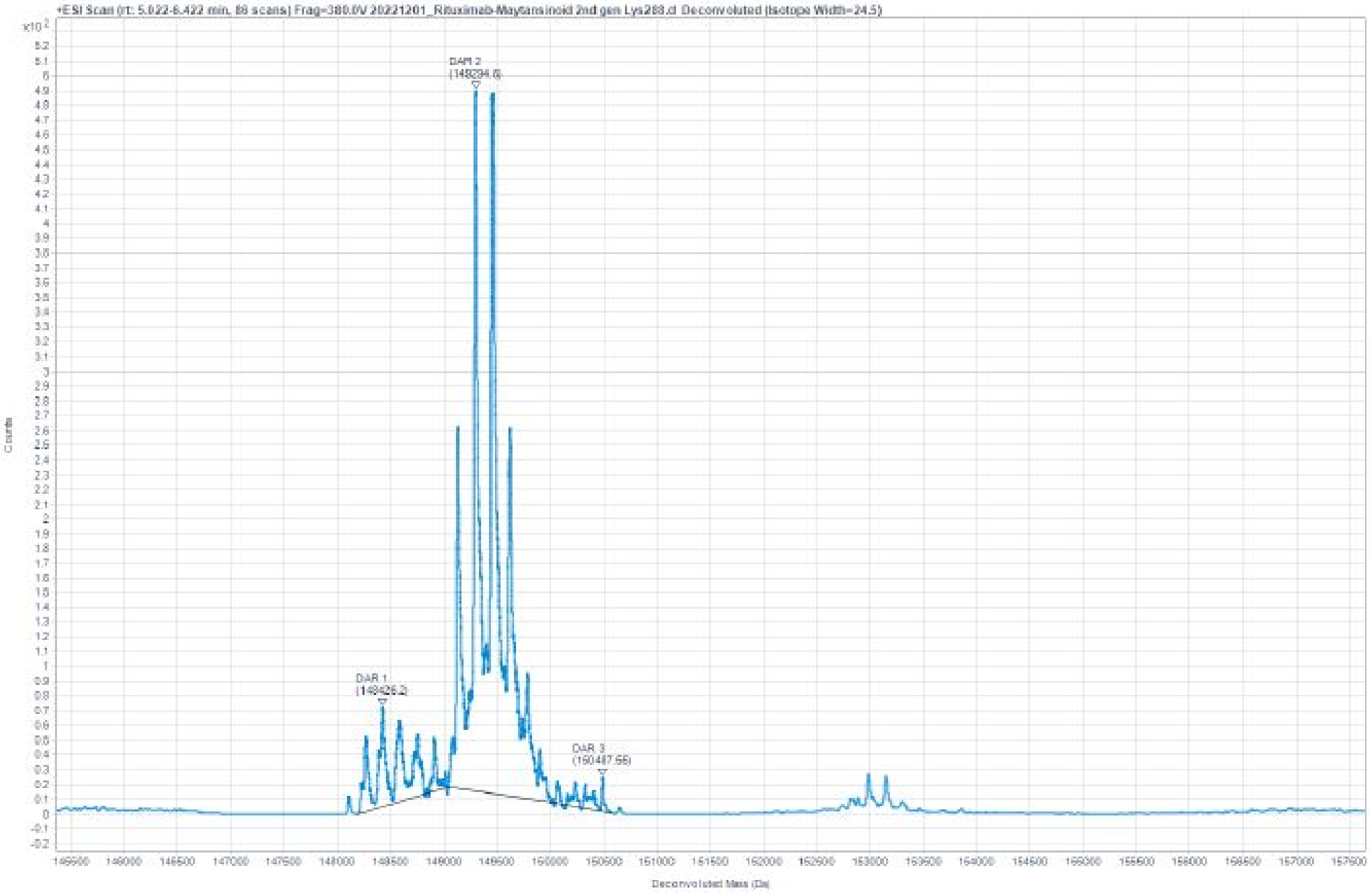

DAR Peak List

DAR Peak	Theoretical Mass (Da)	Observed Mass (Da)	Area	% Area
1	148428	148426.2	1.54E+004	13.14
2	149452	149294.6	9.88E+004	84.35
3	150476	150487.55	2.94E+003	2.51

Figure S792. Q-TOF analysis of rituximab-Lys288-maytansinoid

Average DAR = 1.9

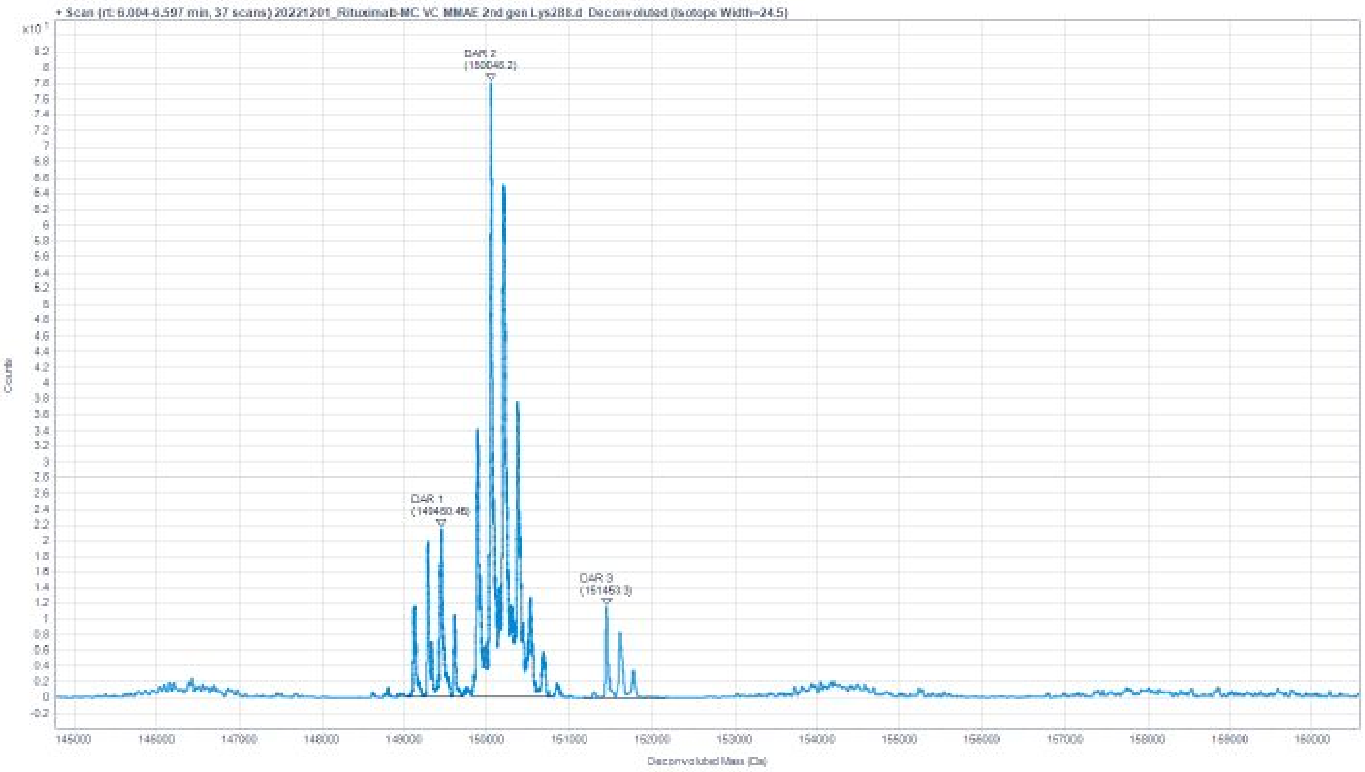

DAR Peak List

DAR Peak	Theoretical Mass (Da)	Observed Mass (Da)	Area	% Area
1	148720	149450.45	2.29E+003	14.91
2	150036	150048.2	1.21E+004	78.80
3	151352	151453.3	9.67E+002	6.29

Figure S~~73~~80. Q-TOF analysis of rituximab-Lys288-MMAE

Average DAR = 1.9

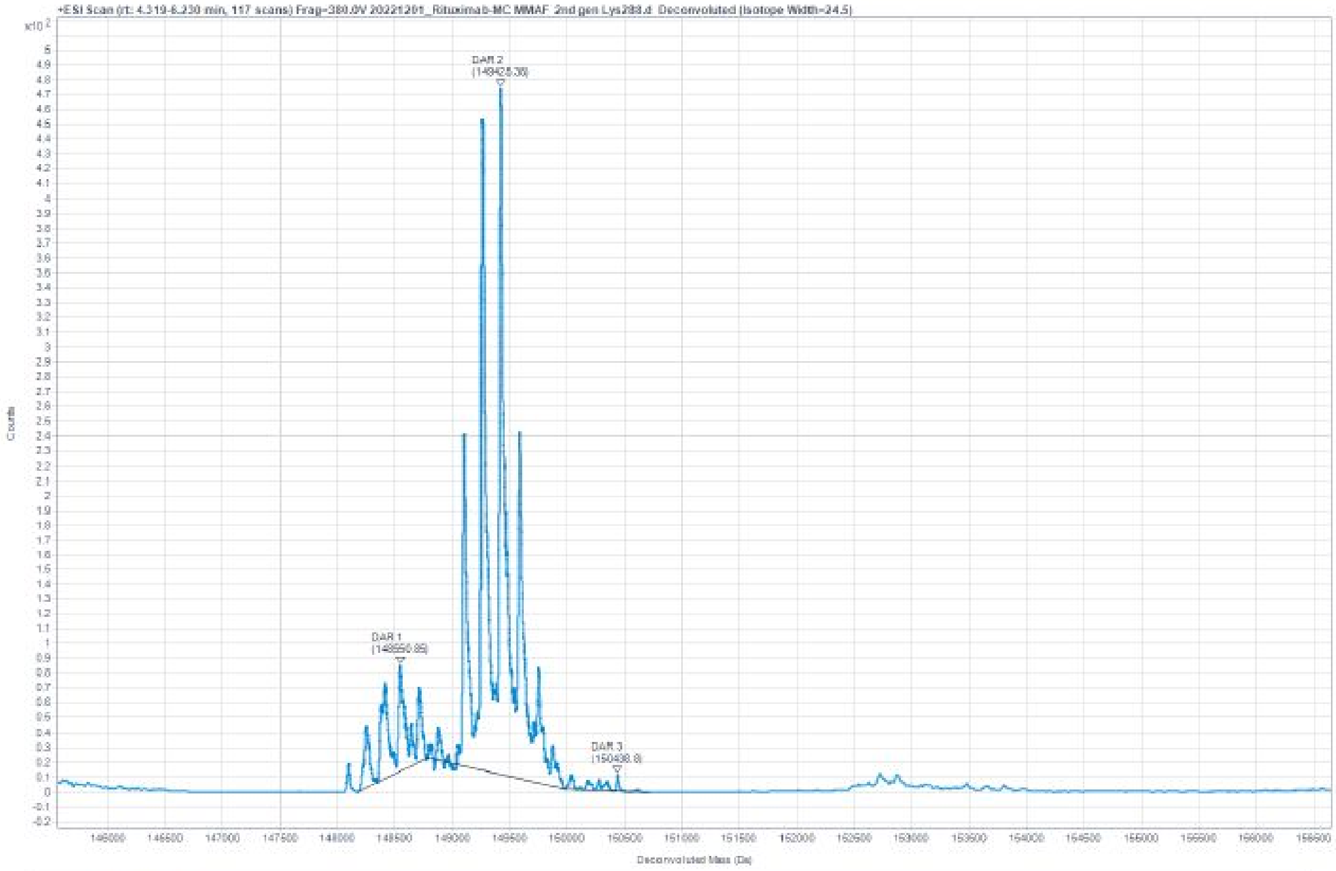

DAR Peak List

DAR Peak	Theoretical Mass (Da)	Observed Mass (Da)	Area	% Area
1	148329	148550.85	1.39E+004	14.56
2	149254	149425.35	8.03E+004	84.02
3	150179	150438.8	1.36E+003	1.42

Figure S8174. Q-TOF analysis of rituximab-Lys288-MMAF

Average DAR = 1.9

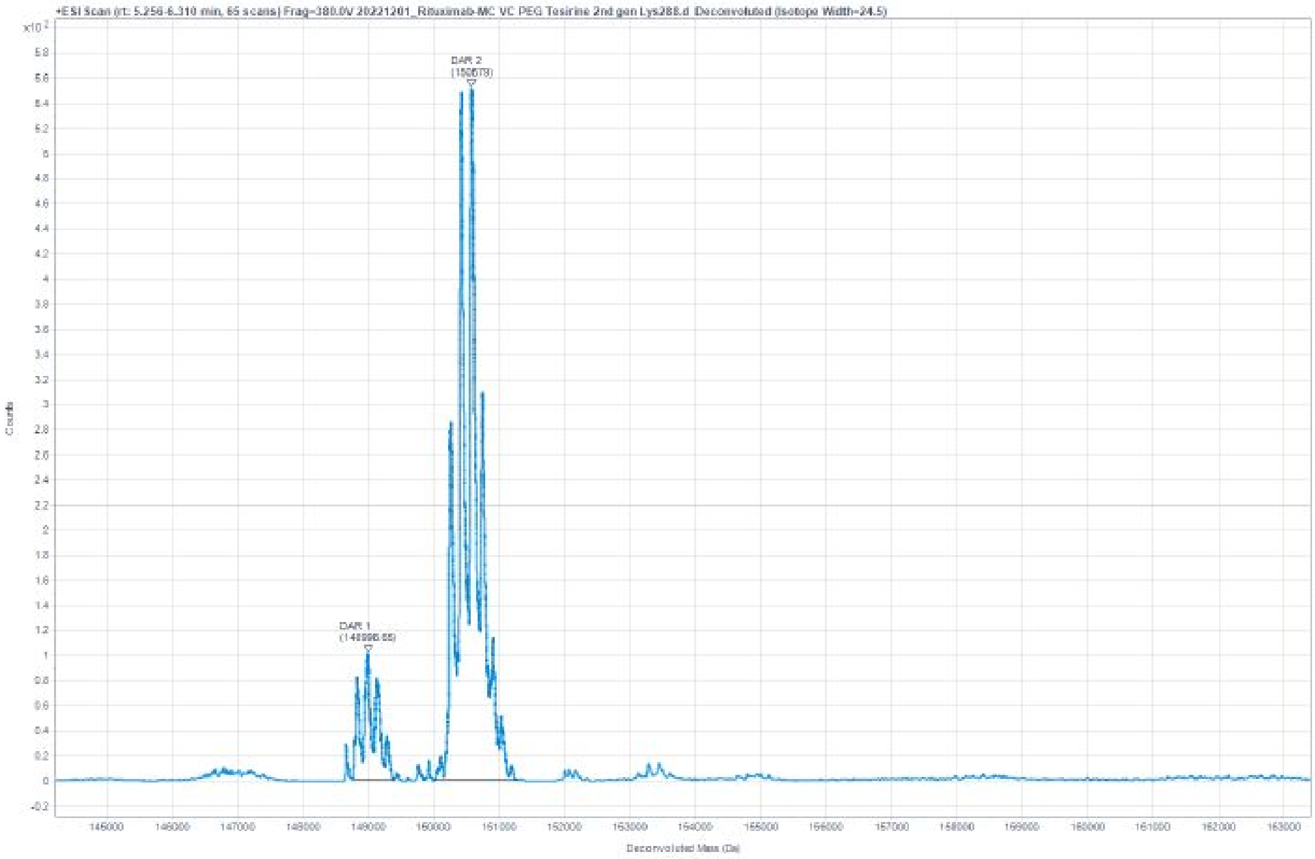

DAR Peak List

DAR Peak	Theoretical Mass (Da)	Observed Mass (Da)	Area	% Area
1	149004	148996.65	2.53E+004	13.75
2	150604	150579	1.59E+005	86.25

Figure S82~~75~~. Q-TOF analysis of rituximab-Lys288-tesirine

Average DAR = 1.9

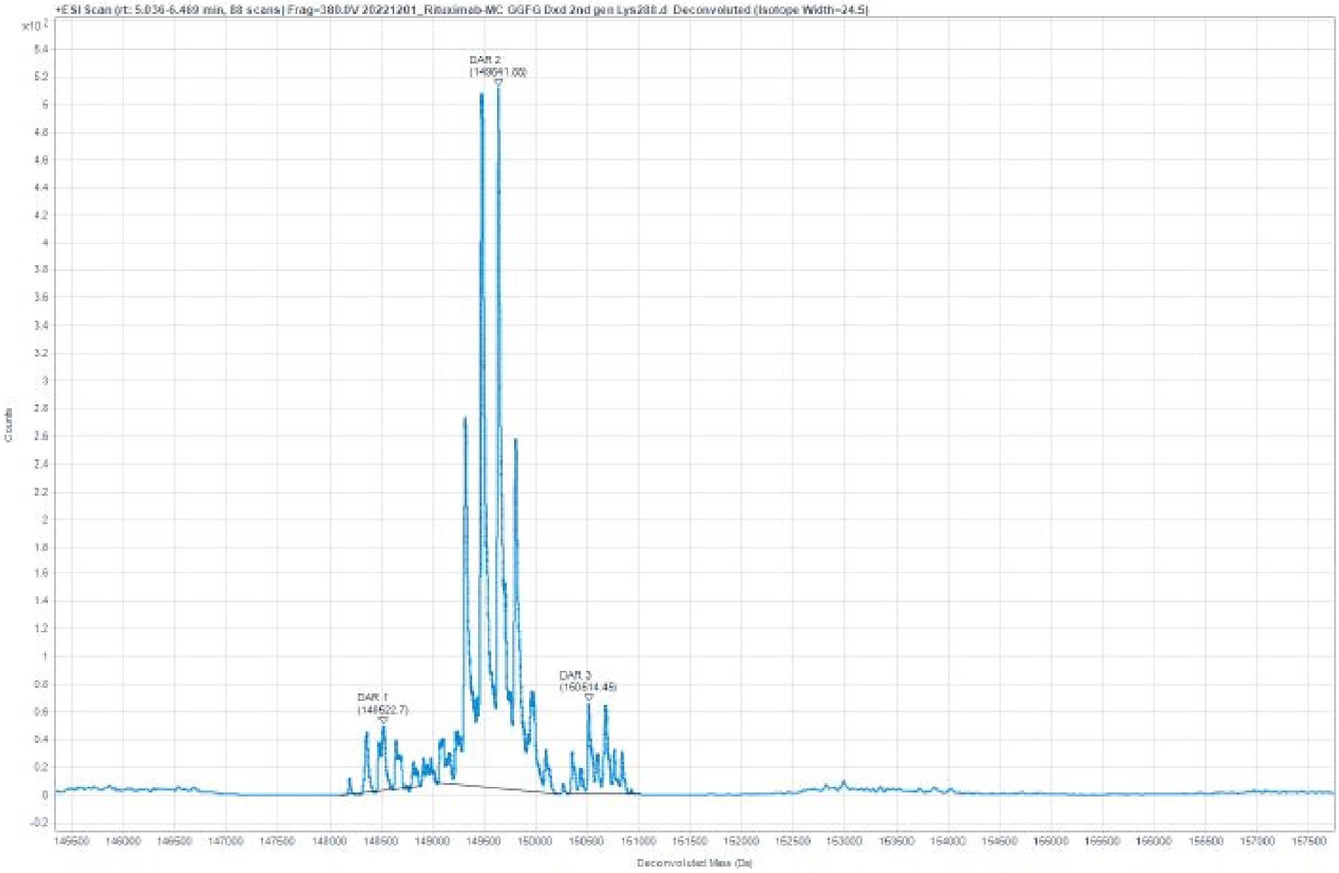

DAR Peak List

DAR Peak	Theoretical Mass (Da)	Observed Mass (Da)	Area	% Area
1	148438	148522.7	7.25E+003	6.24
2	149472	149641.85	9.89E+004	85.15
3	150506	150514.45	1.00E+004	8.61

Figure S~~76~~83. Q-TOF analysis of rituximab-Lys288-Dxd

Average DAR = 2.0

2-5-3 HIC analysis

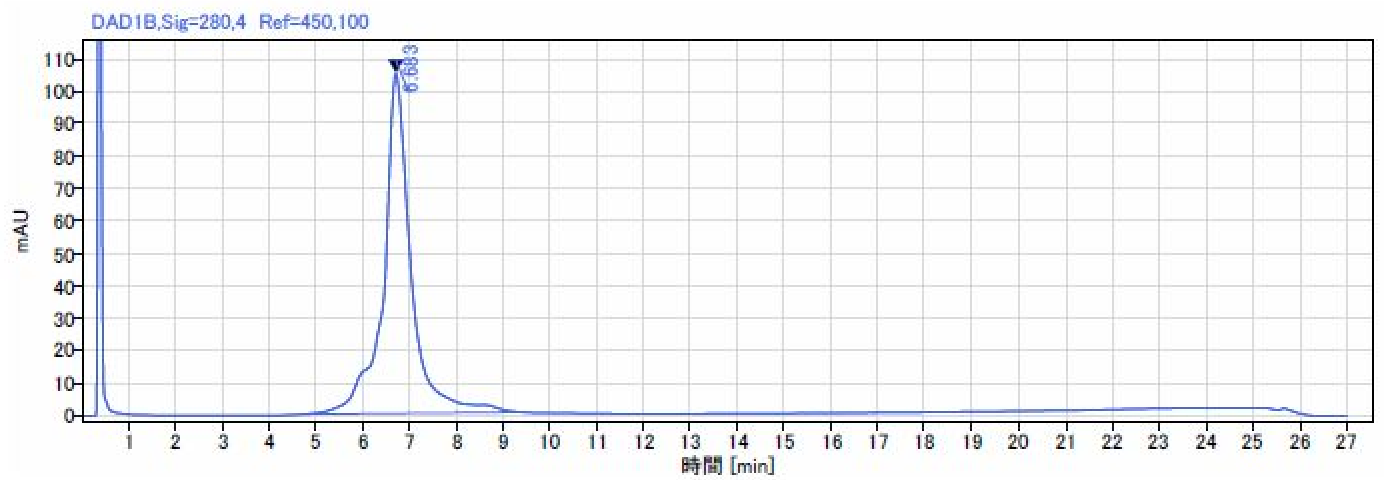

Figure S8477. HIC analysis of trastuzumab-Lys248-maytansinoid

Average DAR = 1.9

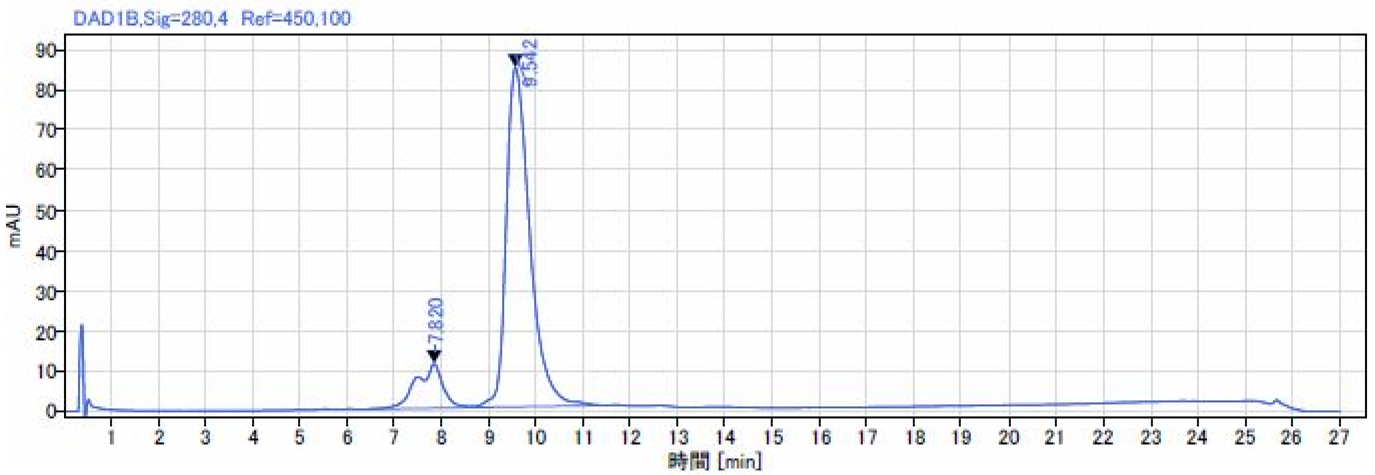

Figure S7885. HIC analysis of trastuzumab-Lys248-MMAE

Average DAR = 1.9

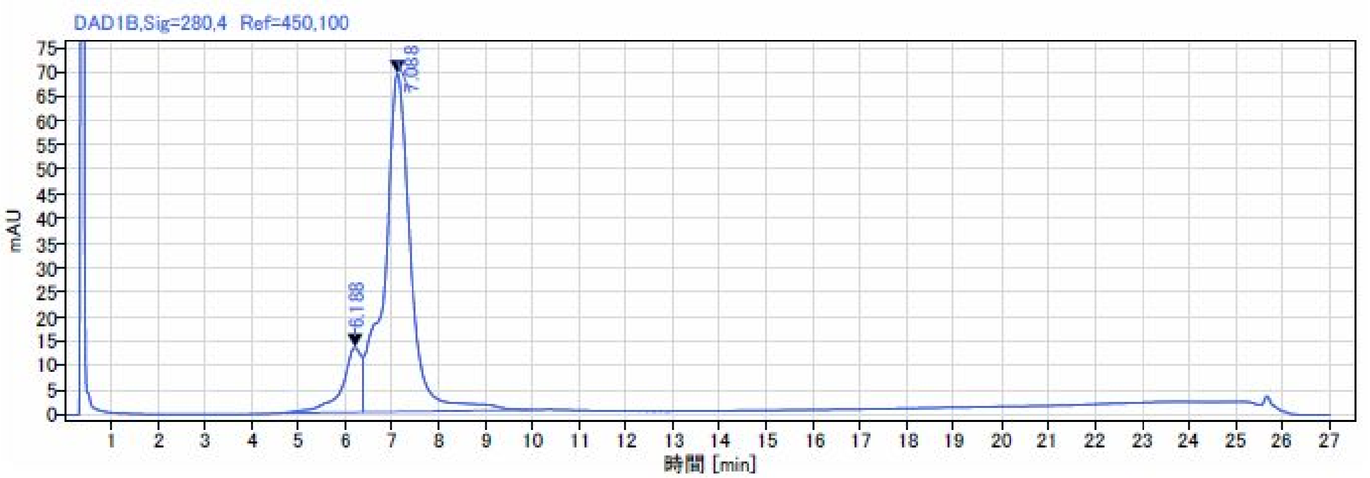

Figure S86~~79~~. HIC analysis of trastuzumab-Lys248-MMAF

Average DAR = 1.9

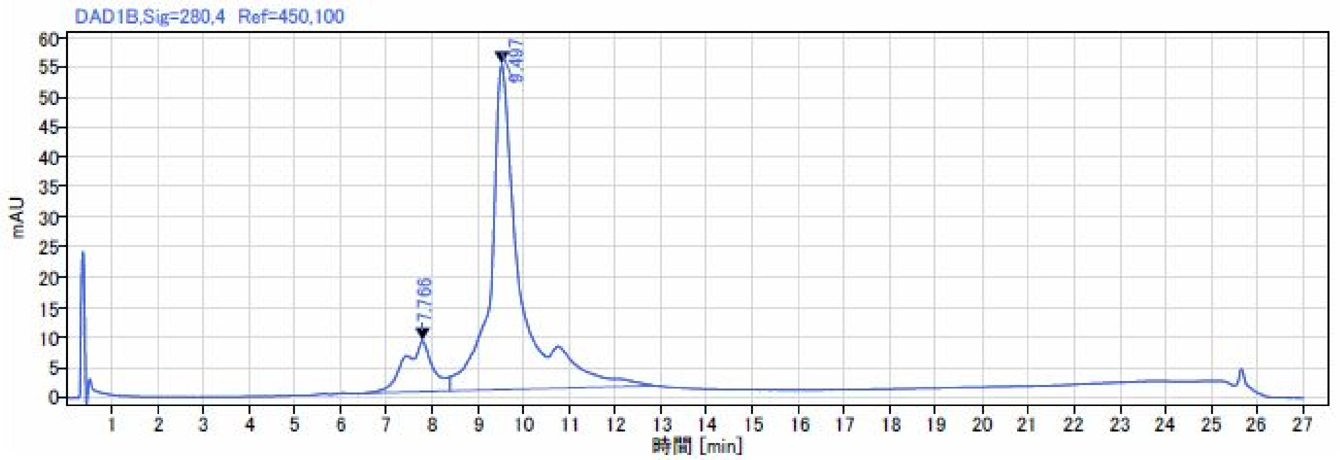

Figure S87~~0~~. HIC analysis of trastuzumab-Lys248-tesirine

Average DAR = 1.9

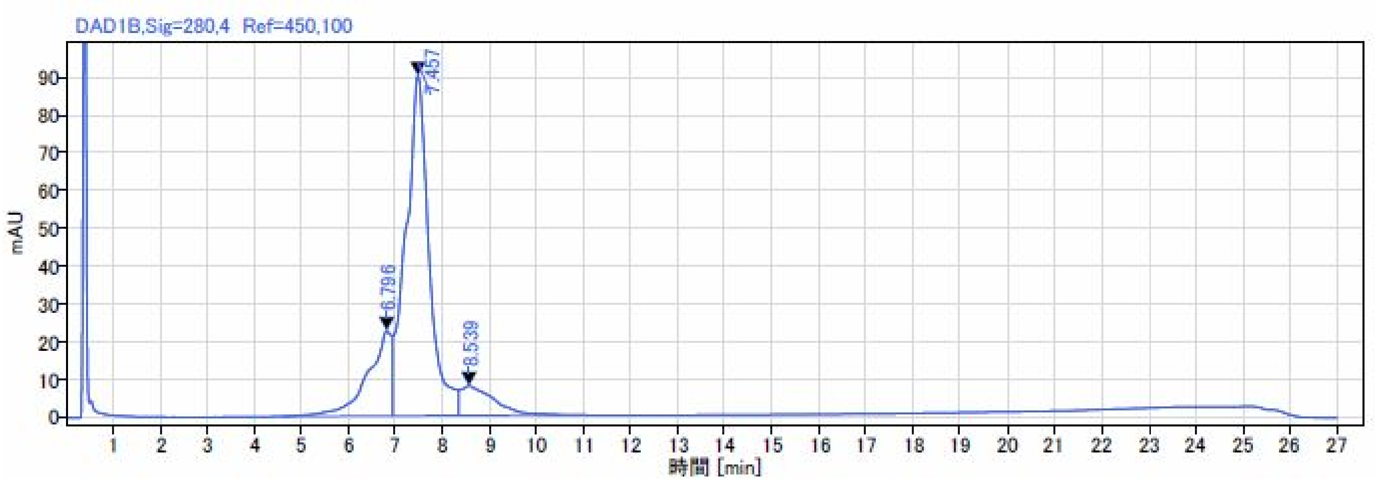

Figure S818. HIC analysis of trastuzumab-Lys248-Dxd

Average DAR = 1.9

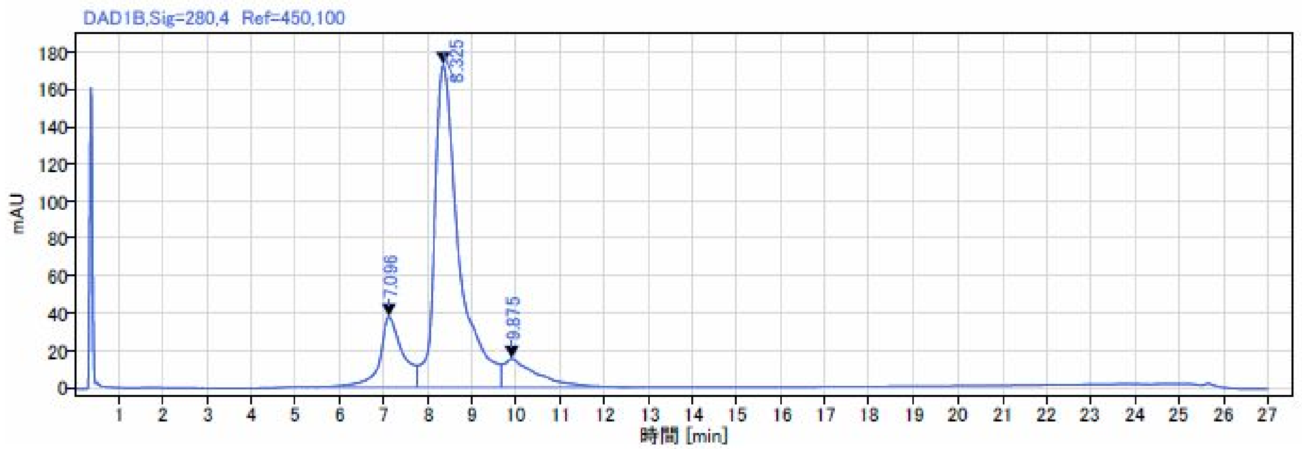

Figure S892. HIC analysis of trastuzumab-Lys288-maytansinoid

Average DAR = 1.9

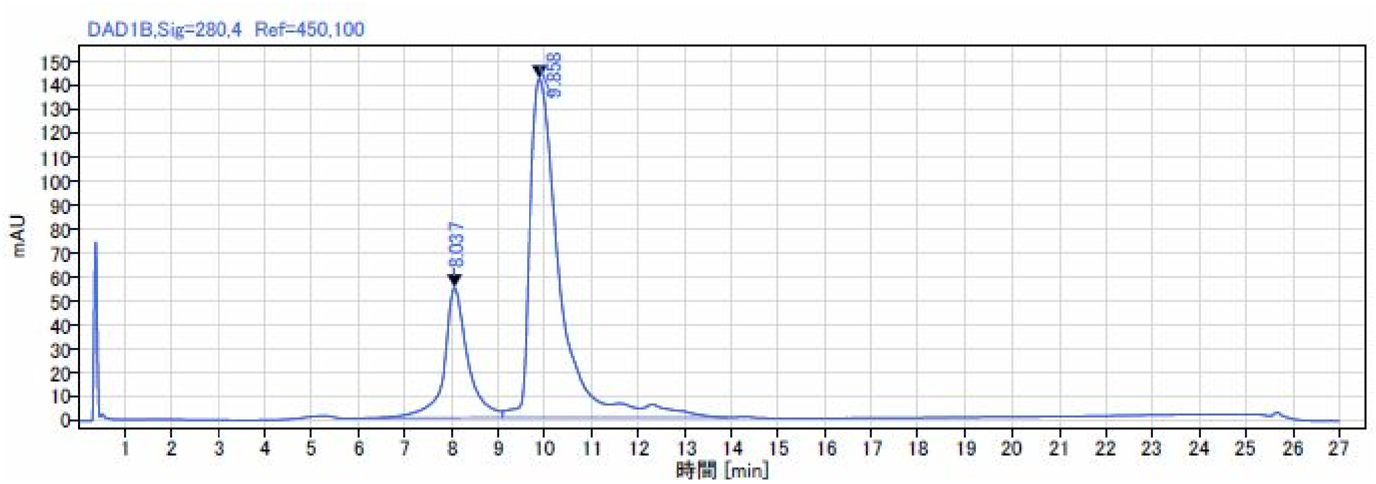

Figure S8390. HIC analysis of trastuzumab-Lys288-MMAE

Average DAR = 1.8

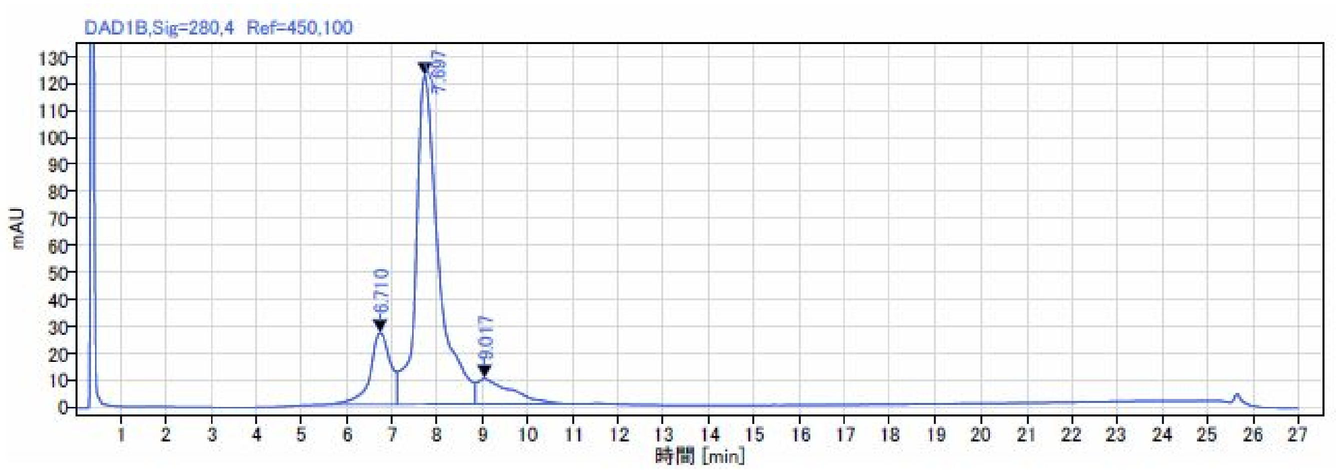

Figure S9184. HIC analysis of trastuzumab-Lys288-MMAF

Average DAR = 1.9

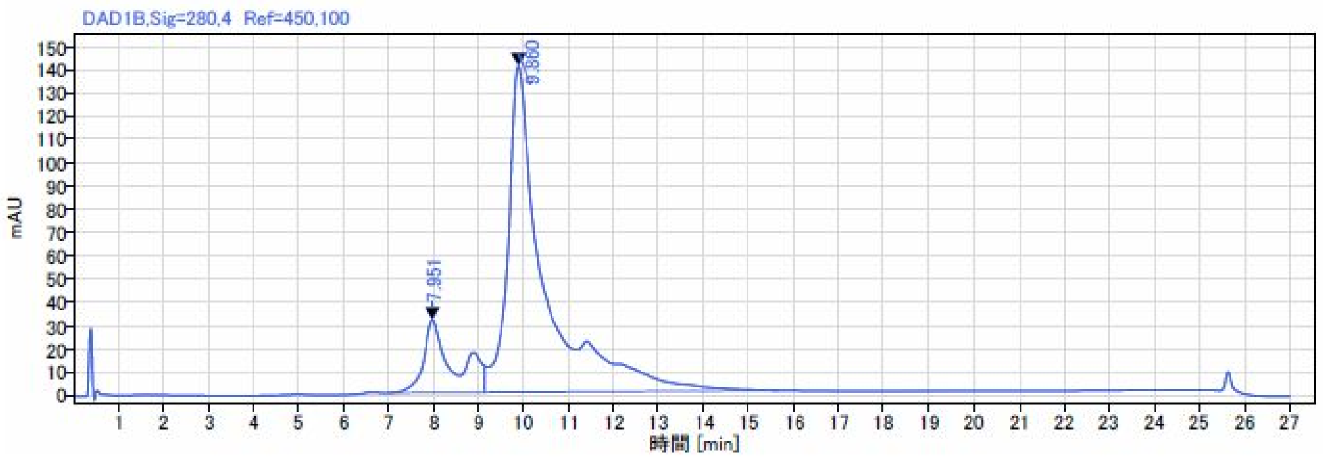

Figure S92~~85~~. HIC analysis of trastuzumab-Lys288-teirine

Average DAR = 1.9

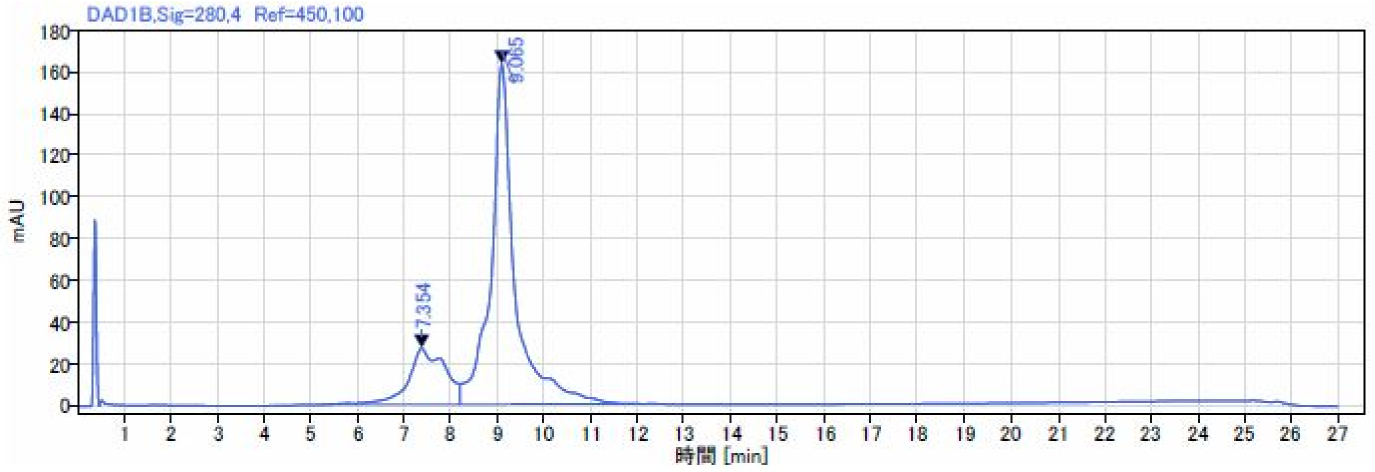

Figure S~~86~~93. HIC analysis of trastuzumab-Lys288-Dxd

Average DAR = 1.8

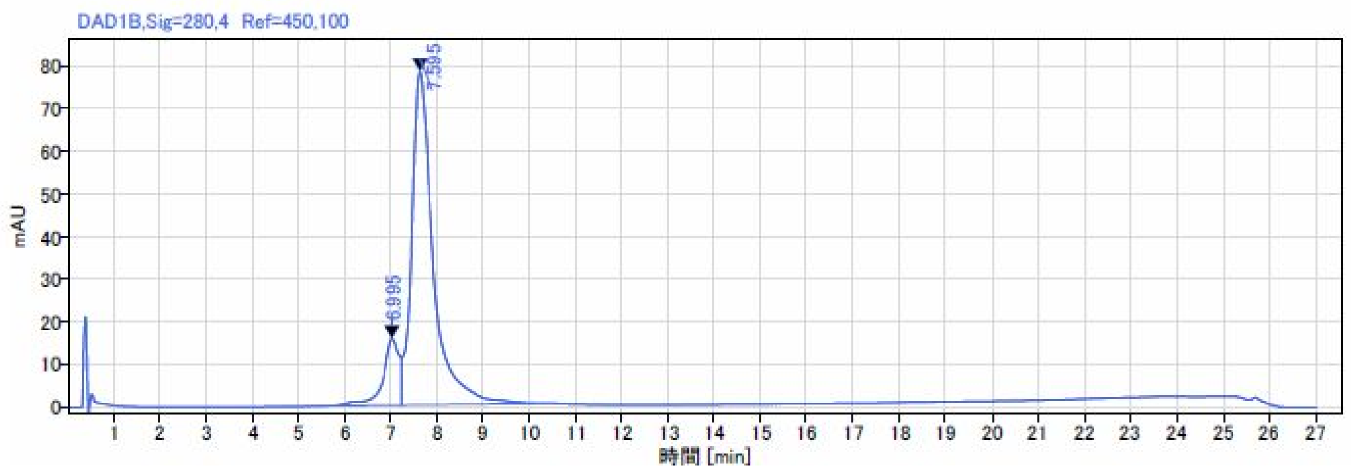

Figure S9487. HIC analysis of rituximab-Lys248-maytansinoid

Average DAR = 1.9

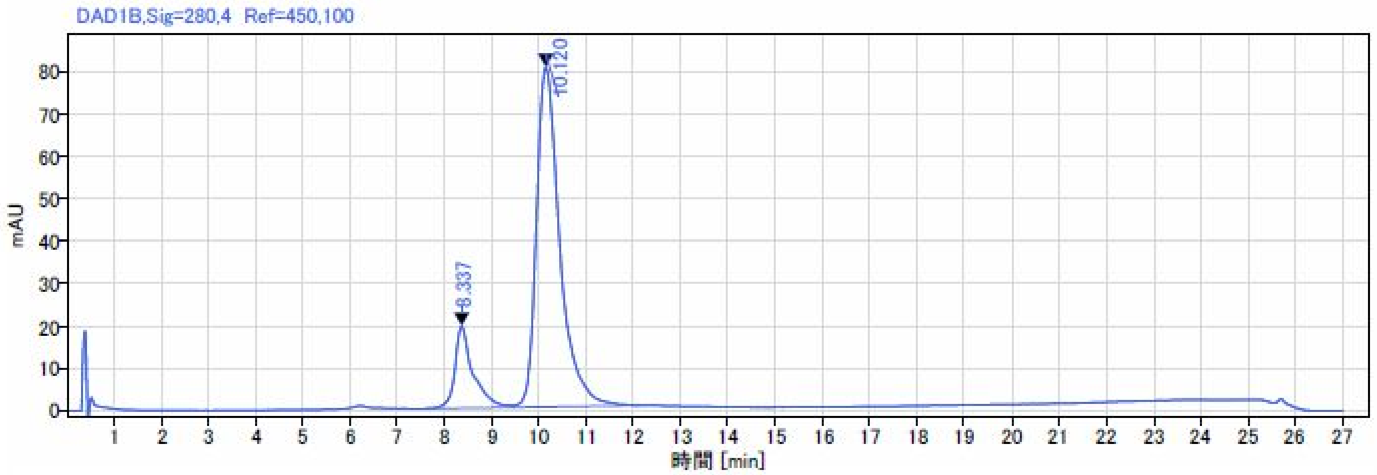

Figure S8895. HIC analysis of rituximab-Lys248-MMAE

Average DAR = 1.9

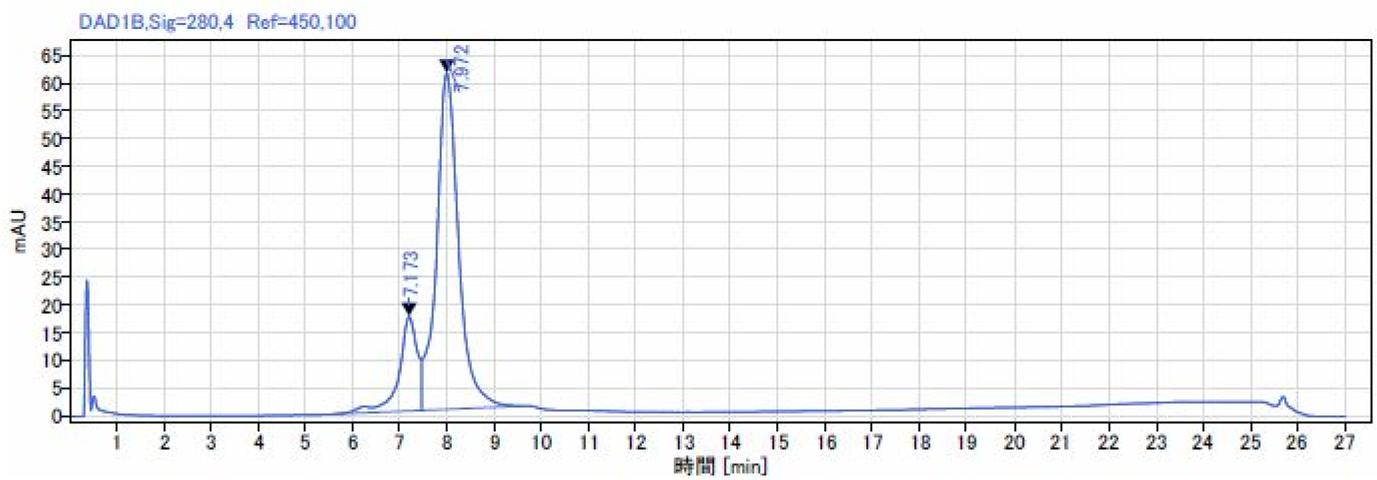

Figure S9689. HIC analysis of rituximab-Lys248-MMAF

Average DAR = 1.8

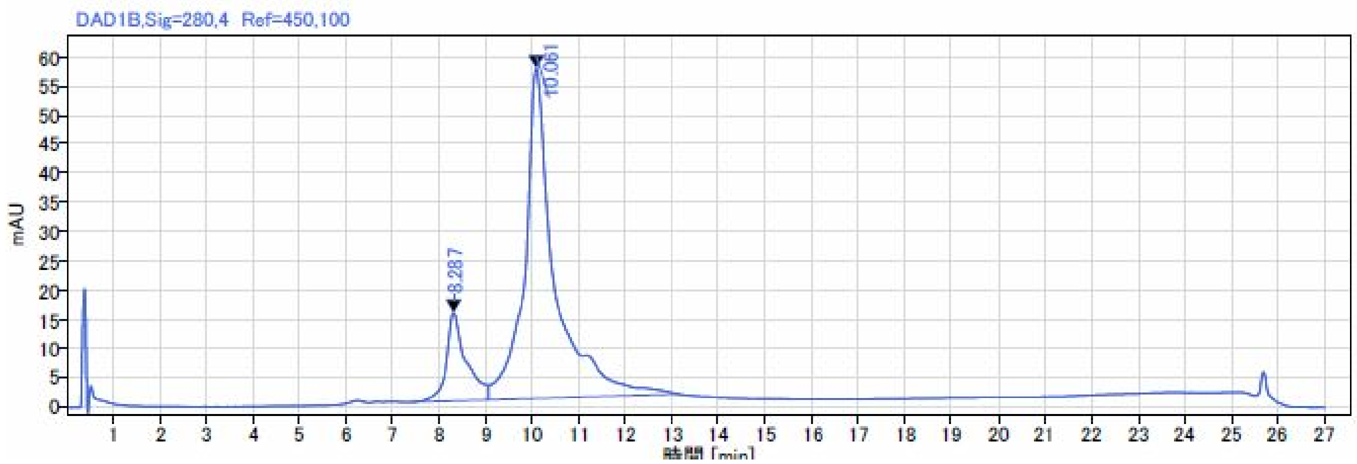

Figure S970. HIC analysis of rituximab-Lys248-tesirine

Average DAR = 1.8

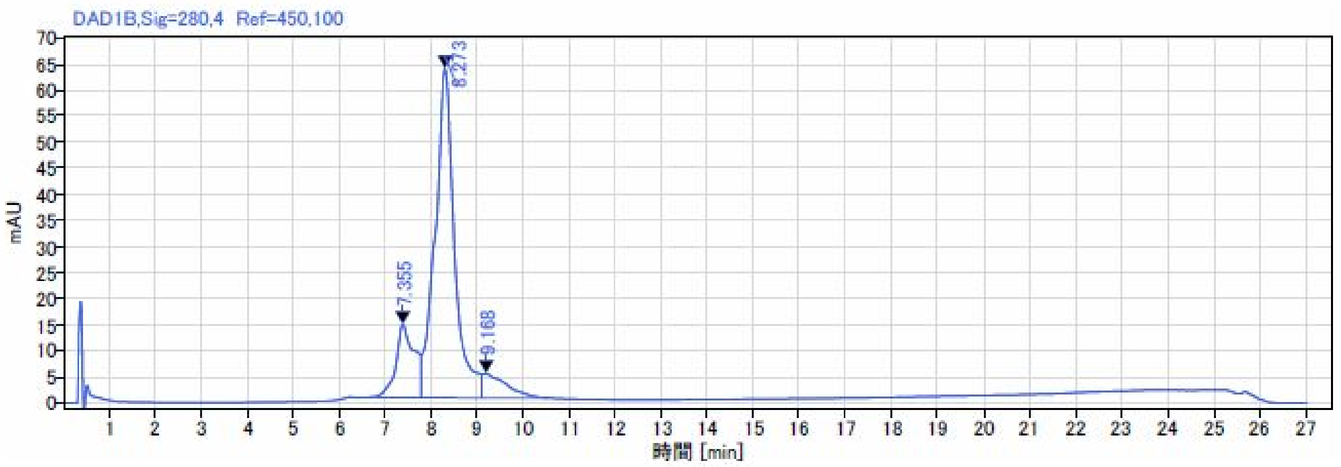

Figure S918. HIC analysis of rituximab-Lys248-Dxd

Average DAR = 1.8

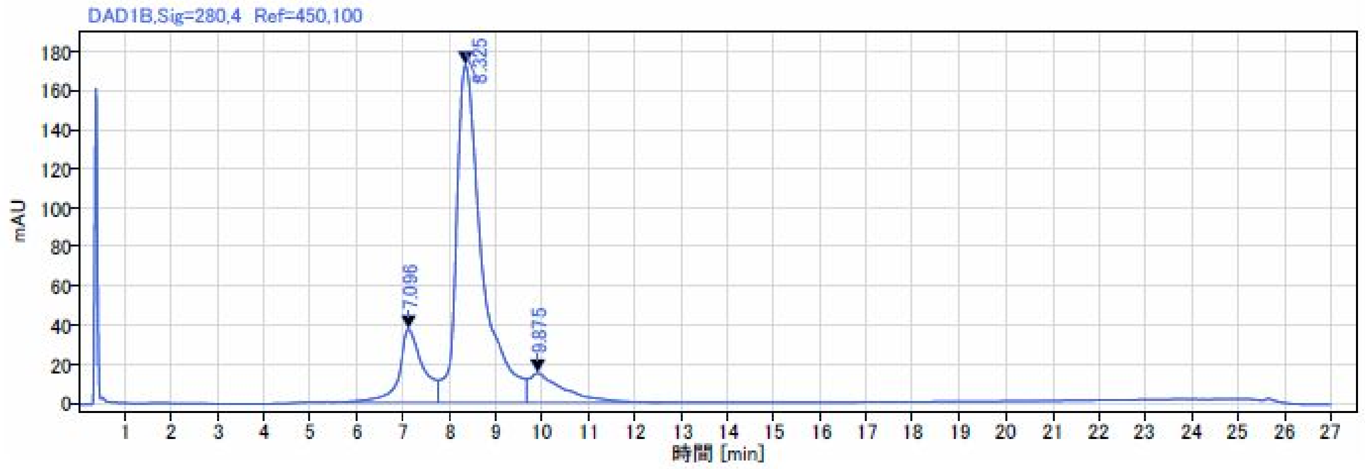

Figure S992. HIC analysis of rituximab-Lys288-maytansinoid

Average DAR = 1.8

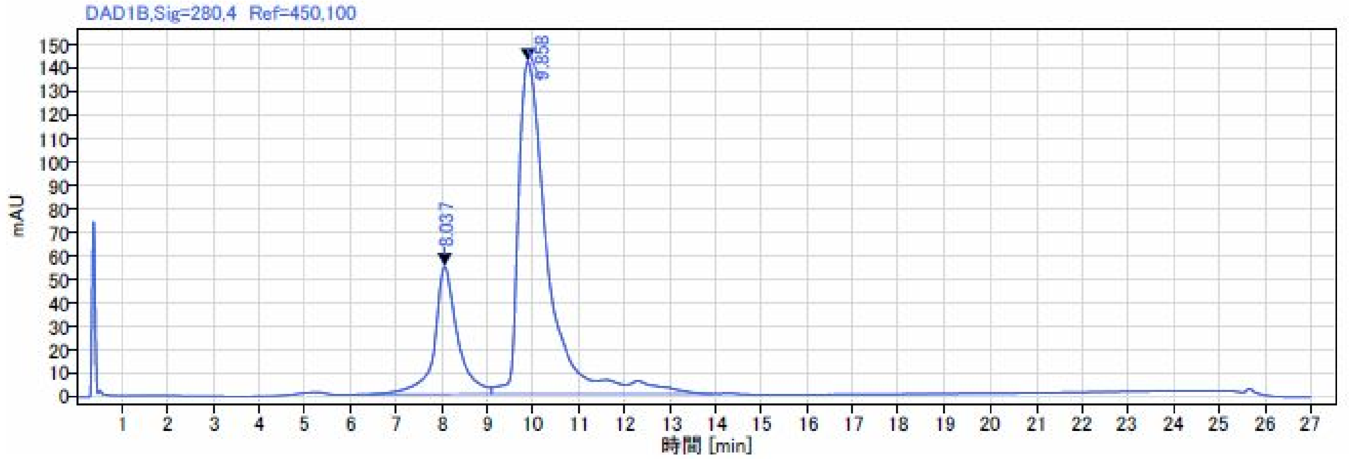

Figure S~~93~~100. HIC analysis of rituximab-Lys288-MMAE

Average DAR = 1.8

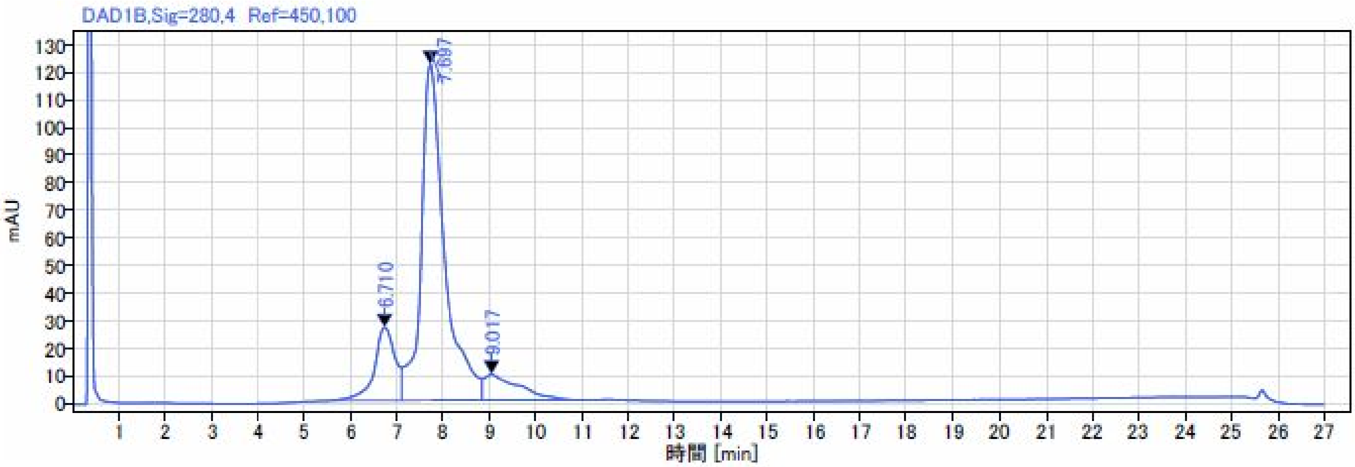

Figure S101~~94~~. HIC analysis of rituximab-Lys288-MMAF

Average DAR = 1.9

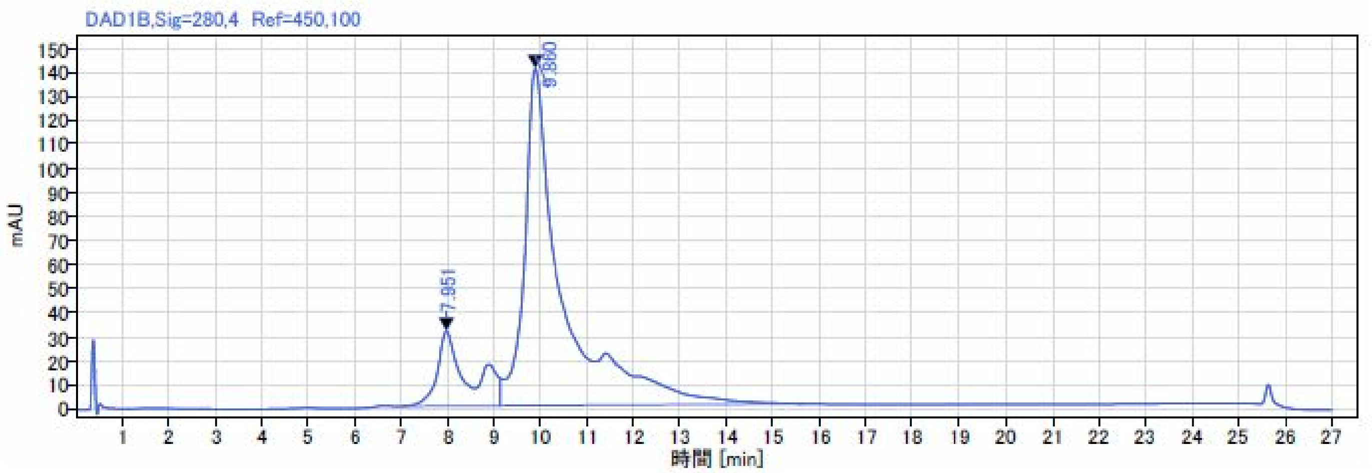

Figure S10295. HIC analysis of rituximab-Lys288-tesirine

Average DAR = 1.9

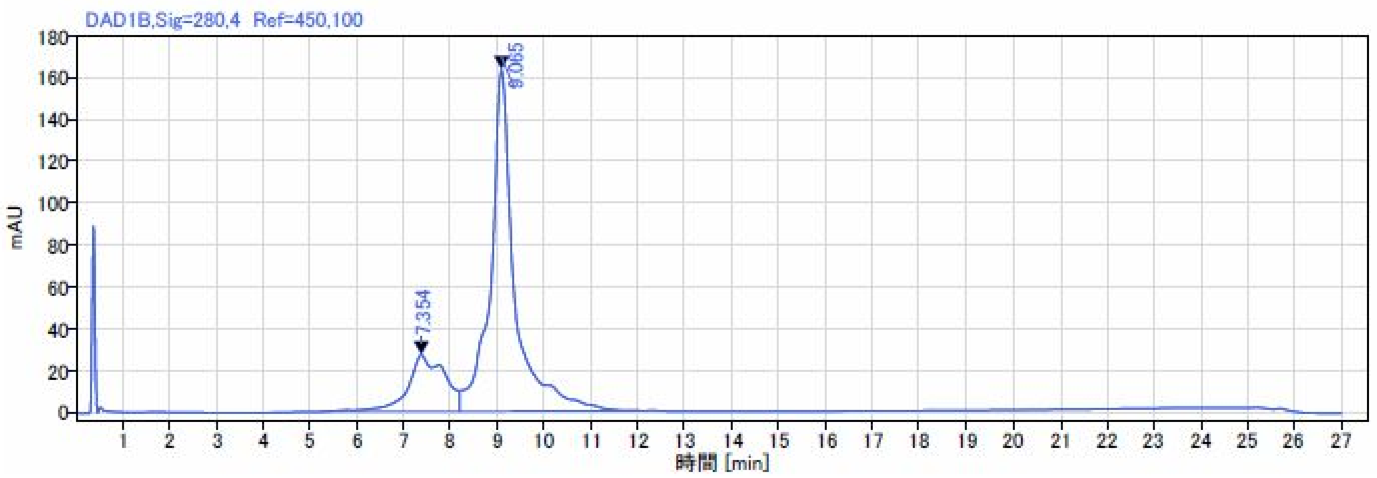

Figure S96103. HIC analysis of rituximab-Lys288-Dxd

Average DAR = 1.8

2-5-4 SEC analysis

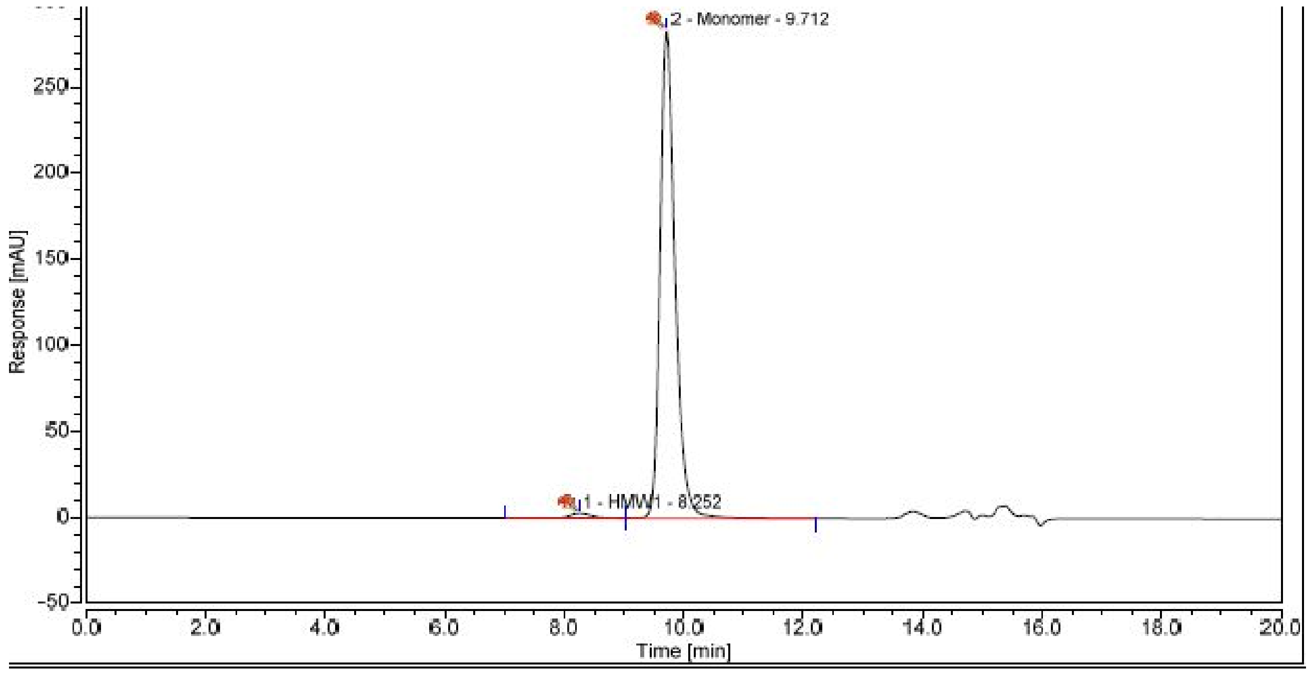

Figure S104~~97~~. SEC analysis of trastuzumab-Lys248-maytansinoid

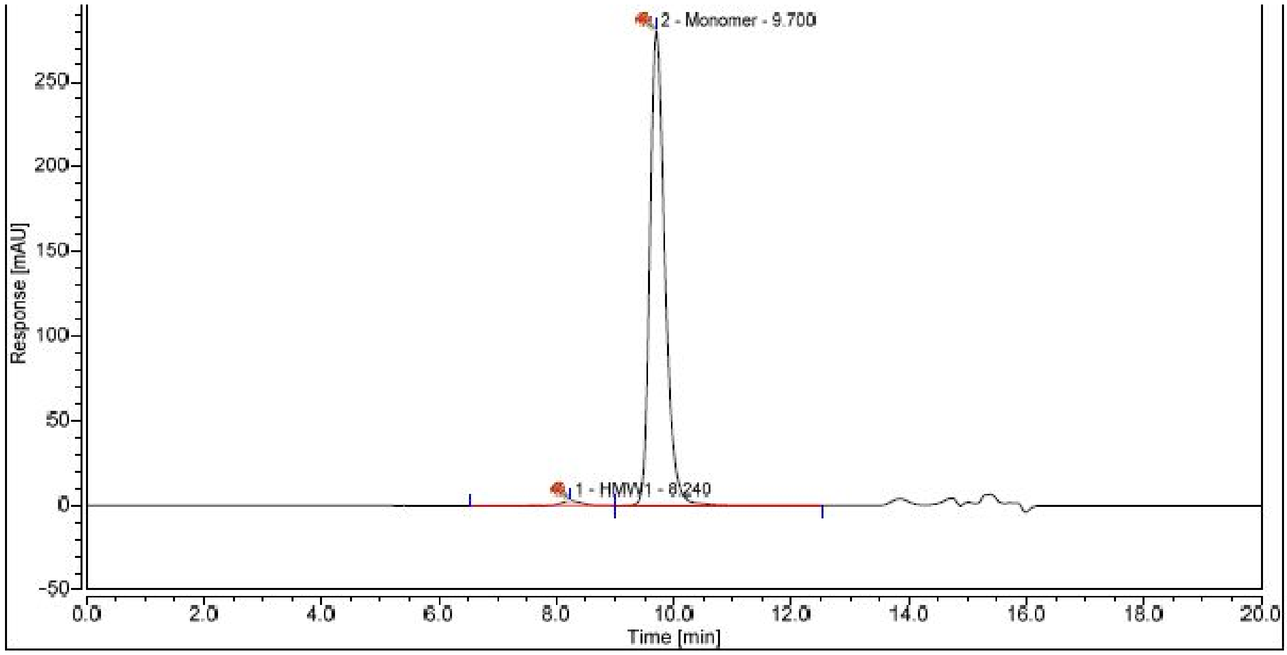

Figure S~~98~~105. SEC analysis of trastuzumab-Lys248-MMAE

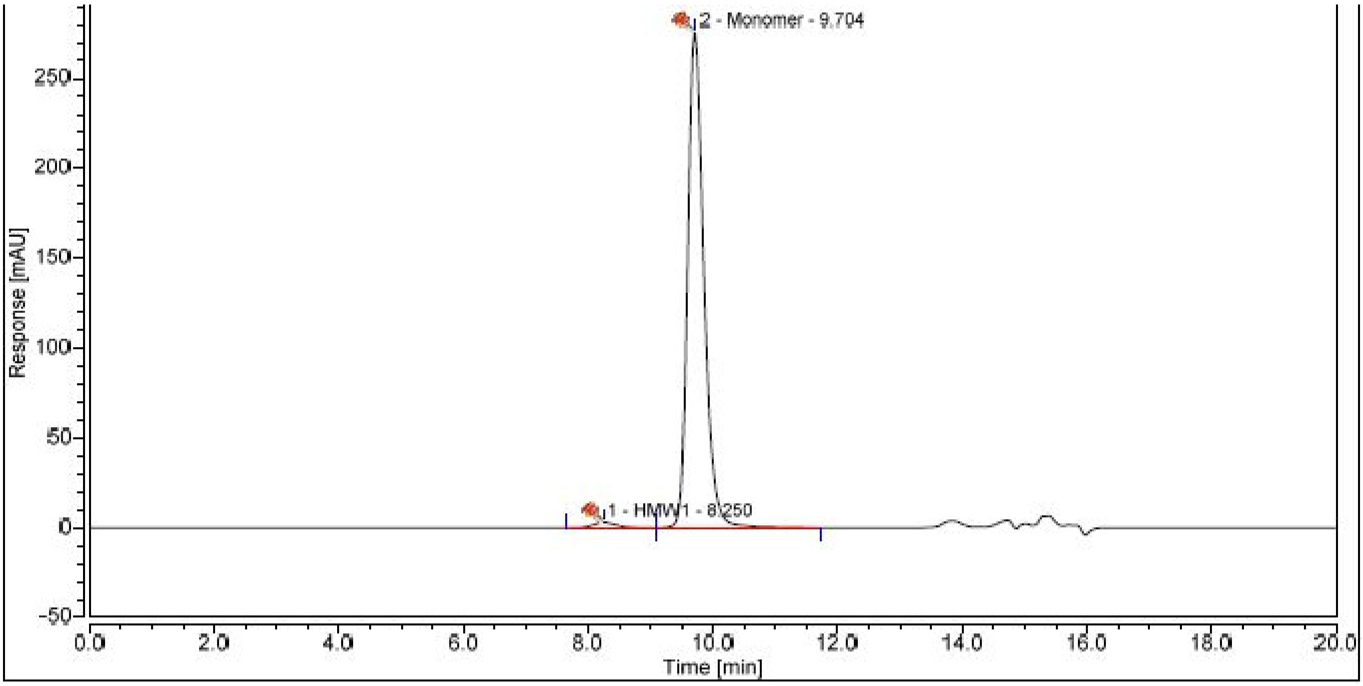

Figure S10699. SEC analysis of trastuzumab-Lys248-MMAF

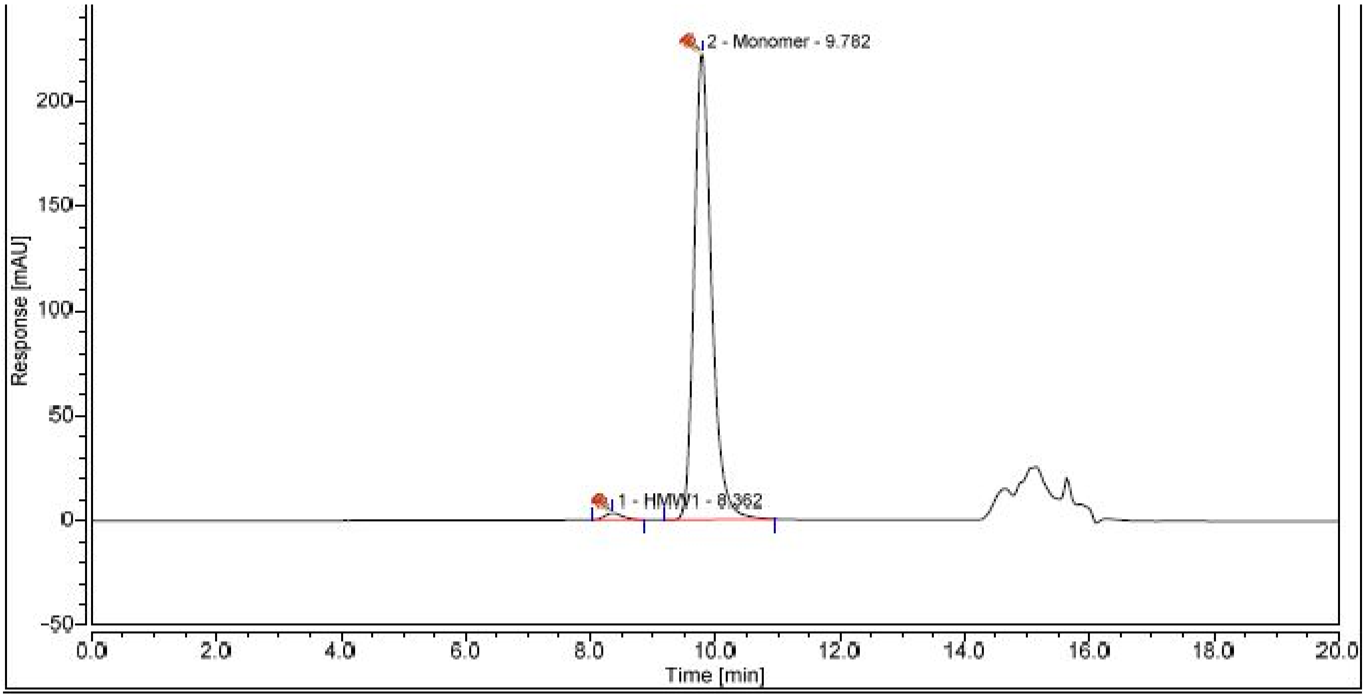

Figure S1070. SEC analysis of trastuzumab-Lys248-tesirine

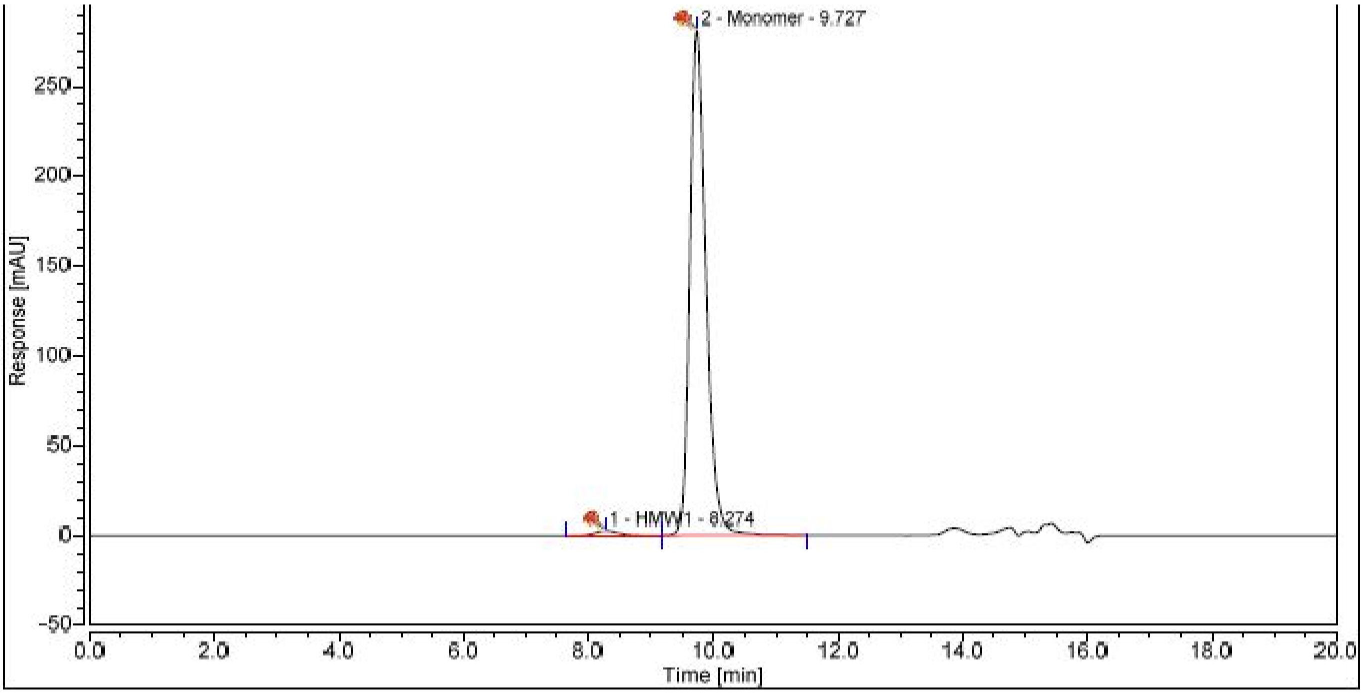

Figure S10~~1~~8. SEC analysis of trastuzumab-Lys248-Dxd

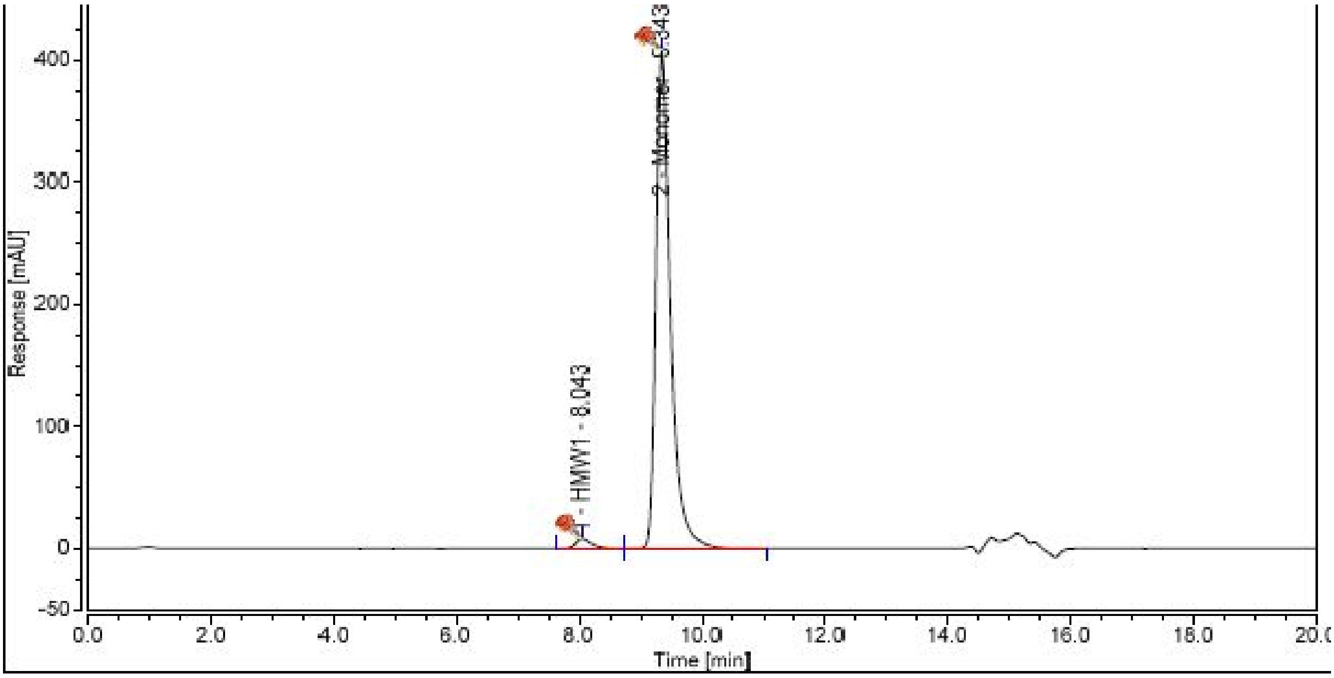

Figure S109~~2~~. SEC analysis of trastuzumab-Lys288-maytansinoid

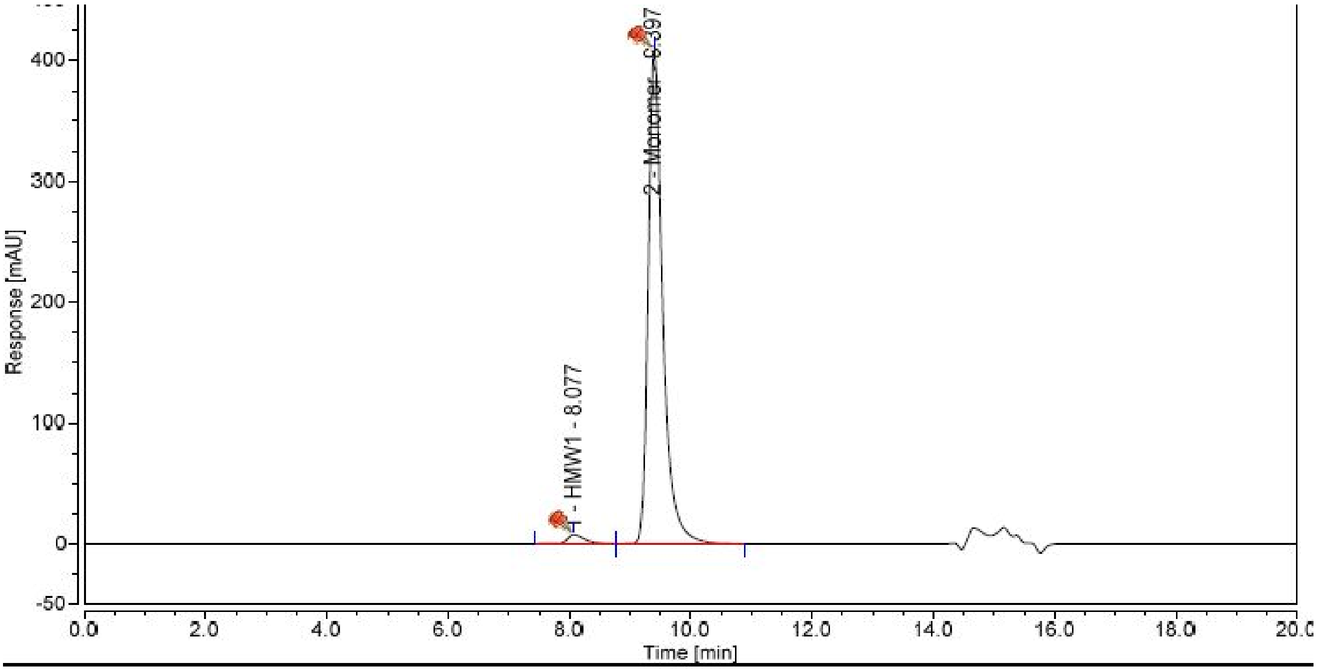

Figure S1~~03~~10. SEC analysis of trastuzumab-Lys288-MMAE

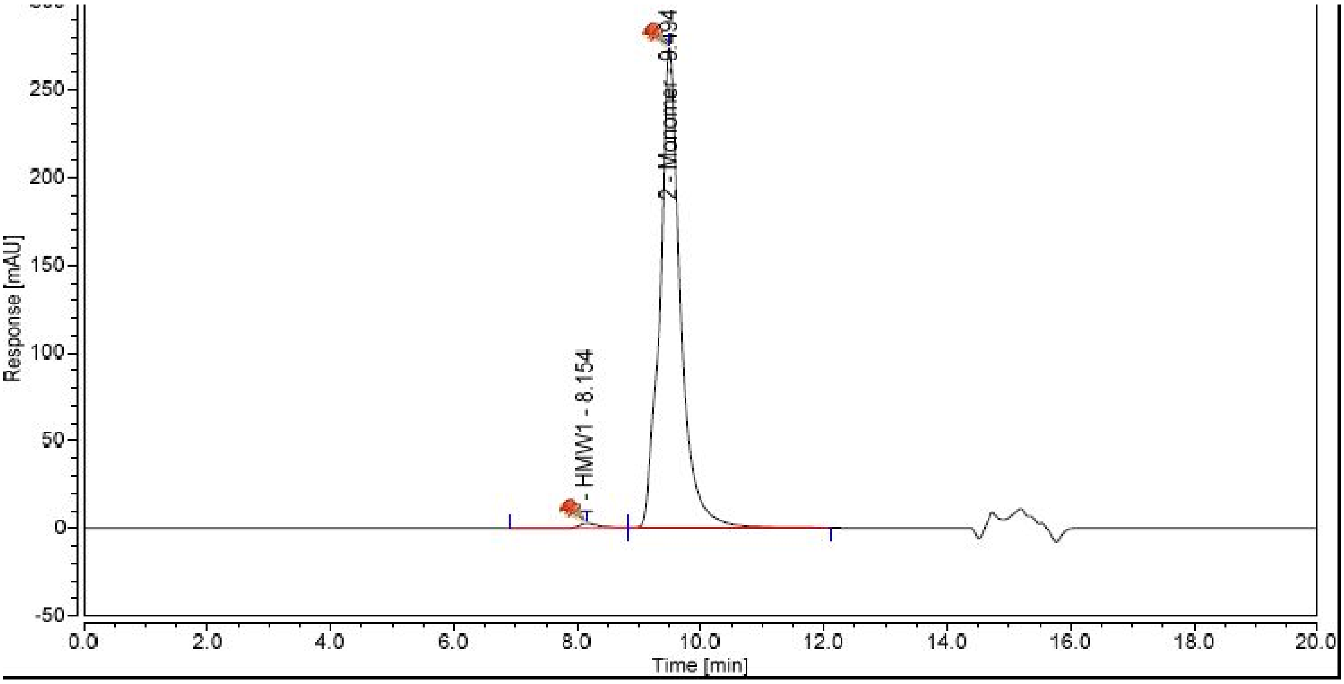

Figure S111~~04~~. SEC analysis of trastuzumab-Lys288-MMAF

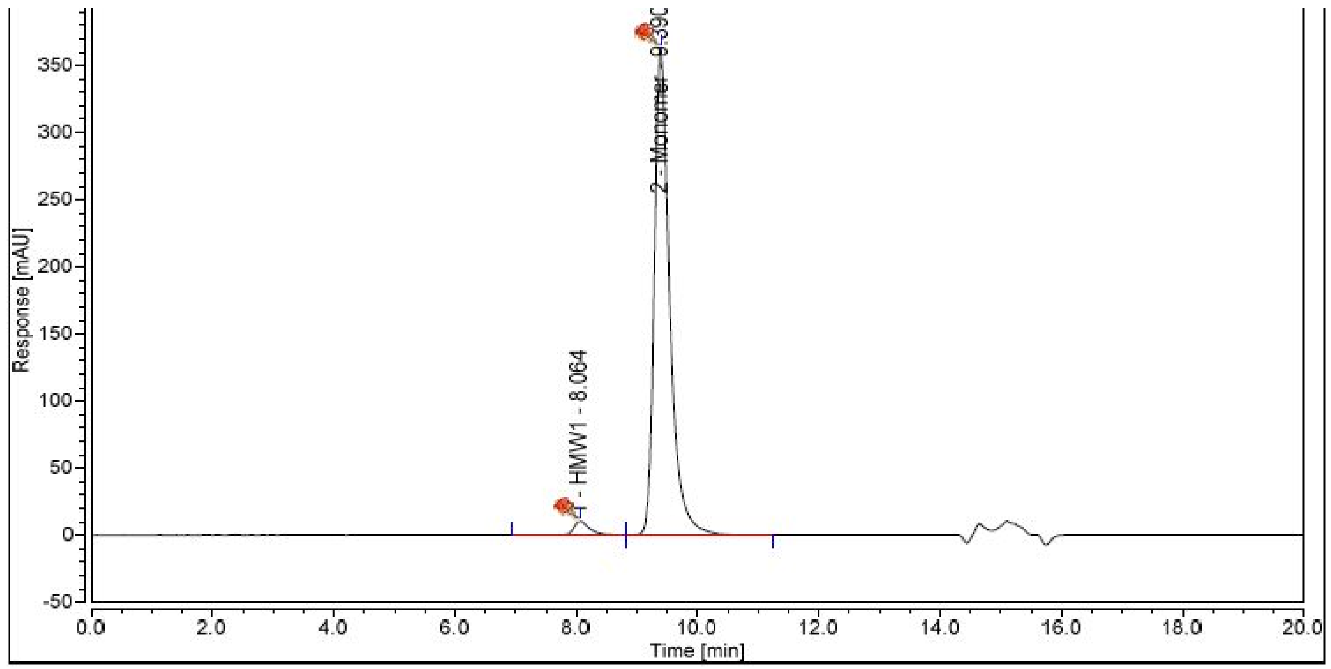

Figure S112~~05~~. SEC analysis of trastuzumab-Lys288-teirine

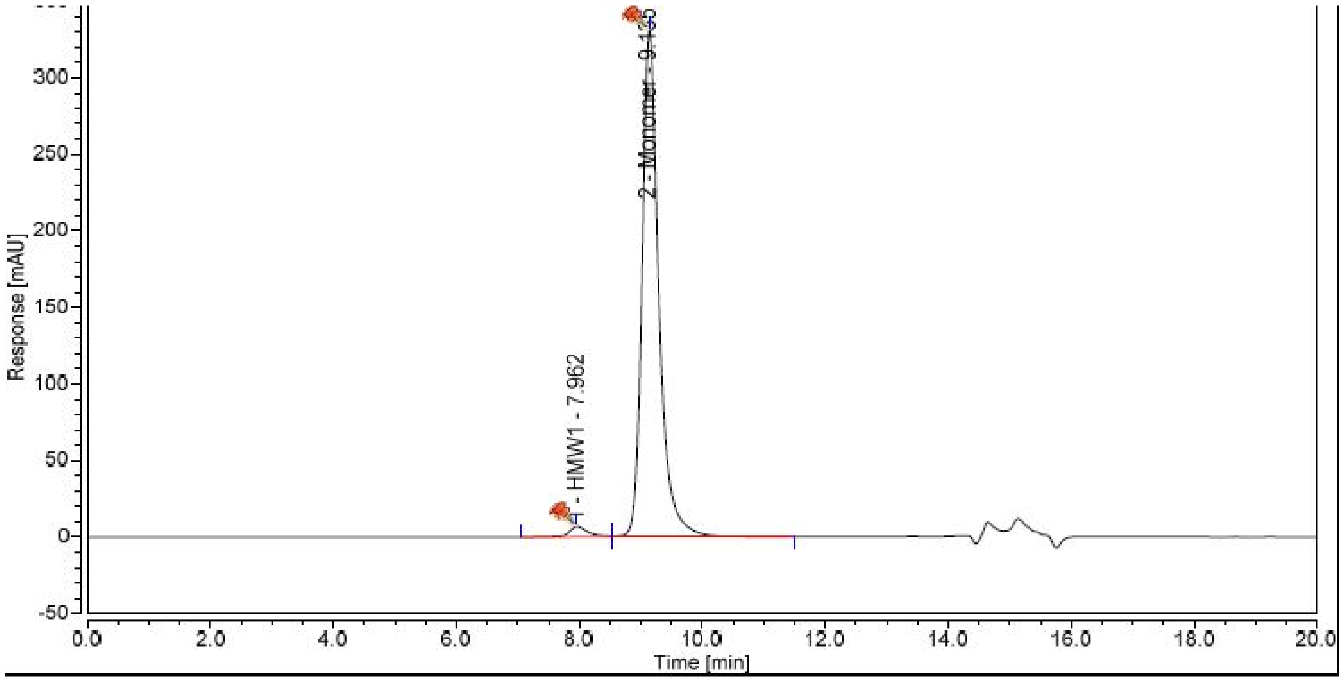

Figure S1~~06~~13. SEC analysis of trastuzumab-Lys288-Dxd

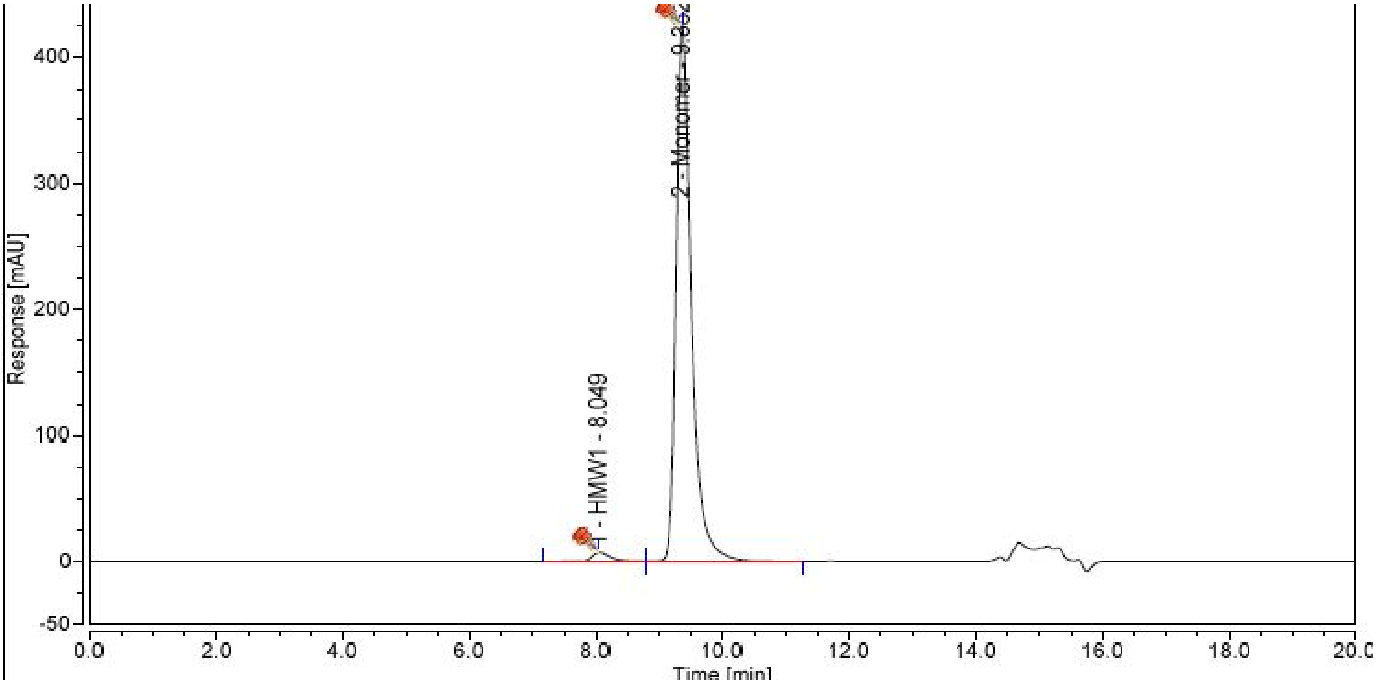

Figure S114~~07~~. SEC analysis of rituximab-Lys248-maytansinoid

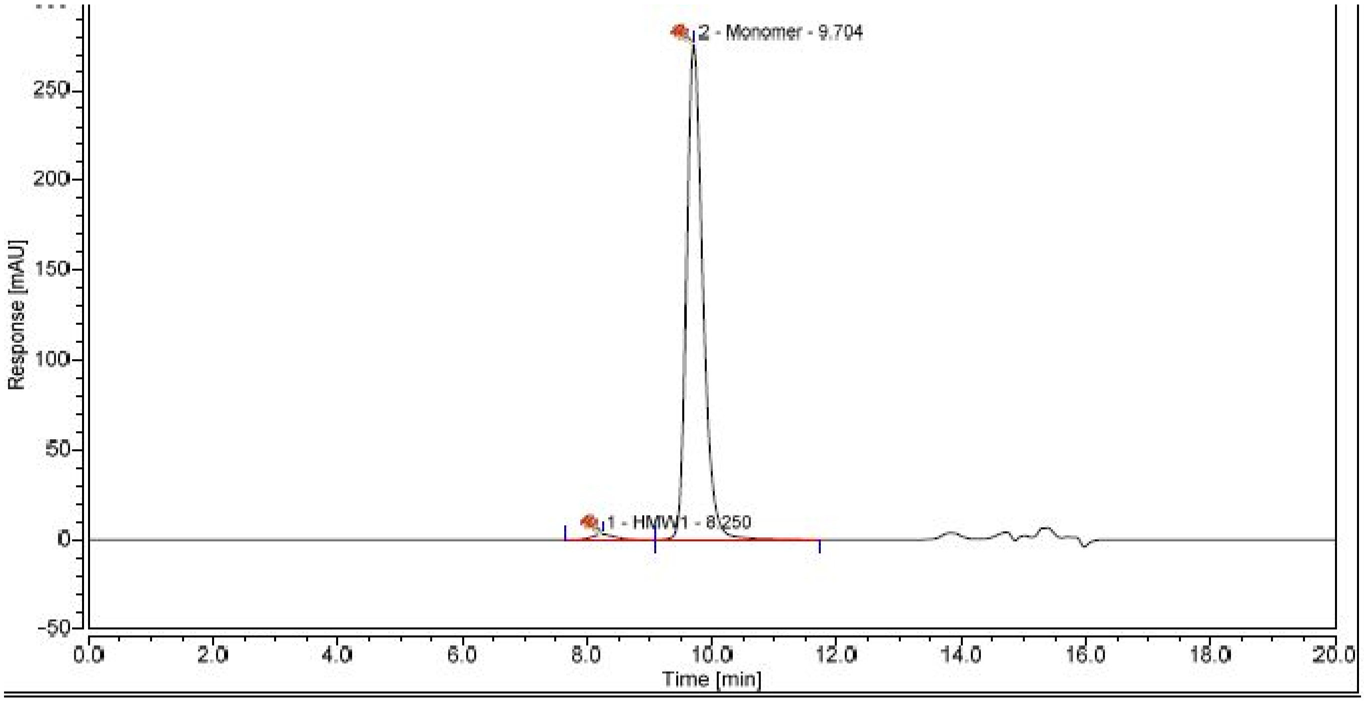

Figure S1~~08~~15. SEC analysis of rituximab-Lys248-MMAE

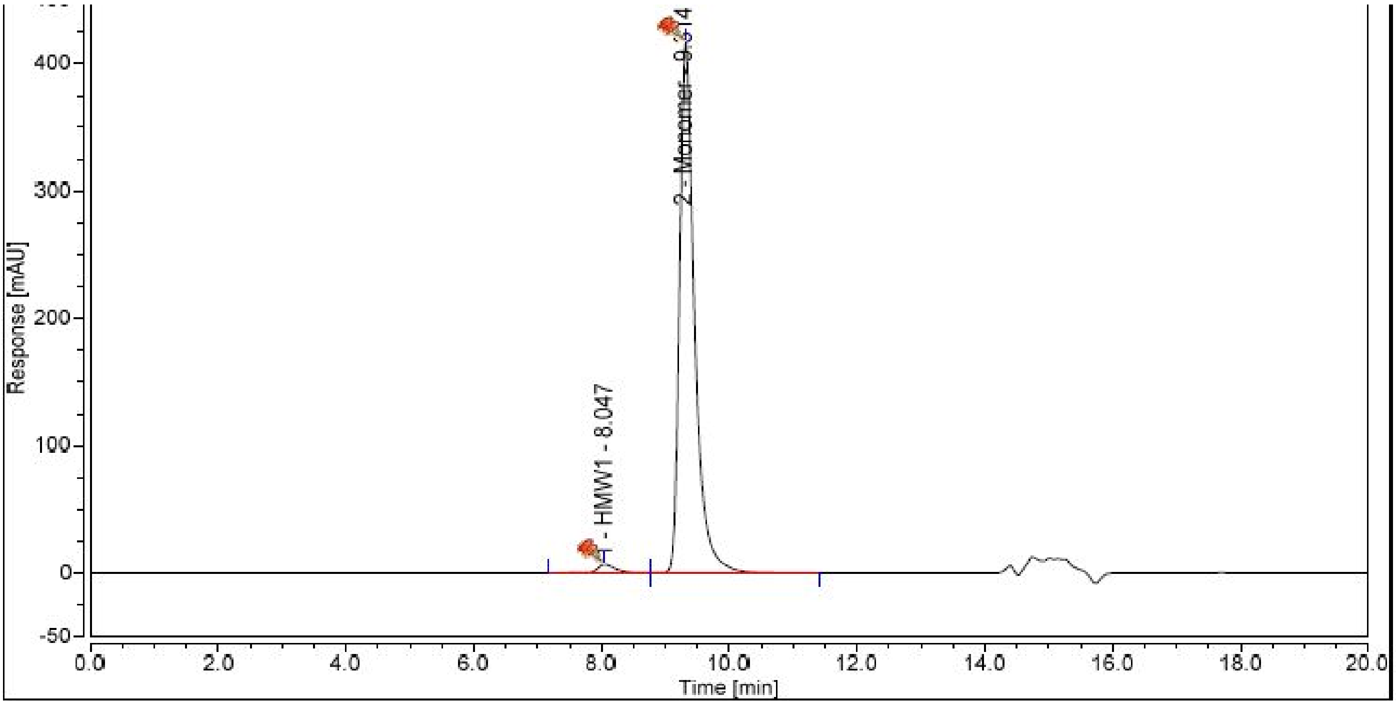

Figure S11609. SEC analysis of rituximab-Lys248-MMAF

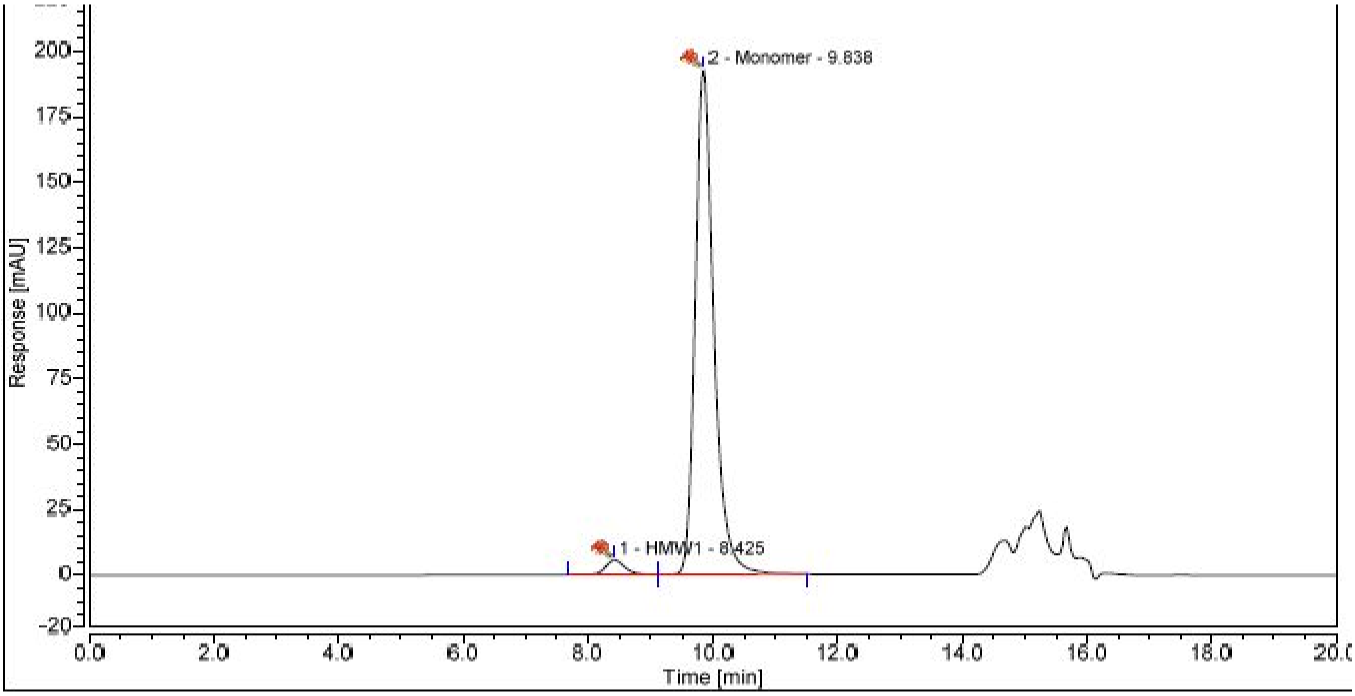

Figure S1170. SEC analysis of rituximab-Lys248-tesirine

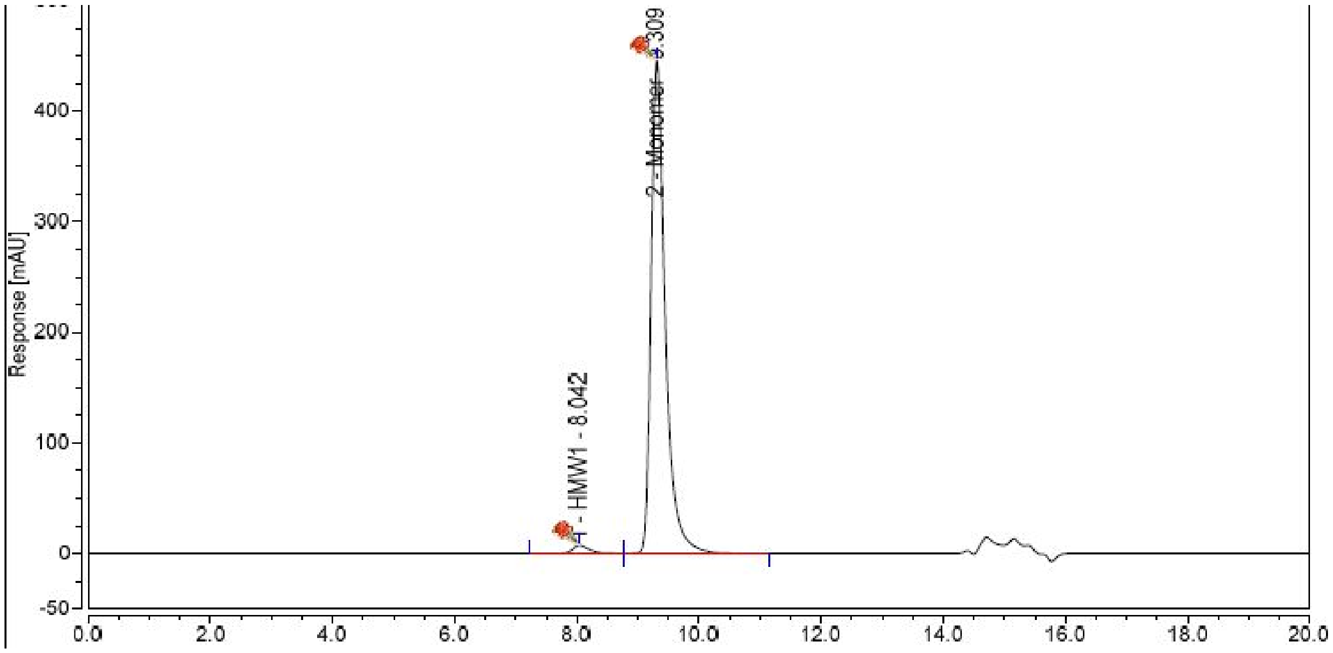

Figure S11~~1~~8. SEC analysis of rituximab-Lys248-Dxd

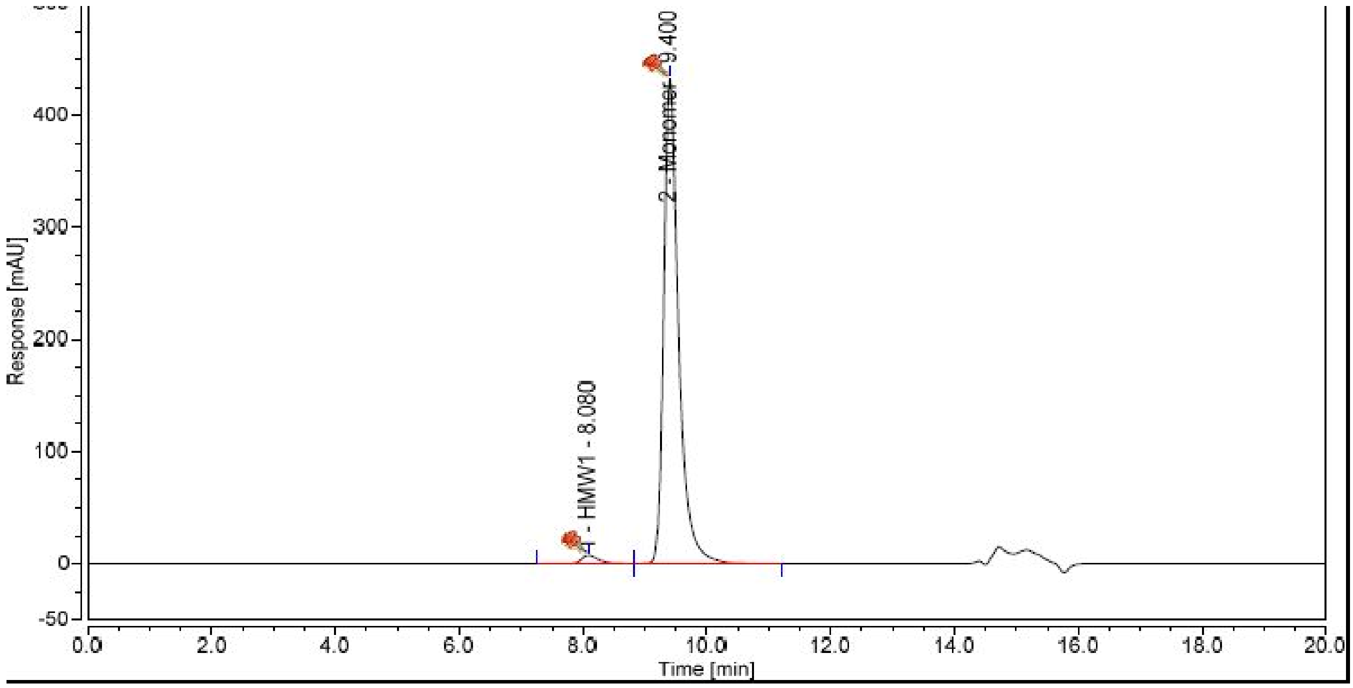

Figure S119~~2~~. SEC analysis of rituximab-Lys288-maytansinoid

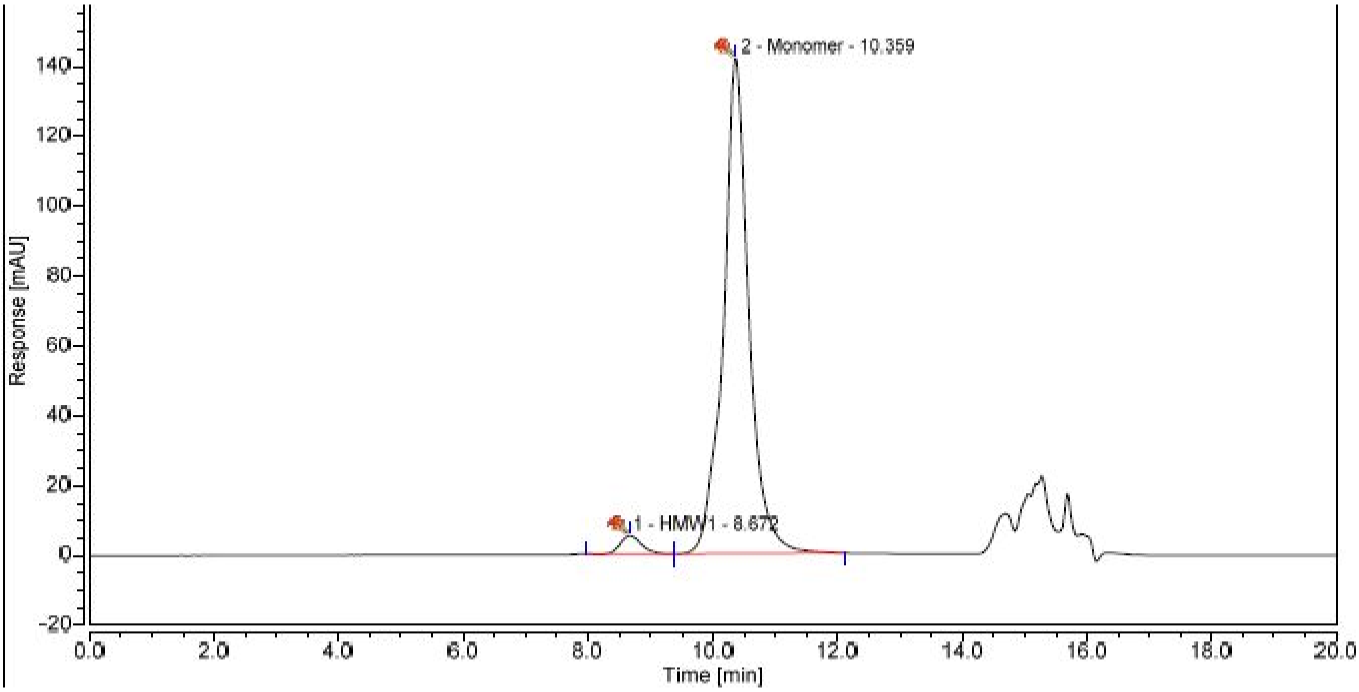

Figure S120~~13~~. SEC analysis of rituximab-Lys288-MMAE

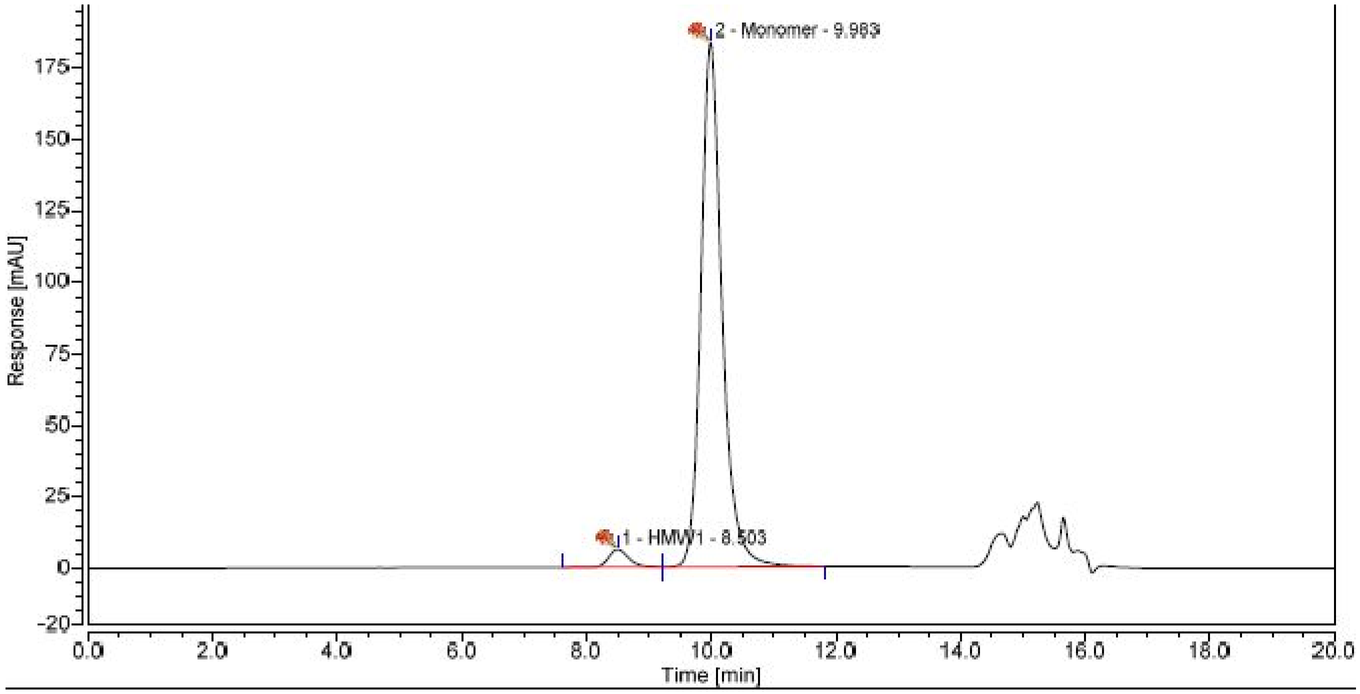

Figure S121~~14~~. SEC analysis of rituximab-Lys288-MMAF

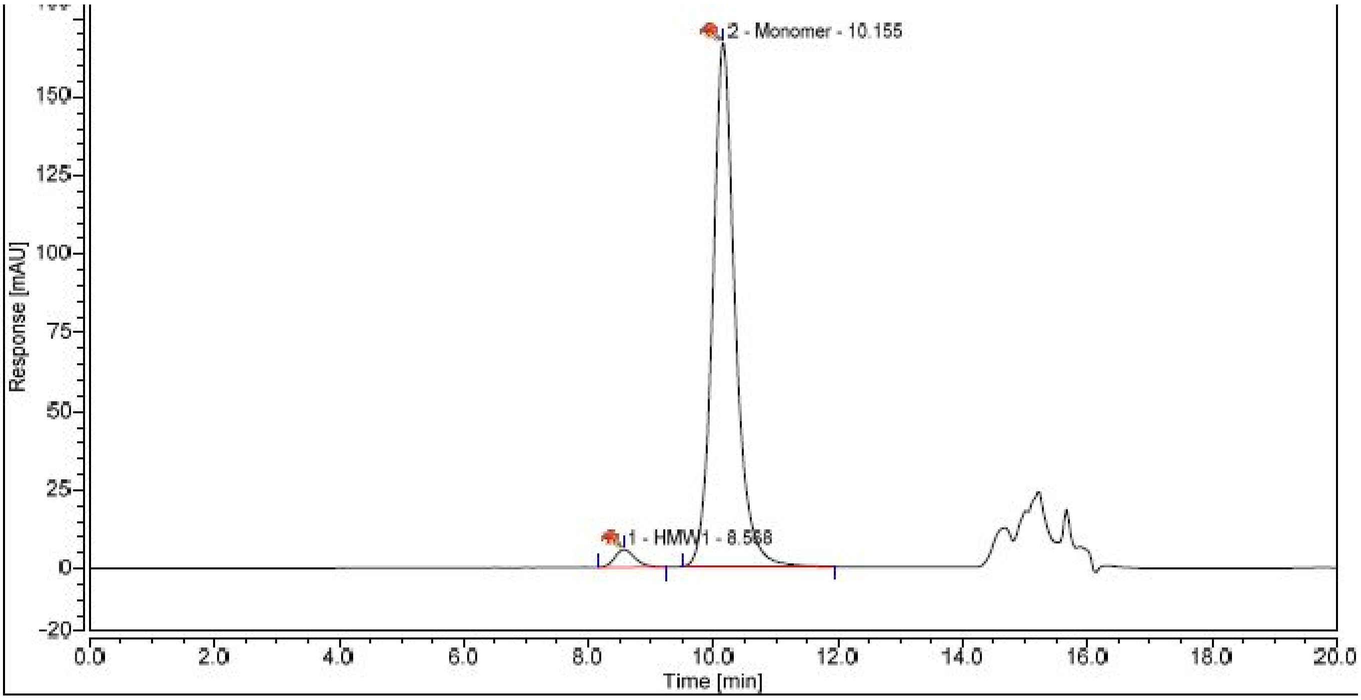

Figure S122~~15~~. SEC analysis of rituximab-Lys288-tesirine

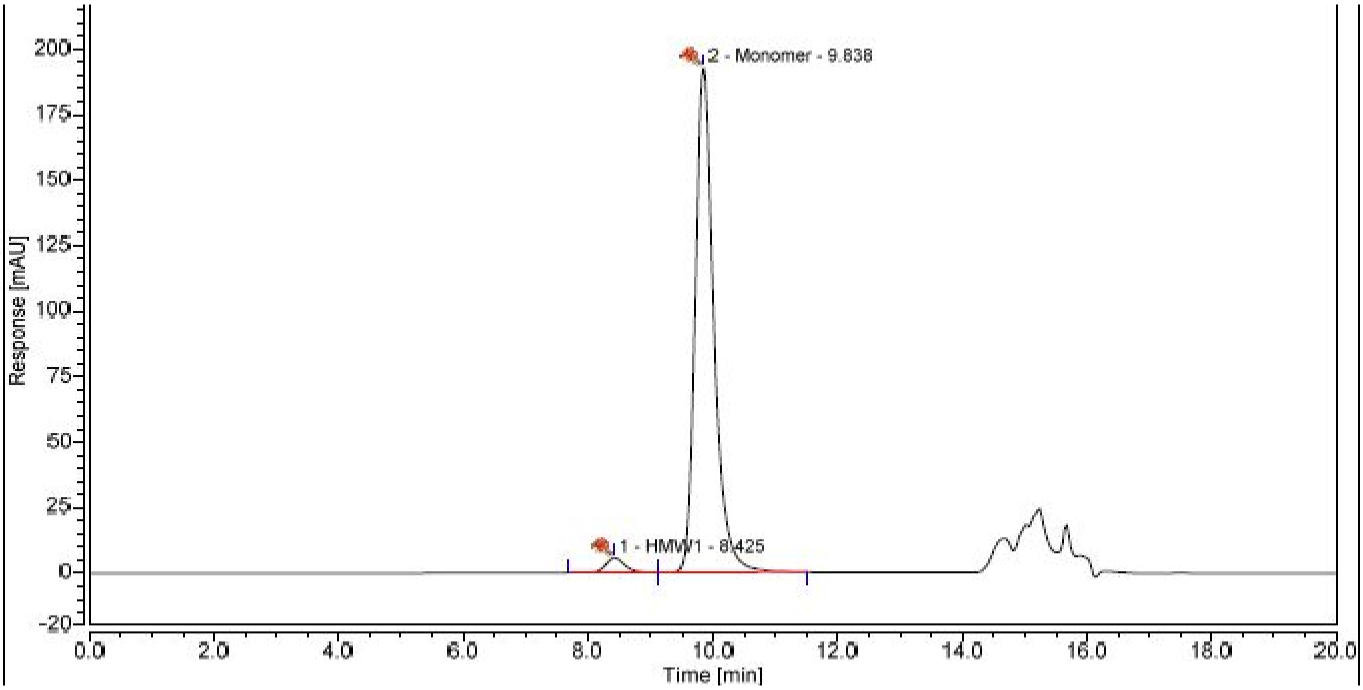

Figure S1~~16~~23. SEC analysis of rituximab-Lys288-Dxd

2-6 Peptide mapping

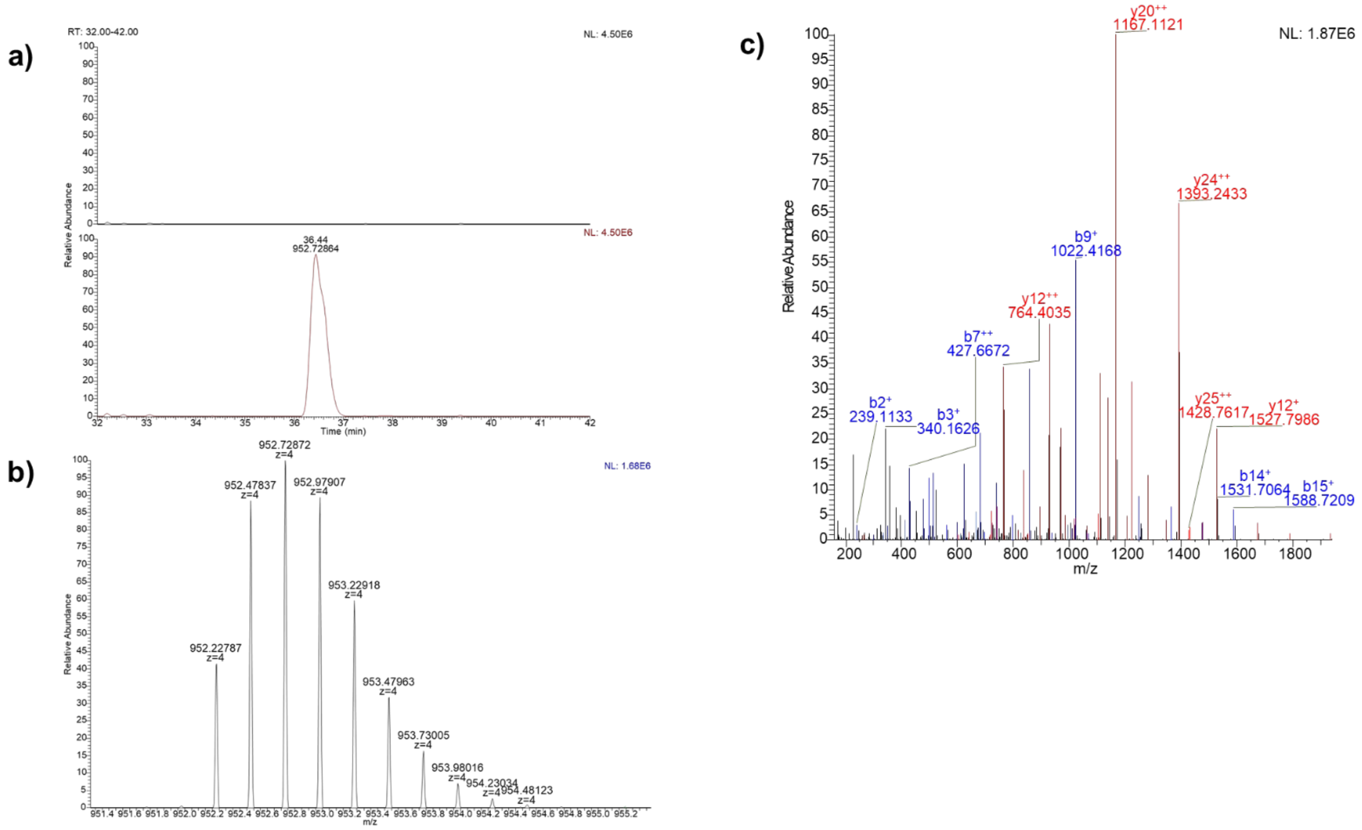

Figure S124~~17~~. The raw data of trastuzumab-Lys248-thiol (**4a**). a) Extracted ion chromatogram (XIC) of THTCPPCPAPELLGGPSVFLFPPKPK^{248}DTLMISR peptide with 3-(2-amino-2-oxo-ethyl) sulfanylpropionate of lysine modification (theoretical *m/z* 952.22942, 4+). The upper black trace shows trastuzumab digested sample and the lower red one shows trastuzumab-Lys248-thiol digested sample. The target conjugated peptide was only detected in trastuzumab-Lys248-thiol. b) The mass spectrum of the THTCPPCPAPELLGGPSVFLFPPKPK^{248}DTLMISR peptide with 3-(2-amino-2-oxo-ethyl) sulfanylpropionate of lysine modification peak shown in a). The measured *m/z* (952.22787, 4+) was in

agreement with the theoretical value of its precursor ion (952.22942, 4+). c) The product ion spectrum of the THTCPPCPAPELLGGPSVFLFPPKPK^{248}DTLMISR peptide with 3-(2-amino-2-oxo-ethyl) sulfanylpropionate of lysine modification. The spectrum matched with theoretical value were shown in blue (b-ion series) or red (y-ion series).

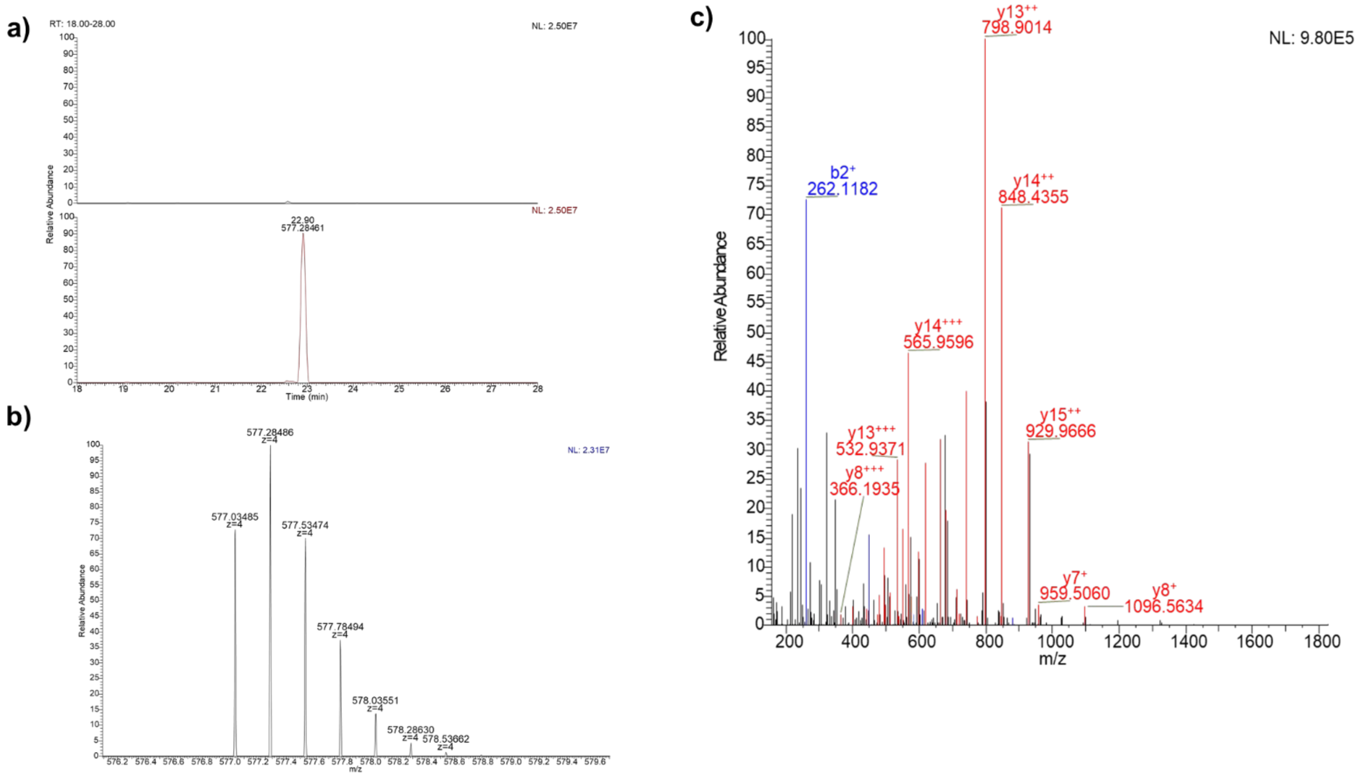

Figure S12518. The raw data of trastuzumab-Lys288-thiol (**4b**). a) Extracted ion chromatogram (XIC) of FNWYVDGVEVHNAK^{288}TKPR peptide with 3-(2-amino-2-oxo-ethyl) sulfanylpropionate of lysine modification (theoretical *m/z* 577.03557, 4+). The upper black trace shows trastuzumab digested sample and the lower red one shows trastuzumab-Lys288-thiol digested sample. The target conjugated peptide was only detected in trastuzumab-Lys288-thiol. b) The spectrum of the FNWYVDGVEVHNAK^{288}TKPR peptide with 3-(2-amino-2-oxo-ethyl) sulfanylpropionate of lysine modification peak shown in a). The measured *m/z* (577.03485, 4+) was in agreement with the theoretical value of its precursor ion (577.03557, 4+). c) The product ion spectrum of the FNWYVDGVEVHNAK^{288}TKPR peptide with 3-(2-amino-2-oxo-ethyl)

sulfanylpropionate of lysine modification. The spectrum matched with theoretical value were shown in blue (b-ion series) or red (y-ion series).

2-7 Structural analysis of FcRn/Fc protein

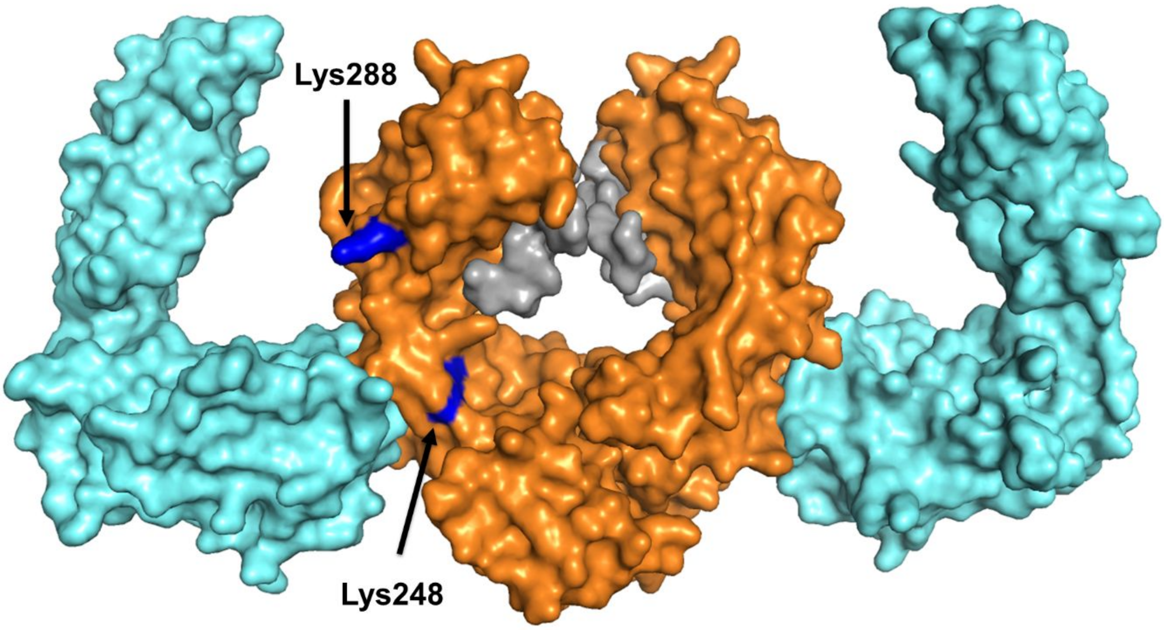

Figure S126~~19~~. Structure of FcRn/Fc protein; Fc region (orange color) and FcRn (skybule color), glycan (gray color), Lys248 and Lys288 (blue color).

2-8 In vivo biological evaluation of AJICAP-ADCs

2-8-1 Mouse xenograft study

No abnormal clinical signs and No remarkable body weight change was observed during test period.

Statistical analysis was shown in Table-S3.

Table S3. Adjusted p-values (ANOVA with posthoc Dunnett analysis) at 50 days.

Dunnett's multiple comparisons test	Mean Diff.	Adj. P Value
Vehicle vs. trastuzumab-K248-MMAE (**5a**) (5 mg/kg)	219.8	<0.0001
Vehicle vs. trastuzumab-K248-MMAE (**5a**) (2.5 mg/kg)	162.4	<0.0001
Vehicle vs. trastuzumab-K288-MMAE (**5b**) (5 mg/kg)	205.3	<0.0001
Vehicle vs. trastuzumab-K288-MMAE (**5b**) (2.5 mg/kg)	127.3	<0.0001

2-8-2 Rat PK study and safety study

No abnormal clinical signs and No remarkable body weight change was observed during test period.

2-8-3 Rat safety study

Clinical observations

In trastuzumab-Lys288-MMAE（**5b**） 80 mg/kg groups, all the rest animals showed no severe clinical signs and stayed alive during test period.

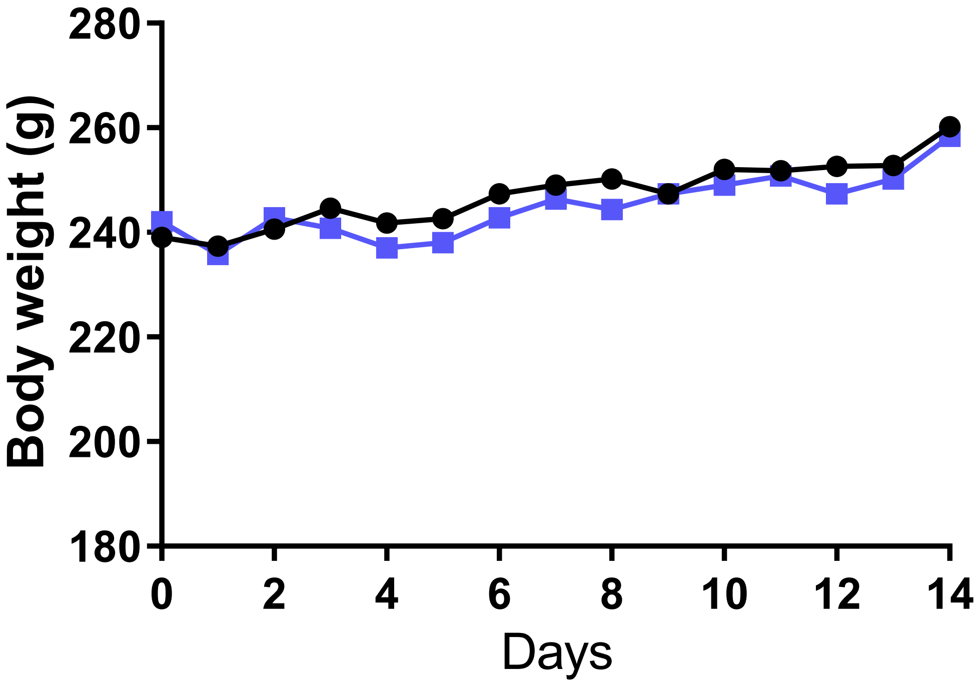

Figure S1207. Rat acute toxicity study measuring body weight change. (N = 5 female SD rats): black line: vehicle, blue line: 80 mg/kg of trastuzumab-Lys288-MMAE（**5b**）

2-9 Application to novel format antibody conjugates

2-9-1 Antibody-siRNA conjugate production

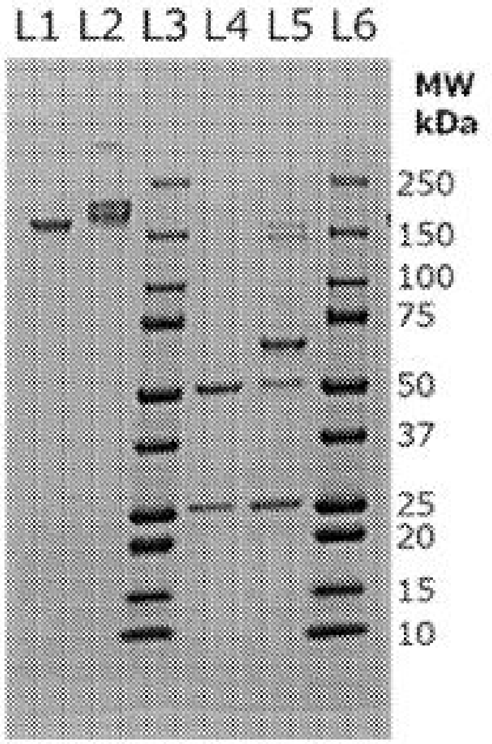

Figure S1281. SDS-PAGE analysis of antibody-siRNA conjugate (8); L1= trastuzumab (non-reduced), L2= antibody-siRNA conjugate (8) (non-reduced), L3= molecular maker, L4= trastuzumab (reduced), L5= antibody-siRNA conjugate (8) (reduced), L6= molecular maker

2-9-2 Antibody-protein conjugate production

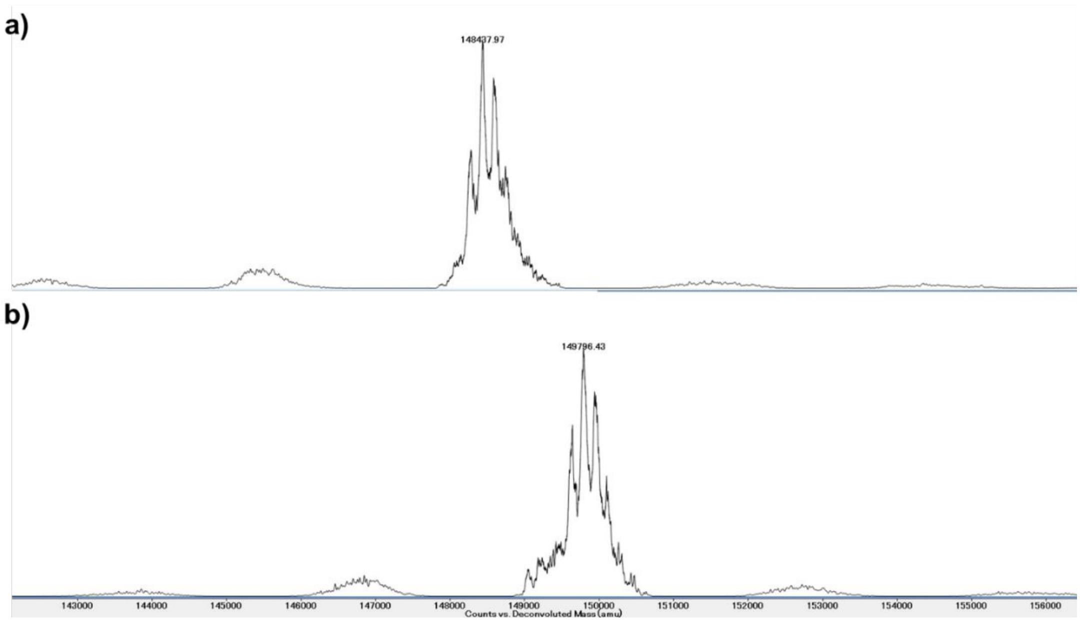

Figure S1292. Q-TOF MS analysis, a) trastuzumab-Lys248-thiol (**4a**), b) trastuzumab-Lys248-DBCO (**10**)

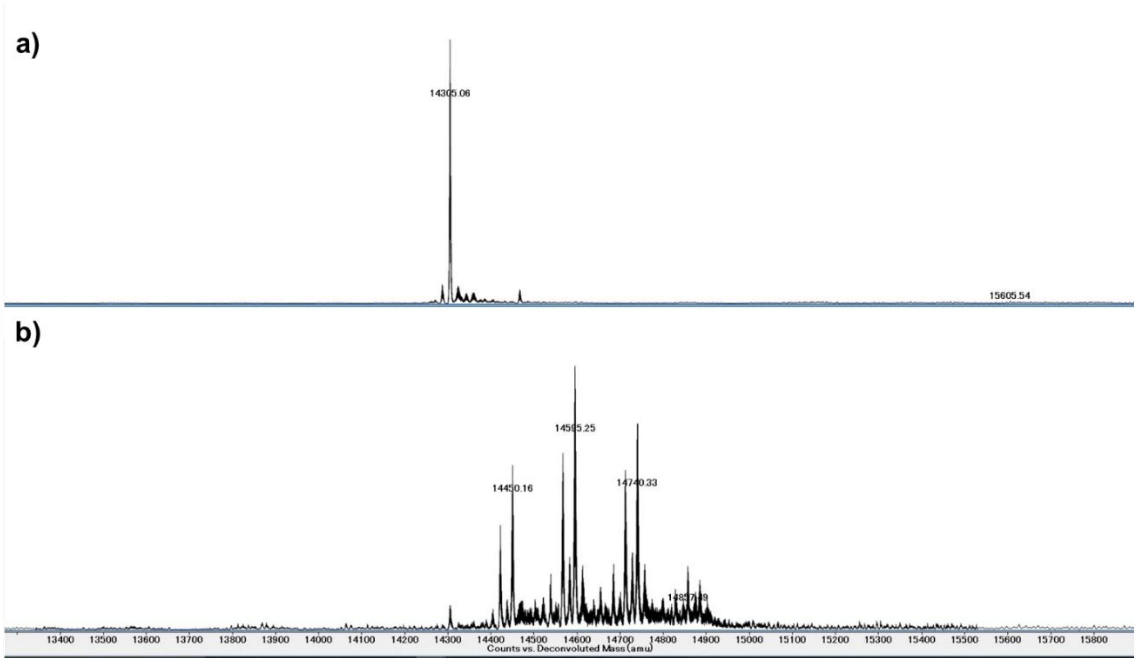

Figure S13023. Q-TOF MS analysis of model protein; a) lysozyme, b) azide modified lysozyme (**11**)

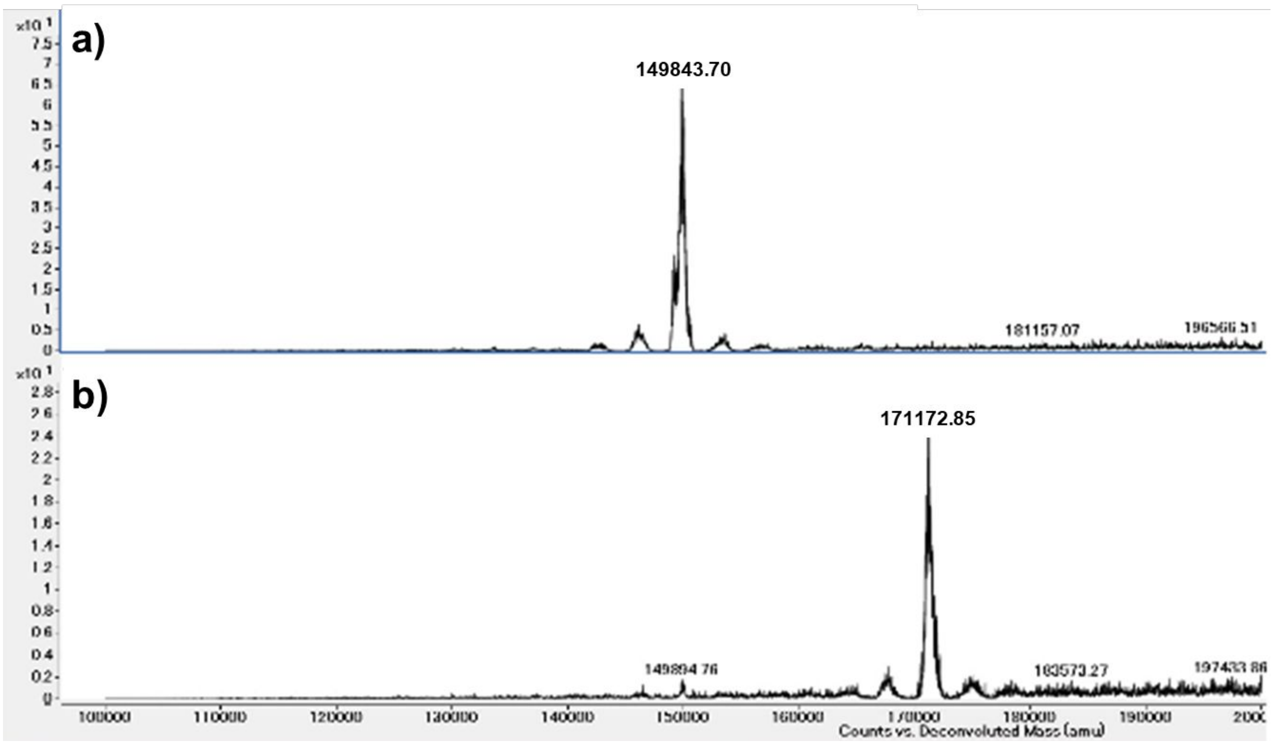

Figure S13124. Q-TOF MS analysis of Antibody-protein conjugate; a) trastuzumab-Lys248-DBCO (**10**), b) trastuzumab-Lys248-lysozyme conjugate (**12**)
